# Total Synthesis of (−)-Glionitrin A and B Enabled by an Asymmetric Oxidative Sulfenylation of Triketopiperazines

**DOI:** 10.1021/jacs.1c10364

**Published:** 2021-11-22

**Authors:** Nicolas R. Koning, Anders P. Sundin, Daniel Strand

**Affiliations:** Centre for Analysis and Synthesis, Department of Chemistry, Lund University, Box 124, SE-221 00 Lund, Sweden

## Abstract

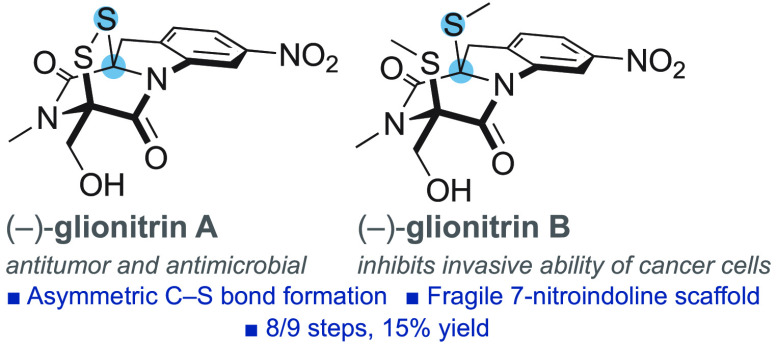

Asymmetric construction of dithiodiketopiperazines on otherwise achiral scaffolds remains a pivotal synthetic challenge encountered in many biologically significant natural products. Herein, we report the first total syntheses of (−)-glionitrin A/B and revise the absolute configurations. Emerging from the study is a novel oxidative sulfenylation of triketopiperazines that enables asymmetric formation of dithiodiketopiperazines on sensitive substrates. The concise route paves the way for further studies on the potent antimicrobial and antitumor activities of glionitrin A and the intriguing ability of glionitrin B to inhibit invasive ability of cancer cells.

The broad family of dithiodiketopiperazine (DTDKP) natural products draws interest from their inspiring molecular architectures and biologically significant antimalarial, antiviral, antibacterial, and cytotoxic properties.^1−5^ Numerous recent reports detail elegant advances in the synthesis of DTDKPs.^6−17^ Previous syntheses, however, all rely on substrate control for installing the stereochemistry of the DTDKP units. Asymmetric construction of this motif on otherwise achiral scaffolds has remained a critical challenge. It is typified by (−)-glionitrin A (**1**)^18^ and (−)-glionitrin B (**2**)^19^ where all stereochemistry resides in a unique 7-nitroindoline fused DTDKP motif (Figure 1A). Despite an intriguing origin and potent biological properties, no synthesis of glionitrin A or B has been reported. Both compounds were isolated by Kwon from a coculture of the bacterial strain *Sphingomonas* KMK-001 and the fungal strain *Aspergillus fumigatus* KMC-901, obtained from “extremely contaminated acid mine drainage” in an abandoned coal mine.^18,19^ Glionitrin A exhibits nanomolar activity against methicillin-resistant *Staphylococcus aureus* and antitumor activity in xenograft DU145 prostate cancer cells.^18,20^ Glionitrin B, by contrast, is nontoxic but inhibits the invasive ability of DU145 cancer cells suggesting utility in suppressing cancer metathesis and recurrence.^19^ The nature of the molecular interactions that underlie these remarkable effects remains an outstanding question and a practical supply of the glionitrins is much needed to support further studies. Here, we describe a mild and efficient asymmetric oxidative sulfenylation of triketopiperazines (TKPs), leading to the first total syntheses of (−)-glionitrin A/B, and revise the proposed absolute configurations.

**Figure 1:**
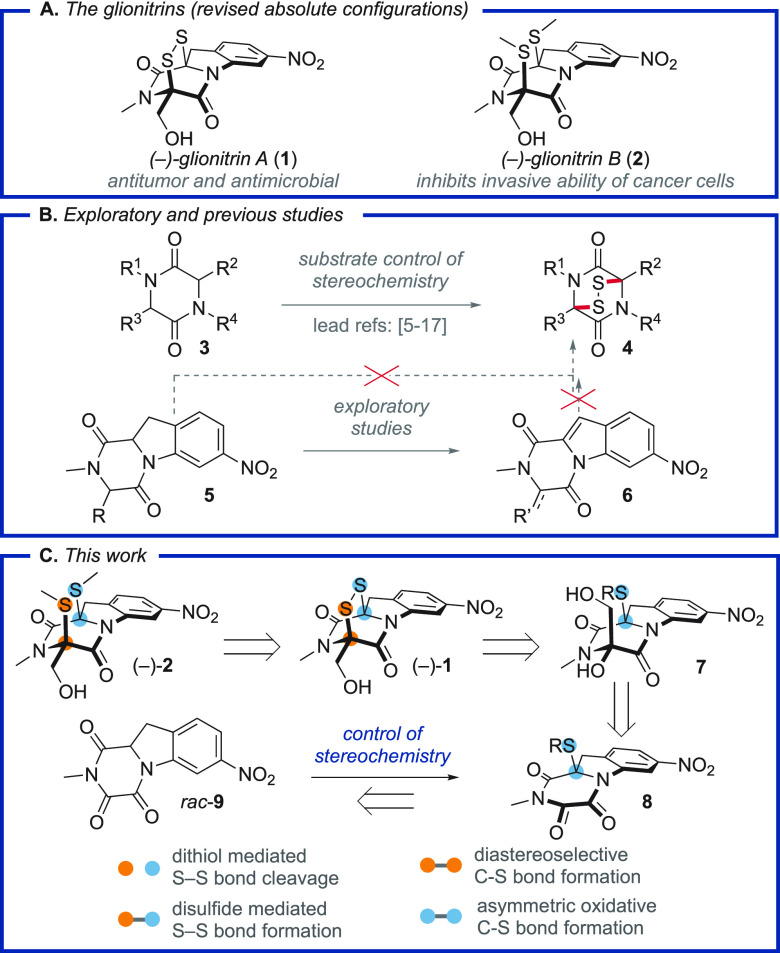
Overview and synthetic approach to glionitrin A/B.

Two principal considerations guided our synthetic approach. First, indoline derivatives like **5** are prone to aromatize to the corresponding indoles^6,21^ under the basic^16^ or oxidative^14,15^ conditions previously employed to elaborate diketopiperazines (DKPs) to the corresponding epidithiodiketopiperazines (ETPs) (Figure 1B). For the glionitrins, this problem is accentuated by the nitro-group at the indoline 7-position and proved prohibitive during our exploratory studies. Acidic conditions^22,23^ for sulfenylation of **6** were also evaluated but proved equally unproductive. Second, the C–S bonds must be introduced asymmetrically and the stereochemistry of the resulting thioaminals preserved throughout subsequent manipulations. A single chiral pool approach to (+)-hyalodendrin (**24**) provided precedence,^24^ but no general denovo asymmetric approach has been reported. This methodological gap is striking in light of the many biologically active natural products where this issue is encountered.^1^

We thus concluded that an asymmetric solution to the glionitrin-problem was contingent on developing a mild and most likely stepwise approach to the DTDKP core. To this end, we considered the low basicity of triketopiperazine (TKP) enolates^25^ as an entry to forge the first C–S bond by reaction with a suitable sulfur electrophile (Figure 1C).^26^ This would give a chiral intermediate **8** from which glionitrin A and B could be pursued. An asymmetric oxidative sulfenylation of a TKP has, to our knowledge, not been reported, but a nonstereoselective sulfenylation of *N*,*N*-dimethyltriketopiperazine was recently shown by Snaddon,^27^ and oxidation of TKPs was pioneered in ETP synthesis by Overman.^7,8^ Furthermore, the quinine catalyzed asymmetric sulfenylation of acyl-activated DKPs^28^ and catalytic enantioselective 1,4-addition of TKP enolates^29,30^ provided encouraging precedence.

In line with our hypothesis, we were delighted to obtain thioaminal **10a** in 75% yield (65:35 er) when reacting **9** with **13a** using quinine (**11**) as catalyst (Table 1). Unfortunately, extensive screening of chiral catalysts and conditions proved fruitless in pursuit of synthetically useful levels of enantioselectivity (Supporting Information, Table S14). The likely culprits are a lack of suitable basic sites in the substrate that can organize the presumed enolate-base ion pair, and limited steric differentiation proximal to the enolate. To address these issues we developed **13b**, a novel chiral variation of electrophile **13a**, accessible in one step from the corresponding thiol.^31^ Unlike **13a**, **13b** required strongly nucleophilic catalysts to efficiently participate in the reaction, a property that proved essential for selectivity. Systematic screening of conditions revealed that the matched pair of (*R*)-**13b** and (*R*)-**12b**^32^ (10 mol %) produced the (10a*R*)-**10b** isomer in 90:10 dr and 82% yield (see also Supporting Information, Table S4). The relative configuration of (10a*R*)-**10b** was assigned by single crystal X-ray diffraction (scXRD). The reaction was completed in ∼15 min at room temperature, did not require exclusion of air or moisture, and could be conducted on a gram scale. Separation of the minor diastereomer was also straightforwardly accomplished by chromatography.

**Table 1:**
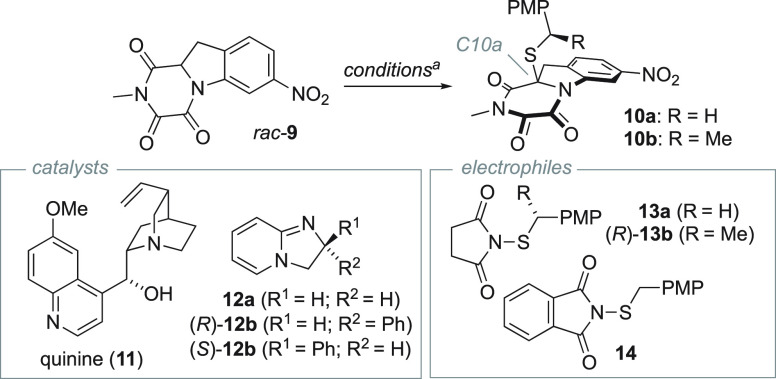
Optimization of the Asymmetric Sulfenylation of TKP **9**^a^

Entry	Electrophile	Catalyst (mol %)	Time	(C10a*R*):(C10a*S*)^b^	Product, Yield (%)^b^
1	**13a**	**11** (10)	15 h	65:35^c^	**10a**, 75
2	**14**	**11** (10)	16 h	50:50^c^	**10a**, 61
3	**13a**	(*R*)-**12b** (10)	15 min	57:43^c^	**10a**, 85
4	(*R*)-**13b**	**12a** (10)	15 min	60:40	**10b**, 83
5	(*R*)-**13b**	(*R*)-**12b** (10)	15 min	90:10	**10b**, 82
6	(*R*)-**13b**	(*S*)-**12b** (10)	15 min	38:62	**10b**, 87
7	(*R*)-**13b**	(*R*)-**12b** (20)	10 min	90:10	**10b**, 79
8^d^	(*R*)-**13b**	(*R*)-**12b** (10)	1 h	90:10	**10b**, 66

a *Rac*-**9** (0.1 mmol), electrophile (0.11 mmol), and catalyst. CH_2_Cl_2_ (2 mL).

b Determined by ^1^H NMR spectroscopy (internal standard for yields).

c Determined by enantioselective HPLC.

d Temperature = 0 °C. PMP = *p*-methoxyphenyl.

The rate difference between **11** and **12a**/**b** as catalysts aligns with *in situ* formation of an activated electrophilic intermediate (Figure 2). In agreement, the formation of **15** was detected by HRMS. Birman invoked a similar intermediate in acylation reactions with **12b**.^32^ To gain insight into the factors governing selectivity, we modeled the transition states (TSs) leading to each diastereomeric product by density functional theory (DFT). In good agreement with experimental data, the lowest found TSs favored (10a*R*)-**10b** over (10a*S*)-**10b** by Δ*G*_298_^‡^ = ∼1.9 kcal/mol. The model also clarified the role of the benzylic methyl group in **13b** for selectivity: this group rigidifies **15** conformationally and leads to well-defined TSs wherein the steering phenyl group of the catalyst reaches *toward* the incoming nucleophile. In doing so, it is capable of relaying interactions to distal parts of a substrate. In TS **17**, leading to the major diastereomer, the enolate is stabilized by an electrostatic interaction with a proton on the PMP ring. This interaction is absent in the TS leading to the minor diastereomer (Supporting Information, Figure S9). Moreover, there is distinguishing attractive electrostatic interaction in TS **17** between a proton on the catalyst phenyl ring and the substrate nitro-group. The TS models also reveal that the reaction is facilitated by buildup of a stabilizing edge-to-face interaction between the electron deficient pyridyl unit of the catalyst and the PMP group.

**Figure 2:**
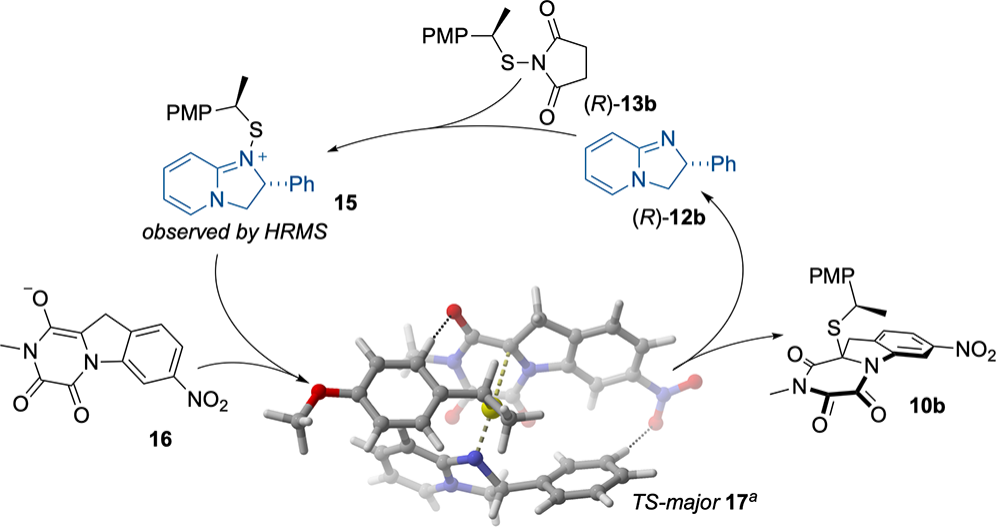
Plausible mechanism for the sulfenylation of TKP **9**. ^*a*^Modeled using the m06-2x functional and the 6-31G** basis set. Thin dotted lines highlight key electrostatic interactions.

The efficient formation of **10b** prompted us also to explore the scope of this reaction briefly. Six TKPs were selected based on their structural relation to one or more natural products (Table 2).

**Table 2:**
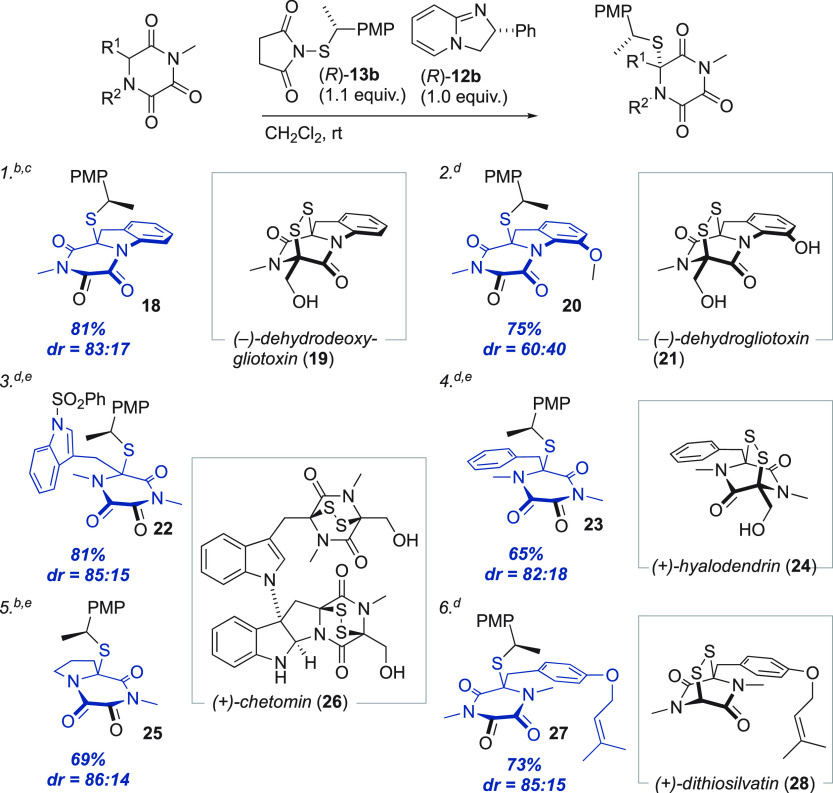
Scope of the Asymmetric Sulfenylation of TKPs^a^

a Isolated yields. Atoms mapping onto natural products are highlighted in blue.

b Relative configuration determined by scXRD.

c 0.1 equiv of **12b** used.

d Relative configuration assigned by analogy.

e (*S*)-**12b** and (*S*)-**13b** used.

Significantly, all evaluated substrates gave the desired sulfenylated products with synthetically useful levels of efficiency. Compared to **9**, longer reaction times and stoichiometric amounts of the base were needed to reach high conversions with most substrates. Methoxy substitution at the indoline 6-position gave reduced selectivity, presumably due to a steric interaction with the steering phenyl group of **12b**.

With a practical method to install the C10a stereocenter at hand, we turned to completing the syntheses of glionitrin A/B (Scheme 1). The first two steps from **29** to **30** were telescoped on a decagram scale and **30** collected by filtration in 88% yield. Indoline **30** could be cyclized to **9** with catalytic 1,8-diazabicyclo[5.4.0]undec-7-ene in 77% yield (see Supporting Information). The use of substoichiometric base was essential to suppress oxidative aromatization of the indoline unit.

**Scheme 1:**
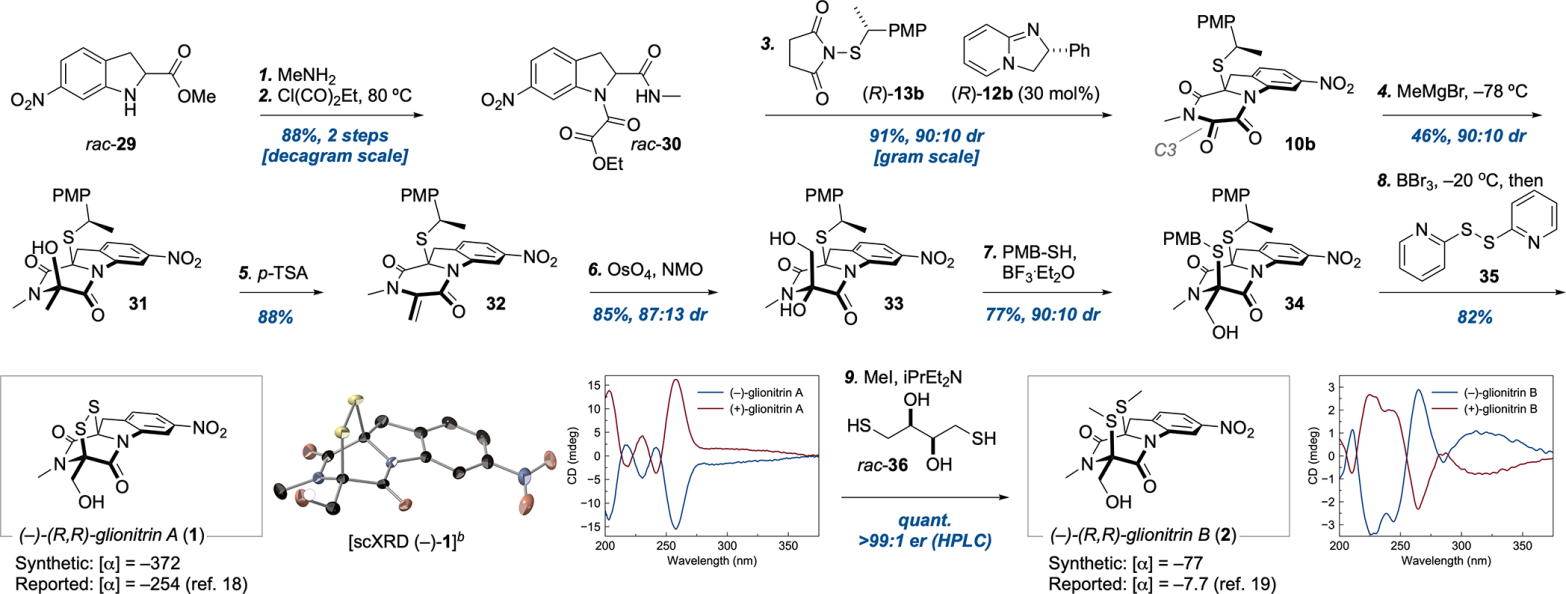
Total Synthesis of (−)-Glionitrin A/B Reagents and conditions: (1) methylamine (excess, aq., 40% (w/w)), 1 h, 91%; (2) ethyl-2-chloro-2-oxoacetate (4.0 equiv), PhMe, 80 °C, 1.5 h, 95%; (3) (*R*)-**13b** (1.1 equiv), (*R*)-**12b** (0.3 equiv), CH_2_Cl_2_, 15 min, 91%; (4) MeMgBr (1.2 equiv), THF, −78 °C, 1 h, 46%; (5) *p*-TsOH·H_2_O (1.0 equiv), CH_2_Cl_2_, 2 h, 88%; (6) OsO_4_ (0.1 equiv, 2.5% (w/w) in *tert*-BuOH), NMO (2.0 equiv), acetone/H_2_O, 16 h, 85%; (7) BF_3_·Et_2_O (20 equiv), 4-methoxy-α-toluenethiol (10 equiv), THF, 19 h, 77%; (8) BBr_3_ (2.5 equiv), CH_2_Cl_2_, −20 °C, 15 min; **35** (1.0 equiv), CH_2_Cl_2_, 5 min, 82%; (9) MeI (20 equiv), *rac*-**36** (2.0 equiv), iPrEt_2_N (3.0 equiv), CH_2_Cl_2_, 15 min, quant. Thermal ellipsoids shown at 30% probability. PMB = *p*-methoxybenzyl.

Significantly, the catalytic conditions also pointed to the possibility of performing the subsequent thiolation as part of a cascade, wherein **12b** would also trigger the annulation of **30** to the corresponding TKP. In practice, this proved effective: with a slightly increased catalyst loading (30 mol %), **30** was converted to **10b** on a gram scale with retained stereoselectivity in 91% yield—a substantial improvement over the stepwise approach.

Completion of the glionitrin framework then required the introduction of a hydroxymethyl unit at C3. After unsuccessfully screening conditions for direct olefination and oxymethylation of **10b**, we employed a three-step approach^7,27^ to reach **33**. Thus, a chemoselective methylation with MeMgBr in THF at −78 °C gave **31** in 46% yield and 90:10 dr. Acidic elimination of the alcohol then produced **32**, which was dihydroxylated to **33** in 75% yield over two steps. The three steps leading from **10b** to **33** could also be telescoped with a minor loss in overall efficiency (28% yield over three steps). Diagnostic NOEs revealed that the major diastereomer of alcohol **31** had a cis relationship of the exocyclic heteroatoms at C3/C10a, whereas the major diastereomer of diol **33** was trans. Under acidic conditions (*p*-TsOH·H_2_O), *trans*-**33** slowly equilibrated to the thermodynamically favored *cis*-isomer.

The second C–S bond was installed in 77% yield by treating **33** with an excess of 4-methoxy-α-toluenethiol and BF_3_·Et_2_O. Pleasingly, this addition occurred with a 90:10 kinetic preference for the desired *cis*-isomer **34**. At this junction, completion of glionitrin A left only deprotection of the thioethers and an oxidative closure of the disulfide bridge. Deprotection was accomplished with BBr_3_ at −20 °C, but a subsequent oxidation with commonly used iodine led to decomposition. In contrast, we found that a disulfide mediated closure^33^ using reagent **35**, previously not used in the context of DTDKP synthesis, effectively produced the desired annulation in 82% isolated yield and thus completed the synthesis of glionitrin A. The reported procedure for converting glionitrin A to B (MeI, pyridine, then NaBH_4_)^19^ was attempted but proved capricious in our hands. A mixture of dithiol **36**, MeI, and iPrEt_2_N, however, gave a clean rupture of the S–S bridge along with a surprisingly efficient double S-methylation that concluded the synthesis of glionitrin B in quantitative yield. The ring-opening of **1** with **36** was modeled by DFT as an isodesmic reaction and found to be favored by ΔΔ*G*_298_ = 2.8 kcal/mol.

Enantioselective HPLC analysis of synthetic glionitrin B confirmed a >99:1 er and thus that the final steps of the synthesis proceeded without racemization. The analytical data for synthetic (*S*,*S*)-glionitrin A/B were in agreement with those reported except for the optical rotations. These were reversed from (−) to (+) and of a lower magnitude. We therefore prepared the corresponding (−)-isomers of both compounds. The absolute configuration of synthetic (−)-glionitrin A was corroborated as *R*,*R* by scXRD analysis (Flack = 0.01(6)). Moreover, the CD-spectra of the (−)-(*R*,*R*) isomers displayed Cotton effects matching those reported for natural material. Accordingly, the absolute configuration of natural glionitrin A/B should be revised to *R*,*R*.

In conclusion, practical syntheses of (−)-glionitrin A/B were achieved in eight/nine steps and 15% overall yield from abundant **29**. The route can be executed in 7 days with only four/five purification steps in the sequence. On a salient note, chemical synthesis thus requires less than half the time needed to obtain glionitrin B by fermentation.^19^ The introduction of a mild oxidative sulfenylation of TKPs was critical for asymmetric construction of DTDKPs on the sensitive 7-nitro indoline-fused scaffold **9**. As evident from DFT models, the rate and stereoselectivity of this reaction reflect a finely tuned interplay between the substrate, catalyst, and electrophile. Still, the methodology was successfully extended to substructures of several related natural products including (+)-chetomin (**26**) and (+)-dithiosilvatin (**28**) that are yet to be reached by chemical synthesis. While synthetically viable, the selectivities with certain substrates leave room for improvement and we anticipate refinements in both catalyst and electrophile design. Such studies are underway and will be reported in due course.

## Supporting Information Available

Experimental procedures, characterization data for all new compounds, HPLC data, assignment of relative configuration for *cis*-**31**, *trans*-**33**, and *cis*-**34**, crystallographic information for **1**, **10b**, **13b**, **18**, and **25**, details of DFT investigations, and copies of ^1^H and ^13^C NMR spectra (PDF)
DFT geometries (XYZ)

## Supplementary Material

ja1c10364_si_001.pdf

ja1c10364_si_002.xyz

## Accession Codes

CCDC 2110183–2110187 contain the supplementary crystallographic data for this paper. These data can be obtained free of charge via www.ccdc.cam.ac.uk/data_request/cif, or by emailing data_request@ccdc.cam.ac.uk, or by contacting The Cambridge Crystallographic Data Centre, 12 Union Road, Cambridge CB2 1EZ, UK; fax: +44 1223 336033.
