# Supplementary material for: Total Synthesis of (−)-Glionitrin A and B Enabled by an Asymmetric Oxidative Sulfenylation of Triketopiperazines
Source: J Am Chem Soc. 2021 Nov 22;143(50):21218–22. doi: 10.1021/jacs.1c10364 (PMC8704193; doi:10.1021/jacs.1c10364)

Supporting Information

Total Synthesis of (–)-Glionitrin A and B Enabled by an Asymmetric Oxidative Sulfenylation of Triketopiperazines

Nicolas R. Koning, Anders P. Sundin, and Daniel Strand*

Centre for Analysis and Synthesis, Department of Chemistry, Lund University, Box 124, SE-221 00 Lund, Sweden

Index

I.	*General procedures*	*S3*
II.	*Experimental procedures and analytical data for* ***1-2**, **9**, **10a-b**, **13b**, **18**, **20**, **22-23**, **25**, **27**, **30-34**, **S1**, **S4**, **S6-S8**, **S10**, **S12**, **S15**, and **S17-S18***	*S5*
III.	*Assignment of the relative configuration of cis-**31**, trans-**33**, and cis-**34***	*S43*
IV.	*Single crystal X-ray diffraction (scXRD) analysis of (–)-**1**, (10aR)-**10b**, (R)-**13b**, (10aR)-**18**, and (2aR)-**25***	*S46*
V.	*Expanded optimization of the stereoselective sulfenylation of triketopiperazine rac-**9***	*S52*
VI.	*Reassignment of the absolute configuration of natural (–)-glionitrin A (**1**) and (–)-glionitrin B (**2**)*	*S56*
VII.	*Density Functional Theory calculations*	*S57*
VIII.	*Copies of ^{1}H and ^{13}C NMR spectra for* ***1-2**, **9**, **10a-b**, **13b**, **18**, **20**, **22-23**, **25**, **27**, **30-34**, **S1**, **S4**, **S6-S8**, **S10**, **S12**, **S15**, and **S17-S18***	*S59*

SAFETY STATEMENT: No unusual or unexpected safety issues were encountered in this work.

I. General procedures

Reaction and purification details: Unless otherwise stated, all reactions were conducted in air. Reactions that were conducted under an inert atmosphere were performed in glassware that was dried by evacuation followed by heating with a heat gun. The vessels were then backfilled with dry nitrogen or argon gas and allowed to cool to room temperature. Dry argon was supplied from a balloon and dry nitrogen from a manifold. Elevated temperatures were achieved with a thermostat-controlled heating plate and aluminum heating blocks. Unless otherwise stated, reactions with a total volume above 5 mL were conducted in round bottom flasks equipped with a magnetic stir bar. Reactions with a total volume below 5 mL were conducted in Biotage® microwave vessels equipped with a magnetic stir bar and sealed with a septum crimp cap.

Reagents and solvents: All reagents and solvents were bought from commercial suppliers and used as received unless otherwise stated. Dichloromethane, THF, and toluene were obtained from an MBraun MB-SPS 800 solvent purification system.

HPLC analysis: Reversed-phase HPLC analysis was performed on an Agilent 1100 system and normal-phase HPLC analysis was performed on a Shimadzu Prominance-i LC-2030C 3D system.

NMR spectroscopy: ^{1}H and ^{13}C (^{1}H decoupled) NMR spectroscopy data was collected on a Bruker Avance II 400 MHz (^{1}H 400 MHz; ^{13}C 101 MHz) equipped with a 5 mm BBOF Z-gradient probe or on a Bruker Avance NEO 600 MHz (^{1}H 600 MHz) equipped with a 5 mm QCI(P) H&F Z-gradient CryoProbe. Chemical shifts are reported in parts per million (ppm) and coupling constants (*J*) are given in hertz. All data was recorded in $CDCl_3$ and referenced to residual $CHCl_3$ (δ 7.26 ^{1}H; 77.16 ^{13}C), DMSO-d_6 and referenced to residual DMSO (δ 2.50 ^{1}H; 39.52 ^{13}C), or methanol-d_4 and referenced to residual MeOH (δ 3.31 ^{1}H; 49.00 ^{13}C). Multiplicities are denoted by singlet (s), doublet (d), doublet of doublets (dd), doublet of doublet

of doublets (ddd), triplet (t), apparent triplet (app. t), doublet of triplets (dt), quartet (q), doublet of quartets (dq), triplet of quintets (tquin.), and multiplet (m). Broad peaks are denoted by (br).

IR spectroscopy: IR spectra were recorded on a Bruker Alpha II spectrometer as thin films using the ATR attachment. Frequencies are reported in wavenumbers (cm^{-1}) and peaks denoted as strong (s), medium (m), weak (w), and broad (br).

Specific rotation: Optical rotations $[\alpha]_D^T$ were recorded at room temperature (~20 °C) using a Perkin Elmer model 341 polarimeter. D represents the sodium D line (589 nm). Concentrations (*c*) are reported in g/100mL.

Circular dichroism: Circular dichroism (CD) spectra were recorded on a JASCO J-715 spectropolarimeter. Samples were dissolved acetonitrile (*c* = 0.1 mg/mL). Raw data were processed using CDToolX (baseline subtraction). Graphics were produced with MagicPlot 3.0.1 using a moving average (20pt) smoothing function.

Mass spectrometry: HRMS data was obtained using an ESI-QTOF mass spectrometer (Waters Xevo-G2) in positive mode between m/z 50-1200, employing lockmass correction according to the manufacturer's instructions.

Chromatography: Thin layer chromatography (TLC) was performed using Merck 60 F_{254} silica gel bound to aluminum plates. The plates were visualized using UV light (254 nm), phosphomolybdic acid (PMA) stain (phosphomolybdic acid hydrate (10 g), EtOH (100 mL), potassium permanganate ($KMnO_4$) stain ($KMnO_4$ (1.5 g), K_2CO_3 (10 g), NaOH (20 g), H_2O (180 mL), or ninhydrin stain (ninhydrin (1.5 g, *n*-BuOH (100 mL), AcOH (3 mL)). Purification by column chromatography was performed using Merck 60 Å (40-63 µm particle size) silica or using the Biotage® Isolera™ One system.

*II. Experimental procedures and analytical data for **1-2, 9, 10a-b, 13b, 18, 20, 22-23, 25, 27, 30-34, S1, S4, S6-S8, S10, S12, S15,** and **S17-S18***

Enantiomeric series: The described procedures refer to synthesis of (–)-(*R*,*R*)-glionitrin A and B. For the syntheses of (+)-(*S*,*S*)-glionitrin A and B, identical procedures were used but with enantiomeric reagents where applicable. Racemic glionitrin B, used as a standard in enantioselective HPLC (see Table S7), was prepared from thioaminal *rac*-**10a** using the described procedures (not shown).

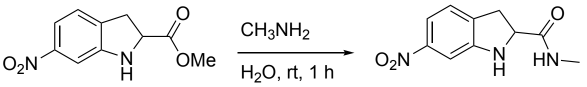

rac-**29** 91% *rac*-**S1**

***N*-Methyl-6-nitroindoline-2-carboxamide (*rac*-S1).** Methyl ester *rac*-**29**[1] (8.60 g, 38.7 mmol) was charged in a round bottom flask and methylamine (100 mL, aq., 40% (w/w)) was added. The resulting bright red suspension was stirred for 1 h and then filtered. The collected solid residue was washed with water, then with pentane, and finally dried *in vacuo* to give amide *rac*-**S1**.

Yield: 7.82 g (91%). Isolated as a red/orange solid. >95% pure by NMR.

R*f*: 0.20 in EtOAc. Stains yellow/brown with ninhydrin stain.

^{1}H NMR (DMSO-*d*$_6$, 400 MHz): δ 7.94 (br, q, *J* = 4.4 Hz, 1H), 7.43 (dd, *J* = 8.0, 2.0 Hz, 1H), 7.23-7.17 (m, 2H), 6.60 (d, *J* = 2.2 Hz, 1H), 4.33 (ddd, *J* = 10.4, 7.2, 2.2 Hz, 1H), 3.38 (dd, *J* = 17.6, 10.4, 1H), 3.02 (dd, *J* = 17.6, 7.2 Hz, 1H), 2.62 (d, *J* = 4.4 Hz, 3H) ppm.

^{13}C NMR (DMSO-*d*$_6$, 101 MHz): δ 172.9, 152.5, 147.8, 135.9, 124.2, 122.9, 101.4, 60.9, 33.9, 25.6 ppm.

[1] Prepared following: Lavrenov, S.N.; Lakatosh, S.A.; Lysenkova, L.N.; Korolev, A.M.; Preobrazhenskaya, M.N. *Synthesis* **2002**, *3*, 320-322.

FTIR (film): 3377 (br), 3305 (br), 2925 (w), 1656 (s), 1515 (s), 1339 (s), 738 (w) cm^{-1}.

HRMS-ESI (*m/z*): $[M + Na]^+$ Calcd for $C_{10}H_{11}N_3NaO_3$ 244.0698, Found 244.0692.

mp: 186-187 °C (obtained by recrystallization from EtOH/H_2O).

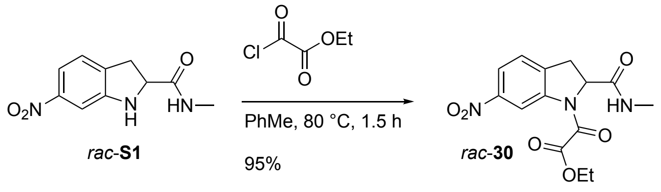

Ethyl 2-(2-(methylcarbamoyl)-6-nitroindolin-1-yl)-2-oxoacetate (*rac*-30). To a stirred suspension of amide *rac*-**S1** (15.6 g, 70.7 mmol) in toluene (250 mL) was added ethyl 2-chloro-2-oxoacetate (31.6 mL, 283 mmol) in one portion under an N_2 atmosphere. The resulting mixture was then heated to 80 °C. After 1.5 h, the reaction mixture was cooled to room temperature and filtered. The collected solid residue was washed with pentane and dried *in vacuo* to give oxoacetate *rac*-**30**.

Yield: 21.7 g (95%). Isolated as a beige solid. >95% pure by NMR.

R*f*: 0.29 in EtOAc.

^{1}H NMR (DMSO-*d*$_6$, 400 MHz): δ 8.79 (dd, J = 2.0 Hz, 1H), 8.28 (br, q, J = 4.6 Hz, 1H), 8.03 (dd, J = 8.4, 2.0 Hz, 1H), 7.54 (d, J = 8.4 Hz, 1H), 5.41 (dd, J = 11.0, 3.6 Hz, 1H), 4.26 (dq, J = 10.8, 7.2 Hz, 1H), 4.23 (dq, J = 10.8, 7.2 Hz, 1H), 3.74 (dd, J = 18.0, 11.0 Hz, 1H), 3.22 (dd, J = 18.0, 3.6 Hz, 1H), 2.60 (d, J = 4.6 Hz, 3H), 1.26 (t, J = 7.2 Hz, 3H) ppm. **^{13}C NMR (DMSO-*d*$_6$, 101 MHz):** δ 170.4, 160.1, 158.4, 147.0, 143.0, 138.8, 125.5, 120.4, 111.0, 62.4, 62.3, 34.2, 25.7, 13.6 ppm.

FTIR (film): 3313 (w), 1742 (w), 1666 (s), 1525 (w), 1336 (w), 1233 (w), 739 (w) cm^{-1}.

HRMS-ESI (*m/z*): $[M + Na]^+$ Calcd from $C_{14}H_{15}N_3NaO_6$ 344.0859, Found 344.0849.

mp: 198-201 °C (obtained by filtration, see above).

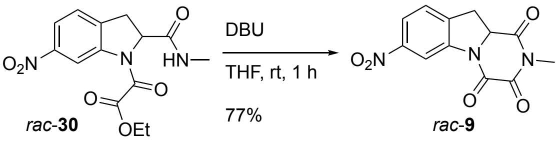

rac-**30** rac-**9**

2-Methyl-7-nitro-10,10a-dihydropyrazino[1,2-a]indole-1,3,4(2*H*)-trione (*rac*-9). To a stirred suspension of oxoacetate *rac*-**30** (4.18 g, 13.0 mmol) in THF (260 mL) was added 1,8-diazabicyclo[5.4.0]undec-7-ene (450 µL, 3.01 mmol) dropwise over 30 min under an argon atmosphere. The reaction mixture gradually turned into a clear red solution. After 30 min, NH_4Cl (100 mL, sat. aq.) was added in one portion. The organic layer was separated, dried over Na_2SO_4, filtered, and concentrated under reduced pressure. The resulting crude residue was purified by column chromatography (50-100% EtOAc/*n*-heptane) to give triketopiperazine *rac*-**9**.

Yield: 2.74 g (77%). Isolated as a yellow solid. >95% pure by NMR and a single spot by TLC.

R*f*: 0.24 in 50% EtOAc/*n*-heptane. Stains yellow with $KMnO_4$ stain.

^{1}H NMR (DMSO-*d*$_6$, 400 MHz): δ 8.61 (d, *J* = 2.4 Hz, 1H), 8.10 (dd, *J* = 8.2, 2.4 Hz, 1H), 7.67 (d, *J* = 8.2 Hz, 1H), 5.47 (dd, *J* = 10.8, 9.4 Hz, 1H), 3.62 (dd, *J* = 16.8, 9.4 Hz, 1H), 3.48 (ddd, *J* = 16.8, 10.8, 1.2 Hz, 1H), 3.14 (s, 3H) ppm.

^{13}C NMR (DMSO-*d*$_6$, 101 MHz): δ 168.2, 158.2, 150.9, 147.1, 141.4, 138.8, 126.1, 121.0, 110.0, 61.1, 31.5, 27.0 ppm.

FTIR (film): 2921 (w), 1687 (s), 1528 (w), 1350 (w), 1061 (w), 740 (w) cm^{-1}.

HRMS-ESI (*m*/*z*): $[M + Na]^+$ Calcd for $C_{12}H_9N_3NaO_5$ 298.0440, Found 298.0436.

mp: 206-208 °C (obtained by recrystallization from EtOAc/toluene). Decomposes, gas evolution observed.

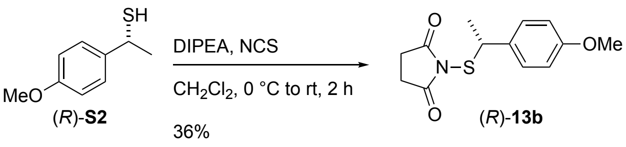

(*R*)-1-((1-(4-Methoxyphenyl)ethyl)thio)pyrrolidine-2,5-dione ((*R*)-13b). To a stirred solution of thiol (*R*)-**S2**[2] (590 mg, 3.51 mmol, 88:12 er) in CH_2Cl_2 (35 mL) was added *N*,*N*-diisopropylethylamine (610 µL, 3.51 mmol) under an N_2 atmosphere. The reaction mixture was cooled to 0 °C and *N*-chlorosuccinimide (469 mg, 3.51 mmol) was added in one portion. The cooling bath was removed after 30 min. After 1.5 h, the reaction mixture was concentrated under reduced pressure. The resulting crude residue was purified by column chromatography (33-75% EtOAc/*n*-heptane) to obtain *N*-thiosuccinimide (*R*)-**13b** as a white crystalline solid (483 mg, 52%). Two recrystallizations from EtOH (10 mL) gave (*R*)-**13b** of high enantiopurity.[3]

Yield: 327 mg (36%). Isolated as white needles. >95% pure by NMR and a single spot by TLC.

R*f*: 0.29 in 50% EtOAc/*n*-heptane. Stains dark green with PMA stain.

Optical rotation: $[\alpha]_D^{20}$: +251 (c = 0.1 in $CHCl_3$).

^{1}H NMR ($CDCl_3$, 400 MHz): δ 7.25-7.19 (m, 2H), 6.86-6.80 (m, 2H), 4.62 (q, J = 7.2 Hz, 1H), 3.78 (s, 3H), 2.70-2.52 (m, 4H), 1.55 (d, J = 7.2 Hz, 3H) ppm.

^{13}C NMR ($CDCl_3$, 101 MHz): δ 176.8, 159.7, 130.8, 129.4, 114.0, 55.4, 47.8, 28.5, 18.6 ppm.

FTIR (film): 2964 (w), 2933 (w), 1719 (s), 1608 (w), 1511 (w), 1298 (w), 1243 (s), 1177 (w), 1140 (s), 1028 (w), 1006 (w), 833 (w), 814 (w), 651 (w) cm^{-1}.

---

[2] Prepared in enantioenriched form following: Peschiulli, A.; Procuranti, B.; O'Connor, C.J.; Connon, S.J. *Nature Chem.* **2010**, 380-384.

[3] In an alternative procedure, thiol **S2** (of either enantiomer) was prepared in 69:31 er from the corresponding optically pure alcohol following: Hashiguchi, S.; Fuji, A.; Takehara, J.; Ikariya, T.; Noyori, R. *J. Am. Chem. Soc.* **1995**, *117*, 7562-7563; Corey, E.J.; Cimprich, K.A. *Tetrahedron Lett.* **1992**, *33*, 4099-4102. *N*-thiosuccinimide **13b** prepared via this route had an er of 69:31 and required four recrystallizations from EtOH to obtain >99:1 er.

HRMS-ESI (*m*/*z*): $[M + Na]^+$ Calcd for $C_{13}H_{15}NNaO_3S$ 288.0670, Found 288.0663.

mp: 125-127 °C (obtained by recrystallization from EtOH).

HPLC: Chiralpak® AS-RH column, 5μm silica-gel, MeCN/H_2O = 50/50, flow rate = 0.5 mL/min, λ = 254 nm. t_R = 7.99 min (minor), 8.62 min (major).

Enantiomeric ratio: >99:1

Table S1. HPLC chromatogram of *rac*-**13b**.

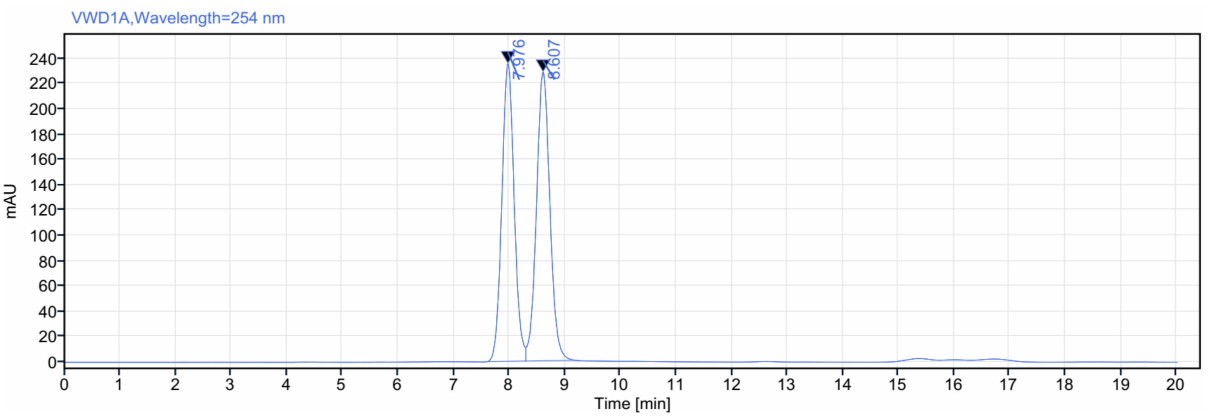

Peak	Retention time (min)	Height [mAU]	Area (%)
1	7.98	234.98	48.6
2	8.61	227.90	51.4

Table S2. HPLC chromatogram of (+)-(*R*)-**13b**.

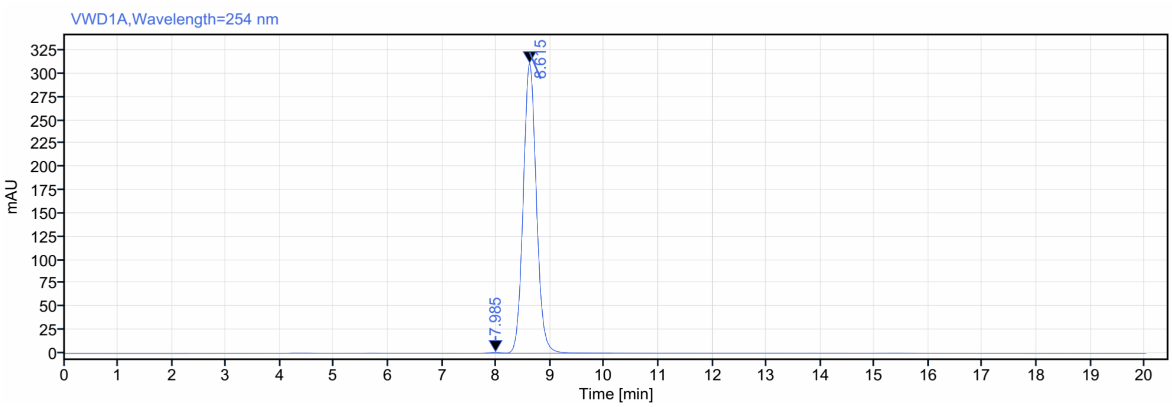

Peak	Retention time (min)	Height [mAU]	Area (%)
1	7.99	1.0	0.2
2	8.62	310.93	99.8

Table S3. HPLC chromatogram of (–)-(*S*)-**13b**.

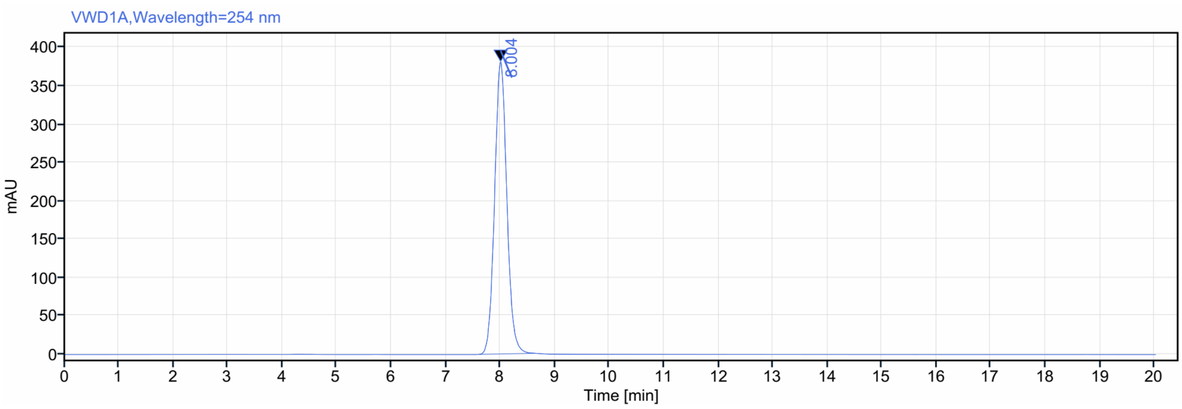

Peak	Retention time (min)	Height [mAU]	Area (%)
1	8.00	380.6	100
2	-	-	LoD

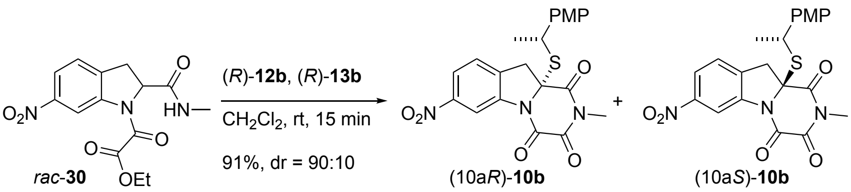

(*R*)-10a-(((*R*)-1-(4-Methoxyphenyl)ethyl)thio)-2-methyl-7-nitro-10,10a-dihydropyrazino[1,2-a]indole-1,3,4(2*H*)-trione ((10a*R*-10b). Method A. To a stirred suspension of oxoacetate *rac*-**30** (1.78 g, 5.50 mmol) and *N*-thiosuccinimide (*R*)-**13b** (1.59 g, 6.05 mmol) in CH_2Cl_2 (100 mL) was added a solution of catalyst (*R*)-**12b**[4] (324 mg, 1.65 mmol) in CH_2Cl_2 (10 mL) in one portion. A clear, orange solution gradually formed. After 15 min, HCl (100 mL, aq., 1.0 M) was added in one portion. The organic layer was separated and the aqueous layer was extracted with CH_2Cl_2 (50 mL). The combined organic extracts were passed through a phase separator and concentrated under reduced pressure. The resulting crude residue was purified by column chromatography (25% EtOAc/*n*-heptane) to give thioaminal (10a*R*)-**10b** as a single detected diastereomer along with a second fraction containing diastereomer (10a*S*)-**10b**.

Yield: Combined yield (10a*R*)-**10b** and (10a*S*)-**10b**: (2.20 g, 91%); Yield for (10a*R*)-**10b**: (1.95 g, 80%). Isolated as white/yellow crystalline solid. >95% pure by NMR and a single spot by TLC.

Selectivity: Measured by ^{1}H NMR spectroscopy of the crude residue. (10a*R*)-**10b** (δ = 1.44 ppm) / (10a*S*)-**10b** (δ = 1.38 ppm) = 90:10.

[4] Prepared following: Birman, V.B.; Uffman, E.W.; Jiang, H.; Li, X.; Kilbane, C.J. *J. Am. Chem. Soc.* **2004**, *126*, 12226-12227.

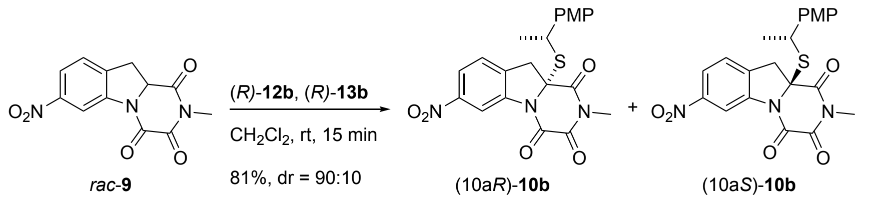

Method B. To a stirred suspension of triketopiperazine *rac*-**9** (1.27 g, 5.00 mmol) and *N*-thiosuccinimide (*R*)-**13b** (1.59 g, 5.50 mmol) in CH_2Cl_2 (100 mL) was added catalyst (*R*)-**12b** (98.0 mg, 500 µmol) in one portion. A clear, orange solution gradually formed. After 15 min, HCl (100 mL, aq., 1.0 M) was added in one portion. The organic layer was separated and the aqueous layer was extracted with CH_2Cl_2 (2 × 50 mL). The combined organic extracts were passed through a phase separator and concentrated under reduced pressure. The resulting crude residue was purified by column chromatography (25-50% EtOAc/*n*-heptane) to give a mixture of thioaminals (10a*R*)-**10b** and (10a*S*)-**10b**.

Yield: Combined yield (10a*R*)-**10b** and (10a*S*)-**10b**: (1.78 g, 81%). >95% pure by NMR.

Selectivity: Measured by ^{1}H NMR spectroscopy of the crude residue. (10a*R*)-**10b** (δ = 1.44 ppm) / (10a*S*)-**10b** (δ = 1.38 ppm) = 90:10.

Major diastereomer (10a*R*)-**10b**:

R*f*: 0.46 in 50% EtOAc/*n*-heptane. Stains dark green with PMA stain.

Optical rotation: $[\alpha]_D^{20}$: +155 (*c* = 0.1 in $CHCl_3$).

^{1}H NMR ($CDCl_3$, 400 MHz): δ 8.62 (d, *J* = 2.0 Hz, 1H), 8.12 (dd, *J* = 8.4, 2.0 Hz, 1H), 7.47 (d, *J* = 8.4 Hz, 1H), 7.02 (m, 2H), 6.78 (m, 2H), 3.99 (q, *J* = 7.6 Hz, 1H), 3.92 (dd, *J* = 17.8, 0.8 Hz, 1H), 3.80 (s, 3H), 3.60 (d, *J* = 17.8 Hz, 1H), 3.07 (s, 3H), 1.44 (d, *J* = 7.6 Hz, 3H) ppm.

^{13}C NMR ($CDCl_3$, 101 MHz): δ 166.6, 159.4, 156.6, 149.8, 148.4, 139.8, 135.1, 133.6, 128.1, 125.8, 122.2, 114.4, 112.8, 73.7, 55.5, 45.3, 41.0, 28.0, 25.2 ppm.

FTIR (film): 2966 (w), 1748 (w), 1693 (s), 1608 (w), 1527 (w), 1511 (w), 1483 (w), 1406 (w), 1350 (w), 1330 (w), 1313 (w), 1248 (w), 1177 (w), 1064 (w), 1030 (w), 910 (w), 833 (w), 738 (w), 648 (w) cm^{-1}.

HRMS-ESI (*m*/*z*): $[M + Na]^+$ Calcd for $C_{21}H_{19}N_3NaO_3S$ 464.0820, Found 464.0882.

mp: 172-174 °C (obtained by recrystallization from EtOH).

Minor diastereomer (10a*S*)-**10b**:

R_f: 0.51 in 50% EtOAc/*n*-heptane. Stains dark green with PMA stain.

Optical rotation: $[\alpha]_D^{20}$: +83 (c = 0.1 in $CHCl_3$).

^{1}H NMR ($CDCl_3$, 400 MHz): δ 8.86 (d, J = 2.4 Hz, 1H), 8.12 (dd, J = 8.4, 2.4 Hz, 1H), 7.35 (d, J = 8.4 Hz, 1H), 7.21-7.14 (m, 2H), 6.91-6.82 (m, 2H), 3.98 (q, J = 7.2 Hz, 1H), 3.82 (s, 3H), 3.66 (d, J = 17.6 Hz, 1H), 3.28 (d, J = 17.6 Hz, 1H), 3.00 (s, 3H), 1.38 (d, J = 7.2 Hz, 3H) ppm.

^{13}C NMR ($CDCl_3$, 101 MHz): δ 166.6, 159.4, 156.1, 150.0, 148.5, 140.0, 135.7, 134.1, 127.9, 125.6, 122.4, 114.6, 113.1, 74.0, 55.6, 45.2, 40.0, 27.9, 24.7 ppm.

FTIR (film): 2965 (w), 1748 (w), 1692 (s), 1608 (w), 1528 (m), 1511 (m), 1483 (w), 1439 (w), 1406 (w), 1351 (m), 1331 (m), 1250 (m), 1177 (w), 1064 (w), 1030 (w), 833 (w), 738 (w) cm^{-1}.

HRMS-ESI (*m*/*z*): $[M + Na]^+$ Calcd for $C_{21}H_{19}N_3NaO_3S$ 464.0892, Found 464.0893.

mp: 160-162 °C (obtained by recrystallization from EtOH).

Table S4. Optimization of the asymmetric sulfenylation of TKP **9** (continued from Table 1).[a]

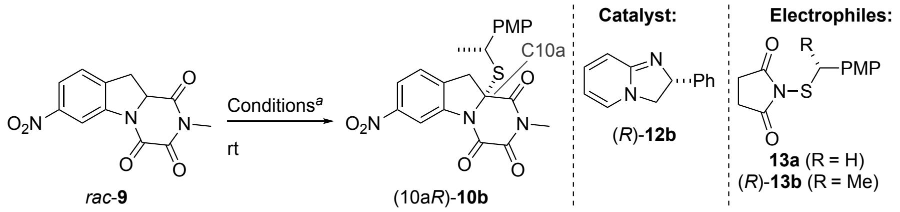

Entry	Electrophile	Catalyst (mol%)	Solvent	Time	(10a*R*/10a*S*)[b]	Product, Yield (%)[b]
1	**13a**[5]	-	CH_2Cl_2	16 h	-	n.r.[c]
2	(*R*)-**13b**	-	CH_2Cl_2	16 h	-	n.r.[c]
3	(*R*)-**13b**	Et_3N (100)	CH_2Cl_2	16 h	51:49	**10b**, 12
4	(*R*)-**13b**	DBU (20)	CH_2Cl_2	1 h	60:40	**10b**, 27
5	(*R*)-**13b**	(*R*)-**12b** (100)	CH_2Cl_2	15 min	89:11	**10b**, 65
6	(*R*)-**13b**	(*R*)-**12b** (50)	CH_2Cl_2	15 min	90:10	**10b**, 75
7	(*R*)-**13b**	(*R*)-**12b** (20)	CH_2Cl_2	15 min	90:10	**10b**, 84
8	(*R*)-**13b**	(*R*)-**12b** (5)	CH_2Cl_2	15 min	87:13	**10b**, 30
9	(*R*)-**13b**	(*R*)-**12b** (10)	$CHCl_3$	15 min	89:11	**10b**, 52
10	(*R*)-**13b**	(*R*)-**12b** (10)	Toluene	15 min	n.d.[d]	**10b**, 9
11	(*R*)-**13b**	(*R*)-**12b** (10)	THF	15 min	85:15	**10b**, 74
12	(*R*)-**13b**	(*R*)-**12b** (10)	MeCN	15 min	90:10	**10b**, 68
13	(*R*)-**13b**	(*R*)-**12b** (10)	MeOH	15 min	n.d.[d]	**10b**, 15

a) Rac-**9** (0.1 mmol), electrophile (0.11 mmol), and catalyst were stirred in the indicated solvent (2 mL) at the indicated temperature for the indicated time; b) Determined by ^1H NMR spectroscopy (internal standard for yields); c) No reaction. d) Not determined. PMP = *p*-methoxyphenyl, TKP = triketopiperazine.

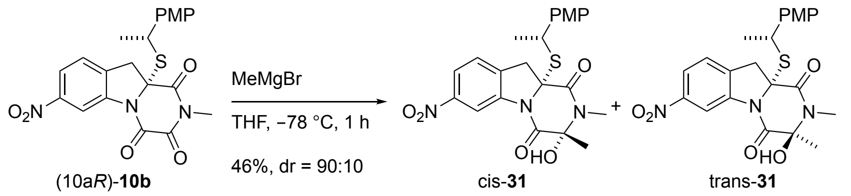

(3*R*,10a*R*)-3-Hydroxy-10a-(((*R*)-1-(4-methoxyphenyl)ethyl)thio)-2,3-dimethyl-7-nitro-2,3,10,10a-tetrahydropyrazino[1,2-a]indole-1,4-dione (cis-31). To a stirred solution of thioaminal (10a*R*)-**10b** (1.69 g, 3.83 mmol) in THF (40 mL) at –78 °C was added MeMgBr (1.53 mL, 3.0 M in Et_2O) dropwise over 5 min under an N_2 atmosphere. A bright yellow/orange solution formed. After 1 h, NH_4Cl (40 mL, sat. aq.) was added in one portion and the cooling bath was removed. After a further 15 min, the organic layer was separated, and the aqueous layer extracted with CH_2Cl_2 (3 × 50 mL). The combined organic extracts were dried over Na_2SO_4, filtered, and concentrated under reduced pressure. The resulting crude residue was

[5] Prepared following: Shunichi, K.; Toyokazu, H.; Nakabayashi, T.; Masayuki, H. *Bull. Chem. Soc. Jpn,* **1975**, *48*, 2993-2994.

purified by column chromatography (30-50% EtOAc/*n*-heptane) to give a mixture of alcohol cis-**31** and trans-**31**. Analytically pure cis-**31** was obtained by heating the obtained mixture of cis-**31** and trans-**31** in 50:50 EtOH/H_2O and collecting the precipitate that formed after cooling to room temperature.

Yield: Combined yield cis-**31** and trans-**31**: (802 mg, 46%). Isolated as yellow foam. Analytically pure cis-**31** was isolated as off-white solid. >95% pure by NMR.

Selectivity: Measured by ^{1}H NMR spectroscopy of the crude residue. cis-**31** (δ = 8.87 ppm) / trans-**31** (δ = 8.81 ppm) = 90:10.

Major diastereomer cis-**31**:

R*f*: 0.27 in 50% EtOAc/*n*-heptane. Stains dark green with PMA stain.

Optical rotation: $[\alpha]_D^{20}$: +65 (*c* = 0.5 in $CHCl_3$).

^{1}H NMR ($CDCl_3$, 400 MHz): δ 8.87 (d, *J* = 2.1 Hz, 1H), 8.06 (dd, *J* = 8.2, 2.1 Hz, 1H), 7.36 (d, *J* = 8.2 Hz, 1H), 7.12-7.04 (m, 2H), 6.82-6.74 (m, 2H), 4.83 (br. s, 1H), 4.21 (q, *J* = 7.2 Hz, 1H), 4.01 (dd, *J* = 18.2, 1.5 Hz, 1H), 3.79 (s, 3H), 3.63 (d, *J* = 18.2 Hz, 1H), 2.91 (s, 3H), 1.76 (s, 3H), 1.60 (d, *J* = 7.2 Hz, 3H) ppm.

^{13}C NMR ($CDCl_3$, 101 MHz): δ 165.3, 163.5, 159.2, 148.4, 141.1, 135.4, 134.2, 128.3, 125.5, 121.3, 114.2, 112.2, 85.7, 72.9, 55.5, 45.2, 42.3, 28.2, 25.0, 23.1 ppm.

FTIR (film): 3358 (br), 2964 (m), 1691 (m), 1672 (m), 1607 (m), 1526 (m), 1512 (m), 1481 (m), 1432 (m), 1392 (m), 1348 (m), 1247 (m), 1177 (m), 1130 (m), 1109 (m), 1030 (m), 907 (m), 832 (m), 738 (m) cm^{-1}.

HRMS-ESI (*m*/*z*): [M + Na^+] Calcd for $C_{22}H_{23}N_3NaO_6S$ 480.1205, Found 480.1208.

mp: 161-162 °C (obtained by trituration from EtOH/H_2O).

Minor diastereoisomer trans-**31**:

^{1}H NMR ($CDCl_3$, 400 MHz): 8.81 (d, J = 2.4 Hz, 1H), 8.09 (dd, J = 8.4 Hz, 1H), 7.35 (d, J = 8.4, 2.4 Hz, 1H), 7.13-7.08 (m, 2H), 6.83-6.78 (m, 2H), 4.42 (q, J = 7.2 Hz, 1H), 3.95 (d, J = 17.6 Hz, 1H), 3.78 (s, 3H), 3.67 (d, J = 17.6 Hz, 1H), 2.84 (s, 3H), 1,78 (s, 3H), 1.55 (d, J = 7.2 Hz, 3H) ppm. The signal from the tertiary hydroxyl proton could not be located.

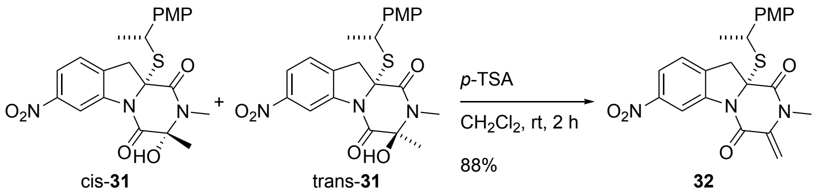

(*R*)-10a-(((*R*)-1-(4-Methoxyphenyl)ethyl)thio)-2-methyl-3-methylene-7-nitro-2,3,10,10a-tetrahydropyrazino[1,2-a]indole-1,4-dione (32). To a stirred solution of alcohols cis-**31** and trans-**31** (502 mg, 1.06 mmol) in CH_2Cl_2 (10 mL) was added *p*-TsOH·H_2O (201 mg, 1.06 mmol) in one portion. After 2 h, $NaHCO_3$ (10 mL, sat. aq.) was added in one portion. The mixture was passed through a phase separator and the filtrate was concentrated under reduced pressure. The resulting crude residue was purified by column chromatography (25-33% EtOAc/*n*-heptane) to give olefin **32**.

Yield: 411 mg (88%). Isolated as yellow foam. >95% pure by NMR and a single spot by TLC.

R_f: 0.43 in 50% EtOAc/*n*-heptane. Stains dark green with PMA stain.

Optical rotation: $[\alpha]_D^{20}$: +213 (c = 0.1 in $CHCl_3$).

^{1}H NMR ($CDCl_3$, 400 MHz): δ 8.75 (d, J = 2.0 Hz, 1H), 8.04 (dd, J = 8.4, 2.0 Hz, 1H), 7.37 (d, J = 8.4 Hz, 1H), 7.06 (m, 2H), 6.76 (m, 2H), 5.92 (d, J = 1.6 Hz, 1H), 5.04 (d, J = 1.6 Hz, 1H), 4.07 (q, J = 7.2 Hz, 1H), 3.92 (dd, J = 17.8, 1.2 Hz, 1H), 3.79 (s, 3H), 3.55 (d, J = 17.8 Hz, 1H), 2.97 (s, 3H), 1.43 (d, J = 7.2 Hz, 3H) ppm.

^{13}C NMR ($CDCl_3$, 101 MHz): δ 163.2, 159.0, 157.1, 148.3, 141.1, 138.5, 135.7, 135.0, 128.1, 125.4, 121.1, 113.9, 112.1, 105.6, 73.2, 55.5, 44.1, 41.7, 30.6, 25.3 ppm.

FTIR (film): 2966 (w), 2925 (w), 1689 (s), 1610 (w), 1526 (w), 1511 (w), 1482 (w), 1403 (w), 1348 (s), 1248 (w), 1176 (w), 1031 (w), 892 (w), 832 (w), 739 (w) cm^{-1}.

HRMS-ESI (*m/z*): $[M + Na]^+$ Calcd for $C_{22}H_{21}N_3NaO_5S$ 462.1100, Found 462.1099.

mp: 170-173 °C (obtained by concentration from EtOAc/*n*-heptane).

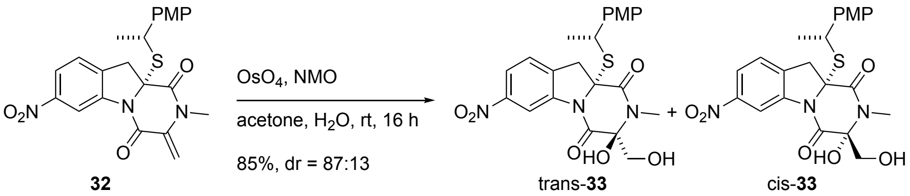

(3*S*,10a*R*)-3-Hydroxy-3-(hydroxymethyl)-10a-(((*R*)-1-(4-methoxyphenyl)ethyl)thio)-2-methyl-7-nitro-2,3,10,10a-tetrahydropyrazino[1,2-a]indole-1,4-dione (trans-33). To a stirred suspension of olefin **32** (411 mg, 935 µmol) in a 75:25 mixture of acetone and H_2O (13 mL) was added OsO_4 (950 µL, 2.5% (w/w) in *tert*-BuOH) in one portion followed by addition of 2-methylpyridine *N*-oxide (332 mg, 1.87 mmol) in one portion. After 16 h, the reaction was added to a 50:50 mixture of EtOAc and H_2O (100 mL). The organic layer was separated and the aqueous layer was extracted with EtOAc (2 × 50 mL). The combined organic extracts were dried over Na_2SO_4, filtered, and concentrated under reduced pressure. The resulting crude residue was purified by column chromatography (50% EtOAc/*n*-heptane) to give a mixture of diol cis-**33** and trans-**33**. Analytically pure trans-**33** was obtained by column chromatography (50% EtOAc/*n*-heptane).

Yield: Combined yield cis-**33** and trans-**33**: (375 mg, 85%). Isolated as a yellow foam. >95% pure by NMR.

Selectivity: Measured by ^1H NMR spectroscopy of the crude residue. trans-**33** (δ = 8.79 ppm) / cis-**33** (δ = 8.83 ppm) = 87:13.

Major diastereomer trans-**33**:

R_f: 0.38 in 75% EtOAc/*n*-heptane. Stains dark green with PMA stain.

Optical rotation: $[\alpha]_D^{20}$: +41 (c = 0.5 in $CHCl_3$).

^{1}H NMR ($CDCl_3$, 400 MHz): δ 8.77 (d, J = 2.1 Hz, 1H), 8.10 (dd, J = 8.3, 2.1 Hz, 1H), 7.39 (d, J = 8.3 Hz, 1H), 7.15-7.08 (m, 2H), 6.82-6.76 (m, 2H), 4.59 (br. s, 1H), 4.34 (d, J = 12.1 Hz, 1H), 4.24 (q, J = 7.3 Hz, 1H), 3.98-3.90 (m, 1H), 3.79 (s, 3H), 3.73 (d, J = 12.1 Hz, 1H), 3.67 (d, J = 18.0 Hz, 1H), 2.84 (s, 3H), 1.55 (d, J = 7.3 Hz, 3H) ppm. The signal from the primary hydroxyl group proton could not be located.

^{13}C NMR ($CDCl_3$, 101 MHz): δ 165.5, 163.4, 159.0, 148.2, 140.5, 136.2, 135.1, 128.4, 125.8, 121.9, 113.9, 113.0, 85.1, 73.1, 64.8, 55.5, 44.5, 43.2, 28.5, 25.2 ppm.

FTIR (film): 3422 (br), 2963 (m), 2929 (m), 1664 (m), 1607 (m), 1527 (m), 1512 (m), 1433 (m), 1387 (m), 1349 (m), 1248 (m), 1177 (m), 1117 (m), 1064 (m), 1032 (m), 910 (m), 832 (m), 738 (m) cm^{-1}.

HRMS-ESI (*m/z*): $[M + Na]^+$ Calcd for $C_{22}H_{23}N_3NaO_7S$ 496.1144, Found 496.1154.

mp: 94-97 °C (obtained by concentration from EtOAc/*n*-heptane).

Minor diastereoisomer cis-**33**:

^{1}H NMR ($CDCl_3$, 400 MHz): 8.81 (d, J = 2.4 Hz, 1H), 8.08 (dd, J = 8.4, 2.4 Hz, 1H), 7.38 (d, J = 8.4 Hz, 1H), 7.10-7.05 (m, 2H), 6.82-6.76 (m, 2H), 4.42 (d, J = 12.4 Hz, 1H), 4.22 (q, J = 7.2 Hz, 1H), 4.03 (dd, J = 18.0, 0.8 Hz, 1H), 3.83 (d, J = 12.4 Hz, 1H), 3.79 (s, 3H), 3.62 (d, J = 18.0 Hz, 1H), 3.00 (s, 3H), 1.60 (d, J = 7.2 Hz, 3H) ppm. The signals from the tertiary and primary hydroxyl protons could not be located.

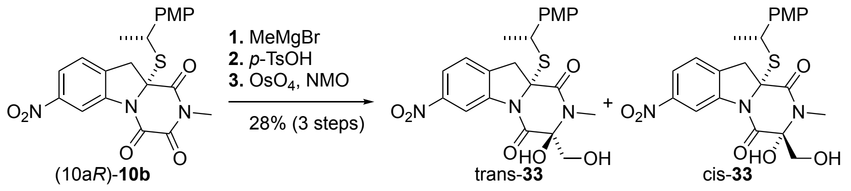

(3*S*,10a*R*)-3-Hydroxy-3-(hydroxymethyl)-10a-(((*R*)-1-(4-methoxyphenyl)ethyl)thio)-2-methyl-7-nitro-2,3,10,10a-tetrahydropyrazino[1,2-a]indole-1,4-dione (trans-33).

Telescoping procedure (three steps): To a stirred solution of thioaminal (10a*R*)-**10b** (2.21 g, 5.00 mmol) in THF (50 mL) at –78 °C was added MeMgBr (2.00 mL, 3.0 M in Et_2O) dropwise over 5 min under an N_2 atmosphere. The solution turned bright yellow/orange. After 1.5 h, NH_4Cl (30 mL, sat. aq.) was added in one portion and the cooling bath was removed. After a further 15 min, the organic layer was separated, and the aqueous layer extracted with CH_2Cl_2 (2 × 50 mL). The combined organic extracts were dried over Na_2SO_4, filtered, and concentrated under reduced pressure. The resulting crude residue (2.4 g) was dissolved in CH_2Cl_2 (25 mL) and *p*-TsOH·H_2O (476 mg, 2.50 mmol) was added in one portion. After stirring for 1 h, $NaHCO_3$ (25 mL, sat. aq.) was added in one portion. The mixture was passed through a phase separator and the filtrate was concentrated under reduced pressure. The resulting crude residue (2.2 g) was dissolved in a 75:25 mixture of acetone and H_2O (24 mL) and stirred. Then OsO_4 (2.5 mL, 2.5% (w/w) in *tert*-BuOH) was added in one portion followed by addition of 2-methylpyridine *N*-oxide (586 mg, 5.00 mmol) in one portion. After 16 h, a solution of sodiumhydrosulfite (500 mg) in water (5 mL) was added. After 10 min, the mixture was filtered through a plug of Celite. The residue was partially concentrated under reduced pressure and added to a mixture of EtOAc (30 mL) and $NaHCO_3$ (40 mL, sat. aq.). The organic layer was separated and the aqueous layer was extracted with EtOAc (2 × 30 mL). The combined organic extracts were washed with brine (30 mL), dried over Na_2SO_4, filtered, and concentrated under reduced pressure. The resulting crude residue was purified by column chromatography (33-100% EtOAc/*n*-heptane) to give a mixture of diol trans-**33** and cis-**33**.

Yield: Combined yield trans-**33** and cis-**33**: (656 mg, 28% over 3 steps). Isolated as a yellow foam. >95% pure by NMR.

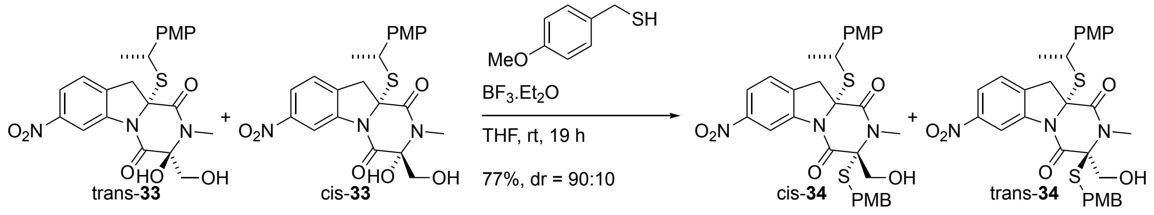

(3*R*,10a*R*)-3-(Hydroxymethyl)-3-((4-methoxybenzyl)thio)-10a-(((*R*)-1-(4-methoxyphenyl)ethyl)thio)-2-methyl-7-nitro-2,3,10,10a-tetrahydropyrazino[1,2-a]indole-1,4-dione (cis-34). To a stirred solution of diols trans-**33** and cis-**33** (375 mg, 792 µmol) and 4-methoxy-α-toluenethiol (1.10 mL, 7.92 mmol) in THF (16 mL) was added $BF_3 \cdot Et_2O$ (1.95 mL, 15.8 mmol) in one portion. After 19 h, $NaHCO_3$ (20 mL, sat. aq.) was carefully added. The organic layer was separated and the aqueous later was extracted with EtOAc (3 × 30 mL). The combined organic layers were dried over Na_2SO_4, filtered, and concentrated under reduced pressure. The resulting crude residue was purified by column chromatography (50-75% EtOAc/*n*-heptane) to give bis-sulfide cis-**34** as a single detected diastereomer.

Yield: 373 mg (77%). Isolated as a yellow foam. >95% pure by NMR and a single spot by TLC.

Selectivity: Measured by ^1H NMR spectroscopy of the crude residue. cis-**34** (δ = 8.78 ppm) / trans-**34** (δ = 8.56 ppm) = 90:10.

Major diastereomer cis-**34**:

R_f: 0.21 in 50% EtOAc/*n*-heptane. Stains dark green with PMA stain.

Optical rotation: $[\alpha]_D^{20}$: +25 (*c* = 0.5 in $CHCl_3$).

^1H NMR ($CDCl_3$, 400 MHz): δ 8.76-8.72 (m, 1H), 8.05-7.99 (m, 1H), 7.37-7.30 (m, 1H), 7.22-7.15 (m, 2H), 7.13-7.06 (m, 2H), 6.85-6.78 (m, 2H), 6.71-6.65 (m, 2H), 4.45 (d, *J* = 12.0 Hz,

1H), 4.29 (q, J = 7.2 Hz, 1H), 3.99 (d, J = 12.4 Hz, 1H), 3.91 (d, J = 12.4 Hz, 1H), 3.88 (d, J = 12.0 Hz, 1H), 3.85 (d, J = 17.6 Hz, 1H), 3.79 (s, 3H), 3.73 (s, 3H), 3.64 (d, J = 17.6 Hz, 1H), 3.02-2.98 (m, 3H), 1.50 (d, J = 7.2 Hz, 3H) ppm. The signal from the hydroxyl group proton could not be located.

^{13}C NMR ($CDCl_3$, 101 MHz): δ 165.7, 162.5, 159.2, 158.7, 148.0, 141.6, 136.4, 135.3, 130.4, 128.4, 128.1, 125.4, 121.5, 114.2, 113.7, 112.9, 73.0, 72.6, 64.7, 55.41, 55.36, 44.8, 43.3, 35.0, 29.1, 25.0 ppm.

FTIR (film): 3448 (br), 2959 (m), 2933 (m), 2836 (m), 1669 (m), 1608 (m), 1525 (m), 1510 (m), 1405 (m), 1383 (m), 1347 (m), 1302 (m), 1247 (m), 1176 (m), 1032 (m), 909 (m), 831 (m), 735 (m), 655 (m), 542 (m), 423 (m) cm^{-1}.

HRMS-ESI (*m/z*): $[M + Na]^+$ Calcd for $C_{30}H_{31}N_3NaO_7S_2$ 632.1501, Found 632.1501.

mp: 80-83 °C (obtained by concentration from EtOAc/*n*-heptane).

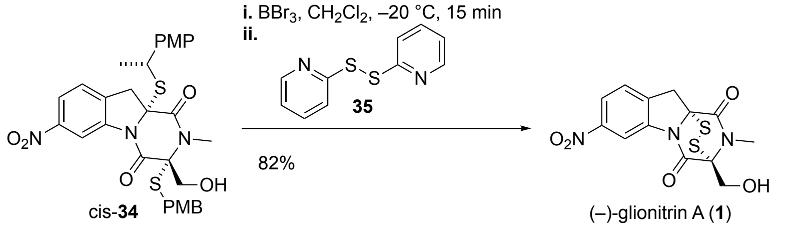

(–)-Glionitrin A (1). To a stirred solution of bis-sulfide cis-**34** (61.0 mg, 100 µmol) in CH_2Cl_2 (12 mL) at –20 °C (ice/NaCl bath) was added BBr_3 (250 µL, 1.0 M in *n*-hexane) dropwise over 1 min resulting in an orange/yellow suspension. After 15 min, H_2O (12 mL) was added, and the cooling bath was removed. The reaction mixture was warmed to room temperature and passed through a phase separator. To the filtrate was added disulfide **35** (22.0 mg, 100 µmol) in one portion resulting in a color change to bright yellow. The mixture was then concentrated under reduced pressure. The resulting crude residue was purified by column chromatography (33-50% EtOAc/*n*-heptane) to give (–)-glionitrin A (**1**).

Yield: 29 mg (82% yield). Isolated as yellow powder. >95% pure by NMR and a single spot by TLC.

R_f: 0.34 in 50% EtOAc/*n*-heptane. Stains yellow with $KMnO_4$ stain.

Optical rotation: $[\alpha]_D^{20}$: –372 (c = 0.1 in $CHCl_3$).[6]

^{1}H NMR ($CDCl_3$, 400 MHz): δ 8.75 (d, J = 2.2 Hz, 1H), 8.13 (dd, J = 8.4, 2.2 Hz, 1H), 7.52 (d, J = 8.4 Hz, 1H), 4.51 (dd, J = 12.8, 5.8 Hz, 1H), 4.39 (dd, J = 19.2, 1.2 Hz, 1H), 4.33 (dd, J = 12.8, 9.8 Hz, 1H), 3.42 (d, J = 19.2 Hz, 1H), 3.41 (dd, J = 9.8, 5.8 Hz, 1H), 3.29 (s, 3H) ppm.

^{13}C NMR ($CDCl_3$, 101 MHz): δ 165.4, 161.3, 148.8, 139.2, 135.2, 125.9, 121.6, 111.3, 76.6, 74.3, 60.8, 36.7, 27.7 ppm.

FTIR (film): 3496 (br), 2925 (w), 2854(w), 1690 (s), 1606 (w), 1526 (m), 1485 (m), 1440 (m), 1369 (m), 1347 (m), 1329 (w), 1238 (w), 1166 (w), 1065 (w), 890 (w), 825 (w), 739 (m) cm^{-1}.

HRMS-ESI (*m/z*): $[M + Na]^+$ Calcd for $C_{13}H_{11}N_3NaO_5S_2$ 376.0038, Found 376.0030.

mp: 180-182 °C (obtained by recrystallization from MeCN/H_2O).

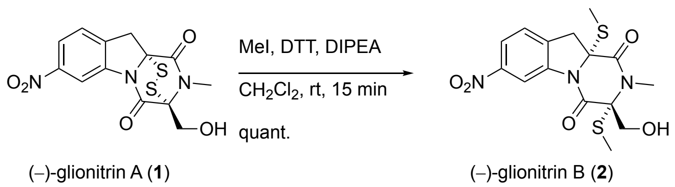

(–)-Glionitrin B (2). To a stirred solution of (–)-glionitrin A (**1**) (18.0 mg, 50.9 μmol) in CH_2Cl_2 was added MeI (63 μL, 1.01 mmol) followed by DL-dithiothreitol (15.7 mg, 102 μmol) and *N*,*N*-diisopropylethylamine (27 μL, 153 μL) in one portion respectively, resulting in a color change to faint yellow. After 10 min, the reaction mixture was directly purified by column chromatography (50-75% EtOAc/*n*-heptane) to obtain (–)-glionitrin B (**2**).

[6] The specific rotation was also measured at half the reported concentration with the same result.

Yield: 20 mg (quant.). Isolated as white solid. >95% pure by NMR and a single spot by TLC.

R*f*: 0.16 in 50% EtOAc/*n*-heptane.

Optical rotation: $[\alpha]_D^{20}$: –77 (*c* = 0.1 in MeOH).

^{1}H NMR (methanol-*d*$_4$, 400 MHz): δ 8.82 (d, *J* = 2.0 Hz, 1H), 8.13 (dd, *J* = 8.4, 2.0 Hz, 1H), 7.61 (d, *J* = 8.4, 2.0 Hz, 1H), 4.38 (d, *J* = 11.5 Hz, 1H), 3.97 (d, *J* = 11.5 Hz, 1H), 3.72 (br, s, 2H), 3.19 (s, 3H), 2.35 (s, 3H), 2.26 (s, 3H) ppm. The signal from the hydroxyl group proton could not be located.

^{13}C NMR (methanol-*d*$_4$, 101 MHz): δ 167.3, 164.1, 149.2, 143.1, 138.5, 127.1, 122.3, 113.4, 73.9, 72.7, 64.9, 40.4, 29.3, 14.4, 13.6 ppm.

FTIR (film): 3424 (br), 3129 (w), 2924 (w), 1722 (w), 1668 (s), 1604 (w). 1525 (s), 1480 (m), 1429 (m), 1386 (s), 1346 (s), 1256 (w), 1219 (m), 1182 (w), 1126 (m), 1062 (w), 1048 (w), 894 (w), 822 (w), 735 (m), 658 (w), 535 (w), 426 (w) cm^{-1}.

HRMS-ESI (*m/z*): $[M + Na]^+$ Calcd for $C_{15}H_{17}N_3NaO_5S_2$ 406.0507, Found 406.0507.

HPLC: Chiralpak® AS-H, 5 μm silica gel, IPA/*n*-hexane = 50/50, flow rate = 0.5 mL/min, λ = 325 nm. t_R = 12.13 min (major), 13.24 min (minor).

Enantiomeric ratio: >99:1

Table S5. HPLC chromatogram of (–)-(*R*)-glionitrin B (**2**).

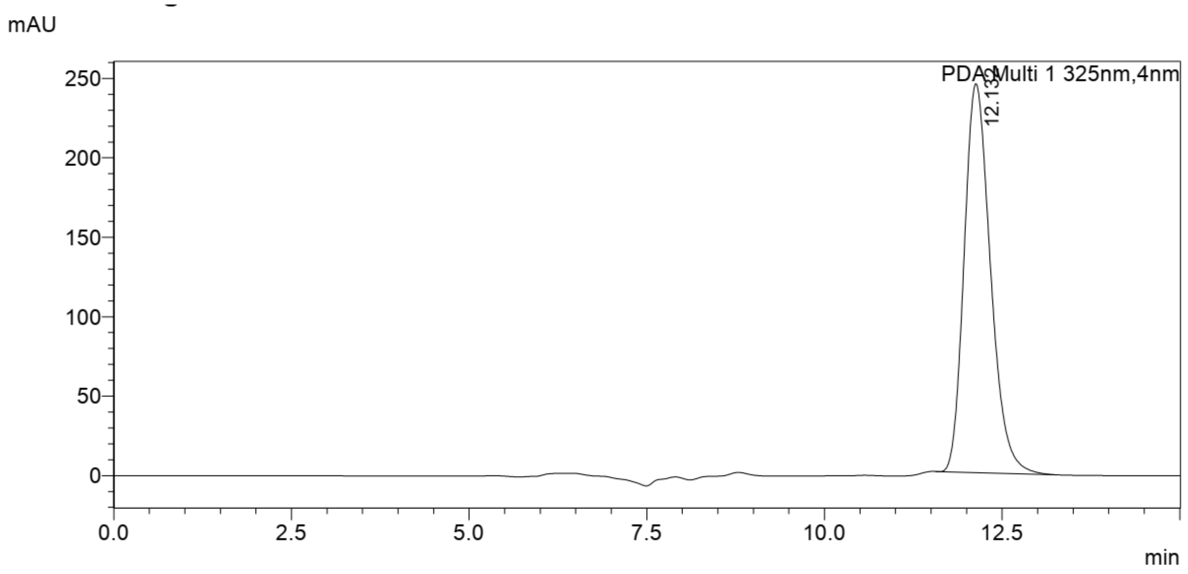

Peak	Retention time (min)	Height [mAU]	Area (%)
1	12.13	244.69	100
2	-	LoD	-

Table S6. HPLC chromatogram of (+)-(*S*)-glionitrin B (**2**).

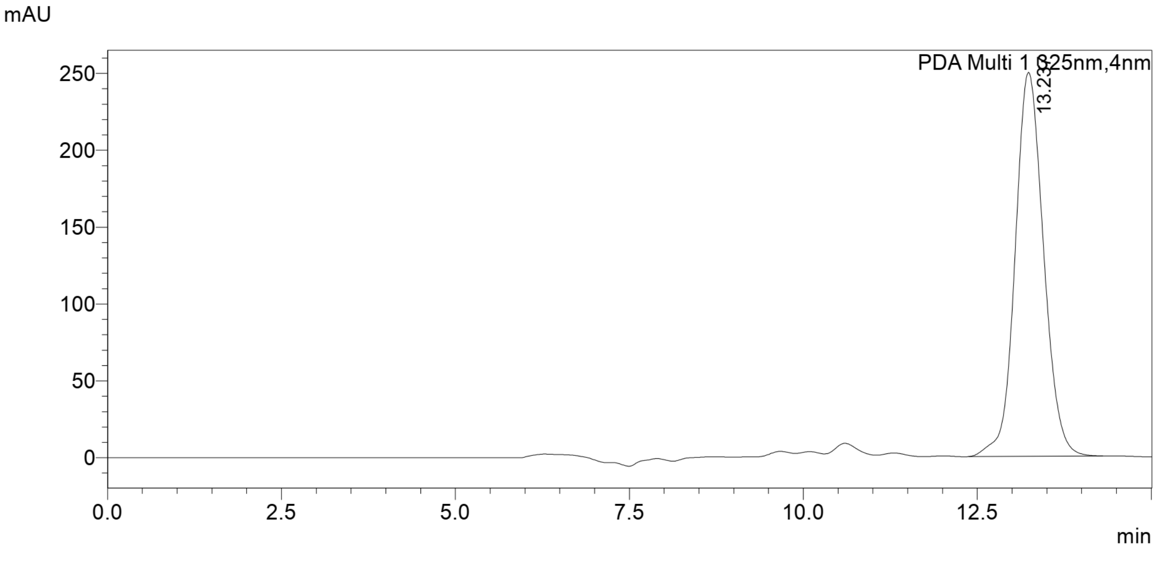

Peak	Retention time (min)	Height [mAU]	Area (%)
1	13.24	249.78	100
2	-	LoD	-

Table S7. HPLC chromatogram of *rac*-glionitrin B (**2**).

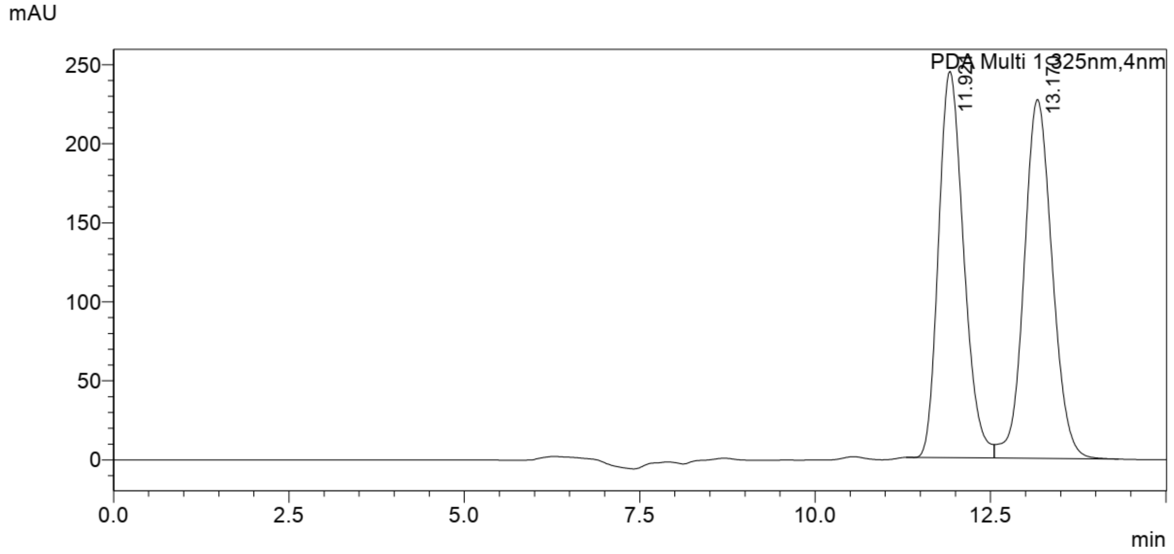

Peak	Retention time (min)	Height [mAU]	Area (%)
1	11.92	244.26	49.0
2	13.17	227049	51.0

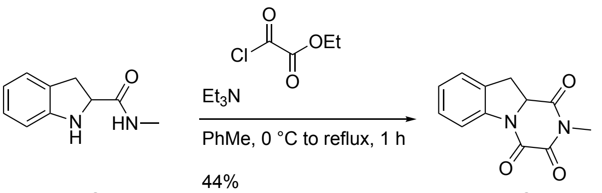

2-Methyl-10,10a-dihydropyrazino[1,2-a]indole-1,3,4(2*H*)-trione (*rac*-S4). To a stirred suspension of amide *rac*-**S3**[7] (5.29 g, 30.0 mmol) in toluene (250 mL) was added Et_3N (12.5 mL, 90.0 mmol) in one portion under an N_2 atmosphere. The resulting mixture was cooled to 0 °C using an ice-water bath and ethyl 2-chloro-2-oxoacetate (6.70 mL, 60.0 mmol) was added dropwise. The cooling bath was then removed and the reaction mixture was heated to reflux. After 1 h, the reaction mixture was cooled to room temperature and filtered. The collected solid

[7] Prepared following: Herscheid, J.D.M.; Scholten, H.P.H.; Tijhuis, M.W.; Ottenheijm, H.J.C. *Res. Trav. Chim. Pays-Bas* **1981**, 73-78.

residue was washed successively with water, EtOH, pentane, and was finally dried *in vacuo* to give triketopiperazine *rac*-**S4**.

Yield: 44%. Isolated as a brown solid. >95% pure by NMR.

R*f*: 0.47 in 50% EtOAc/*n*-heptane. Stains dark green with PMA stain.

^{1}H NMR (DMSO-*d*$_6$, 400 MHz): δ 7.95 (d, *J* = 7.6 Hz, 1H), 7.74 (d, *J* = 7.2 Hz, 1H), 7.31 (t, *J* = 7.2 Hz, 1H), 7.19 (dt, *J* = 7.6, 0.8 Hz, 1H), 5.35 (dd, *J* = 10.2, 9.2 Hz, 1H), 3.45 (dd, *J* = 16.0, 9.2 Hz, 1H), 3.37 (dd, *J* = 16.0, 10.2 Hz, 1H), 3.13 (s, 3H) ppm.

^{13}C NMR (DMSO-*d*$_6$, 101 MHz): δ 168.6, 158.7, 150.3, 140.6, 130.5, 127.6, 125.6, 125.3, 116.1, 60.6, 31.3, 26.9 ppm.

FTIR (film): 1747 (w), 1723 (s), 1603 (w), 1589 (w), 1489 (w), 1465 (w), 1416 (w), 1377 (w), 1337 (w), 1298 (w), 1246 (w), 1216 (w), 1188 (w), 1116 (w), 1069 (w), 795 (m), 626 (w), 561 (w), 509 (w) cm^{-1}.

HRMS-ESI (*m/z*): [M + Na]$^+$ Calcd $C_{12}H_{10}NaN_2O_3$ 253.0589, Found 253.0595.

mp: 244-246 °C (obtained by filtration, see above). Decomposes, gas evolution observed.

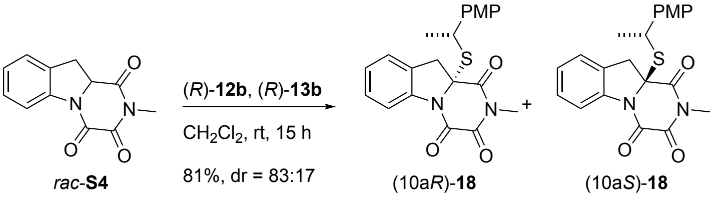

(*R*)-10a-(((*R*)-1-(4-Methoxyphenyl)ethyl)thio)-2-methyl-10,10a-dihydropyrazino[1,2-a]indole-1,3,4(2*H*)-trione ((10a*R*-18). To a stirred mixture of triketopiperazine *rac*-**S4** (23.0 mg, 100 µmol), *N*-thiosuccinimide (*R*)-**13b** (29.2 mg, 110 µmol), and catalyst (*R*)-**12b** (2.0 mg, 10.0 µmol) was added CH_2Cl_2 (2 mL). After 15 h, HCl (2.0 mL, aq., 1.0 M) was added in one portion. The resulting mixture was passed through a phase separator and concentrated under reduced pressure. The resulting crude residue was purified by column chromatography (33-

50% EtOAc/*n*-heptane) to give a mixture of thioaminal (10a*R*)-**18** and (10a*S*)-**18**. Analytically pure (10a*R*)-**18** was obtained by purification by column chromatography (10-25% EtOAc/*n*-heptane).

Yield: Combined yield (10a*R*)-**18** and (10a*S*)-**18**: (32 mg, 81%). Isolated as white solid. >95% pure by NMR.

Selectivity: Measured by ^{1}H NMR spectroscopy of the crude residue. (10a*R*)-**18** (δ = 1.44 ppm) / (10a*S*)-**18** (δ = 1.36 ppm) = 83:17.

Major diastereomer (10a*R*)-**18**:

R*f*: 0.58 in 50% EtOAc/*n*-heptane. Stains dark green with PMA stain.

Optical rotation: $[\alpha]_D^{20}$: +262 (*c* = 0.1 in $CHCl_3$).

^{1}H NMR ($CDCl_3$, 400 MHz): δ 8.00-7.95 (m, 1H), 7.37-7.29 (m, 2H), 7.27-7.20 (m, 1H), 7.09-7.03 (m, 2H), 6.83-6.77 (m, 2H), 4.01 (q, *J* = 7.2 Hz, 1H), 3.86 (d, *J* = 17.0 Hz, 1H), 3.79 (s, 3H), 3.52 (d, *J* = 17.0 Hz, 1H), 3.00 (s, 3H), 1.44 (d, *J* = 7.2 Hz, 3H) ppm.

^{13}C NMR ($CDCl_3$, 101 MHz): δ 167.2, 159.2, 157.3, 150.0, 139.2, 134.3, 128.6, 128.2, 128.0, 126.8, 125.4, 117.7, 114.2, 73.2, 55.5, 44.9, 40.7, 27.7, 25.3 ppm.

FTIR (film): 2965 (w), 29.25 (w), 1746 (w), 1690 (s), 1608 (w), 1511 (m), 1482 (m), 1463 (m), 1408 (m), 1354 (m), 1317 (m), 1248 (m), 1178 (m), 1076 (w), 1030 (w), 832 (w), 733 (w) cm^{-1}.

HRMS-ESI (*m*/*z*): $[M + H]^+$ Calcd for $C_{21}H_{20}N_2O_4S$ 397.1222, Found 397.1213.

mp: 140-141 °C (EtOH).

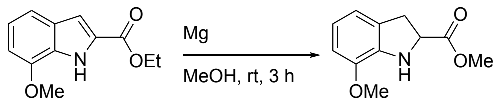

S5 *rac*-S6

Methyl 7-methoxyindoline-2-carboxylate (*rac*-S6). To a stirred suspension of indole **S5** (2.19 g, 10.0 mmol) in MeOH (25 mL) were added magnesium turnings (1.22 g, 50.0 mmol) in one portion under an N_2 atmosphere. After 2 h an exothermic reaction occurred accompanied by gas evolution. After an additional 1 h, the reaction mixture was cooled to 0 °C with an ice-water bath and HCl (aq., 1M) was added until pH <1. The unreacted magnesium was removed by filtration. The filtrate was neutralized with $NaHCO_3$ and then extracted with CH_2Cl_2 (3 × 50 mL). The combined organic extracts were dried over Na_2SO_4, filtered, and concentrated under reduced pressure. The resulting crude residue was purified by column chromatography (25% EtOAc/*n*-heptane) to obtain indoline *rac*-**S6**.

Yield: 1.49 g (72%). Isolated as a yellow oil. >95% pure by NMR and a single spot by TLC.

R*f*: 0.33 in 25% EtOAc/*n*-heptane. Stains dark green with PMA stain.

^{1}H NMR ($CDCl_3$, 400 MHz): δ 6.78-6.71 (m, 2H), 6.70-6.64 (m, 1H), 4.44 (dd, *J* = 10.0, 5.8 Hz, 1H), 3.84 (s, 3H), 3.75 (s, 3H), 3.42 (dd, *J* = 16.0, 10.0 Hz, 1H), 3.36 (dd, *J* = 16.0, 5.8 Hz, 1H) ppm. The signal from the indoline nitrogen proton could not be located.

^{13}C NMR ($CDCl_3$, 101 MHz): δ 174.5, 145.8, 138.9, 127.9, 120.5, 117.0, 109.7, 60.4, 55.5, 52.6, 34.4 ppm.

FTIR (film): 3369 (br), 3001 (w), 2952 (w), 2908 (w), 2837 (w), 1736 (s), 1619 (m), 1595 (m), 1491 (s), 1470 (m), 1439 (s), 1395 (w), 1295 (m), 1266 (s), 1198 (s), 1180 (m), 1161 (m), 1081 (m), 1013 (w), 939 (w), 738 (m), 684 (w) cm^{-1}.

HRMS-ESI (*m/z*): $[M + H]^+$ Calcd for $C_{11}H_{13}NO_3$ 208.0974, Found 208.0974.

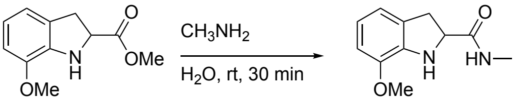

rac-**S6** *rac*-**S7**

7-Methoxy-*N*-methylindoline-2-carboxamide (*rac*-S7). Indoline *rac*-**S6** (936 mg, 4.52 mmol) was charged in a round bottom flask and methylamine (25 mL, aq., 40% (w/w)) was added. The resulting white suspension was stirred for 30 min and then filtered. The collected solid residue was washed with water, then with pentane, and finally dried *in vacuo* to give amide *rac*-**S7**.

Yield: 749 mg (80%). Isolated as a white solid. >95% pure by NMR and a single spot by TLC.

R*f*: 0.23 in 75% EtOAc/*n*-heptane. Stains dark green with PMA stain.

^{1}H NMR (CDCl$_3$, 400 MHz): δ 7.25 (br, s, 1H), 6.84-6.79 (m, 1H), 6.78-6.73 (m, 1H), 6.71-6.67 (m, 1H), 4.45 (dd, *J* = 11.0, 8.8 Hz, 1H), 3.85 (s, 3H), 3.62 (dd, *J* = 16.4, 11.0 Hz, 1H), 3.11 (dd, *J* = 16.4, 8.8 Hz, 1H), 2.84 (d, *J* = 5.2 Hz, 3H) ppm. The signal from the indoline nitrogen proton could not be located.

^{13}C NMR (CDCl$_3$, 101 MHz): δ 174.5, 146.1, 138.2, 129.3, 121.5, 117.2, 109.4, 61.8, 55.5, 36.3, 25.9 ppm.

FTIR (film): 3331 (s), 2945 (w), 2920 (w), 2844 (w), 1736 (w), 1640 (s), 1594 (m), 1533 (m), 1489 (m), 1473 (m), 1434 (w), 1405 (w), 1328 (w), 1279 (m), 1258 (m), 1230 (m), 1206 (w), 1155 (s), 1092 (w), 999 (w), 944 (w), 746 (s), 717 (m), 696 (w), 667 (w), 608 (m), 589 (w), 536 (w), 480 (w) cm^{-1}.

HRMS-ESI (*m*/*z*): $[M + H]^+$ Calcd for $C_{11}H_{14}N_2O_2$ 207.1134, Found 207.1130.

mp: 177-179 °C (obtained by filtration, see above).

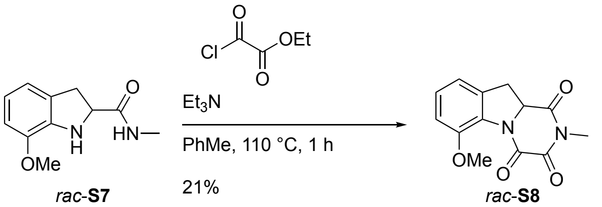

rac-**S7** *rac*-**S8**

6-Methoxy-2-methyl-10,10a-dihydropyrazino[1,2-a]indole-1,3,4(2H)-trione (*rac*-S8). To a stirred suspension of amide *rac*-**S7** (31.0 mg, 150 µmol) in toluene (1.5 mL) was added Et_3N (84 µL, 600 µmol) in one portion followed by ethyl 2-chloro-2-oxoacetate (34 µL, 300 µmol) in one portion under an N_2 atmosphere. The reaction mixture was heated to 110 °C. After 1 h, the reaction mixture was cooled to room temperature and directly purified by column chromatography (33-50% EtOAc/*n*-heptane) to obtain triketopiperazine *rac*-**S8**.

Yield: 8 mg (21%). Isolated as a white crystalline solid. >95% pure by NMR and a single spot by TLC.

R_f: 0.28 in 50% EtOAc/*n*-heptane. Stains dark green with PMA stain.

^{1}H NMR ($CDCl_3$, 400 MHz): δ 7.25 (t, *J* = 8.0 Hz, 1H), 6.95 (d, *J* = 8.0 Hz, 2H), 5.06 (dd, *J* = 11.2, 8.4 Hz, 1H), 3.96 (s, 3H), 3.49 (dd, *J* = 15.2, 8.4 Hz, 1H), 3.41 (dd, *J* = 15.2, 11.2 Hz, 1H), 3.35 (s, 3H) ppm.

^{13}C NMR ($CDCl_3$, 101 MHz): δ 167.6, 158.6, 150.9, 149.3, 133.2, 129.1, 129.0, 117.2, 113.2, 63.4, 56.6, 35.1, 27.7 ppm.

FTIR (film): 3016 (w), 2939 (w), 2840 (w), 1742 (w), 1684 (s), 1610 (w), 1488 (w), 1438 (m), 1410 (m), 1360 (m), 1325 (m), 1276 (m), 1210 (m), 1117 (w), 1070 (m), 950 (w), 933 (w), 789 (m), 734 (m), 633 (w), 580 (w), 519 (w), 410 (m) cm^{-1}.

HRMS-ESI (*m/z*): $[M + H]^+$ Calcd for $C_{13}H_{12}N_2O_4$ 261.0875, Found 261.0870.

mp: 236-238 °C (obtained by trituration from EtOH).

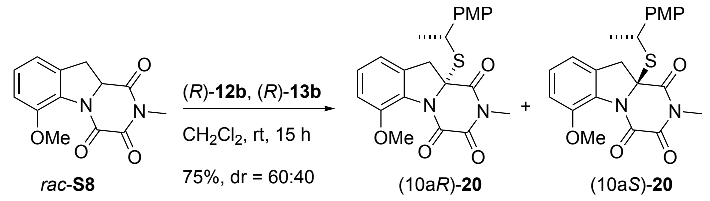

(*R*)-6-Methoxy-10a-(((*R*)-1-(4-methoxyphenyl)ethyl)thio)-2-methyl-10,10a-dihydropyrazino[1,2-a]indole-1,3,4(2H)-trione ((10a*R*)-20). To a stirred mixture of triketopiperazine *rac*-**S8** (13.0 mg, 50.0 µmol), *N*-thiosuccinimide (*R*)-**13b** (14.6 mg, 55.0 µmol), and catalyst (*R*)-**12b** (9.8 mg, 50.0 µmol) was added CH_2Cl_2 (1 mL). After 15 h, HCl (1 mL, aq., 1.0 M) was added in one portion. The resulting mixture was passed through a phase separator and concentrated under reduced pressure. The resulting crude residue was purified by column chromatography (25-50% EtOAc/*n*-heptane) to give a mixture of thioaminals (10a*R*)-**20** and (10a*S*)-**20**. Analytically pure (10a*R*)-**20** was obtained by purification by column chromatography (25-50% EtOAc/*n*-heptane).

Yield: Combined yield (10a*R*)-**20** and (10a*S*)-**20**: (19 mg, 75%). Isolated as white solid. >85% pure by NMR.

Selectivity: Measured by ^{1}H NMR spectroscopy of the crude residue. (10a*R*)-**20** (δ = 1.47 ppm) / (10a*S*)-**20** (δ = 1.31 ppm) = 60:40.

Major diastereomer (10a*R*)-**20**:

R*f*: 0.38 in 50% EtOAc/*n*-heptane. Stains dark green with PMA stain.

Optical rotation: $[\alpha]_D^{20}$: +123 (*c* = 0.1 in $CHCl_3$).

^{1}H NMR ($CDCl_3$, 400 MHz): δ 7.25 (dd, *J* = 8.4, 7.6 Hz, 1H), 7.08-7.00 (m, 2H), 6.97-6.89 (m, 2H), 6.80-6.74 (m, 2H), 4.00 (q, *J* = 7.2 Hz, 1H), 3.91 (s, 3H), 3.78 (s, 3H), 3.76 (d, *J* = 16.8 Hz, 1H), 3.36 (d, *J* = 16.8 Hz, 1H), 3.24 (s, 3H), 1.47 (d, *J* = 7.2 Hz, 3H) ppm.

^{13}C NMR ($CDCl_3$, 101 MHz): δ 168.0, 159.0, 157.9, 151.2, 148.7, 134.0, 132.4, 129.2, 128.1, 127.3, 117.2, 114.3, 113.1, 76.1, 56.4, 55.4, 44.6, 42.7, 27.9, 24.7 ppm.

FTIR (film): 2962 (w), 2930 (w), 2837 (w), 1745 (w), 1687 (s), 1611 (w), 1511 (m), 1490 (m), 1458 (w), 1396 (m), 1351 (m), 1319 (m), 1287 (m), 1201 (m), 1179 (m), 1078 (m), 1030 (w), 942 (w), 910 (w), 833 (w), 768 (w), 732 (m) cm^{-1}.

HRMS-ESI (*m/z*): $[M + H]^+$ Calcd for $C_{22}H_{22}N_2O_5S$ 427.1328, Found 427.1321.

mp: 79-81 °C (obtained by concentration from EtOAc/*n*-heptane).

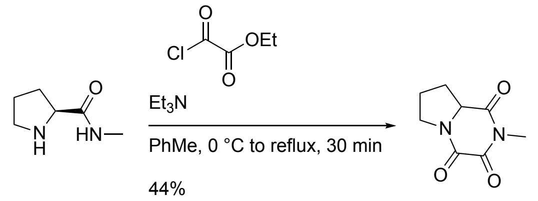

S9 *rac*-**S10**

2-Methyltetrahydropyrrolo[1,2-a]pyrazine-1,3,4(2*H*)-trione (*rac*-S10). To a stirred mixture of amide **S9**[8] (1.90 g, 14.8 mmol) in toluene (50 mL) was added Et_3N (8.26 mL, 59.3 mmol) in one portion under an N_2 atmosphere. The resulting mixture was cooled to 0 °C using an ice-water bath and ethyl 2-oxoacetate (3.31 mL, 29.6 mmol) was added dropwise. The cooling bath was removed and the reaction mixture was heated to reflux. After 30 min, the reaction mixture was cooled to room temperature and filtered. The filtrate was concentrated under reduced pressure and the resulting crude residue was purified by column chromatography (50-100% EtOAc/*n*-heptane) to give triketopiperazine *rac*-**S10**.

Yield: 44%. Isolated as a yellow oil that crystallized upon standing. >95% pure by NMR and a single spot by TLC.

R*f*: 0.12 in 75% EtOAc/*n*-heptane. Stains dark green with PMA stain.

[8] Prepared following: Betschart, C.; Schmidt, B.; Seebach, D. *Helv. Chim. A.* **1988**, 1999-2021.

^{1}H NMR (CDCl$_3$, 400 MHz): δ 4.48-4.40 (m, 1H), 3.87-3.77 (m, 1H), 3.71-3.61 (m, 1H), 3.25 (s, 3H), 2.63-2.52 (m, 1H), 2.23-1.92 (m, 3H) ppm.

^{13}C NMR (CDCl$_3$, 101 MHz): δ 168.3, 158.8, 151.9, 60.5, 45.9, 29.5, 27.4, 21.6 ppm.

IR (film): 2960 (w), 2889 (w), 1746 (w), 1684 (s), 1457 (w), 1419 (m), 1369 (m), 1336 (m), 1304 (m), 1216 (w), 1149 (w), 1097 (w), 1052 (w) cm^{-1}.

HRMS-ESI (*m/z*): [M + Na]$^{+}$ Calcd $C_8H_{10}NaN_2O_3$ 205.0589, Found 205.0595.

mp: 96-98 °C (obtained by concentration from EtOAc/*n*-heptane).

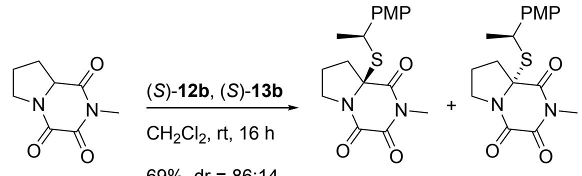

rac-**S10** (2a*S*)-**25** (2a*R*)-**25**

(*S*)-8a-(((*S*)-1-(4-Methoxyphenyl)ethyl)thio)-2-methyltetrahydropyrrolo[1,2-a]pyrazine-1,3,4(2*H*)-trione ((2a*S*-25). To a stirred mixture of triketopiperazine *rac*-**S10** (18.2 mg, 100 μmol), *N*-thiosuccinimide (*S*)-**13b** (29.2 mg, 110 μmol), and catalyst (*S*)-**12b** (19.6 mg, 110 μmol) was added CH_2Cl_2 (2 mL). After 16 h, the reaction mixture was cooled to room temperature and HCl (2 mL, aq., 1.0 M) was added in one portion. The resulting mixture was passed through a phase separator and concentrated under reduced pressure. The resulting crude residue was purified by column chromatography (33-50% EtOAc/*n*-heptane) to give a mixture of thioaminals (2a*S*)-**25** and (2a*R*)-**25**. Analytically pure (2a*S*)-**25** was obtained by recrystallizing the obtained mixture of (2a*S*)-**25** and (2a*R*)-**25** from EtOH.

Yield: Combined yield (2a*S*)-**25** and (2a*R*)-**25**: (24 mg, 69%). Isolated as white crystalline solid. Analytically pure (2a*S*)-**25** was isolated as white needles. >95% pure by NMR.

Selectivity: Measured by ^{1}H NMR spectroscopy of the crude residue. (2a*S*)-**25** (δ = 1.50 ppm) / (2a*R*)-**25** (δ = 1.42 ppm) = 86:14.

Major diastereomer (2a*S*)-**25**:

R*f*: 0.20 in 50% EtOAc/*n*-heptane. Stains dark green with PMA stain.

Optical rotation: $[\alpha]_D^{20}$: –225 (*c* = 0.1 in $CHCl_3$).

^{1}H NMR ($CDCl_3$, 400 MHz): δ 7.15-7.09 (m, 2H), 6.87-6.80 (m, 2H), 3.87 (q, *J* = 7.2 Hz, 1H), 3.79 (s, 3H), 3.75-3.65 (m, 1H), 3.53-3.43 (m, 1H), 3.06 (s, 3H), 2.60-2.48 (m, 1H), 2.44-2.24 (m, 2H), 2.15-2.01 (m, 1H), 1.50 (d, *J* = 7.2 Hz, 3H) ppm.

^{13}C NMR ($CDCl_3$, 101 MHz): δ 167.9, 159.1, 157.6, 151.7, 134.6, 128.0, 114.3, 72.8, 55.5, 45.8, 45.4, 36.5, 27.5, 25.2, 19.8 ppm.

FTIR (film): 2962 (w), 2927 (w), 1745 (w), 1610 (s), 1511 (m), 1443 (m), 1413 (m), 1358 (m), 1336 (m), 1306 (m), 1248 (w), 1178 (w), 1078 (w), 1030 (w), 833 (w), 531 (w) cm^{-1}.

HRMS-ESI (*m/z*): $[M + Na]^+$ Calcd $C_{17}H_{20}N_2NaO_4S$ 371.1041, Found 371.1044.

mp: 166-168 °C (obtained by recrystallization from EtOH).

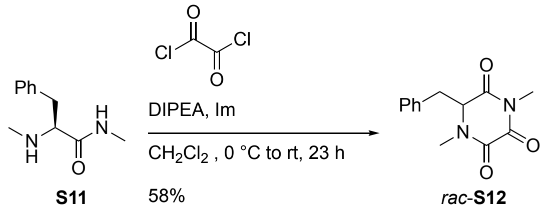

6-Benzyl-1,4-dimethylpiperazine-2,3,5-trione (*rac*-S12). To a stirred solution of imidazole (1.02 g, 15.0 mmol) in CH_2Cl_2 (15 mL) was added *N*,*N*-diisopropylethylamine (2.61 mL, 15.0 mmol) in one portion, followed by dropwise addition of a solution of oxalyl chloride (635 µL, 7.50 mmol) in CH_2Cl_2 (7.5 mL) over 10 min under an N_2 atmosphere. The reaction mixture was cooled to 0 °C with an ice-water bath and a solution of amine **S11**[9] (961 mg, 5.00 mmol) in CH_2Cl_2 (5 mL) was added dropwise over 5 min. After 23 h, HCl (50 mL, aq., 1.0 M) was added

[9] Prepared following: Hansen, T.K.; Ankersen, M.; Hansen, B.S.; Raun, K.; Nielsen, K.K.; Lau, J.; Peschke, B.; Lundt, B.F.; Thøgersen, H.; Johansen, N.L.; Madsen, K.; Andersen, P.H. *J. Med. Chem.* **1998**, 3705-3714.

in one portion. The organic layer was separated and the aqueous layer was extracted with CH_2Cl_2 (3 × 50 mL). The combined organic extracts were dried over Na_2SO_4, filtered, and concentrated under reduced pressure. The resulting crude residue was recrystallized from EtOH to give triketopiperazine *rac*-**S12**.

Yield: 708 mg (58%). Isolated as white crystals. >95% pure by NMR.

R*f*: 0.23 in 75% EtOAc/*n*-heptane. Stains dark green with PMA stain.

^{1}H NMR ($CDCl_3$, 400 MHz): δ 7.30-7.24 (m, 3H), 6.94-6.88 (m, 2H), 4.55 (app. t, *J* = 4.0, 3.6 Hz, 1H), 3.30 (dd, *J* = 14.0, 3.6 Hz, 1H), 3.25 (dd, *J* = 14.0, 4.0 Hz, 1H), 3.22 (s, 3H), 2.95 (s, 3H) ppm.

^{13}C NMR ($CDCl_3$, 101 MHz): δ 168.3, 155.8, 153.3, 132.6, 129.4, 129.3, 128.7, 64.4, 38.7, 33.4, 26.8 ppm.

FTIR (film): 2941 (w), 1744 (w), 1680 (s), 1601 (w), 1494 (w), 1454 (w), 1421 (m), 1368 (m), 1336 (m), 1321 (m), 1251 (m), 1143 (w), 1042 (w), 1003 (w), 749 (w), 704 (m), 586 (w), 533 (w), 408 (m) cm^{-1}.

HRMS-ESI (*m/z*): $[M + Na]^+$ Calcd $C_{13}H_{14}NaN_2O_3$ 269.0902, Found 269.0907.

mp: 173-175 °C (obtained by recrystallization from EtOH).

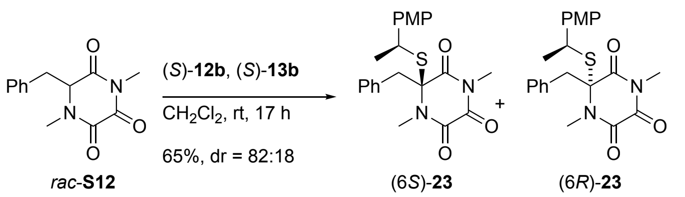

rac-**S12** (6*S*)-**23** (6*R*)-**23**

(*S*)-6-Benzyl-6-(((*S*)-1-(4-methoxyphenyl)ethyl)thio)-1,4-dimethylpiperazine-2,3,5-trione ((6*S*)-23). To a stirred mixture of triketopiperazine *rac*-**S12** (24.6 mg, 100 µmol), *N*-thiosuccinimide (*S*)-**13b** (29.2 mg, 110 µmol), and catalyst (*S*)-**12b** (19.6 mg, 100 µmol) was added CH_2Cl_2 (2 mL). After 17 h, HCl (2 mL, aq., 1.0 M) was added in one portion. The

resulting mixture was passed through a phase separator and concentrated under reduced pressure. The resulting crude residue was purified by column chromatography (33% EtOAc/*n*-heptane) to give a mixture of thioaminals (6*S*)-**23** and (6*R*)-**23**. Analytically pure (6*S*)-**23** was obtained by purification by column chromatography (50% Et_2O/*n*-heptane).

Yield: Combined yield (6*S*)-**23** and (6*R*)-**23**: (27 mg, 65%). Isolated as a clear colorless oil. >95% pure by NMR.

Selectivity: Measured by ^{1}H NMR spectroscopy of the crude residue. (6*S*)-**23** (δ = 7.02-6.96 ppm) / (6*R*)-**23** (δ = 7.09-7.04 ppm) = 82:18.

Major diastereomer (6*S*)-**23**:

R*f*: 0.38 in 80% Et_2O/*n*-heptane. Stains dark green with PMA stain.

Optical rotation: $[\alpha]_D^{20}$: –241 (*c* = 0.1 in $CHCl_3$).

^{1}H NMR ($CDCl_3$, 400 MHz): δ 7.23-7.17 (m, 3H), 7.03-6.97 (m, 2H), 6.92-6.85 (m, 2H), 6.79-6.73 (m, 2H), 3.96 (q, *J* = 7.2 Hz, 1H), 3.75 (s, 3H), 3.53 (d, *J* = 13.8 Hz, 1H), 3.18 (d, *J* = 13.8 Hz, 1H), 3.10 (s, 3H), 3.04 (s, 3H), 1.52 (d, *J* = 7.2 Hz, 3H) ppm.

^{13}C NMR ($CDCl_3$, 101 MHz): δ 167.9, 159.5, 154.7, 152.0, 133.2, 132.4, 129.3, 129.1, 128.6, 127.6, 114.6, 78.0, 55.6, 45.3, 45.0, 31.1, 27.5, 23.6 ppm.

FTIR (film): 2958 (w), 2927 (w), 1746 (w), 1685 (s), 1610 (w), 1512 (m), 1454 (w), 1417 (w), 1393 (w), 1349 (m), 1331 (m), 1250 (m), 1177 (w), 1031 (w), 834 (w), 757 (w), 704 (w) cm^{-1}.

HRMS-ESI (*m/z*): $[M + H]^+$ Calcd $C_{22}H_{24}N_2O_4S$ 413.1535, Found 413.1530.

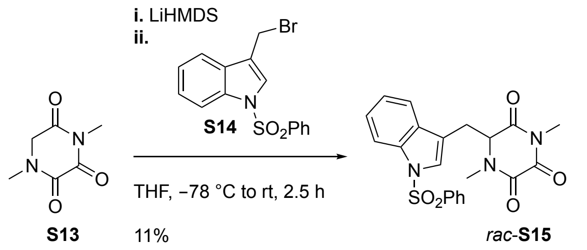

1,4-Dimethyl-6-((1-(phenylsulfonyl)-1*H*-indol-3-yl)methyl)piperazine-2,3,5-trione (*rac*-S15). To a stirred solution of triketopiperazine **S13**[10] (156 mg, 1.00 mmol) in THF (10 mL) at –78 °C was added LiHMDS (1.10 mL, 1.0 M in THF) dropwise over 2 min under an N_2 atmosphere. After 20 min, a solution of bromide **S14**[11] (385 mg, 1.10 mmol) in THF (5 mL) was added dropwise over 5 min. The cooling bath was then removed and after 2 h, NH_4Cl (10 mL, sat. aq.) was added in one portion. The organic layer was separated and the aqueous layer was extracted with EtOAc (2 × 5 mL). The combined organic extracts were dried over Na_2SO_4, filtered, and concentrated under reduced pressure. The resulting crude residue was purified by column chromatography (25-100% EtOAc/*n*-heptane) to give a white solid which was recrystallized from EtOH to give triketopiperazine *rac*-**S15**.

Yield: 46 mg, 11%. Isolated as white crystals. >95% pure by NMR.

R*f*: 0.19 in 75% EtOAc/*n*-heptane. Stains dark green with PMA stain.

^{1}H NMR ($CDCl_3$, 400 MHz): δ 7.88-7.81 (m, 3H), 7.64-7.52 (m, 3H), 7.34-7.18 (m, 4H), 4.56 (dd, *J* = 4.8, 3.4 Hz, 1H), 4.53 (ddd, *J* = 14.8, 3.4, 0.8 Hz, 1H), 3.35 (ddd, *J* = 14.8, 4.8, 0.8 Hz, 1H), 3.23 (s, 3H), 2.63 (s, 3H) ppm.

^{13}C NMR ($CDCl_3$, 101 MHz): δ 168.5, 155.6, 153.2, 137.7, 134.8, 134.3, 129.8, 129.5, 127.2, 126.1, 125.6, 123.6, 119.2, 113.8, 112.9, 63.3, 33.3, 28.8, 27.0 ppm.

---

[10] Prepared following: Snaddon, T.N.; Scaggs, T.D.; Pearson, C.M.; Fyfe, J.W.B. *Org. Lett.* **2019**, 4873-4877.

[11] Prepared following: Liu, R.; Zhang, P.; Gan, T.; Cook, J.M. *J. Org. Chem.* **1997**, 7447-7456.

FTIR (film): 3070 (w), 2950 (w), 1743 (w), 1684 (s), 1448, 1422 (w), 1367 (m), 1324 (m), 1174 (m), 1132 (m), 1123 (m), 1097 (w), 977 (w), 911 (w), 739, 685 (w), 598 (m), 571 (m), 551 (w) cm^{-1}.

HRMS-ESI (*m/z*): $[M + Na]^+$ Calcd $C_{21}H_{19}N_3NaO_5S$ 448.0943, Found 448.0937.

mp: 209-211 °C (obtained by recrystallization from EtOH).

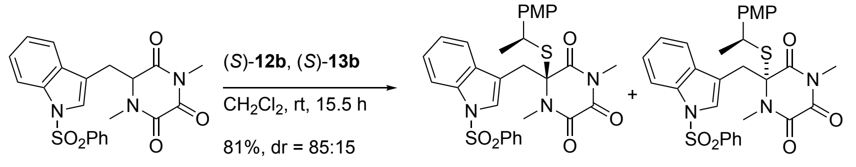

rac-**S15** (6*S*)-**22** (6*R*)-**22**

(*S*)-6-(((*S*)-1-(4-Methoxyphenyl)ethyl)thio)-1,4-dimethyl-6-((1-(phenylsulfonyl)-1H-indol-3-yl)methyl)piperazine-2,3,5-trione ((6*S*)-22). To a stirred mixture of triketopiperazine *rac*-**S15** (42.5 mg, 100 µmol), *N*-thiosuccinimide (*S*)-**13b** (29.2 mg, 110 µmol), and catalyst (*S*)-**12b** (19.6 mg, 100 µmol) was added CH_2Cl_2 (1 mL). After 15.5 h, HCl (1 mL, aq., 1.0 M) was added in one portion. The resulting mixture was passed through a phase separator and concentrated under reduced pressure. The resulting crude residue was purified by column chromatography (33-50% EtOAc/*n*-heptane) to give a mixture of thioaminals (6*S*)-**22** and (6*R*)-**22**. Analytically pure (6*S*)-**22** was obtained by purification by column chromatography (0-30% EtOAc/*n*-heptane).

Yield: Combined yield (6*S*)-**22** and (6*R*)-**22**: (48 mg, 81%). Isolated as yellow/white solid. >95% pure by NMR.

Selectivity: Measured by ^{1}H NMR spectroscopy of the crude residue. (6*S*)-**22** (δ = 7.03-6.96 ppm) / (6*R*)-**22** (δ = 7.09-7.04 ppm) = 85:15.

Major diastereomer (6*S*)-**22**:

R*f*: 0.52 in 50% EtOAc/*n*-heptane. Stains dark green with PMA stain.

Optical rotation: $[\alpha]_D^{20}$: –171 (c = 0.1 in $CHCl_3$).

^{1}H NMR ($CDCl_3$, 400 MHz): δ 7.87-7.82 (m, 1H), 7.69-7.63 (m, 2H), 7.55-7.49 (m, 1H), 7.48-7.38 (m, 3H), 7.30-7.24 (m, 1H), 7.23-7.28 (m, 1H), 7.17 (s, 1H), 7.03-6.97 (m, 2H), 6.79-6.74 (m, 2H), 3.94 (q, J = 7.2 Hz, 1H), 3.79 (dd, J = 14.8, 1.0 Hz, 1H), 3.76 (s, 3H), 3.26 (dd, J = 14.8, 1.0 Hz, 1H), 3.03 (s, 3H), 2.87 (s, 3H), 1.52 (d, J = 7.2 Hz, 3H) ppm.

^{13}C NMR ($CDCl_3$, 101 MHz): δ 168.0, 159.6, 154.5, 151.9, 137.5, 134.6, 134.2, 133.1, 129.8, 129.6, 127.5, 126.6, 125.5, 125.1, 123.6, 119.5, 114.7, 113.7, 113.5, 76.7, 55.6, 45.5, 35.0, 30.7, 27.6, 23.8 ppm.

FTIR (film): 2963 (w), 2928 (w), 2250 (w), 1745 (w), 1681 (s), 1610 (w), 1583 (m), 1511 (m), 1447 (m), 1363 (m), 1347 (m), 1331 (m), 1305 (m), 1174 (s), 1123 (m), 1096 (m), 1030 (m), 973 (m), 910 (m), 834 (m), 748 (m), 724 (s), 686 (m), 606 (m), 571 (m), 549 (m), 423 (w) cm^{-1}.

HRMS-ESI (*m/z*): $[M + H]^+$ Calcd $C_{30}H_{29}N_3O_6S_2$ 592.1576, Found 592.1572.

mp: 84-87 °C (obtained by concentration from EtOAc/*n*-heptane).

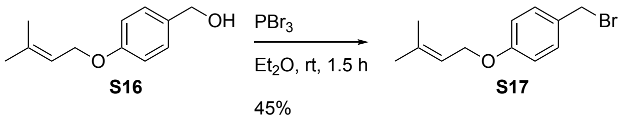

1-(Bromomethyl)-4-((3-methylbut-2-en-1-yl)oxy)benzene (S17). To a stirred solution of alcohol **S16**[12] (1.35 g, 7.00 mmol) in Et_2O (25 mL) was added PBr_3 (987 µL, 10.5 mmol) dropwise over 5 min. After 1.5 h, the reaction mixture was cooled to 0 °C with an ice-water bath and $NaHCO_3$ (12 mL, sat. aq.) was carefully added. After 15 min, the mixture was poured into water (20 mL) and $NaHCO_3$ (30 mL, sat. aq.) was added. The resulting mixture was extracted with Et_2O (3 × 50 mL). The combined organic extracts were dried over Na_2SO_4, filtered, and concentrated under reduced pressure. The resulting crude residue was purified by

[12] Prepared following: Zhao, Z.; Bai, Y.; Xie, J.; Chen, X.; He, X.; Sun, Y.; Bai, Y.; Zhang, Y.; Wu, S.; Zheng, X. *Bioorg. Chem.* **2019**, 102832.

column chromatography (10% EtOAc/*n*-heptane) to give the unstable bromide **S17**, which was used directly in the next reaction.

Yield: 799 mg (45%). Isolated as clear, colorless liquid. >95% pure by NMR.

R*f*: 0.54 in 10% EtOAc/*n*-heptane.

^{1}H NMR ($CDCl_3$, 400 MHz): δ 7.34-7.28 (m, 2H), 6.90-6.84 (m, 2H), 5.48 (tquin, *J* = 6.8, 1.6 Hz, 1H), 4.54-4.48 (m, 4H), 1.80 (d, *J* = 1.6 Hz, 3H), 1.74 (s, 3H) ppm.

^{13}C NMR ($CDCl_3$, 101 MHz): δ 159.1, 138.6, 130.6, 130.0, 119.6, 115.1, 65.0, 34.2, 26.0, 18.4 ppm.

FTIR (film): 2972 (w), 2915 (w), 2860 (w), 1608, 1582 (w), 1510 (s), 1442 (w), 1383 (w), 1300 (w), 1245 (s), 1225 (s), 1202 (m), 1174 (m), 777 (m), 831 (m), 596 (m) cm^{-1}.

HRMS-ESI (*m/z*): $[C_{12}H_{15}O]^+$ Calcd $C_{12}H_{15}OBr$ 253.0228, Found 175.1125. Ionization by loss of Br^-.

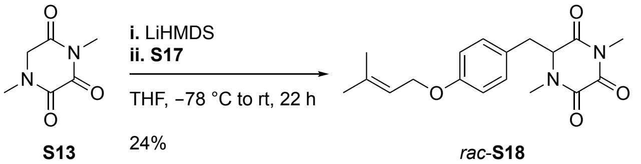

S13 rac-S18

1,4-Dimethyl-6-(4-((3-methylbut-2-en-1-yl)oxy)benzyl)piperazine-2,3,5-trione (*rac*-S18). To a stirred solution of triketopiperazine **S13**[10] (156 mg, 1.00 mmol) in THF (10 mL) at –78 °C was added LiHMDS (1.10 mL, 1.0 M in THF) dropwise over 1 min. After 30 min, a solution of bromide **S17** (281 mg, 1.10 mmol) in THF (500 µL) was added dropwise over 1 min. The cooling bath was removed and after 22 h, NH_4Cl (10 mL, sat. aq.) was added in one portion. The organic layer was separated and the aqueous layer was extracted with EtOAc (2 × 10 mL). The combined organic extracts were dried over Na_2SO_4, filtered, and concentrated under

reduced pressure. The resulting crude residue was purified by column chromatography (50-100% EtOAc/*n*-heptane) to give triketopiperazine *rac*-**S18**.

Yield: 78 mg, 24%. Isolated as a white crystalline solid. >95% pure by NMR and a single spot by TLC.

R*f*: 0.24 in 75% EtOAc/*n*-heptane. Stains dark green with PMA stain.

^{1}H NMR ($CDCl_3$, 400 MHz): δ 6.84-6.76 (m, 4H), 5.44 (tquin, *J* = 6.4, 1.2 Hz, 1H), 4.51 (app. t, *J* = 4.0, 3.6 Hz, 1H), 4.48-4.40 (m, 2H), 3.24 (dd, *J* = 14.0, 3.6 Hz, 1H), 3.21 (s, 3H), 3.18 (dd, *J* = 14.0, 4.0 Hz, 1H), 2.98 (s, 3H), 1.79 (s, 3H), 1.75 (s, 3H) ppm.

^{13}C NMR ($CDCl_3$, 101 MHz): δ 168.5, 159.2, 155.9, 153.4, 138.5, 130.5, 124.1, 119.5, 115.4, 65.0, 64.6, 38.0, 33.3, 26.8, 25.9, 18.4 ppm.

FTIR (film): 2970 (w), 2935 (w), 2864 (w), 1743 (w), 1683 (s), 1610 (w), 1510, 1421 (w), 1369 (w), 1336 (m), 1321 (m), 1242 (m), 1179 (w), 1145 (w), 1043 (m), 1001 (m), 862 (w), 817 (w), 584 (w) cm^{-1}.

HRMS-ESI (*m/z*): $[M + H]^+$ Calcd $C_{18}H_{22}N_2O_4$ 331.1658, Found 331.1649.

mp: 105-107 °C (obtained by concentration from EtOAc/*n*-heptane).

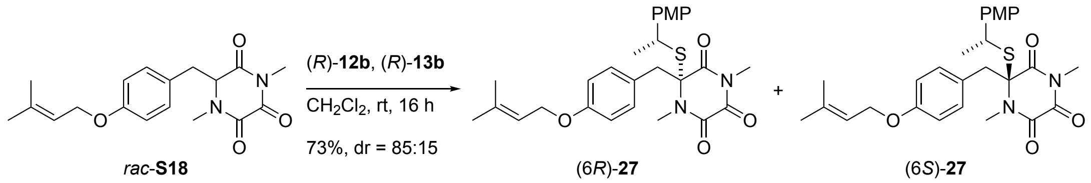

rac-**S18** (6*R*)-**27** (6*S*)-**27**

(*R*)-6-(((*R*)-1-(4-Methoxyphenyl)ethyl)thio)-1,4-dimethyl-6-(4-((3-methylbut-2-en-1-yl)oxy)benzyl)piperazine-2,3,5-trione ((6*R*)-27). To a stirred mixture of triketopiperazine *rac*-**S18** (16.5 mg, 50.0 μmol), *N*-thiosuccinimide (*R*)-**13b** (14.6 mg, 55.0 μmol), and catalyst (*R*)-**12b** (9.8 mg, 50 μmol) was added CH_2Cl_2 (1 mL). After 16 h, HCl (1 mL, aq., 1.0 M) was added in one portion. The resulting mixture was passed through a phase separator and

concentrated under reduced pressure. The resulting crude residue was purified by column chromatography (50% EtOAc/*n*-heptane) to give a mixture of thioaminals (6*R*)-**27** and (6*S*)-**27**. Analytically pure (6*R*)-**27** was obtained by purification by column chromatography (0-35% EtOAc/*n*-heptane).

Yield: Combined yield (6*R*)-**27** and (6*S*)-**27**: (18 mg, 73%). Isolated as a colorless oil. >95% pure by NMR.

Selectivity: Measured by ^{1}H NMR spectroscopy of the crude residue. (6*R*)-**27** (δ = 7.03-6.96 ppm) / (6*S*)-**27** (δ = 7.09-7.03 ppm) = 85:15.

Major diastereomer (6*R*)-**27**:

R*f*: 0.53 in 50% EtOAc/*n*-heptane. Stains dark green with PMA stain.

Optical rotation: $[\alpha]_D^{20}$: +181 (*c* = 0.1 in $CHCl_3$).

^{1}H NMR ($CDCl_3$, 400 MHz): δ 7.03-6.97 (m, 2H), 6.84-6.69 (m, 6H), 5.41 (tquin, *J* = 6.8, 1.2 Hz, 1H), 4.40 (d, *J* = 6.8 Hz, 2H), 3.95 (q, *J* = 7.2 Hz, 1H), 3.75 (s, 3H), 3.46 (d, *J* = 14.0 Hz, 1H), 3.12 (s, 3H), 3.10 (d, *J* = 14.0 Hz, 1H), 3.04 (s, 3H), 1.17 (d, *J* = 0.8 Hz, 3H), 1.71 (s, 3H), 1.52 (d, *J* = 7.2 Hz, 3H) ppm.

^{13}C NMR ($CDCl_3$, 101 MHz): δ 168.1, 159.5, 158.9, 154.8, 152.1, 138.6, 133.3, 130.5, 127.6, 124.0, 119.4, 115.2, 114.6, 78.1, 64.9, 55.6, 45.2, 44.2, 31.0, 27.5, 25.9, 23.6, 18.3 ppm.

FTIR (film): 2965 (w), 2927 (w), 2858 (w), 1745 (w), 1682 (s), 1610, 1510 (s), 1443, 1416, 1392, 1347, 1331 (s), 1247 (m), 1178 (m), 1142 (m), 1031 (m), 1003 (m), 833 (m), 730 (w), 533 (w) cm^{-1}.

HRMS-ESI (*m/z*): $[M + H]^+$ Calcd $C_{27}H_{32}N_2O_5S$ 497.2110, Found 497.2110.

III. *Assignment of the relative configuration of cis-**31**, trans-**33**, and cis-**34***[13]

*Alcohol cis-**31**.* The relative configuration of alcohol cis-**31** was established by diagnostic NOE correlations from the –OH hydroxyl proton (δ = 4.83 ppm) to the aryl group protons H^b (δ = 7.10-7.05 ppm), the benzylic proton H^a (δ = 4.21 ppm), and the methyl group protons CH_3 (δ = 1.60 ppm) of the thioether (–S–CH(CH_3)-CCH-) moiety (Figure S1).

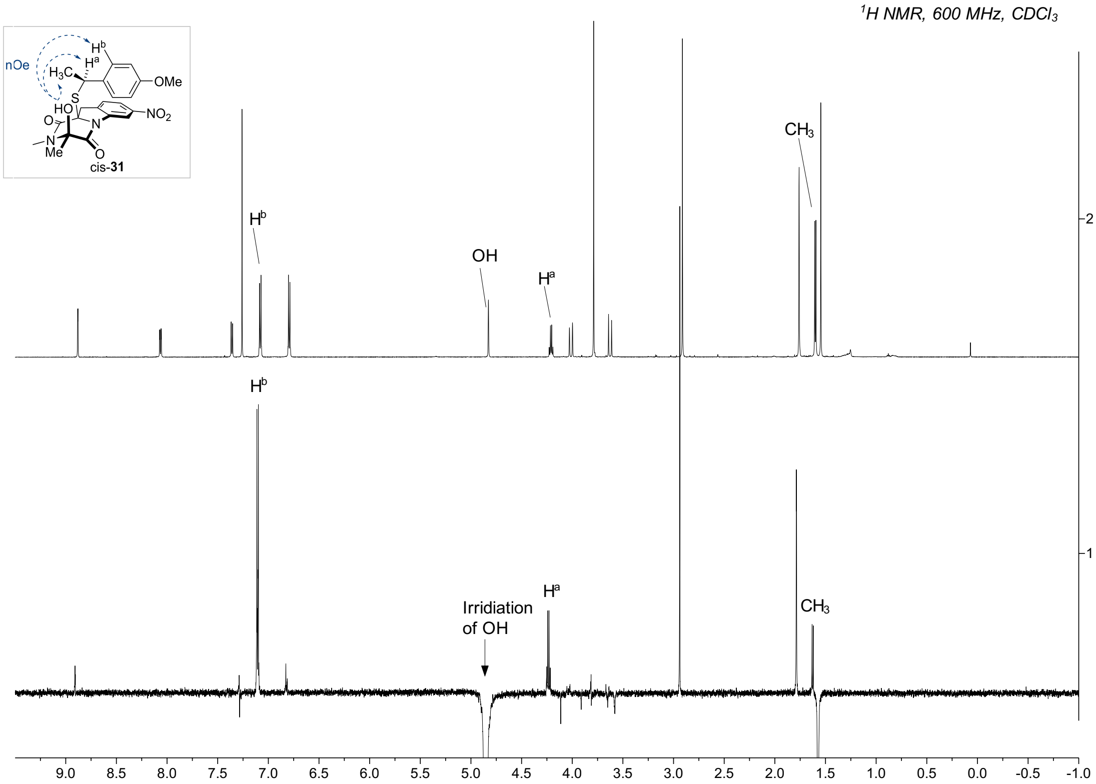

Figure S1. Diagnostic NOE correlations for cis-**31**. Irradiation of the –OH hydroxyl proton (δ = 4.83 ppm).

[13] Cis and trans refers to the relationship between the exocyclic heteroatoms on the diketopiperazine ring.

*Diol trans-**33***. The relative configuration of diol trans-**33** was established by a diagnostic NOE correlation from the –CH_2OH methylene protons (δ = 4.34 and 3.73 ppm) to the aryl group protons H[a] (δ = 7.16-7.09 ppm) of the thioether (–S–CH(CH_3)-PMP) moiety (Figure S2).

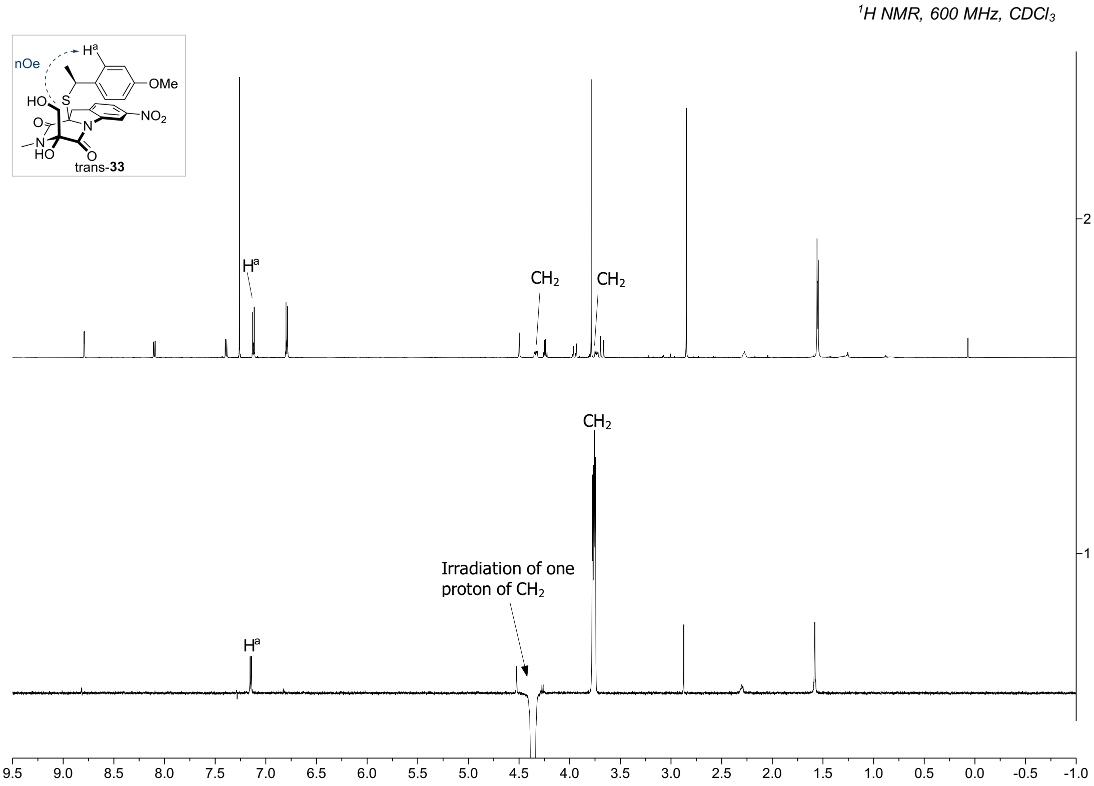

Figure S2. Diagnostic NOE correlation for trans-**33**. Irradiation of one –CH_2OH methylene proton (δ = 4.34 ppm).

*Bis-sulfide cis-**34***. The relative configuration of bis-sulfide cis-**34** was established by diagnostic NOE correlations from the CH_3 protons on the methyl group of the S–CH(CH_3)-PMP moiety (δ = 1.50 ppm) to the CH_2 protons (δ = 4.01 and 3.95 ppm) and aryl group protons H[a] (δ = 7.23-7.17 ppm) of the –S–PMB group (Figure S3).

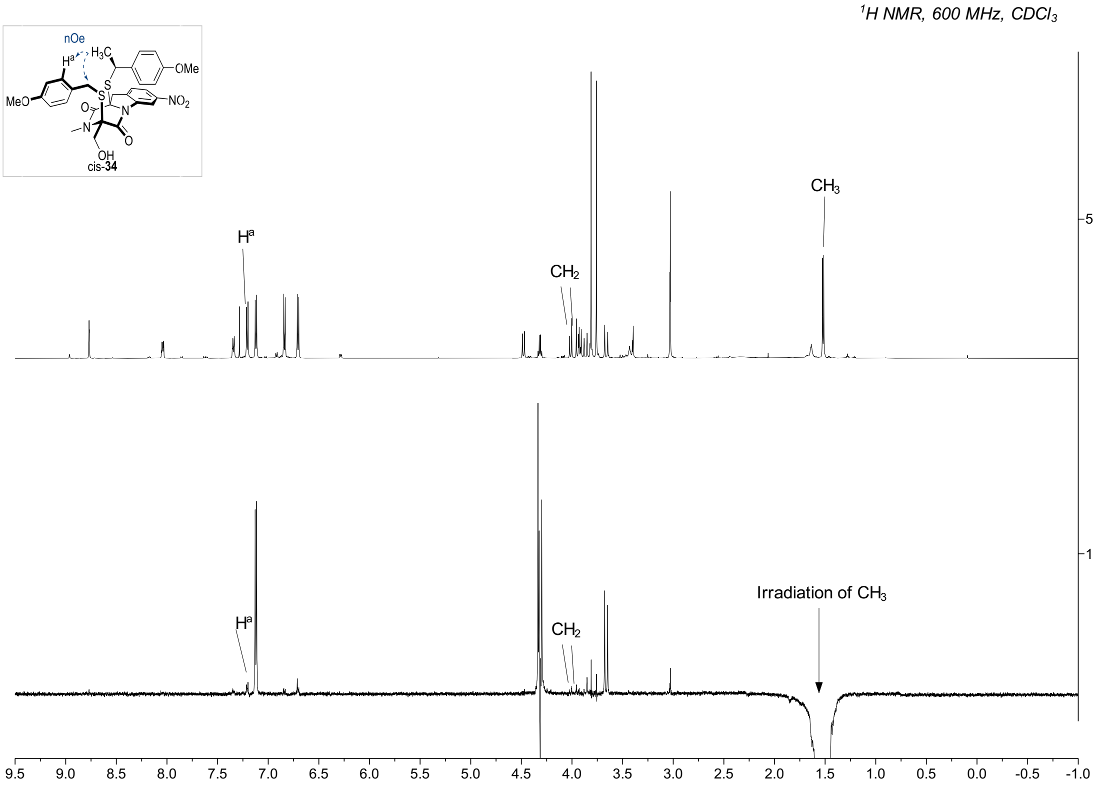

Figure S3. Diagnostic NOE correlations for cis-**34**. Irradiation of CH_3 protons on the methyl group of the S–CH(CH_3)-PMP moiety.

*IV. Single crystal X-ray diffraction (scXRD) analysis of (–)-**1**, (10aR)-**10b**, (R)-**13b**, (10aR)-**18**, and (2aR)-**25***

Single crystals covered in paratone oil were cut to size and mounted on a MiTeGen micromount loop. Data collection was performed on an Agilent Xcalibur Sapphire3 or an Agilent Enhance diffractometer equipped with a MoKα high-brilliance IμS radiation source (λ = 0.71073 Å). Absorption was corrected for using multi-scan empirical absorption correction with spherical harmonics as implemented in the SCALE3 ABSPACK scaling algorithm.[14] The structures were solved in WinGX[15] using SUPERFLIP[16] or SHELXL 2016/4[17] and refined using SHELXL 2016/4. Non-hydrogen atoms were refined anisotropically.

---

[14] CrysAlis PRO. Agilent Technologies 2011.

[15] Farrugia, L. J. *J. Appl. Crystallogr.* **2012**, *45*, 849-854.

[16] Palatinus, L.; Chapuis, G. *J. Appl. Crystallogr.* **2007**, *40* (4), 786-790.

[17] Sheldrick, G. M. *Acta Crystallogr. Sect. A* **2008**, *64*, 112-122.

scXRD data for (–)-glionitrin A (1). *Crystallization:* A suspension of (–)-glionitrin A (**1**) (20 mg) in MeCN/H_2O 60:40 (5.0 mL) was heated until boiling. The resulting clear solution was left at room temperature and then cooled to 4 °C. After 2 d at this temperature, single crystals suitable for X-ray analysis were obtained.

Table S8. Crystal data for (–)-glionitrin A (**1**).

Chemical formula	$C_{13}H_{13}N_3O_6S_2$	
Formula weight	371.38	
Collection temperature /K	293(2)	
Crystal size /mm^3	0.2 × 0.05 × 0.05	
Crystal habit	Pale yellow, rod	
Wavelength /Å	0.71073	
Crystal system	Monoclinic	
Space group	$P2_1$	
Unit cell dimensions:	a = 7.4862(9) Å	α = 90 °
	b = 6.5723(6) Å	β = 100.226(10) °
	c = 15.8866(16) Å	γ = 90 °
Unit cell volume /Å^3	769.23(14)	
Z, Calculated density /Mg/m^3	2, 1.603	
Radiation type	MoKα	
Absorption coefficient, m/mm^{-1}	0.384	
No. reflections collected / unique	5024 / 3149	
R_{int}	0.0292	
Completeness to theta = 25.000 /%	99.6	
Data / restraints / parameters	3149 / 1 / 227	
Goodness of fit on F^2	1.031	
Final R indices $(I > 2\sigma(I))$	R_1 = 0.0558, wR_2 = 0.0823	
R indices *(all data)*	R_1 = 0.0783, wR_2 = 0.0904	
Absolute structure parameter	0.01(6)	
Largest diff. peak and hole /e-/Å^3	0.398 and –0.263	
CCDC	2110183	

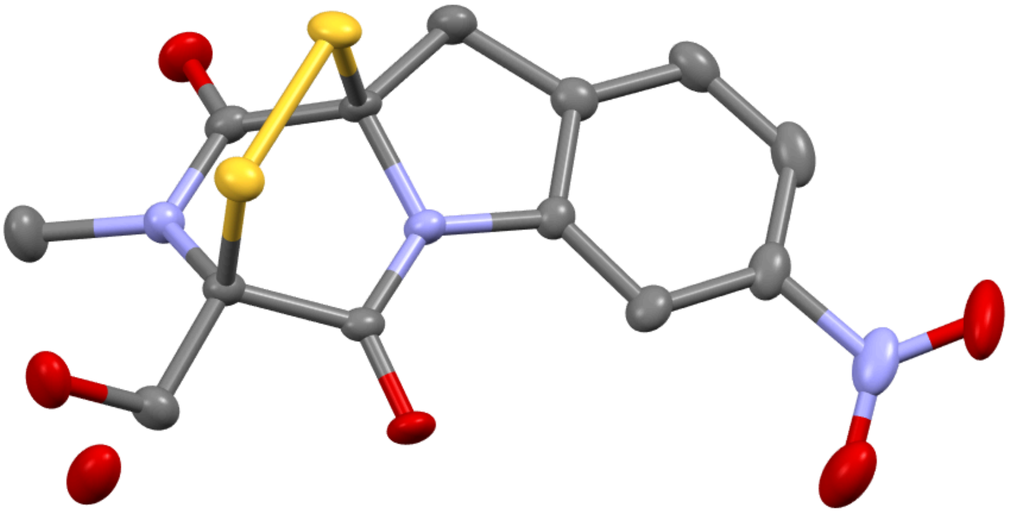

Figure S4. The asymmetric unit of (–)-glionitrin A (**1**). Black = carbon atom; red = oxygen atom; blue = nitrogen atom; yellow sulfur atom. Thermal ellipsoids are shown at 30% probability. Hydrogen atoms are omitted for clarity.

scXRD data for (*R*)-13b. *Crystallization:* A suspension of enantiomerically pure *N*-thiosuccinimide (*R*)-**13b** (3.4 g) in EtOH (70 mL) was heated until homogenous and then slowly cooled to room temperature. After 1 h, single crystals suitable for X-ray analysis were obtained.

Table S9. Crystal data for *N*-thiosuccinimide (*R*)-**13b**.

Chemical formula	$C_{13}H_{15}NO_3S$	
Formula weight	265.32	
Collection temperature /K	293(2)	
Crystal size /mm^3	0.1 × 0.03 × 0.03	
Crystal habit	Colorless, rod	
Wavelength /Å	0.71073	
Crystal system	Orthorhombic	
Space group	$P2_12_12_1$	
Unit cell dimensions:	a = 5.4410(2) Å	α = 90 °
	b = 8.7747(4) Å	β = 90 °
	c = 27.6979(11) Å	γ = 90 °
Unit cell volume /Å^3	1322.38(9)	
Z, Calculated density /Mg/m^3	4, 1.333	
Radiation type	MoKα	
Absorption coefficient, m/mm^{-1}	0.244	
No. reflections collected / unique	15420 / 3199	
R_{int}	0.0295	
Completeness to theta = 25.000 /%	99.6	
Data / restraints / parameters	3199 / 0 / 163	
Goodness of fit on F^2	1.030	
Final R indices $(I > 2\sigma(I))$	R_1 = 0.0404, wR_2 = 0.0810	
R indices *(all data)*	R_1 = 0.0625, wR_2 = 0.0912	
Absolute structure parameter	0.05(3)	
Largest diff. peak and hole /e-/Å^3	0.149 and –0.177	
CCDC	2110184	

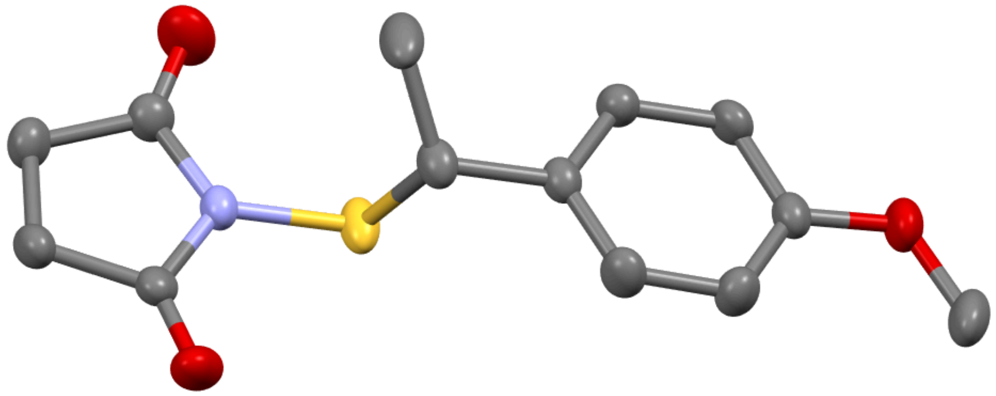

Figure S5. The asymmetric unit of *N*-thiosuccinimide (*R*)-**13b**. Black = carbon atom; red = oxygen atom; blue = nitrogen atom; yellow sulfur atom. Thermal ellipsoids are shown at 30% probability. Hydrogen atoms are omitted for clarity.

scXRD data for (10a*R*)-10b. *Crystallization:* A suspension of thioaminal (10a*R*)-**10b** (25 mg) in EtOAc/*n*-heptane 1:2 (3.0 mL) was heated until homogenous and then slowly cooled to room temperature. After 24 h, single crystals suitable for X-ray analysis were obtained.

Table S10. Crystal data for thioaminal (10a*R*)-**10b**.

Chemical formula	$C_{21}H_{19}N_3O_6S$	
Formula weight	441.45	
Collection temperature /K	293(2)	
Crystal size /mm^3	0.3 × 0.3 × 0.05	
Crystal habit	Colorless, plate	
Wavelength /Å	0.71073	
Crystal system	Orthorhombic	
Space group	$P2_12_12_1$	
Unit cell dimensions:	a = 7.2684(4) Å	α = 90 °
	b = 7.7310(3) Å	β = 90 °
	c = 38.513(2) Å	γ = 90 °
Unit cell volume /Å^3	2164.11(18)	
Z, Calculated density /Mg/m^3	4, 1.355	
Radiation type	MoKα	
Absorption coefficient, m/mm^{-1}	0.192	
No. reflections collected / unique	23614 / 5041	
R_{int}	0.0738	
Completeness to theta = 25.000 /%	99.6	
Data / restraints / parameters	5041 / 0 / 283	
Goodness of fit on F^2	1.002	
Final R indices $(I > 2\sigma(I))$	R_1 = 0.0629, wR_2 = 0.0947	
R indices *(all data)*	R_1 = 0.1561, wR_2 = 0.1183	
Absolute structure parameter	0.06(7)	
Largest diff. peak and hole /e-/Å^3	0.156 and –0.178	
CCDC	2110186	

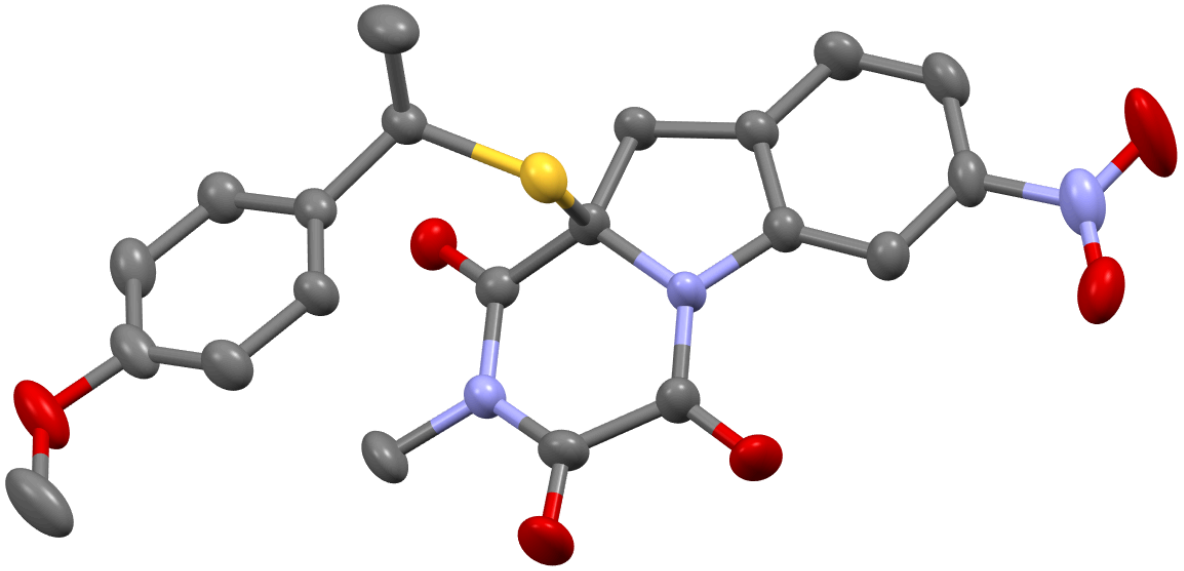

Figure S6. The asymmetric unit of thioaminal (10a*R*)-**10b**. Black = carbon atom; red = oxygen atom; blue = nitrogen atom; yellow = sulfur atom. Thermal ellipsoids are shown at 30% probability. Hydrogen atoms are omitted for clarity.

scXRD data for (2a*R*)-25. *Crystallization:* A suspension of thioaminal (2a*R*)-**25** (15 mg) in EtOH (1.5 mL) was heated until homogenous and then slowly cooled to room temperature. After 16 h, single crystals suitable for X-ray analysis were obtained.

Table S11. Crystal data for thioaminal (2a*R*)-**25**.

Chemical formula	$C_{17}H_{20}N_2O_4S$	
Formula weight	348.41	
Collection temperature /K	293(2)	
Crystal size /mm^3	0.3 × 0.05 × 0.05	
Crystal habit	Colorless, rod	
Wavelength /Å	0.71073	
Crystal system	Orthorhombic	
Space group	$P2_12_12_1$	
Unit cell dimensions:	a = 6.4608(6) Å	α = 90 °
	b = 8.3434(7) Å	β = 90 °
	c = 31.9966(19) Å	γ = 90 °
Unit cell volume /Å^3	1724.8(2)	
Z, Calculated density /Mg/m^3	4, 1.342	
Radiation type	MoKα	
Absorption coefficient, m/mm^{-1}	0.211	
No. reflections collected / unique	17851 / 4016	
R_{int}	0.1125	
Completeness to theta = 25.000 /%	99.6	
Data / restraints / parameters	4016 / 0 / 220	
Goodness of fit on F^2	0.958	
Final R indices $(I > 2\sigma(I))$	R_1 = 0.0625, wR_2 = 0.0828	
R indices *(all data)*	R_1 = 0.1790, wR_2 = 0.1095	
Absolute structure parameter	0.00(10)	
Largest diff. peak and hole /e-/Å^3	0.209 and –0.218	
CCDC	2110187	

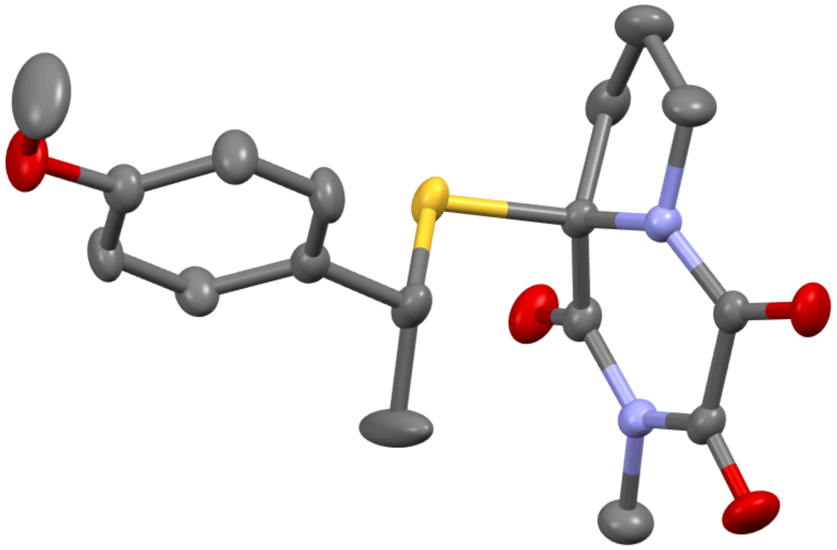

Figure S7. The asymmetric unit of thioaminal (2a*R*)-**25**. Black = carbon atom; red = oxygen atom; blue = nitrogen atom; yellow = sulfur atom. Thermal ellipsoids are shown at 30% probability. Hydrogen atoms are omitted for clarity.

scXRD data for (10a*R*)-18. *Crystallization:* A solution of thioaminal (10a*R*)-**18** (17 mg) in EtOH (10 mL) was left to evaporate slowly. After 3 d, single crystals suitable for X-ray analysis were obtained.

Table S12. Crystal data for thioaminal (10a*R*)-**18**.

Chemical formula	$C_{21}H_{20}N_2O_4S$	
Formula weight	396.45	
Collection temperature /K	293(2)	
Crystal size /mm^3	0.3 × 0.3 × 0.3	
Crystal habit	Colorless, cube	
Wavelength /Å	0.71073	
Crystal system	Monoclinic	
Space group	$P2_1$	
Unit cell dimensions:	a = 8.5189(9) Å	α = 90 °
	b = 18.0102(10) Å	β = 107.851(11) °
	c = 13.5404(12) Å	γ = 90 °
Unit cell volume /Å^3	1977.4(3)	
Z, Calculated density /Mg/m^3	4, 1.332	
Radiation type	MoKα	
Absorption coefficient, m/mm^{-1}	0.193	
No. reflections collected / unique	16130 / 8789	
R_{int}	0.0429	
Completeness to theta = 25.000 /%	99.7	
Data / restraints / parameters	8789 / 1 / 511	
Goodness of fit on F^2	1.036	
Final R indices $(I > 2\sigma(I))$	R_1 = 0.0715, wR_2 = 0.0950	
R indices *(all data)*	R_1 = 0.1362, wR_2 = 0.1158	
Absolute structure parameter	–0.07(5)	
Largest diff. peak and hole /e-/Å^3	0.351 and –0.238	
CCDC	2110185	

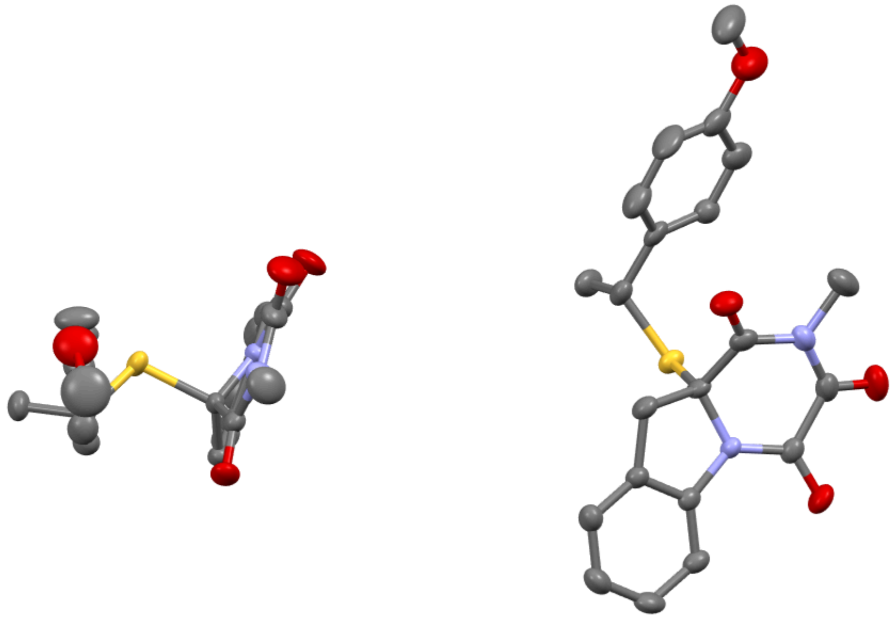

Figure S8. Asymmetric unit of thioaminal (10a*R*)-**18**. Black = carbon atom; red = oxygen atom; blue = nitrogen atom; yellow = sulfur atom. Thermal ellipsoids are shown at 30% probability. Hydrogen atoms are omitted for clarity.

*V. Expanded optimization of the stereoselective sulfenylation of triketopiperazine rac-**9***

General procedure for the sulfenylation of triketopiperazine *rac*-9 with *N*-thiosuccinimide 13a. Triketopiperazine *rac*-**9** (27.5 mg, 100 μmol), *N*-thiosuccinimide **13a**[5] (27.6 mg, 110 μmol), and catalyst (10.0 μmol) were dissolved in CH_2Cl_2 (2 mL) at room temperature. After stirring for the indicated amount of time, HCl (2 mL, aq., 1M) was added in one portion. The resulting mixture was passed through a phase separator and the filtrate was concentrated under reduced pressure. The resulting crude residue was purified by column chromatography and analyzed by enantioselective HPLC unless stated otherwise. Analytically pure thioaminal **10a** was obtained through purification by column chromatography (50-100% EtOAc/*n*-heptane).

R_f: 0.47 in 50% EtOAc/*n*-heptane. Stains yellow with $KMnO_4$ stain.

^{1}H NMR (DMSO-d_6, 400 MHz): δ 8.53 (d, *J* = 2.4 Hz, 1H), 8.19 (dd, *J* = 8.2, 2.4 Hz, 1H), 7.73 (d, *J* = 8.2 Hz, 1H), 7.15-7.07 (m, 2H), 6.86-6.78 (m, 2H), 3.93 (d, *J* = 17.6 Hz, 1H), 3.88 (d, *J* = 13.6 Hz, 1H), 3.79 (d, *J* = 13.6 Hz, 1H), 3.71 (s, 3H), 3.65 (d, *J* = 17.6 Hz, 1H), 3.05 (s, 3H) ppm.

^{13}C NMR (DMSO-d_6, 101 MHz): δ 166.6, 158.5, 156.5, 150.0, 147.2, 139.8, 137.4, 129.8, 126.7, 126.5, 121.9, 114.0, 111.7, 73.5, 55.1, 40.0, 33.8, 27.4 ppm.

FTIR (film): 2921 (w), 1690 (s), 1527 (w), 1511 (w), 1351 (w), 1252 (w), 739 (w) cm^{-1}.

HRMS-ESI (*m/z*): $[M + Na]^+$ Calcd for $C_{20}H_{17}N_3NaO_6S$ 450.0736, Found 450.0727.

mp: 190-192 °C (obtained by recrystallization from EtOH).

HPLC: Chiralpak® AS-RH column, 5μm silica-gel, MeCN/H_2O = 60/40, flow rate = 0.5 mL/min, λ = 254 nm. t_R = 9.85 min ((10a*R*)-**10a**) and 11.38 min ((10a*S*)-**10a**).[18]

[18] The absolute configuration of **10a** was assigned by comparison of optical rotation following conversion of a sample enriched in (10a*R*)-**10a** to enantiomerically enriched (–)-(*R*,*R*)-glionitrin.

Table S13. HPLC chromatogram of *rac*-**10a**.

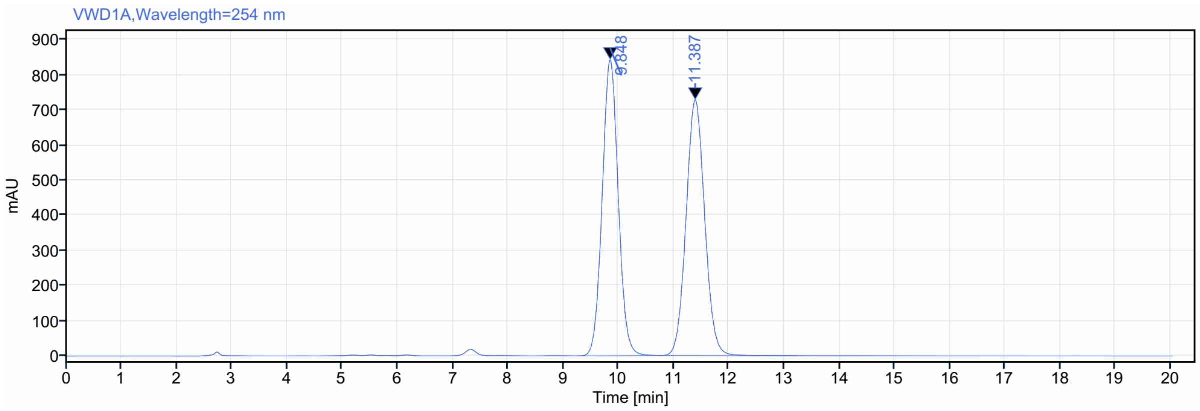

Peak	Retention time (min)	Height [mV.s]	Area (%)
1	9.85	843.71	49.9
2	11.38	728.77	50.1

Table S14. Optimization of the stereoselective sulfenylation of *rac*-**9** using *N*-thiosuccinimide **13a**.[a]

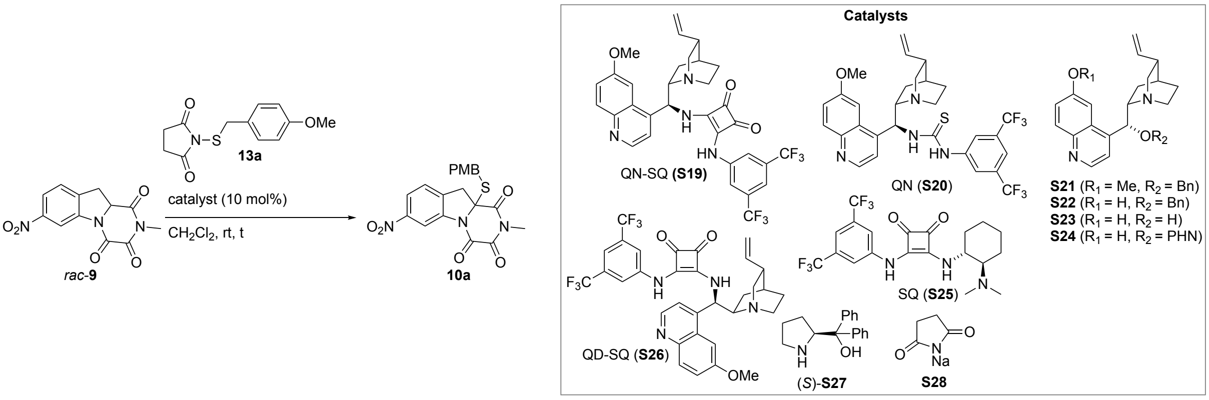

Entry	Catalyst	Time	Yield (%)[a]	er (10a*R*:10a*S*)[b]
1	-	23 h	- [c]	-
2	Et_3N	16 h	58	-
3	**S28**[19]	16 h	9	-
4	Quinidine	15 h	75	47:53
5	Cinchonine	15 h	78	49:51
6	Cinchonidine	15 h	80	65:35
7	$(DHQH)_2$PHAL	15 h	27	48:52
8	**S21** (R_1 = Me, R_2 = Bn)[20]	15 h	17	47:53
9	**S22** (R_1 = H, R_2 = Bn)[20]	15 h	22	41:59
10	**S23** (R_1 = H, R_2 = H)[20]	15 h	22	50:50
11	**S24** (R_1 = H, R_2 = PHN)[20]	15 h	6	41:59
12	QN-SQ (**S19**)[21]	15 h	47	34:66
13	QD-SQ (**S26**)[21]	15 h	47	59:41
14	QN-(**S20**)[22]	15 h	21	66:34
15	SQ-(**S25**)[23]	15 h	37	54:64
16	(*S*)-**S27**	15 h	17	66:34

a) Determined by ^{1}H NMR spectroscopy of the crude residue using an internal standard; b) Determined by enantioselective HPLC; c) No reaction. PMB = *p*-methoxybenzyl, Bn = benzyl, PHN = phenanthrene.

General procedure for the sulfenylation of triketopiperazine *rac*-9 with S29–S30 catalyzed by quinine (11): Triketopiperazine *rac*-**9** (27.5 mg, 100 μmol), electrophile (110 μmol), and quinine (**11**) (3.2 mg, 10.0 μmol) were dissolved in the CH_2Cl_2 (2 mL) at room temperature. After stirring for 16 h, HCl (2 mL, aq., 1M) was added. The resulting mixture was passed

[19] Prepared following: Muchowski, J.M.; Nelson, Peter H. *Tetrahedron Lett.* **1980**, *21*, 4585-4588.

[20] Prepared following: Li, W.; Wang, Y.; Tang, L.; Deng, L. *J. Am. Chem. Soc.* **2004**, *126*, 9906-9907.

[21] Prepared following: Sorrentino, E.; Connon, S.J. *Org. Lett.* **2016**, *18*, 5204-5207.

[22] Prepared following: Vakulya, B.; Varga, S.; Csámpai, A.; Sóos, T. *Org. Lett.* **2005**, *7*, 1967-1969.

[23] Prepared following: Konishi, H.; Lam, T.Y.; Malerich, J.P.; Rawal, V.H. *Org. Lett.* **2010**, *12*, 2028-2031.

through a phase separator and the filtrate was concentrated under reduced pressure. The resulting crude residue was analyzed by HPLC

Table S15. Sulfenylation of triketopiperazine *rac*-**9** with electrophiles **S29**–**S30**.

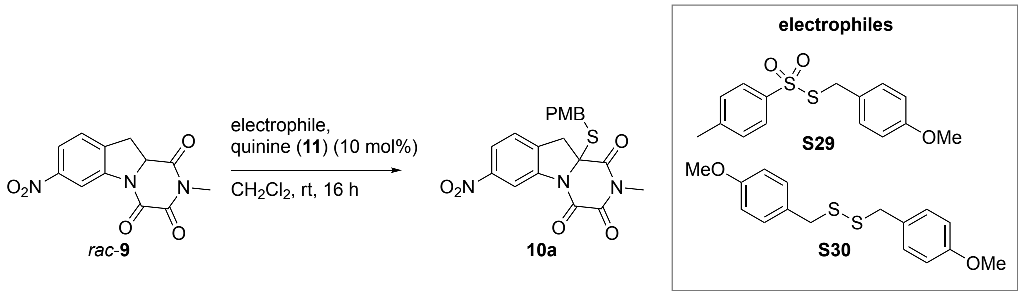

**Entry**	**Electrophile**	**Yield** (%)[a]	**er** (10a*R*:10a*S*)[b]
1	**S29**[24]	9	66:34
2	**S30**[25]	- [c]	-

a) Determined by ^{1}H NMR analysis of the crude residue using an internal standard; b) Determined by enantioselective HPLC; c) No reaction.

[24] Prepared following: Kimura, S.; Bill, E.; Bothe, E.; Weyhermüller, T.; Wieghardt, K. *J. Am. Chem. Soc.* **2001**, *123*, 6025-6039.

[25] Prepared following: Fritze, U.F.; Delius, M. *Chem. Commun.* **2016**, *52*, 6363-6366.

*VI. Reassignment of the absolute configuration of natural (–)-glionitrin A (**1**) and (–)-glionitrin B (**2**)*

The absolute configuration of natural (–)-glionitrin A was originally assigned as *S*,*S* based on the similarity of features in its CD spectrum to the related structure gliotoxin.[26] A scXRD structure of natural (–)-glionitrin A was reported, but no Flack parameter was given.[26] The absolute configuration of natural (–)-glionitrin B was originally assigned as *S*,*S* by comparison of the CD spectra of natural (–)-glionitrin B with semisynthetic (–)-glionitrin B obtained by from (–)-glionitrin A.[27]

The absolute configuration of synthetic (–)-glionitrin A produced by the herein described method was assigned as *R*,*R* based on scXRD analysis (Flack = 0.01(6)). This is opposite to that of the proposed structure for natural (–)-glionitrin A. Synthetic (–)-glionitrin B, formed by semisynthesis from synthetic (–)-glionitrin A, using the herein described method, was assigned as *R*,*R* under the assumption that the stereocenters are unchanged in this transformation. The formation of (–)-glionitrin B as a single detected enantiomer by enantioselective HPLC supports this assumption.

The circular dichroism spectra of synthetic (–)-glionitrin A and (–)-glionitrin B recorded in MeCN (0.1 mg/mL) conforms to data reported for the natural products.[26-27]

Based on these observations, we conclude that natural (–)-glionitrin A and natural (–)-glionitrin B should both be reassigned to the (*R*,*R*)-configuration.

---

[26] Park, H. B.; Kwon, H. C.; Lee, C.; Yang, H. O. *J. Nat. Prod.* **2009**, *72*, 248–252.

[27] Park, H.B.; Kim, Y.J.; Park, J.S.; Yang, H.O.; Lee, K.R.; Kwon, H. C. *J. Nat Prod.* **2011**, *74*, 2309–2312.

VII. Density Functional Theory calculations

All quantum mechanical calculations were performed with Jaguar as implemented in Schrödinger release 2020-1,[28] using default settings except that the SCF accuracy level was set to accurate with a switch to analytical integrals near convergence. Gas phase energy minima and transition state geometries were determined employing density functional theory (DFT) with the m06-2x functional and the 6-31G** basis set.[29] Vibrational frequencies were calculated for all stationary points to obtain the Gibbs Free Energies. A singular negative eigenvalue was found for saddle points. Total free energies are given at 298.15 K and 1.0 atm. Structures are visualized using CYLview 2.0.[30] Atom coordinates of each calculated species are given in the calc_structures.pdb file available for download as supporting information.

Transition state structures leading to each diastereomeric product of 10b. The asymmetric sulfenylation of enolate **16** with a reactive intermediate formed from (*R*)-**12b** and (*R*)-**13b** was modeled. The lowest energy transition states leading to each diastereomeric product respectively were:

TS-minor **S31** *leading to (10aS)-**10b***

Gibbs free energy: –2436.09283 a.u.

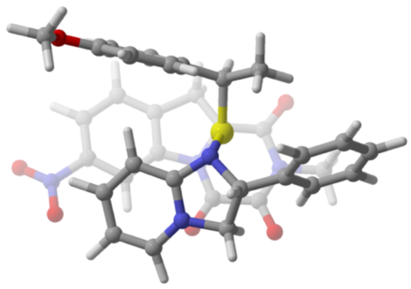

Figure S9. TS-minor **S31**.

TS-major ***17*** *leading to (10aR)-***10b**

Gibbs free energy: –2436.095882 a.u.

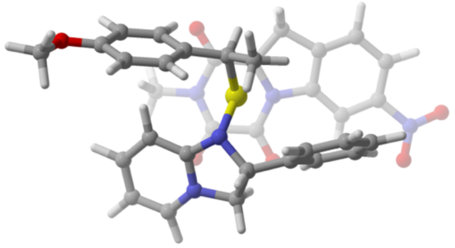

Figure S10. TS-major **17**.

[28] Schrödinger Release 2020-1: Schrödinger, LLC, New York, NY, 2020.

[29] Bochevarov, A. D.; Harder, E.; Hughes, T. F.; Greenwood, Jeremy R.; Braden, D. A.; Philipp, D. M.; Rinaldo, D.; Halls, M. D.; Zhang, J.; Friesner, R. A. Jaguar: A high-performance quantum chemistry software program with strengths in life and materials sciences. *Int. J. Quantum Chem.* **2013**, *113*, 2110-2142.

[30] CYLview20; Legault, C. Y., Université de Sherbrooke, 2020 (http://www.cylview.org).

Comparison of the heat of formation between 36/S32 and 1/S33. Cleavage of the S–S motif in (–)-glionitrin A (**1**) with **36** was modeled as an isodesmic reaction. The gas phase energies were calculated using the Jaguar workflow for Heat of Formation, employing the DFT/M06-2x functional and the 6-311++g-3df-3pd basis set, both for geometry optimizations and for energy calculations. The results support that the reaction is thermodynamically favored by ΔΔG = 2.84 kcal/mol at 298K. For atomic coordinates of **36**/**S32** and **1**/**S33** in .xyz format, see the accompanying file: calc_structures.xyz.

Table S16. Comparison of gas phase energy for the isodesmic reaction of **36**/**S32** and **1**/**69**.

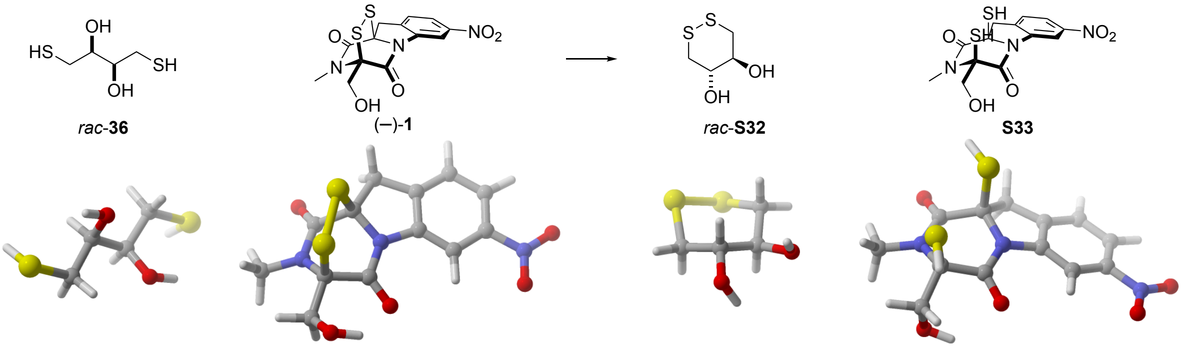

Atomization energy **36** (298K):	1545.11 kcal/mol	Atomization energy **S32** (298K):	1431.41 kcal/mol
ΔH of formation **36** (298K):	–99.59 kcal/mol	ΔH of formation **S32** (298K):	–89.15 kcal/mol
Atomization energy **1** (298K):	3619.51 kcal/mol	Atomization energy **S33** (298K):	3736.05 kcal/mol
ΔH of formation **1** (298K):	–77.98 kcal/mol	ΔH of formation **S33** (298K):	–91.26 kcal/mol

*VIII. Copies of ^{1}H and ^{13}C NMR spectra for **1-2, 9, 10a-b, 13b, 18, 20, 22-23, 25, 27, 30-34, S1, S4, S6-S8, S10, S12, S15**, and **S17-S18***

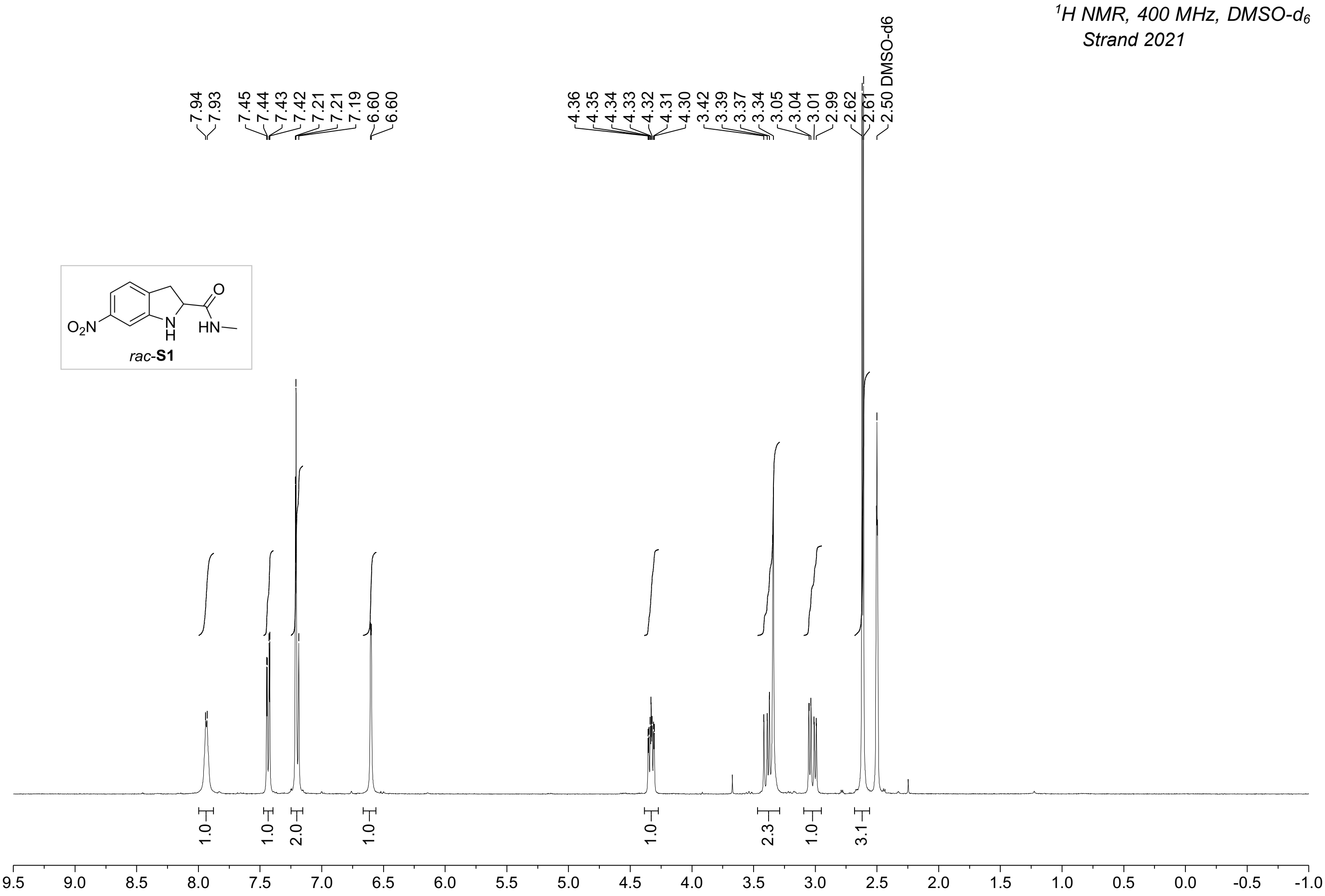

^{1}H NMR, 400 MHz, DMSO-d_6
Strand 2021
7.94 7.93 7.45 7.44 7.43 7.42 7.21 7.21 7.19 6.60 6.60
4.36 4.35 4.34 4.33 4.32 4.31 4.30 3.42 3.39 3.37 3.34 3.05 3.04 3.01 2.99 2.62 2.61 2.50 DMSO-d6
O
O_2N
N
H
HN
rac-S1
1.0 1.0 2.0 1.0 1.0 2.3 1.0 3.1
9.5 9.0 8.5 8.0 7.5 7.0 6.5 6.0 5.5 5.0 4.5 4.0 3.5 3.0 2.5 2.0 1.5 1.0 0.5 0.0 -0.5 -1.0

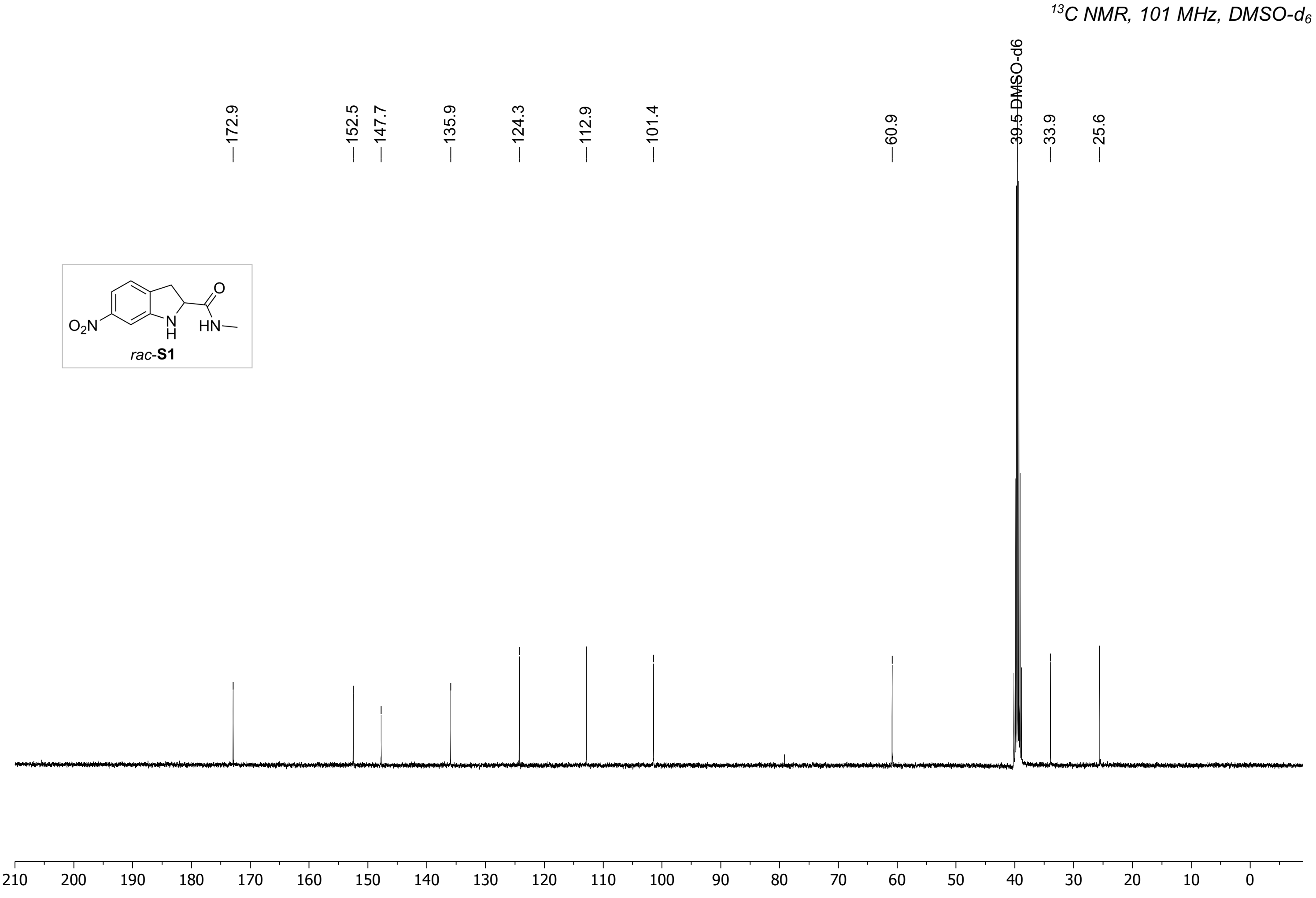

^{13}C NMR, 101 MHz, DMSO-d_6
172.9 152.5 147.7 135.9 124.3 112.9 101.4 60.9 39.5 DMSO-d6 33.9 25.6
O
O_2N
N
H
HN
rac-S1
210 200 190 180 170 160 150 140 130 120 110 100 90 80 70 60 50 40 30 20 10 0

^{1}H NMR, 400 MHz, DMSO-d_6
Strand 2021

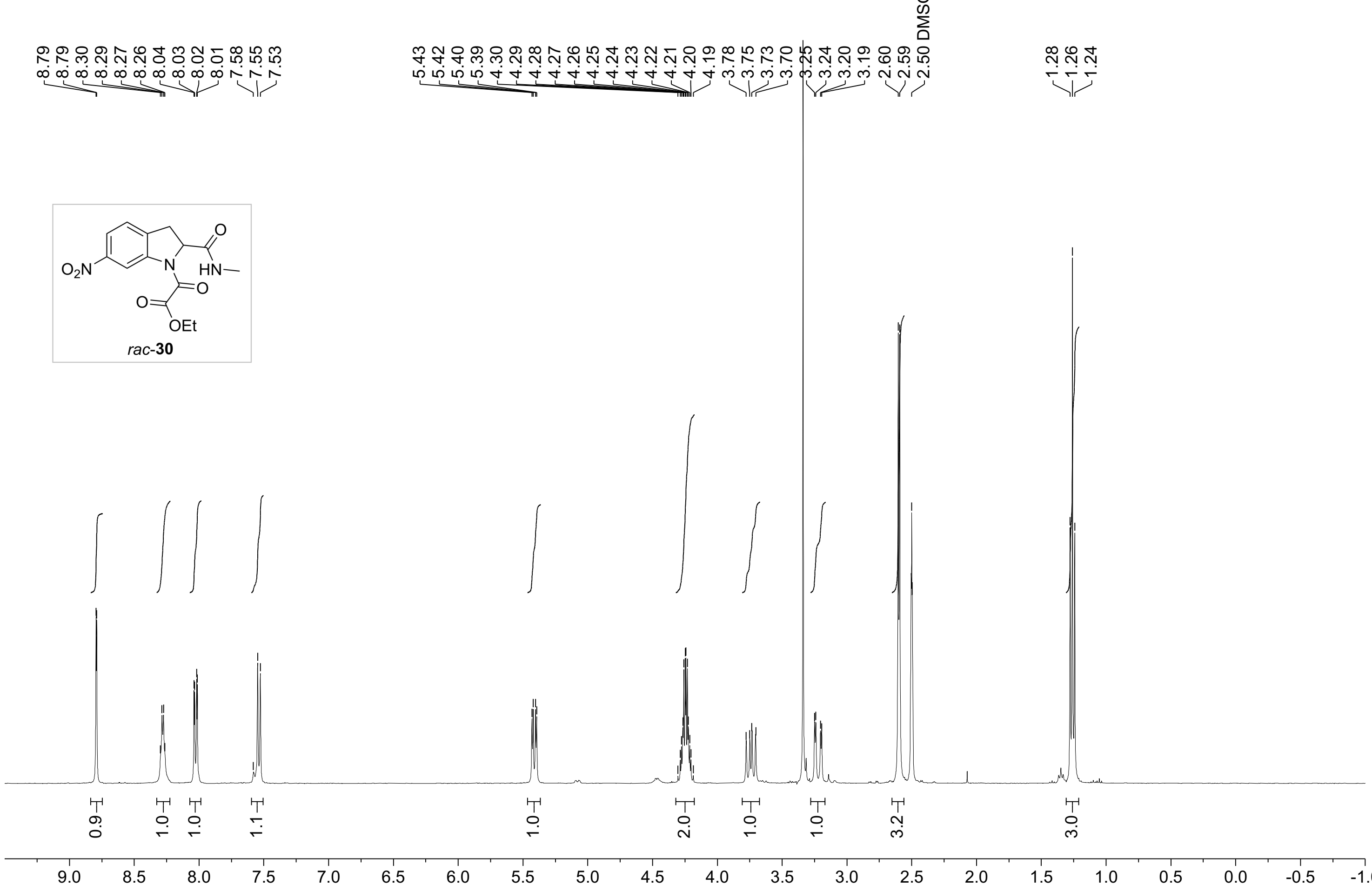

^{13}C NMR, 101 MHz, DMSO-d_6

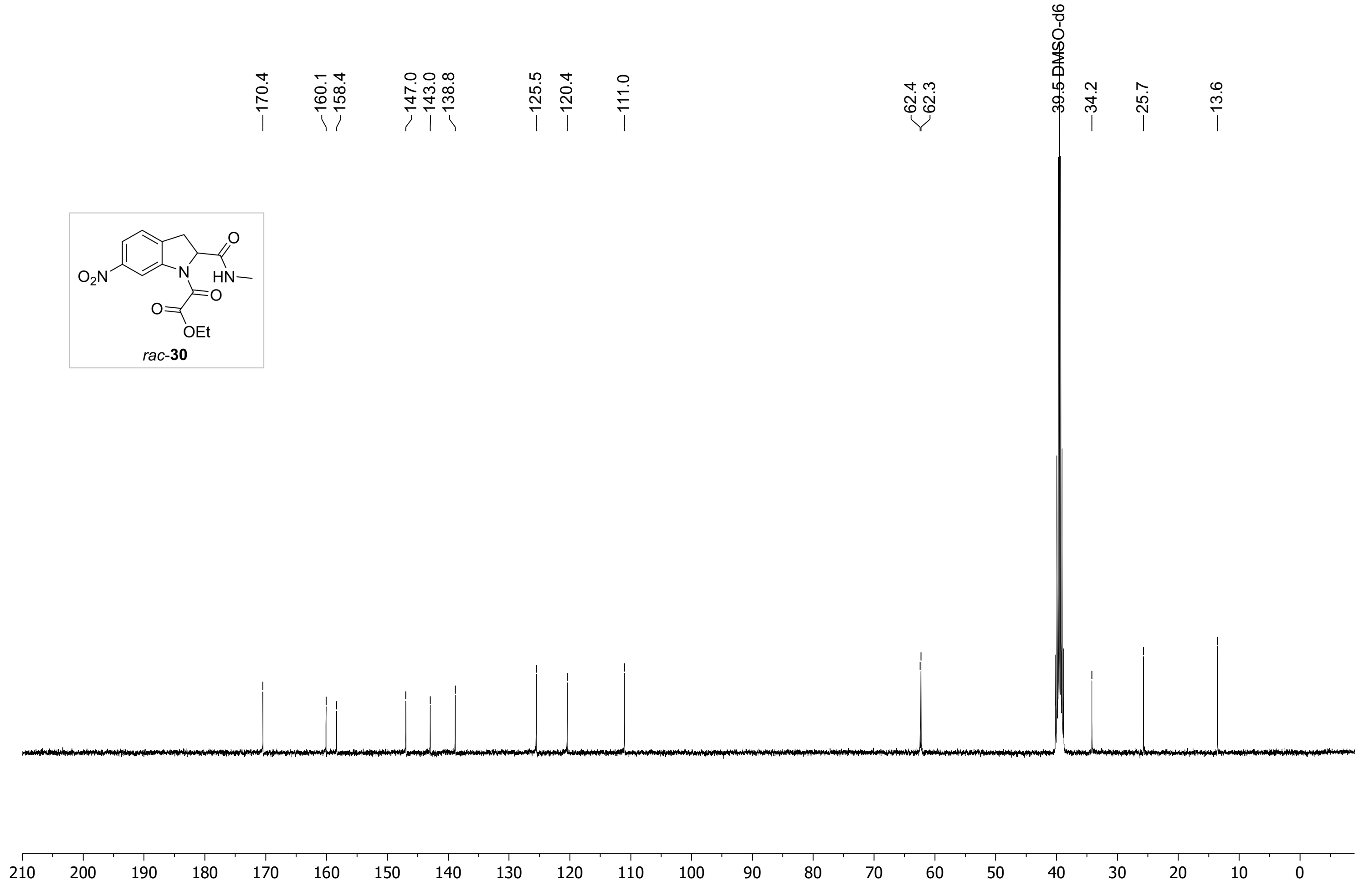

¹H NMR, 400 MHz, DMSO-d₆
Strand 2021

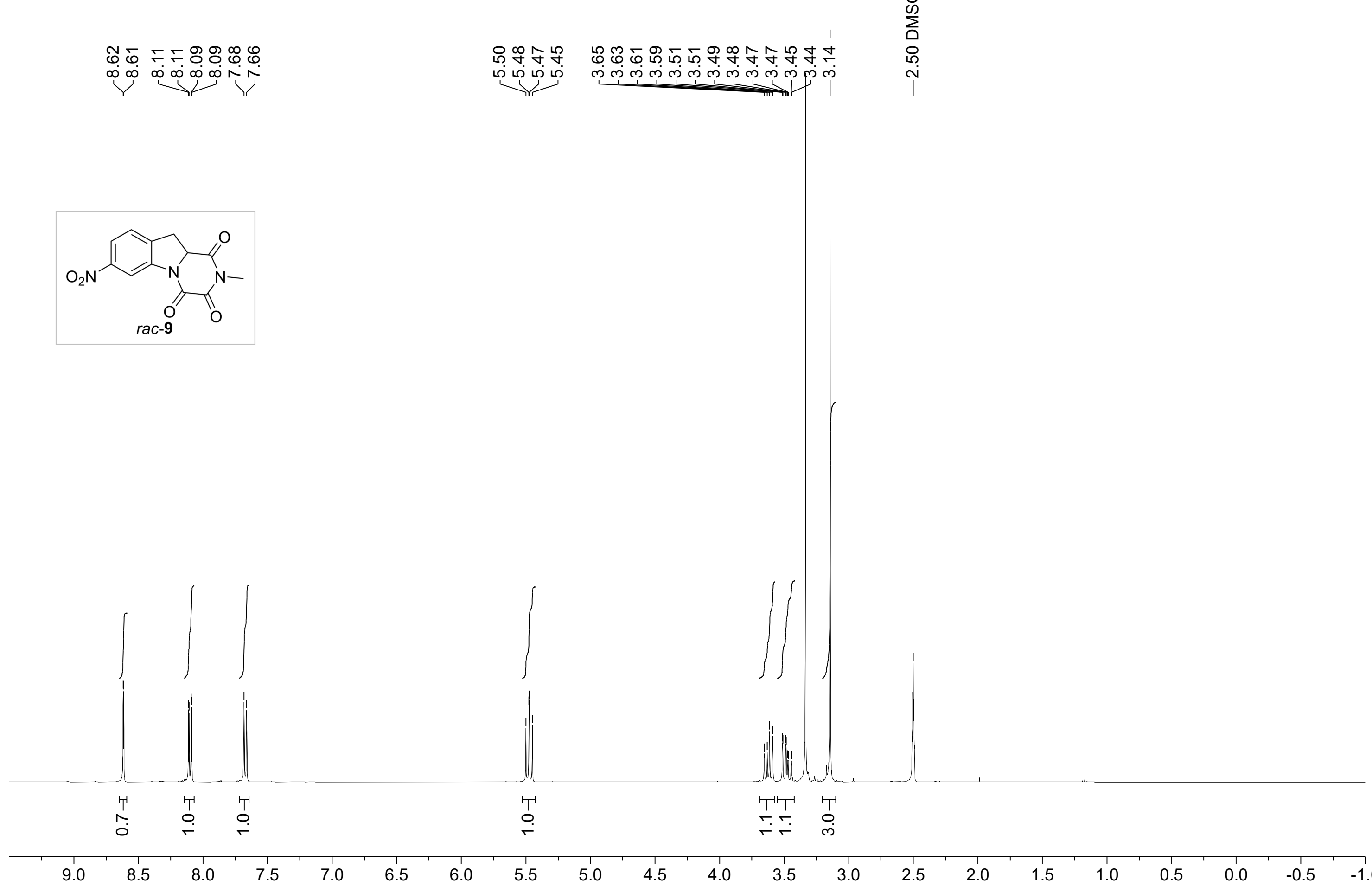

¹³C NMR, 101 MHz, DMSO-d₆

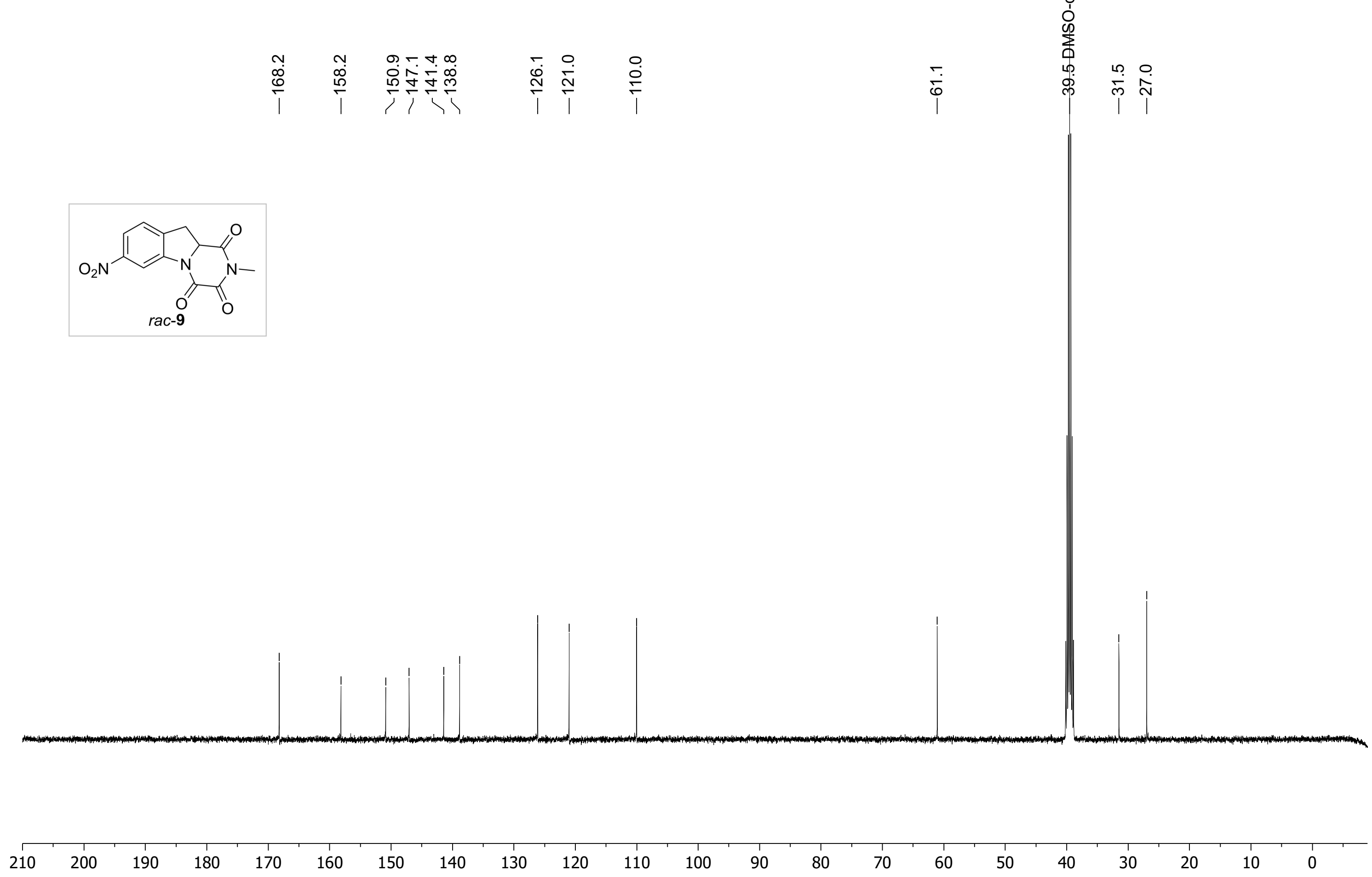

^{1}H NMR, 400 MHz, $CDCl_3$
Strand 2021

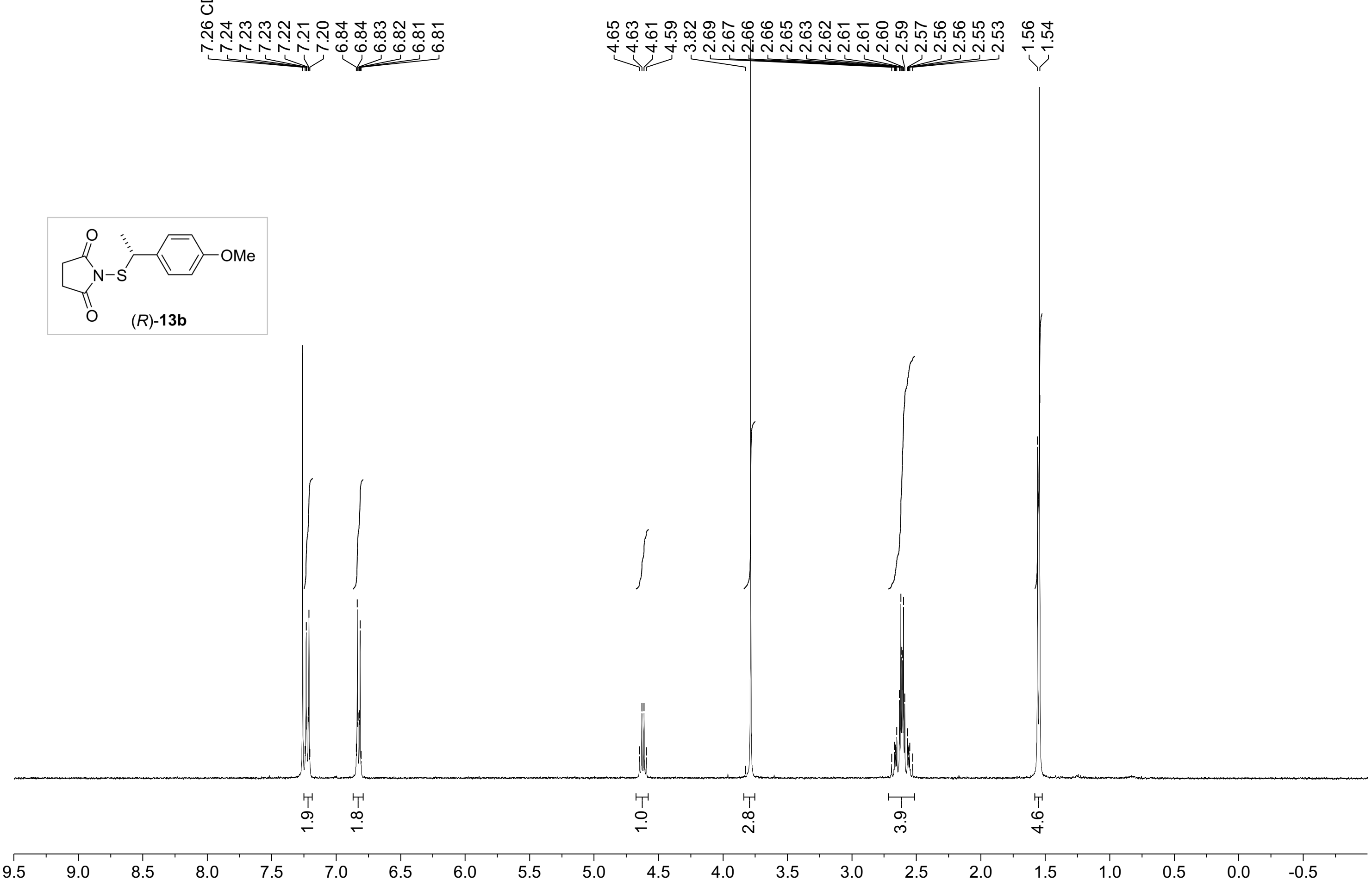

^{13}C NMR, 101 MHz, $CDCl_3$

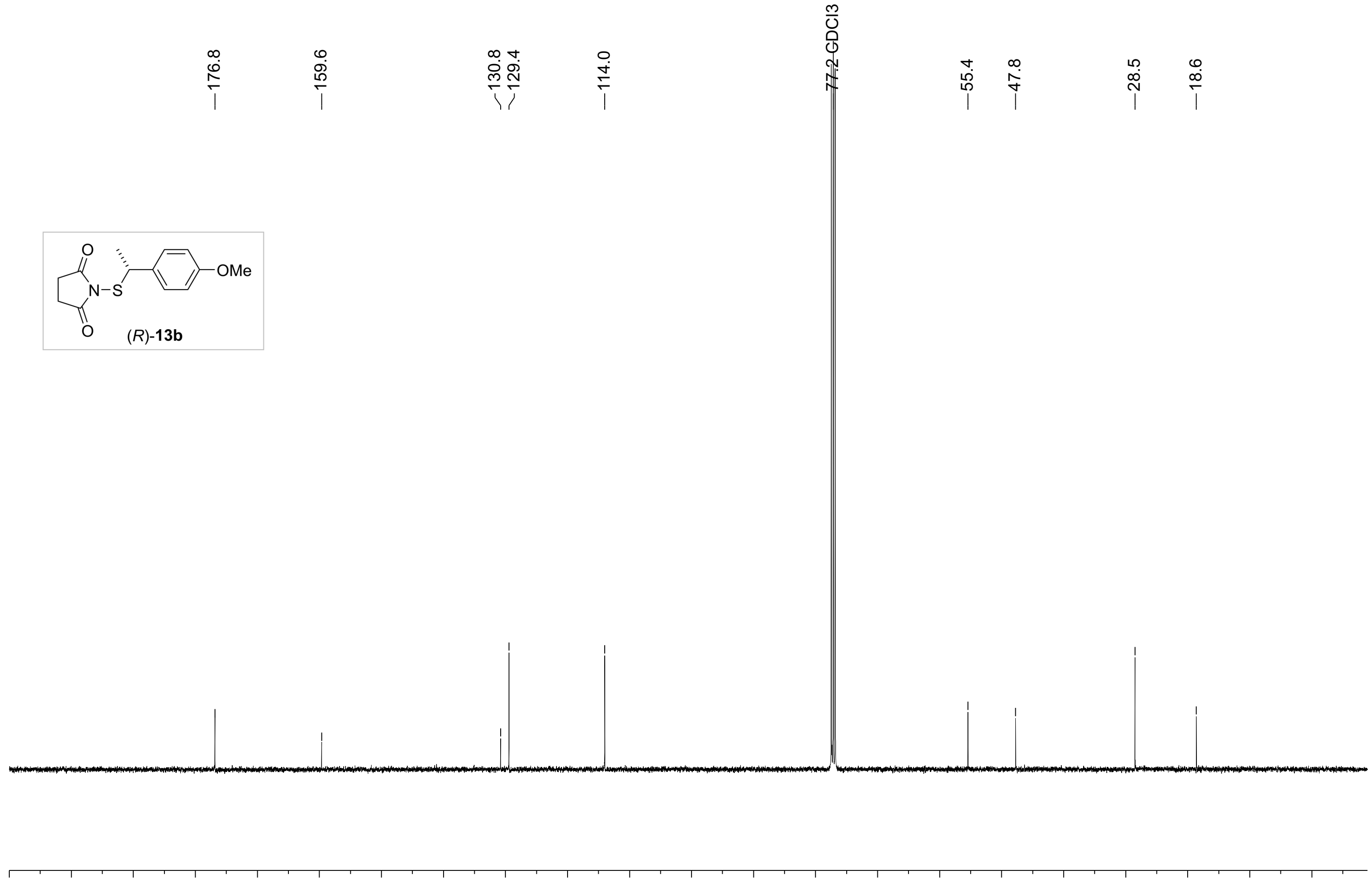

^{1}H NMR, 400 MHz, DMSO-d_6
Strand 2021

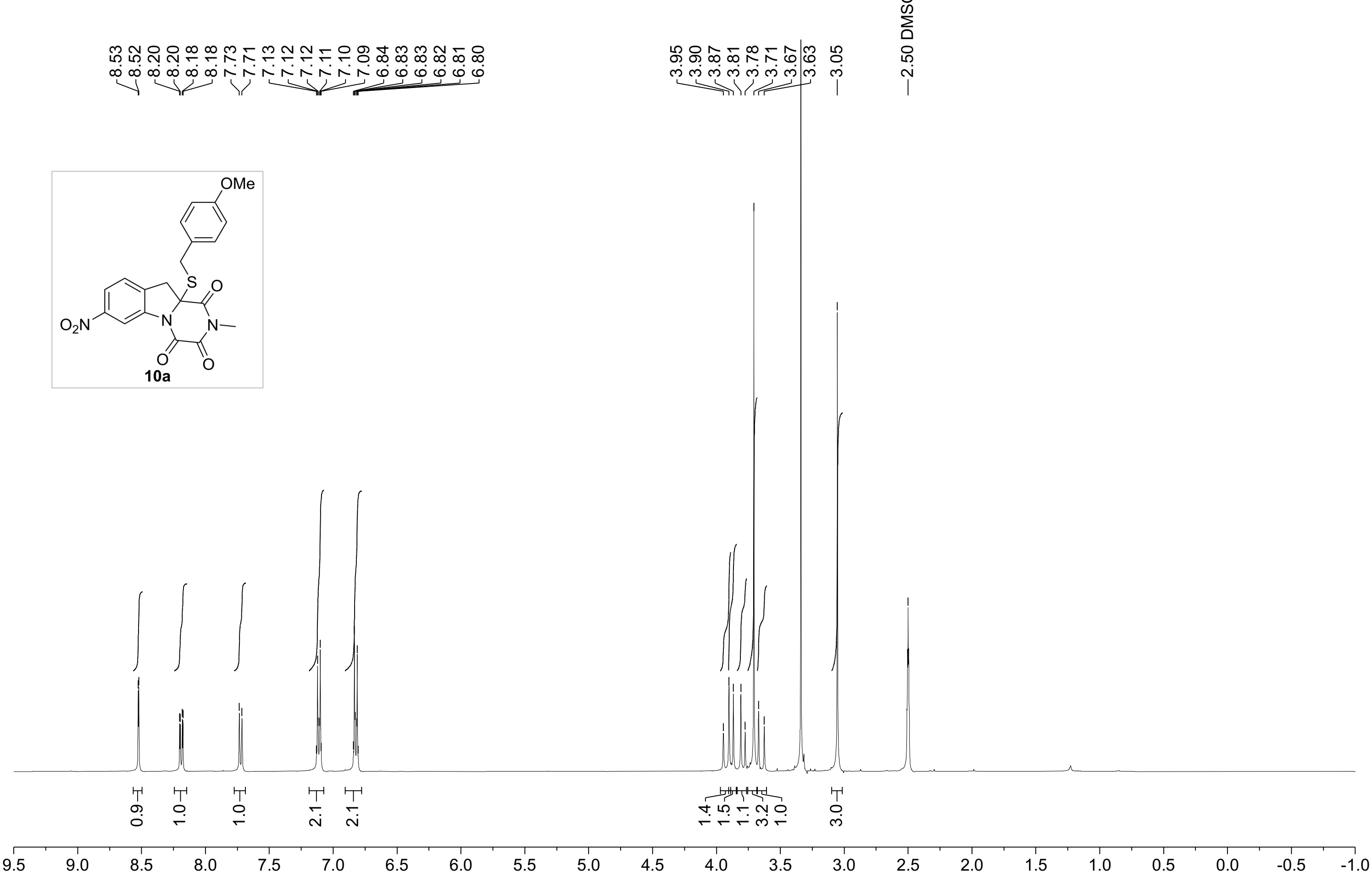

^{13}C NMR, 101 MHz, DMSO-d_6

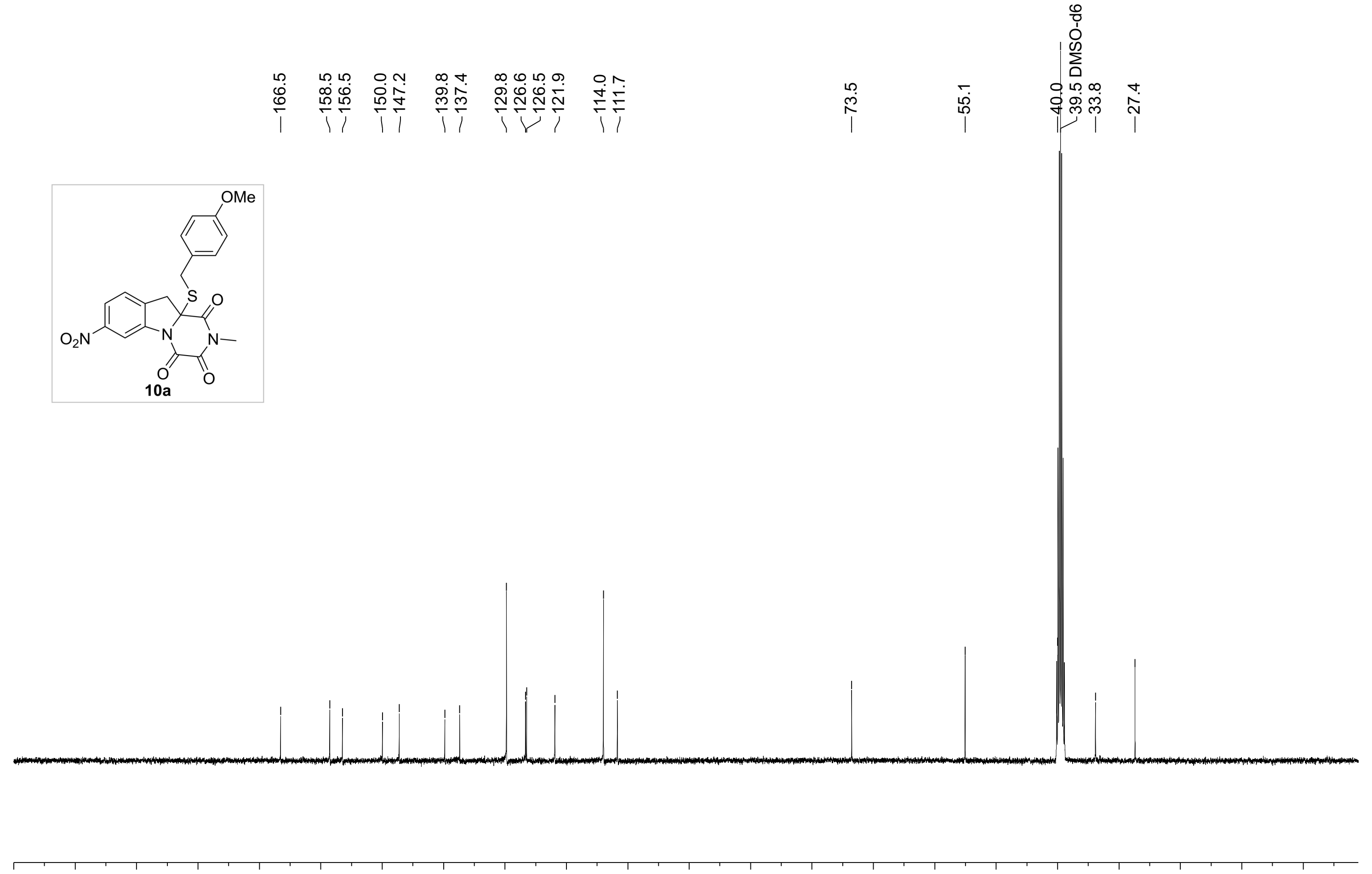

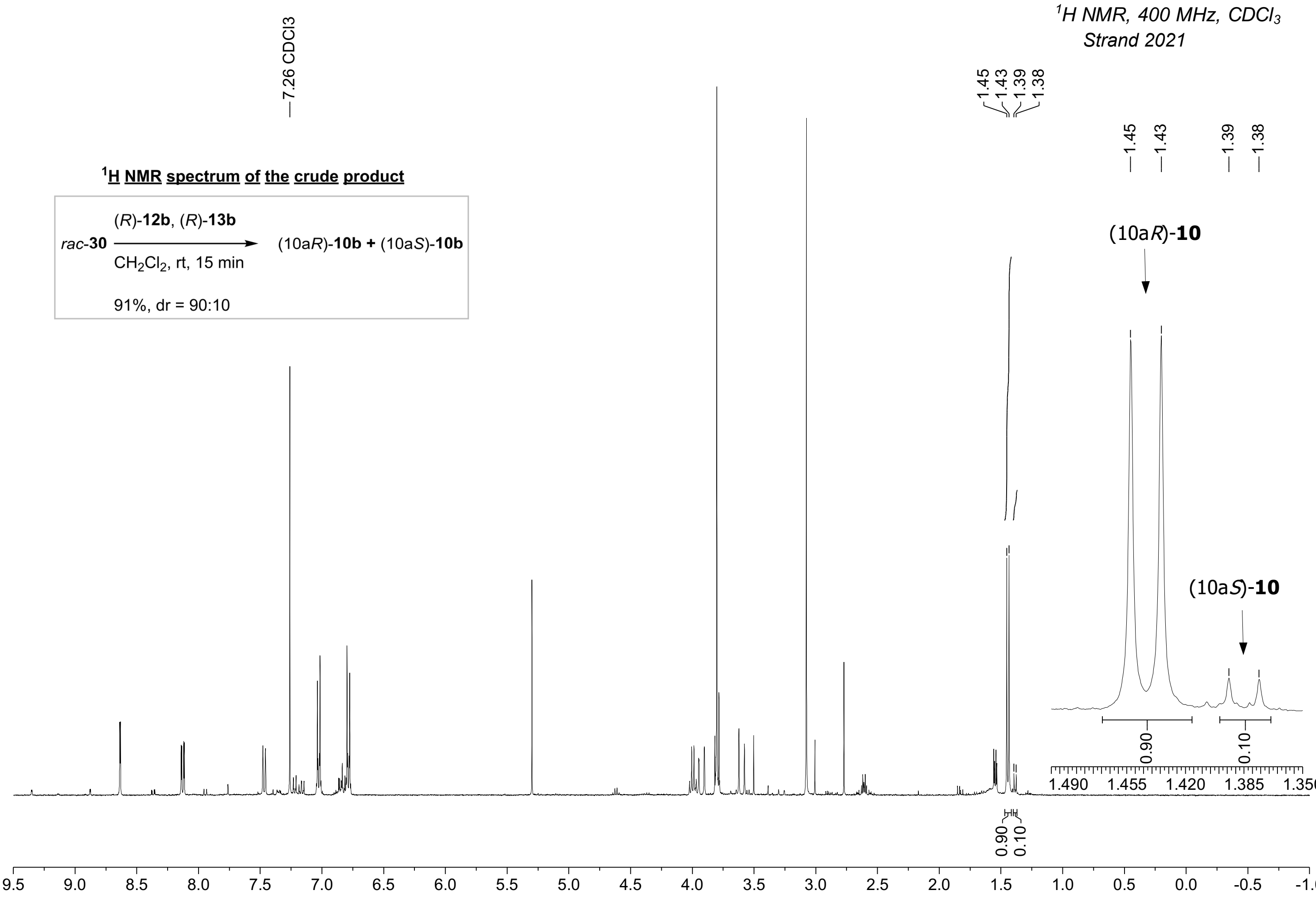
1H NMR, 400 MHz, CDCl3
Strand 2021
7.26 CDCl3
1.45
1.43
1.39
1.38
1H NMR spectrum of the crude product
(R)-12b, (R)-13b
rac-30
(10aR)-10b + (10aS)-10b
CH2Cl2, rt, 15 min
91%, dr = 90:10
(10aR)-10
(10aS)-10
0.90
0.10
1.490 1.455 1.420 1.385 1.350
9.5 9.0 8.5 8.0 7.5 7.0 6.5 6.0 5.5 5.0 4.5 4.0 3.5 3.0 2.5 2.0 1.5 1.0 0.5 0.0 -0.5 -1.0

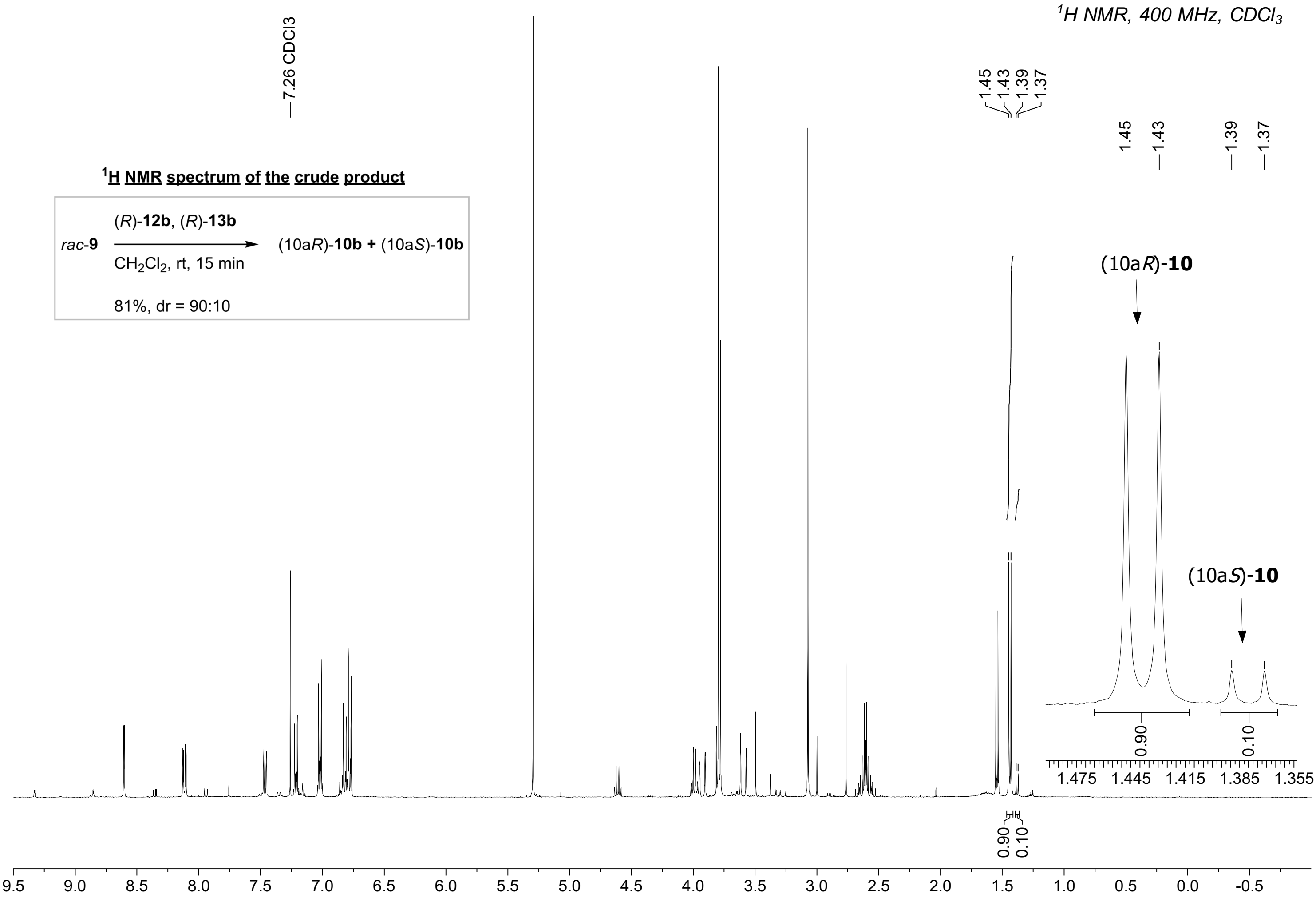
1H NMR, 400 MHz, CDCl3
7.26 CDCl3
1.45
1.43
1.39
1.37
1H NMR spectrum of the crude product
(R)-12b, (R)-13b
rac-9
(10aR)-10b + (10aS)-10b
CH2Cl2, rt, 15 min
81%, dr = 90:10
(10aR)-10
(10aS)-10
0.90
0.10
1.475 1.445 1.415 1.385 1.355
9.5 9.0 8.5 8.0 7.5 7.0 6.5 6.0 5.5 5.0 4.5 4.0 3.5 3.0 2.5 2.0 1.5 1.0 0.5 0.0 -0.5

^{1}H NMR, 400 MHz, $CDCl_3$
Strand 2021

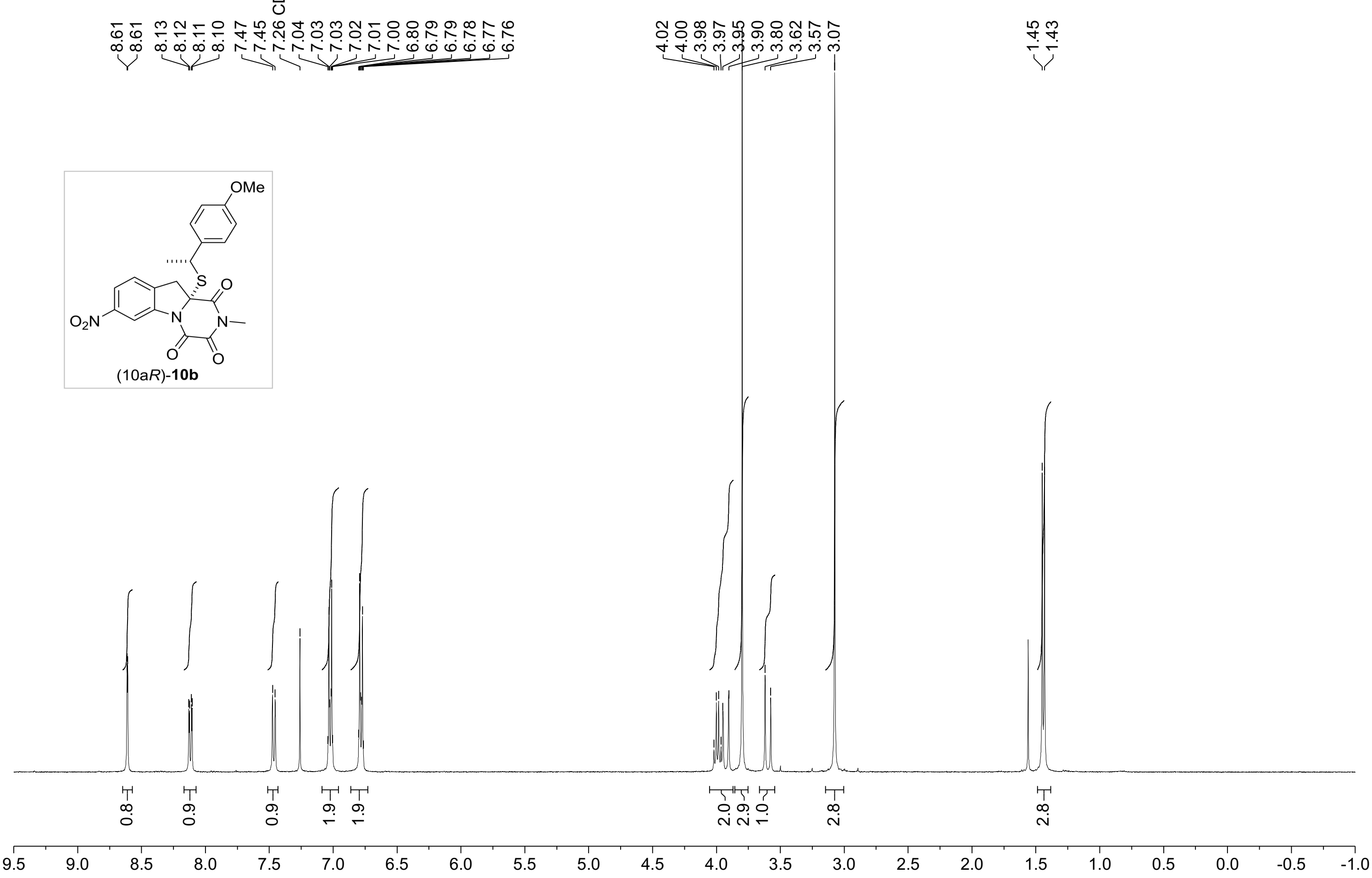

^{13}C NMR, 101 MHz, $CDCl_3$

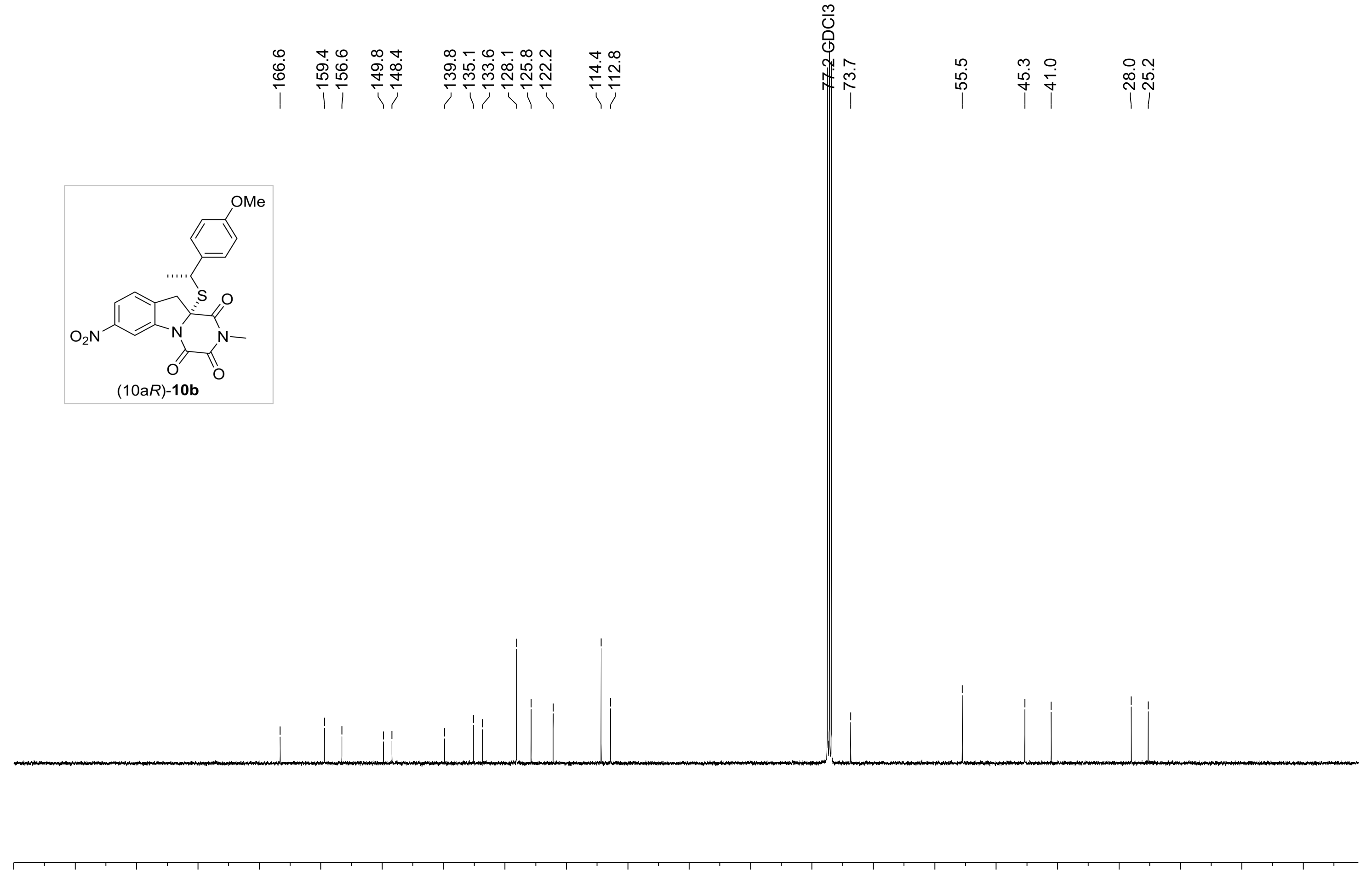

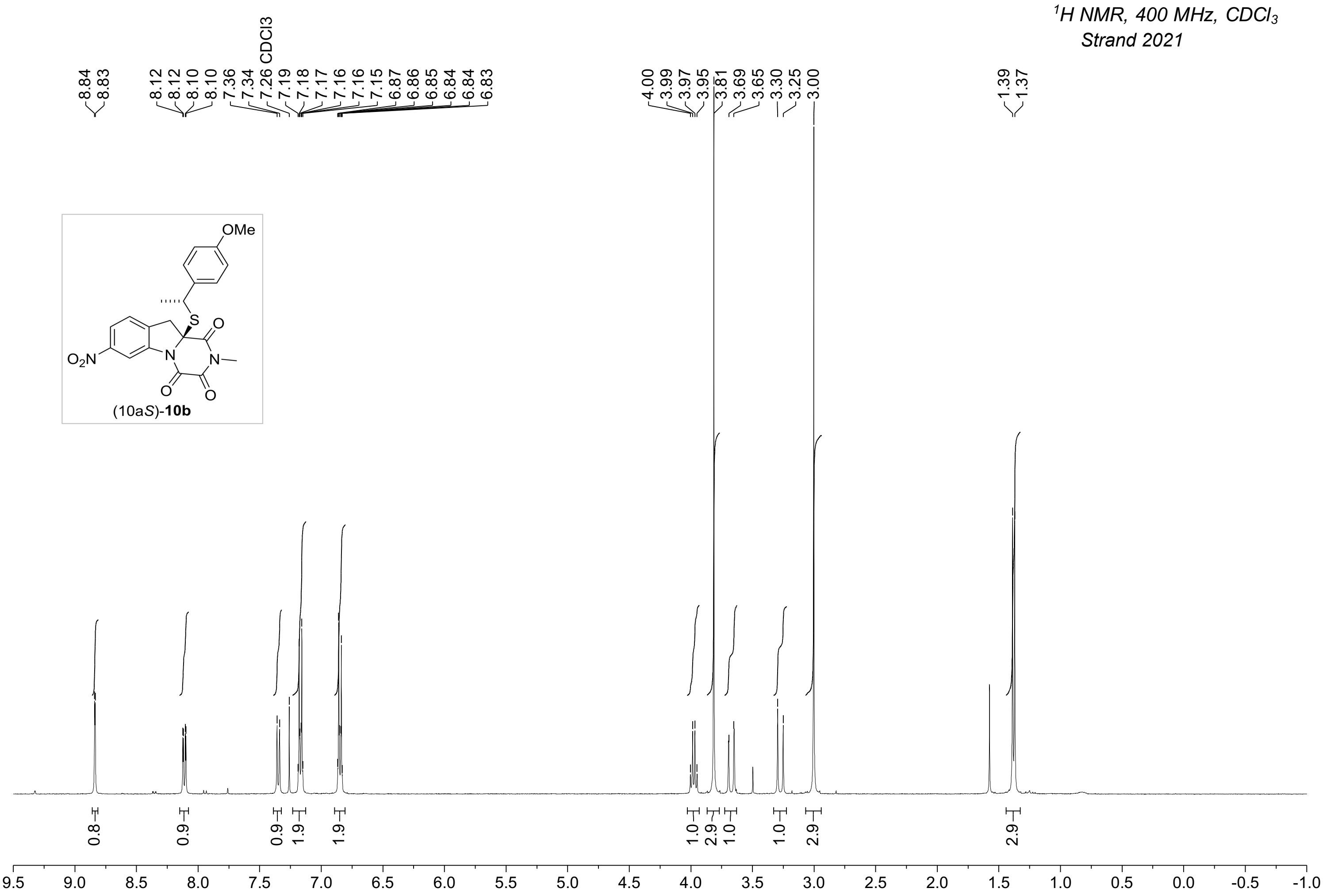

^{1}H NMR, 400 MHz, $CDCl_3$
Strand 2021
8.84 8.83 8.12 8.12 8.10 8.10 7.36 7.34 7.26 CDCl3 7.19 7.18 7.17 7.16 7.16 7.15 6.87 6.86 6.85 6.84 6.84 6.83
4.00 3.99 3.97 3.95 3.81 3.69 3.65 3.30 3.25 3.00
1.39 1.37
OMe
S
O
O_2N
N
N
O
O
(10a*S*)-**10b**
0.8 0.9 0.9 1.9 1.9 1.0 2.9 1.0 1.0 2.9 2.9
9.5 9.0 8.5 8.0 7.5 7.0 6.5 6.0 5.5 5.0 4.5 4.0 3.5 3.0 2.5 2.0 1.5 1.0 0.5 0.0 -0.5 -1.0

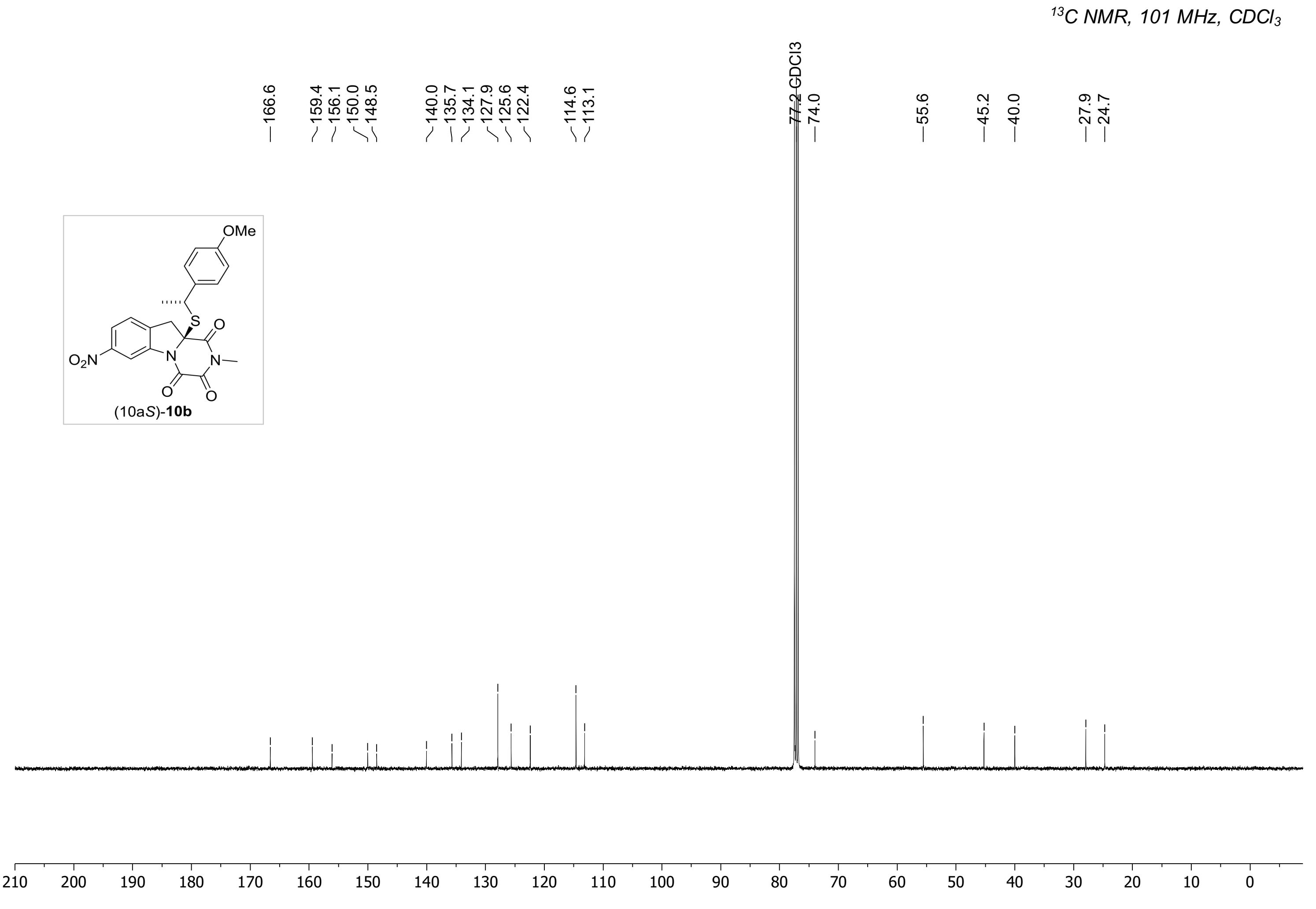

^{13}C NMR, 101 MHz, $CDCl_3$
166.6 159.4 156.1 150.0 148.5 140.0 135.7 134.1 127.9 125.6 122.4 114.6 113.1
77.2 CDCl3 74.0 55.6 45.2 40.0 27.9 24.7
OMe
S
O
O_2N
N
N
O
O
(10a*S*)-**10b**
210 200 190 180 170 160 150 140 130 120 110 100 90 80 70 60 50 40 30 20 10 0

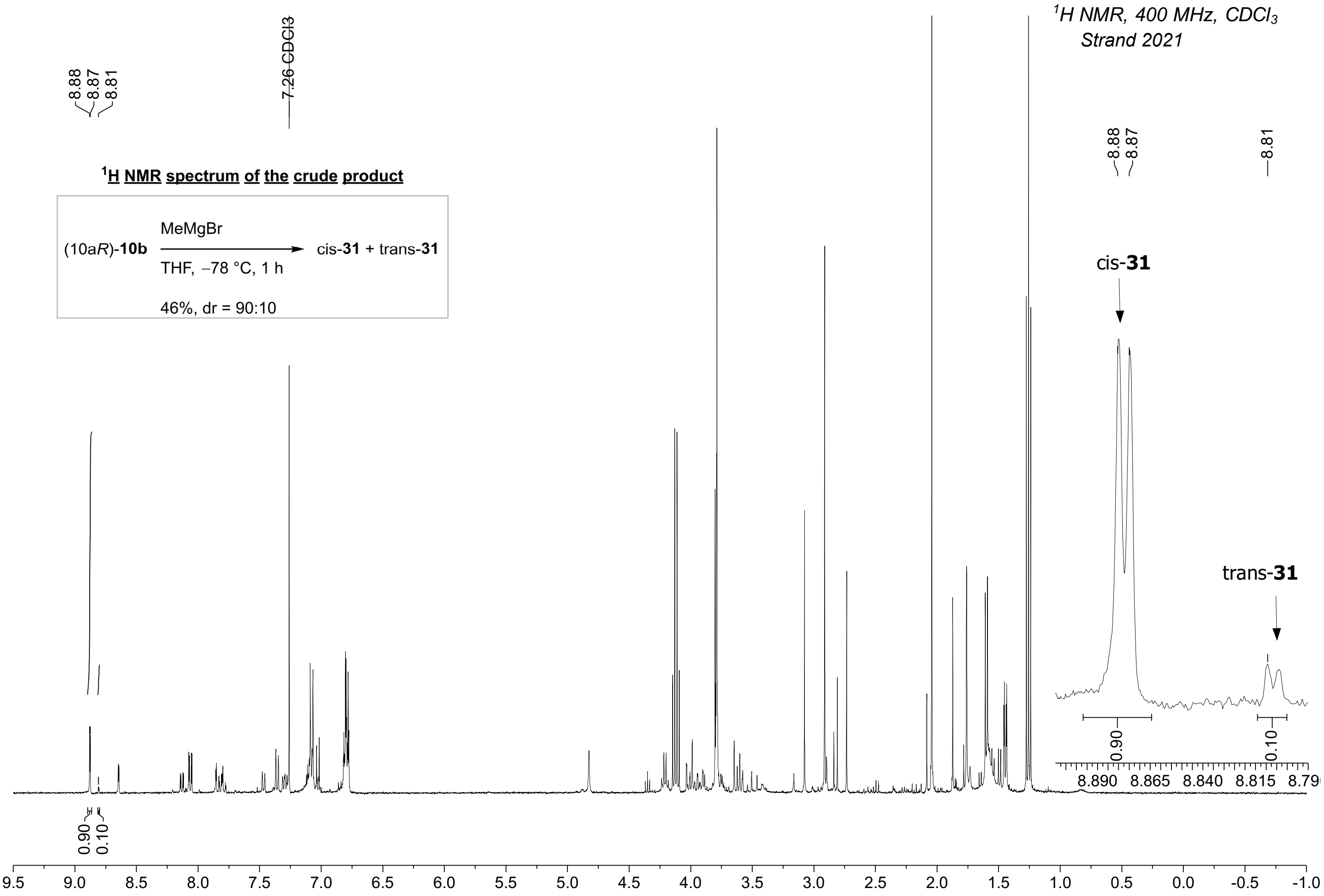

1H NMR, 400 MHz, CDCl3
Strand 2021
1H NMR spectrum of the crude product
(10aR)-10b
MeMgBr
THF, –78 °C, 1 h
cis-31 + trans-31
46%, dr = 90:10
cis-31
trans-31

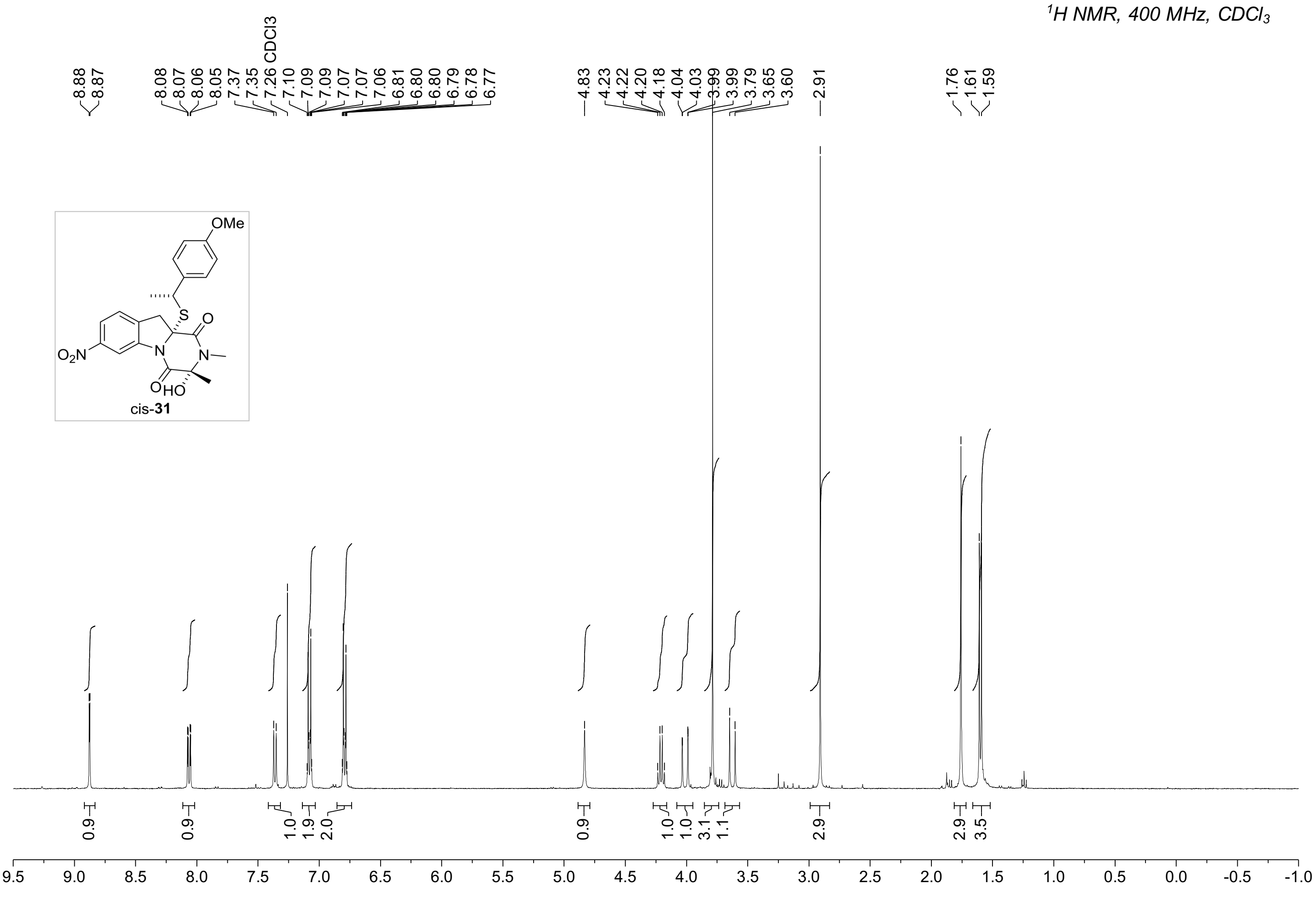

1H NMR, 400 MHz, CDCl3
OMe
S
O
N
O2N
N
O
HO
cis-31

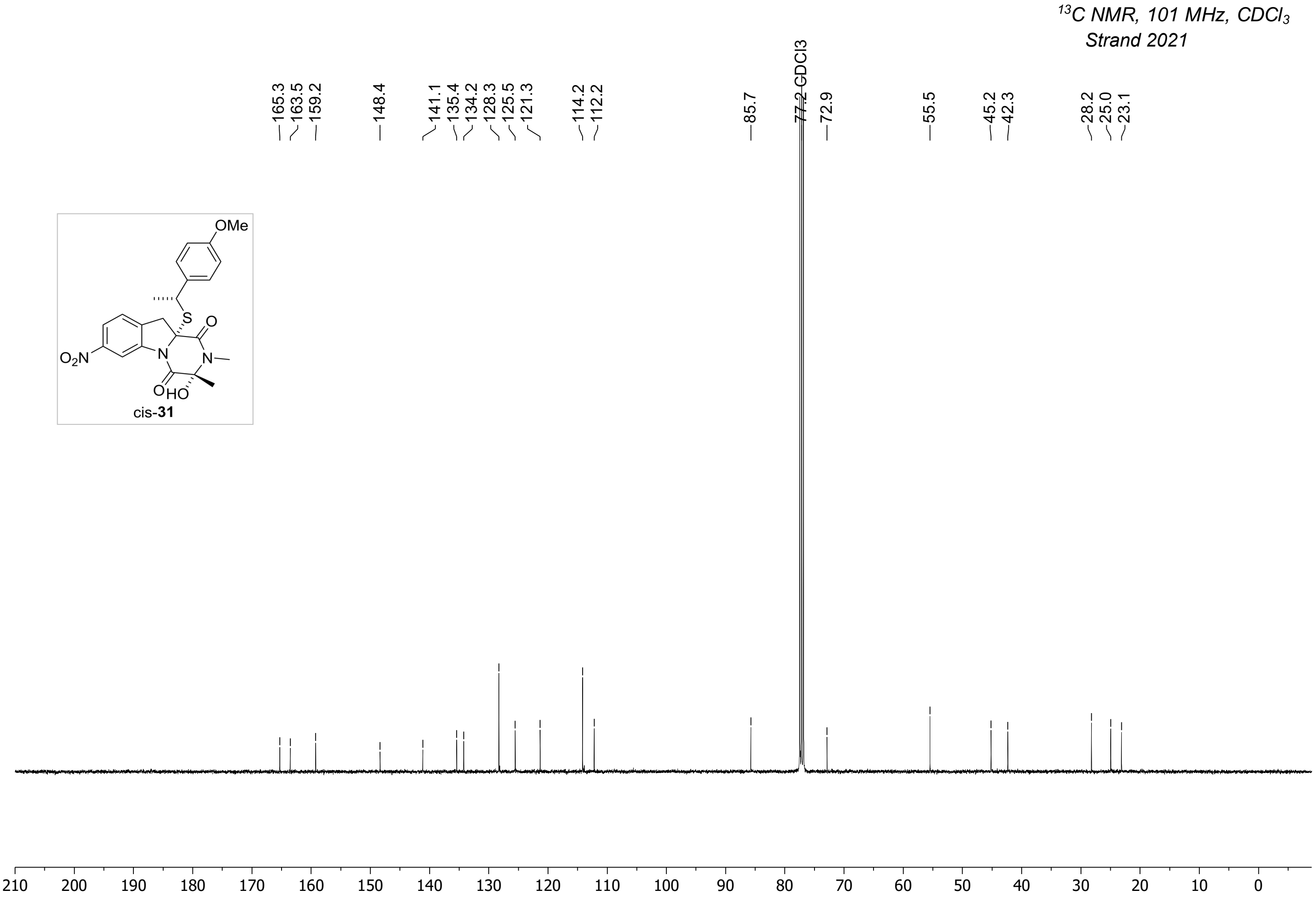

13C NMR, 101 MHz, CDCl3
Strand 2021
165.3
163.5
159.2
148.4
141.1
135.4
134.2
128.3
125.5
121.3
114.2
112.2
85.7
77.2 CDCl3
72.9
55.5
45.2
42.3
28.2
25.0
23.1
OMe
S
O
O2N
N
N
O
HO
cis-31

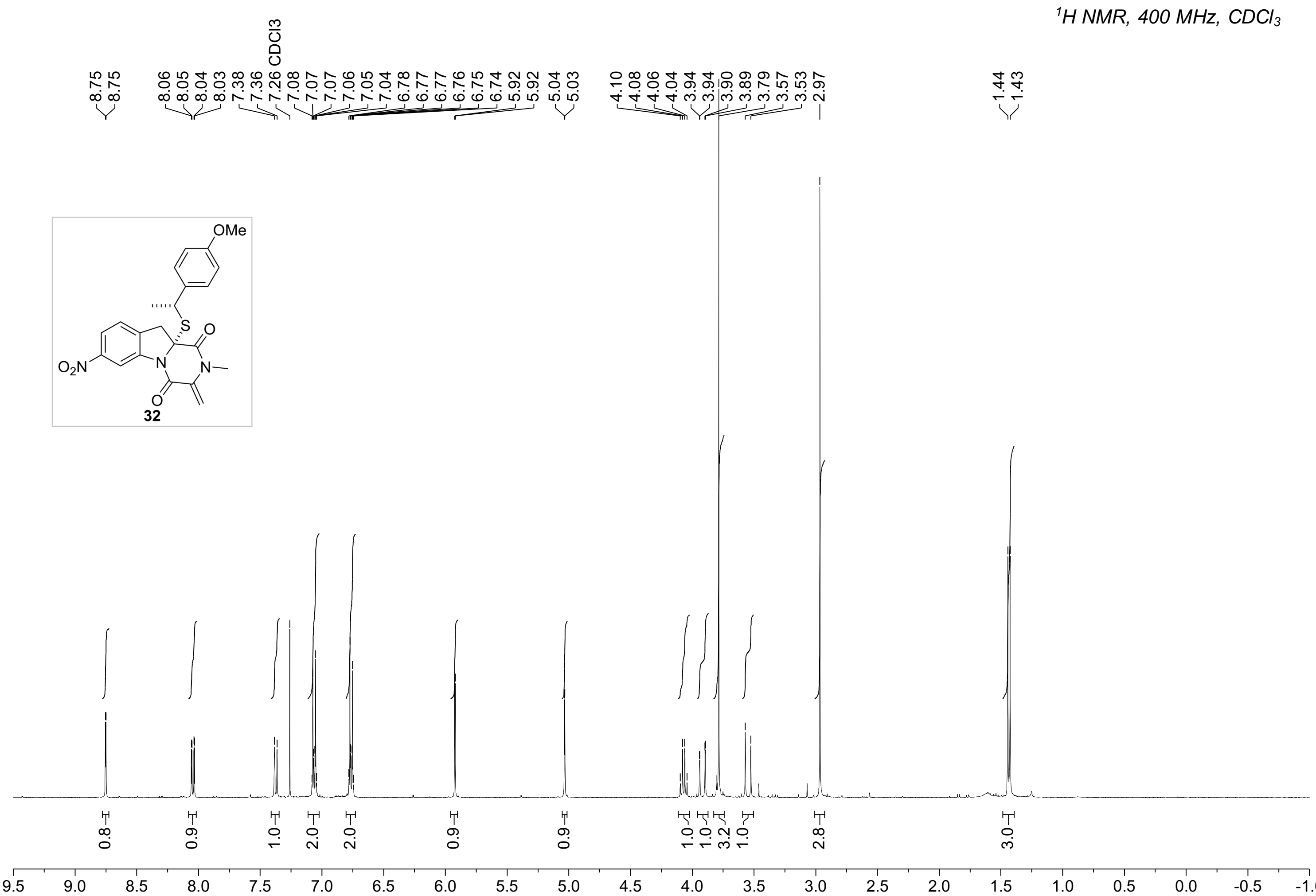

1H NMR, 400 MHz, CDCl3
8.75
8.75
8.06
8.05
8.04
8.03
7.38
7.36
7.26 CDCl3
7.08
7.07
7.07
7.06
7.05
7.04
6.78
6.77
6.77
6.76
6.75
6.74
5.92
5.92
5.04
5.03
4.10
4.08
4.06
4.04
3.94
3.94
3.90
3.89
3.79
3.57
3.53
2.97
1.44
1.43
0.8
0.9
1.0
2.0
2.0
0.9
0.9
1.0
1.0
3.2
1.0
2.8
3.0
OMe
S
O
O2N
N
N
O
32

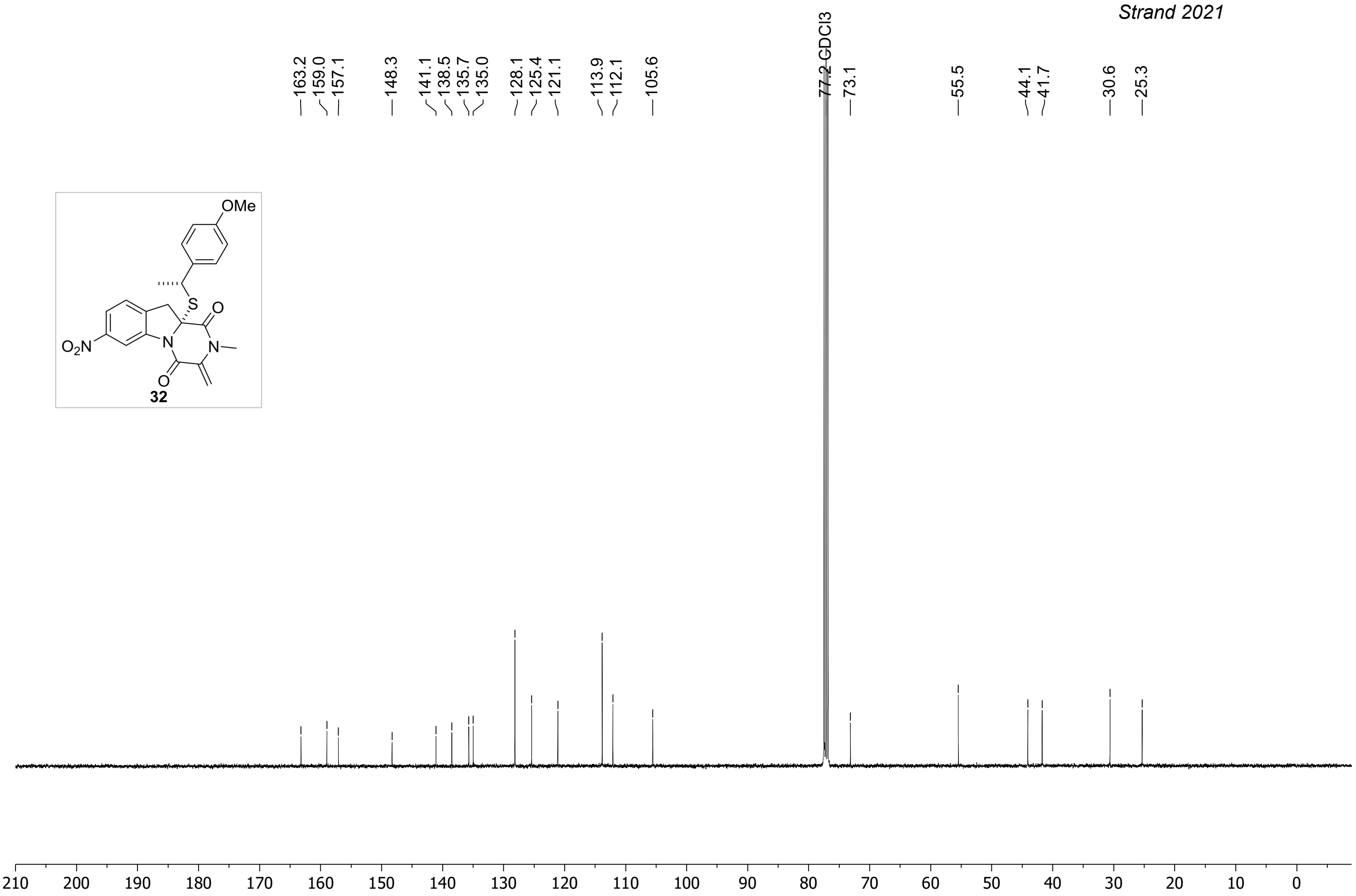
13C NMR, 101 MHz, CDCl3
Strand 2021
163.2
159.0
157.1
148.3
141.1
138.5
135.7
135.0
128.1
125.4
121.1
113.9
112.1
105.6
77.2 CDCl3
73.1
55.5
44.1
41.7
30.6
25.3
OMe
S
O
N
O2N
N
O
32
210 200 190 180 170 160 150 140 130 120 110 100 90 80 70 60 50 40 30 20 10 0

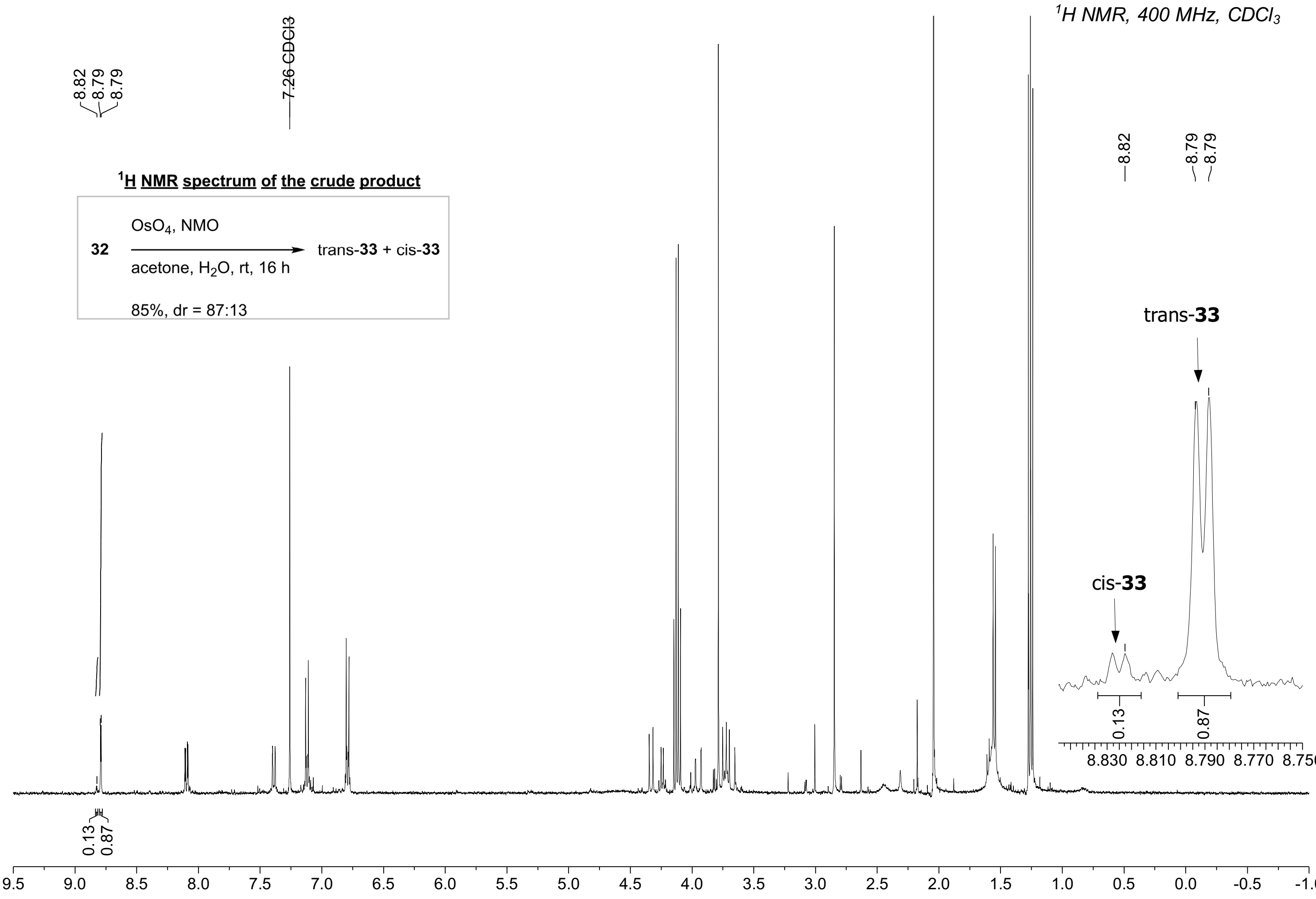
1H NMR, 400 MHz, CDCl3
8.82
8.79
8.79
7.26 CDCl3
1H NMR spectrum of the crude product
OsO4, NMO
32
trans-33 + cis-33
acetone, H2O, rt, 16 h
85%, dr = 87:13
trans-33
cis-33
0.13
0.87
8.830 8.810 8.790 8.770 8.750
9.5 9.0 8.5 8.0 7.5 7.0 6.5 6.0 5.5 5.0 4.5 4.0 3.5 3.0 2.5 2.0 1.5 1.0 0.5 0.0 -0.5 -1.0

^{1}H NMR, 400 MHz, $CDCl_3$
Strand 2021

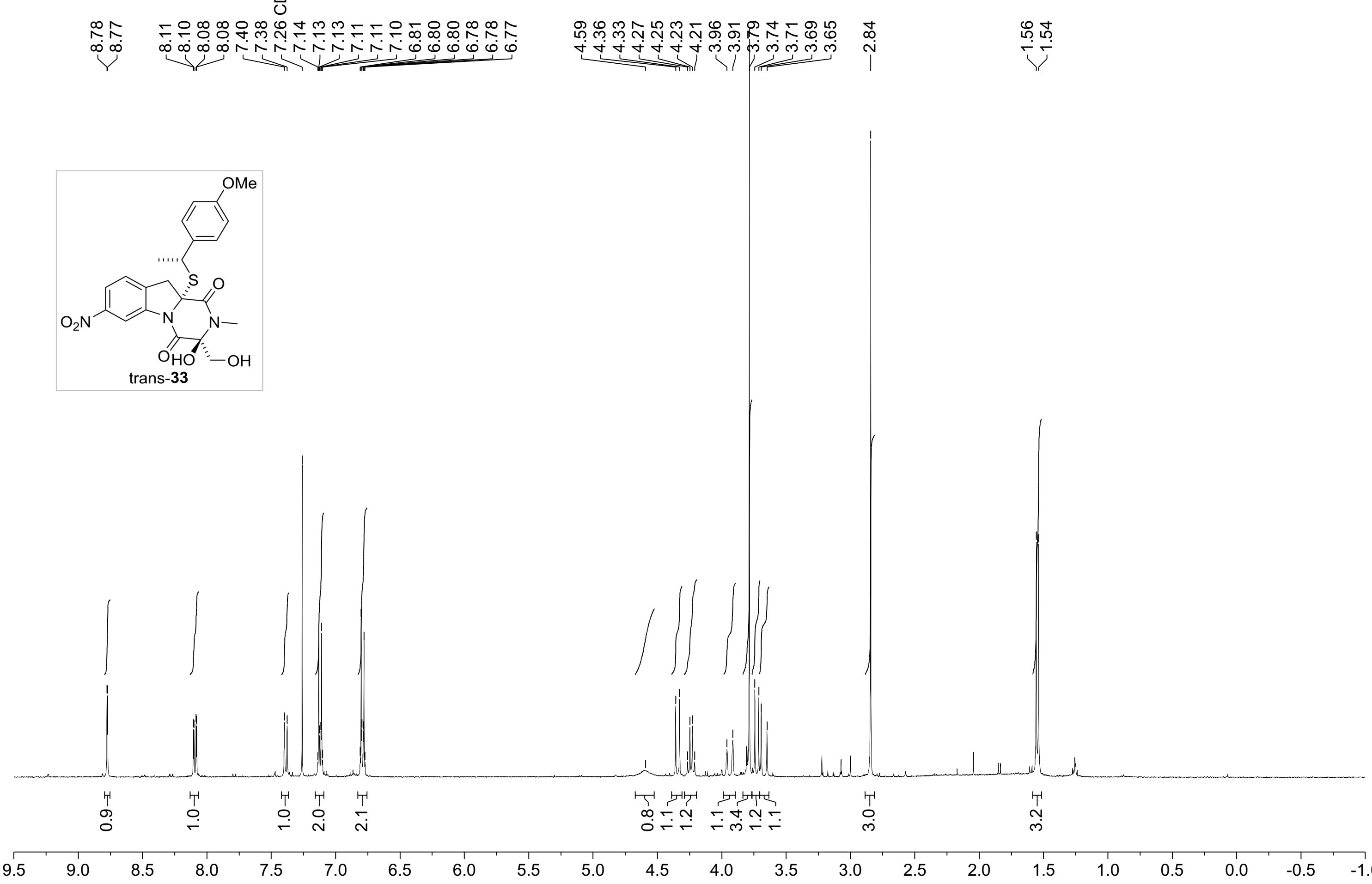

^{13}C NMR, 101 MHz, $CDCl_3$

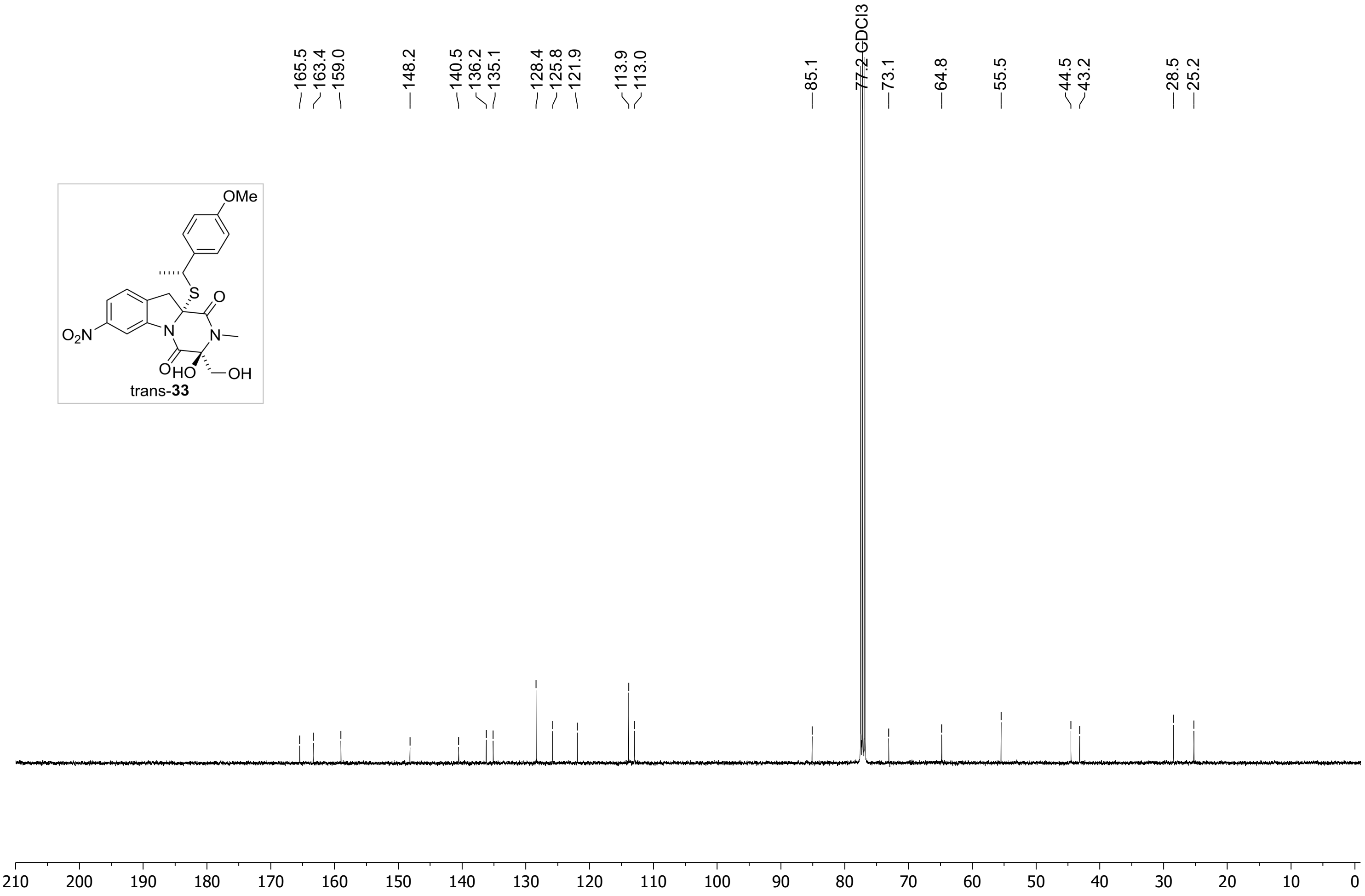

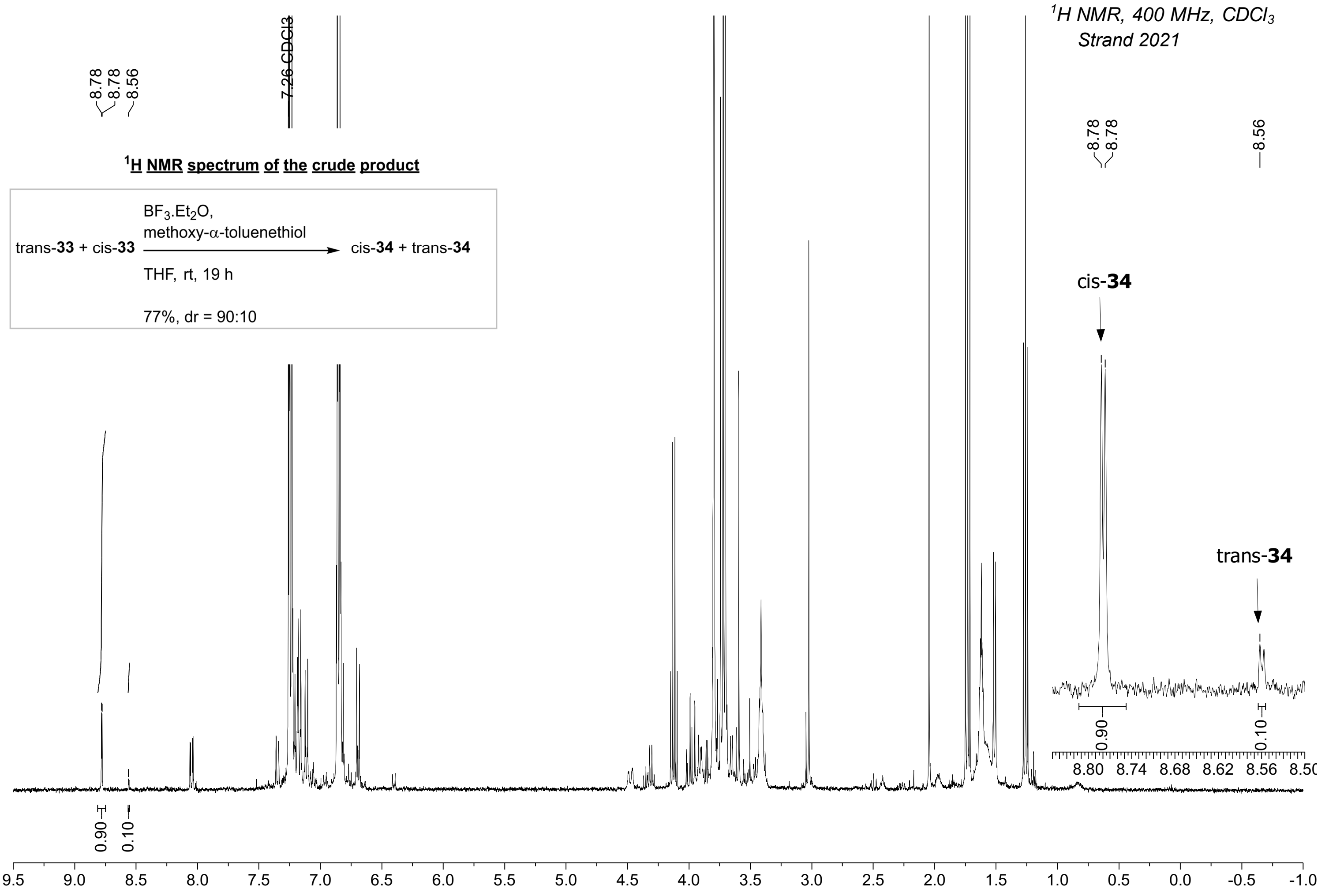

1H NMR, 400 MHz, CDCl3
Strand 2021
1H NMR spectrum of the crude product
BF3.Et2O,
methoxy-α-toluenethiol
trans-33 + cis-33
cis-34 + trans-34
THF, rt, 19 h
77%, dr = 90:10
cis-34
trans-34
7.26 CDCl3
8.78
8.78
8.56
0.90
0.10

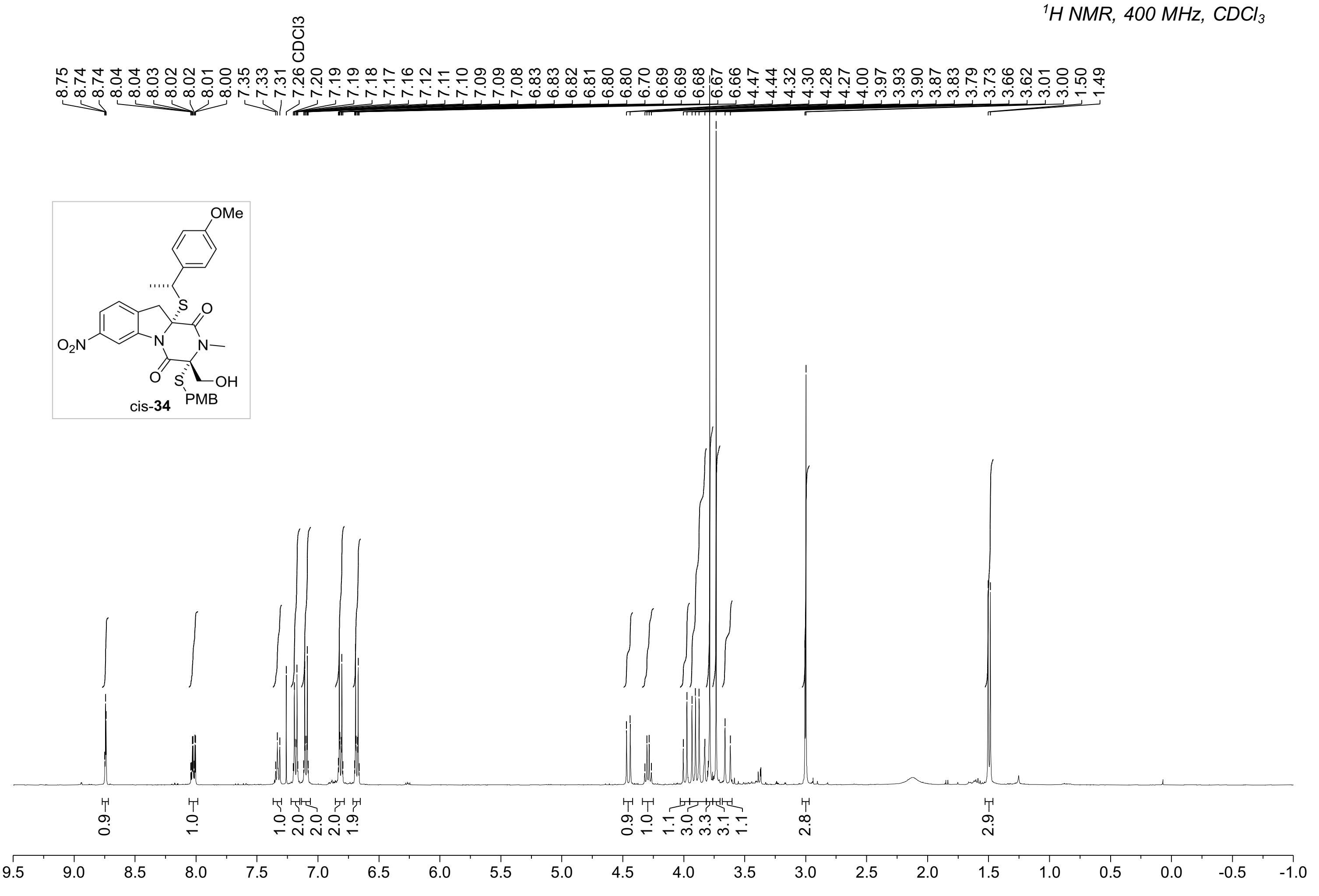

1H NMR, 400 MHz, CDCl3
7.26 CDCl3
OMe
S
O
O2N
N
N
O
S
OH
PMB
cis-34

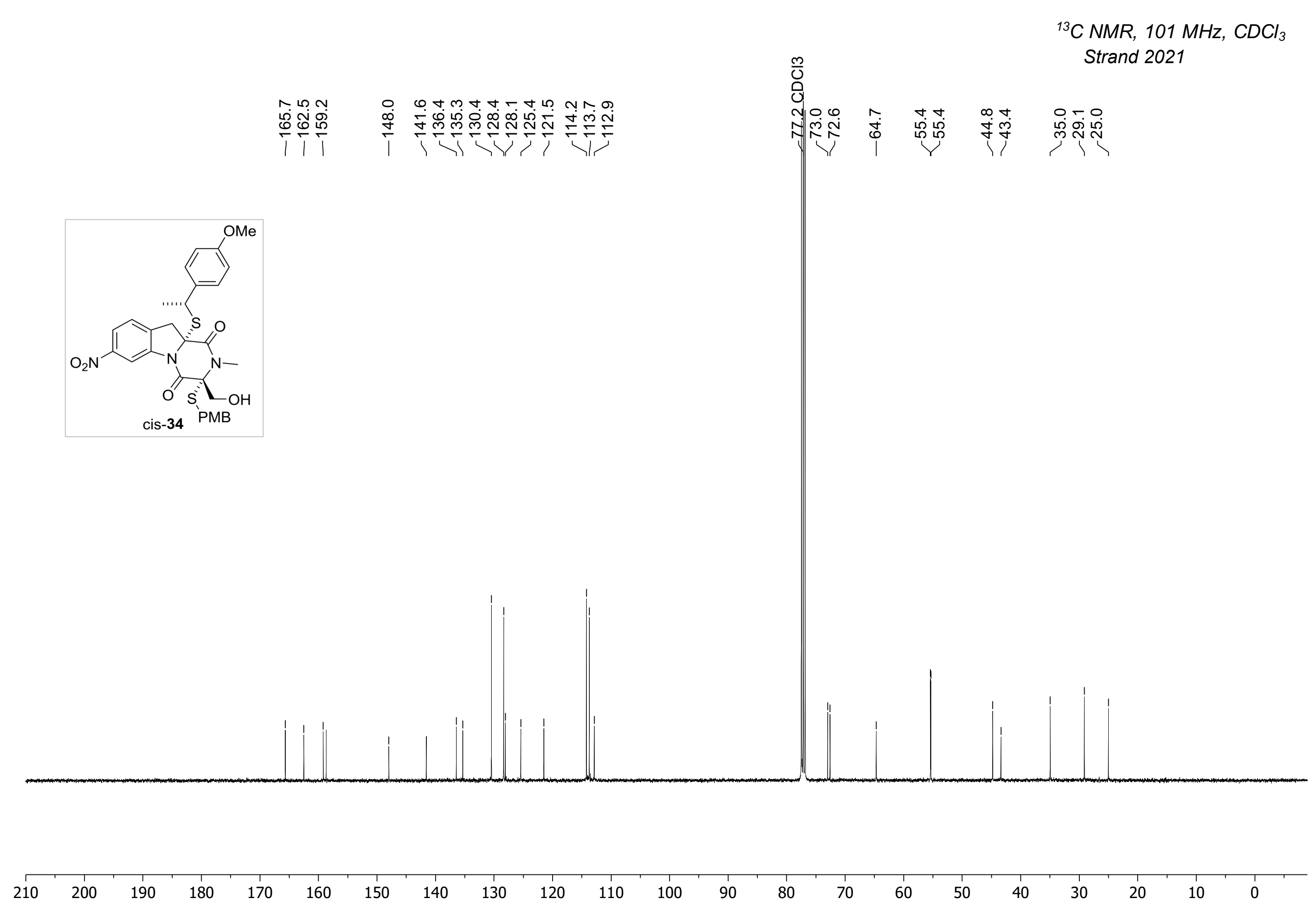
^{13}C NMR, 101 MHz, $CDCl_3$
Strand 2021
165.7
162.5
159.2
148.0
141.6
136.4
135.3
130.4
128.4
128.1
125.4
121.5
114.2
113.7
112.9
77.2 CDCl3
73.0
72.6
64.7
55.4
55.4
44.8
43.4
35.0
29.1
25.0
OMe
S
O
O_2N
N
N
O
S
OH
cis-34
PMB
210
200
190
180
170
160
150
140
130
120
110
100
90
80
70
60
50
40
30
20
10
0

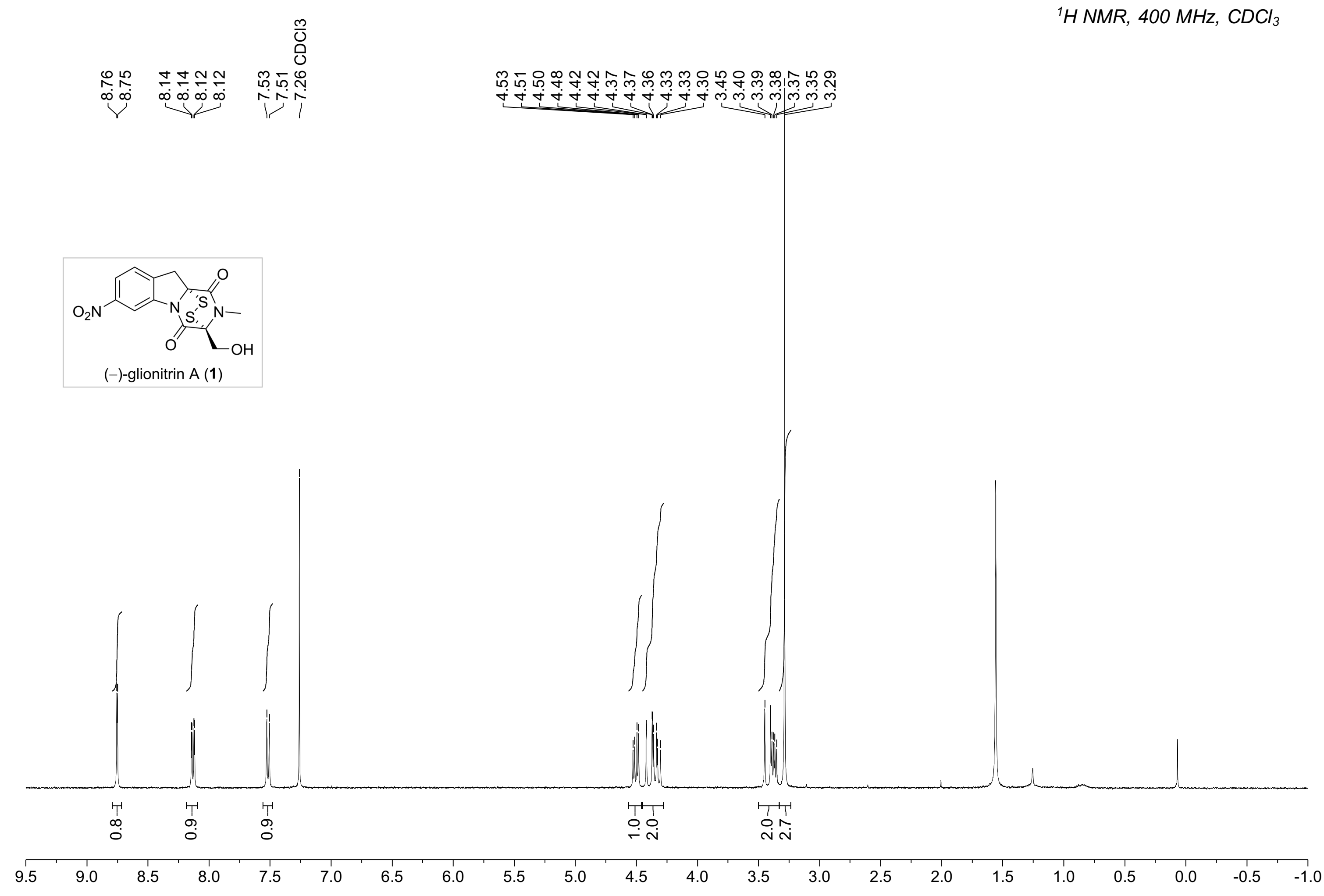
^{1}H NMR, 400 MHz, $CDCl_3$
8.76
8.75
8.14
8.14
8.12
8.12
7.53
7.51
7.26 CDCl3
4.53
4.51
4.50
4.48
4.42
4.42
4.37
4.37
4.36
4.33
4.33
4.30
3.45
3.40
3.39
3.38
3.37
3.35
3.29
O
O_2N
N
S
S
N
O
OH
(–)-glionitrin A (1)
0.8
0.9
0.9
1.0
2.0
2.0
2.7
9.5
9.0
8.5
8.0
7.5
7.0
6.5
6.0
5.5
5.0
4.5
4.0
3.5
3.0
2.5
2.0
1.5
1.0
0.5
0.0
-0.5
-1.0

^{13}C NMR, 101 MHz, $CDCl_3$
Strand 2021

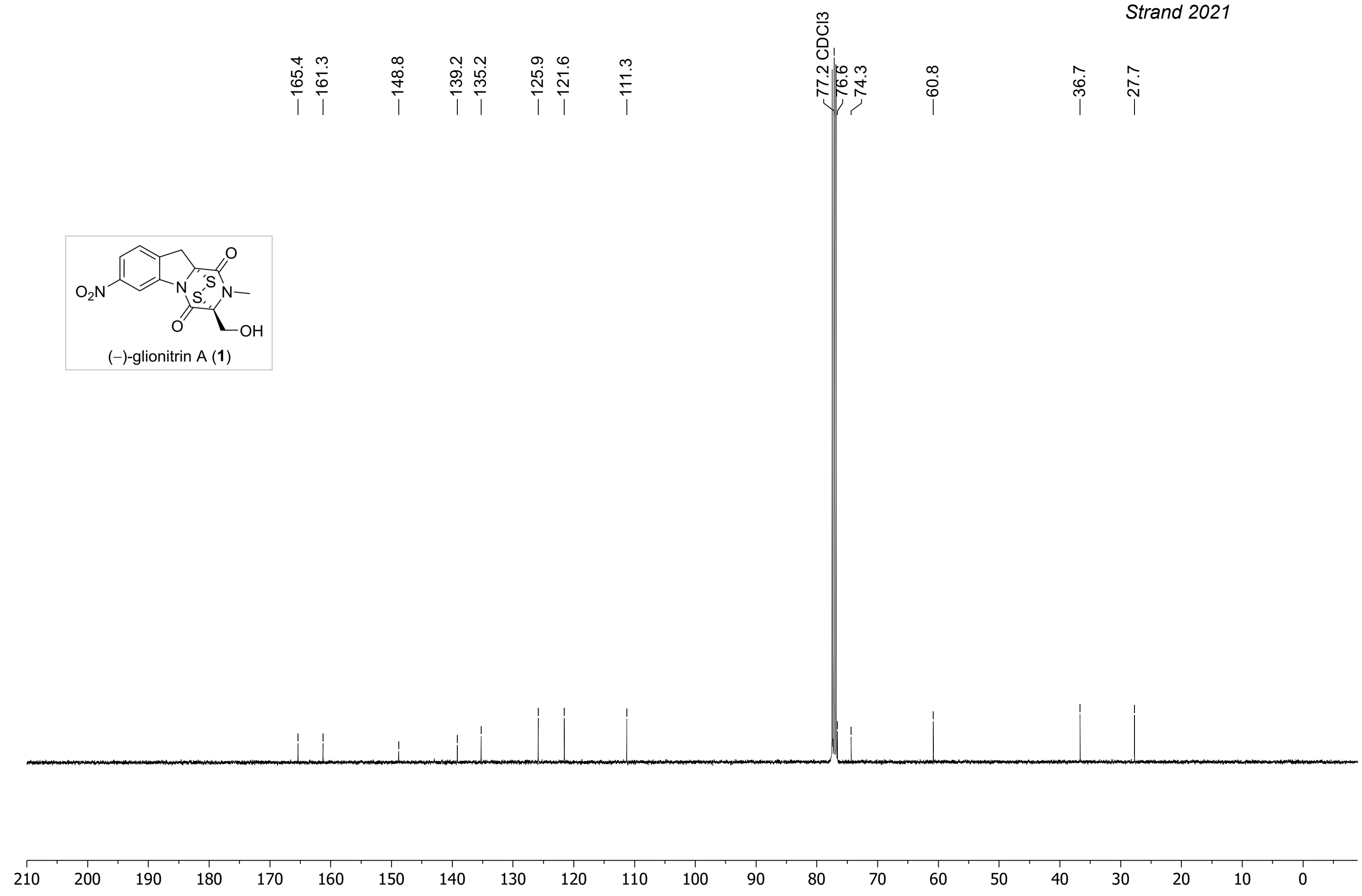

^1H NMR, 400 MHz, methanol-d_4

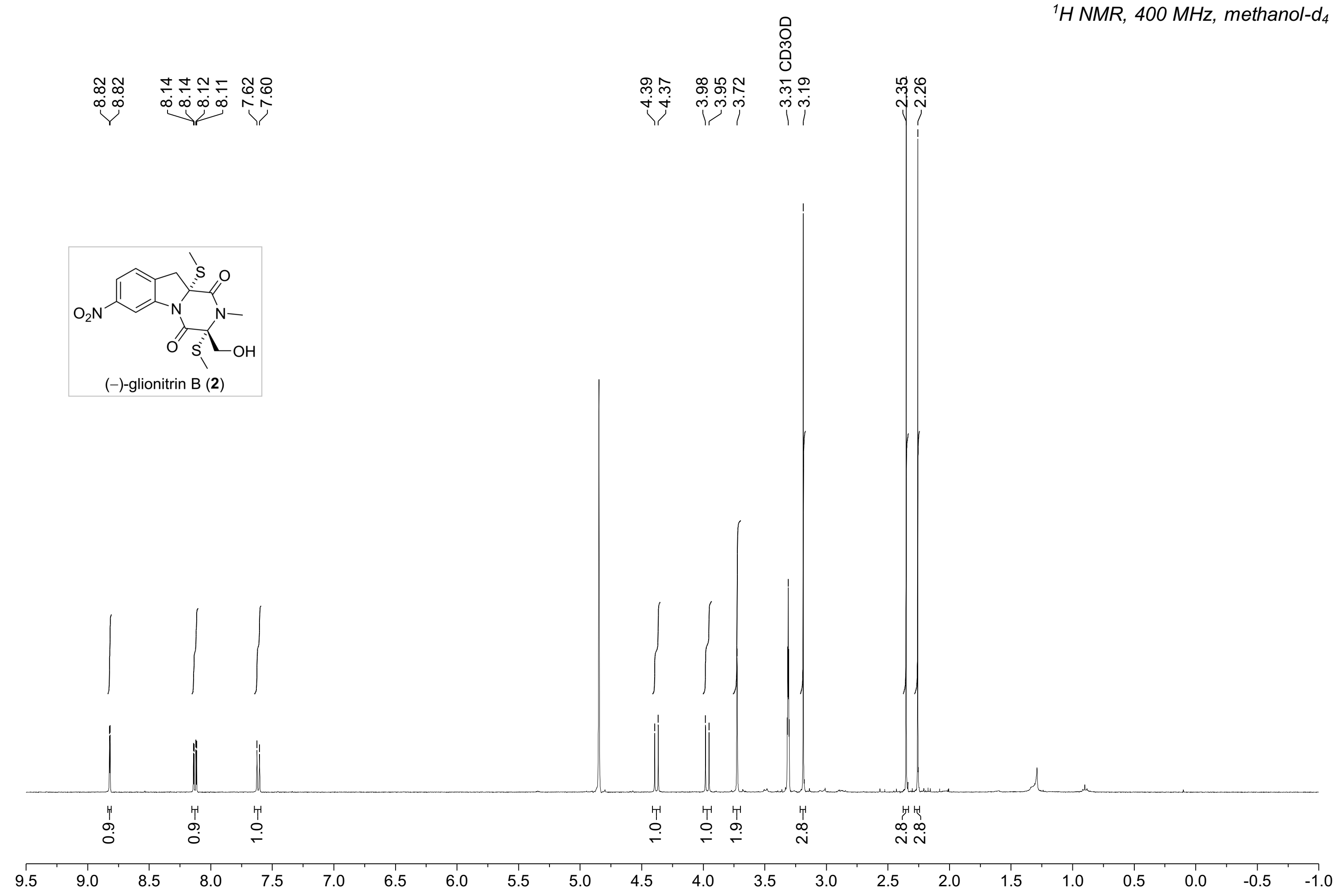

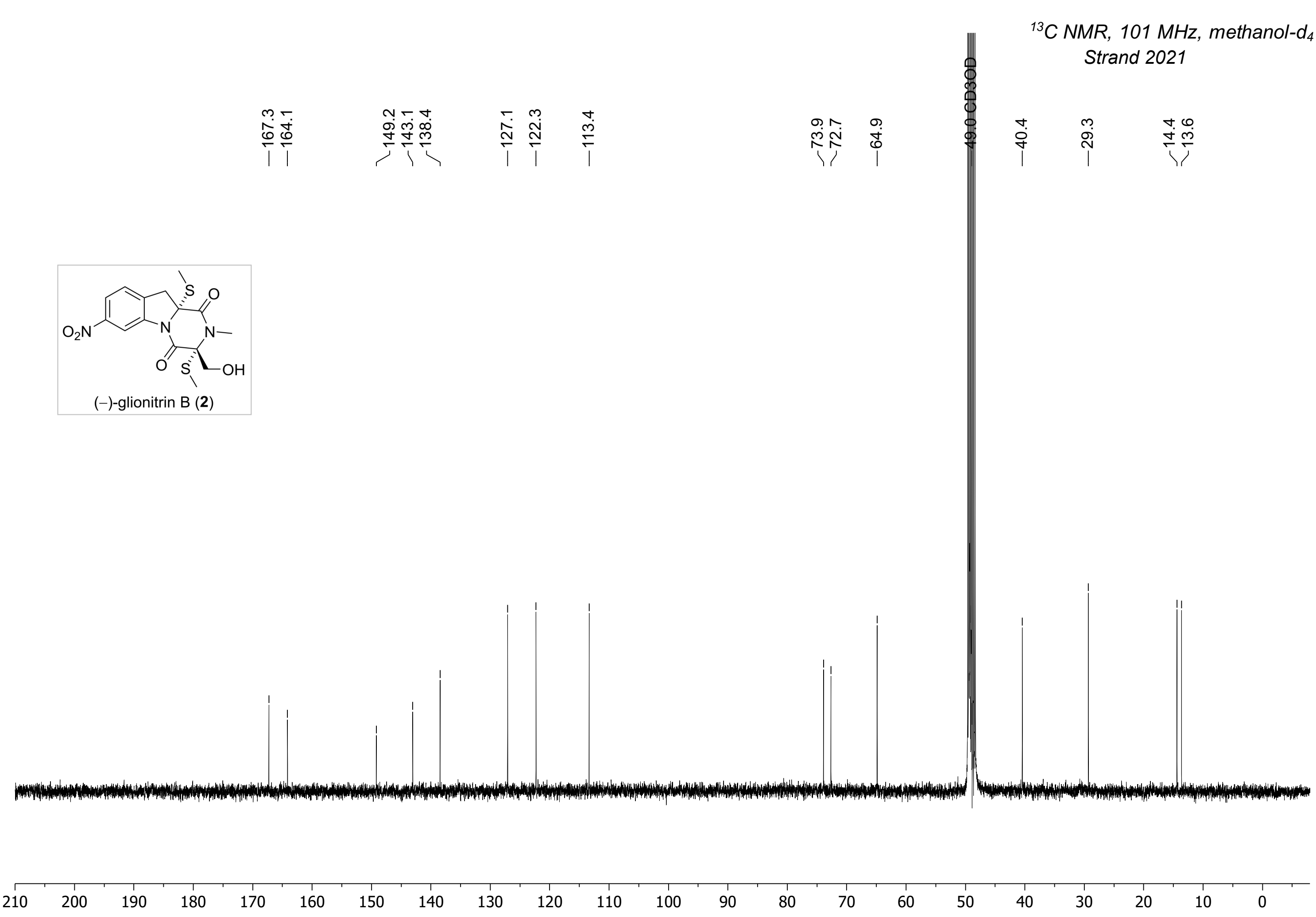
^{13}C NMR, 101 MHz, methanol-d_4
Strand 2021
167.3
164.1
149.2
143.1
138.4
127.1
122.3
113.4
73.9
72.7
64.9
49.0 CD3OD
40.4
29.3
14.4
13.6
(−)-glionitrin B (2)
210 200 190 180 170 160 150 140 130 120 110 100 90 80 70 60 50 40 30 20 10 0

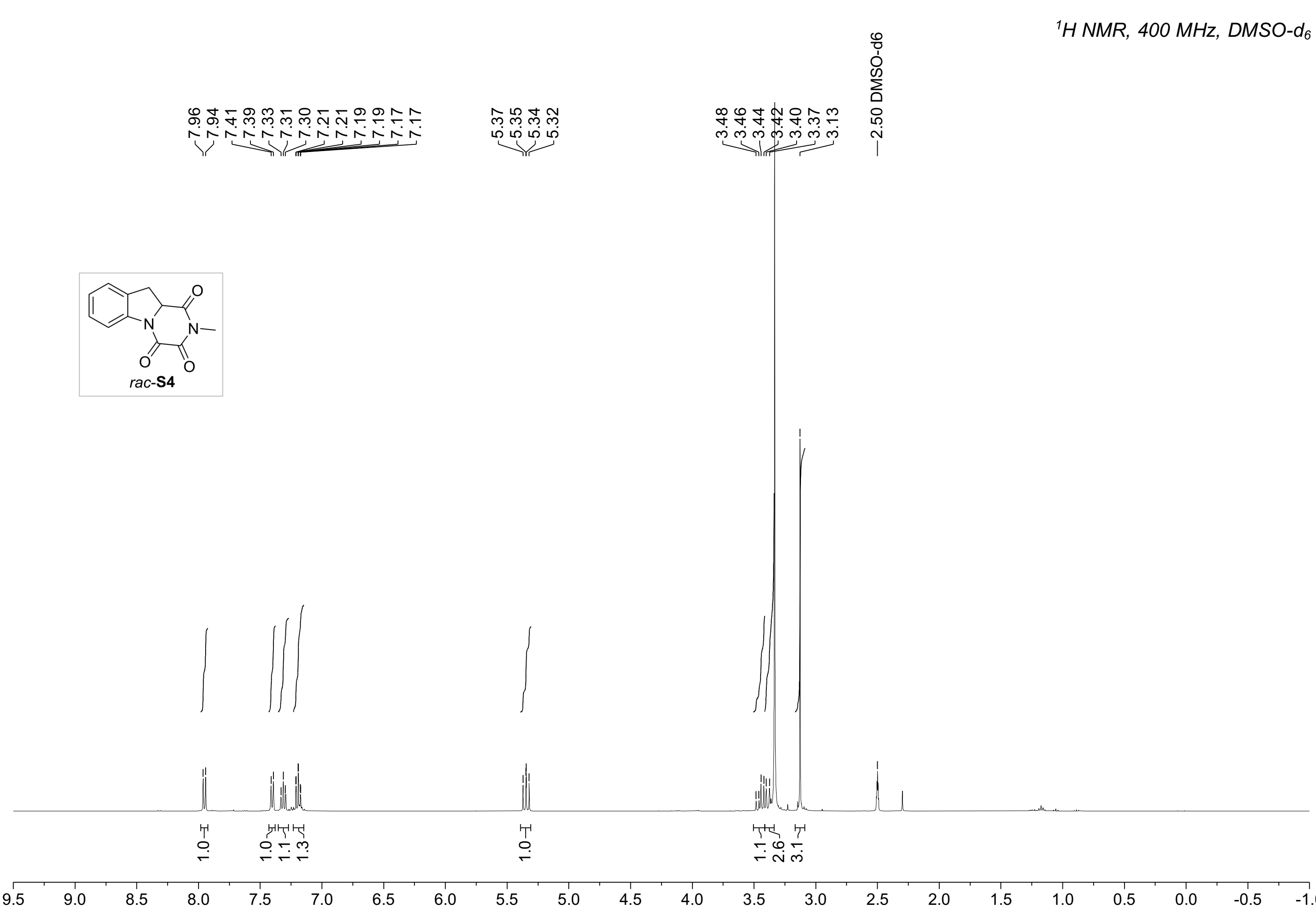
^{1}H NMR, 400 MHz, DMSO-d_6
7.96
7.94
7.41
7.39
7.33
7.31
7.30
7.21
7.21
7.19
7.19
7.17
7.17
5.37
5.35
5.34
5.32
3.48
3.46
3.44
3.42
3.40
3.37
3.13
2.50 DMSO-d6
rac-S4
1.0
1.0
1.1
1.3
1.0
1.1
2.6
3.1
9.5 9.0 8.5 8.0 7.5 7.0 6.5 6.0 5.5 5.0 4.5 4.0 3.5 3.0 2.5 2.0 1.5 1.0 0.5 0.0 -0.5 -1.0

^{13}C NMR, 101 MHz, DMSO-d_6
Strand 2021

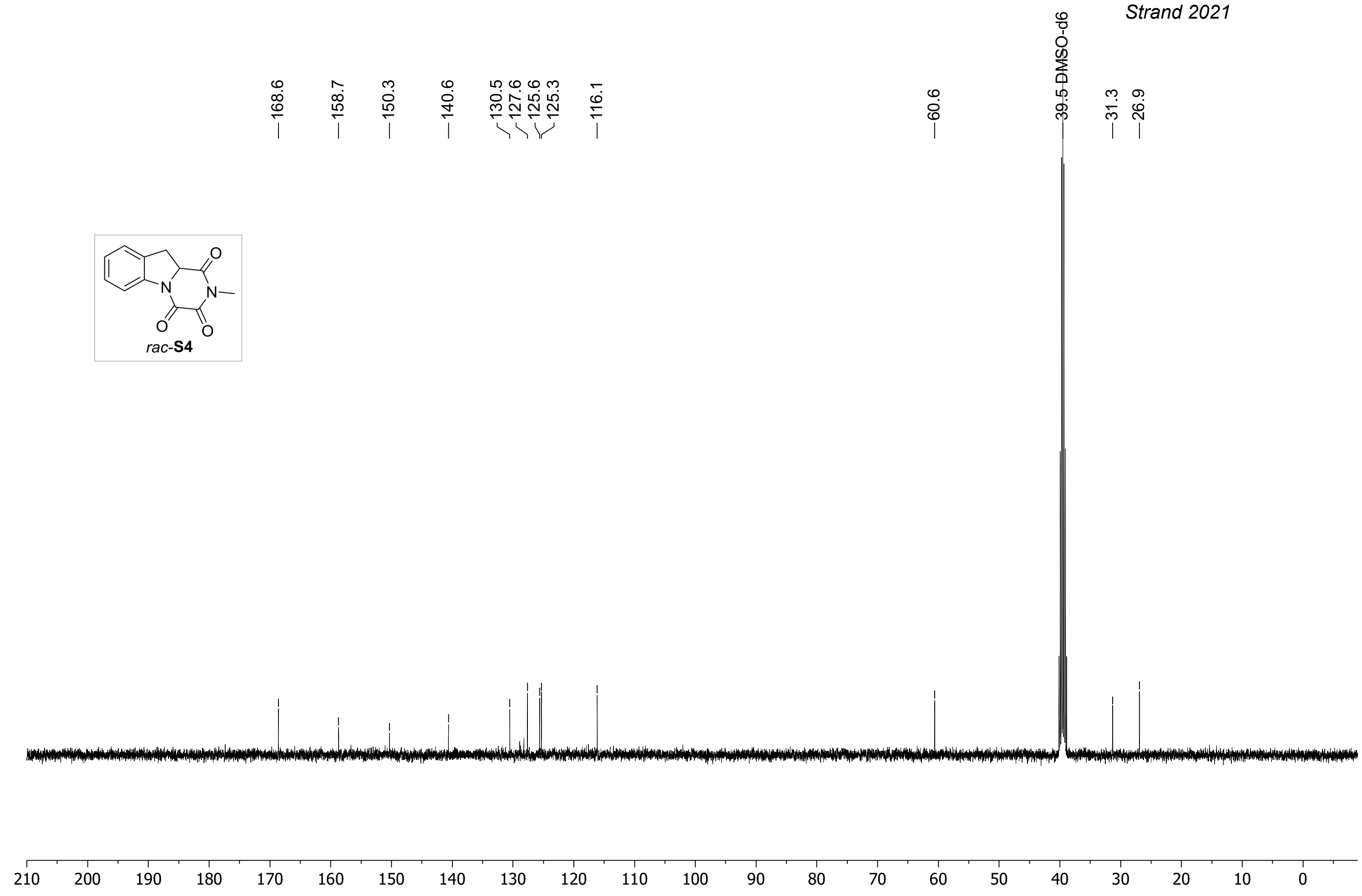

^{1}H NMR, 400 MHz, $CDCl_3$

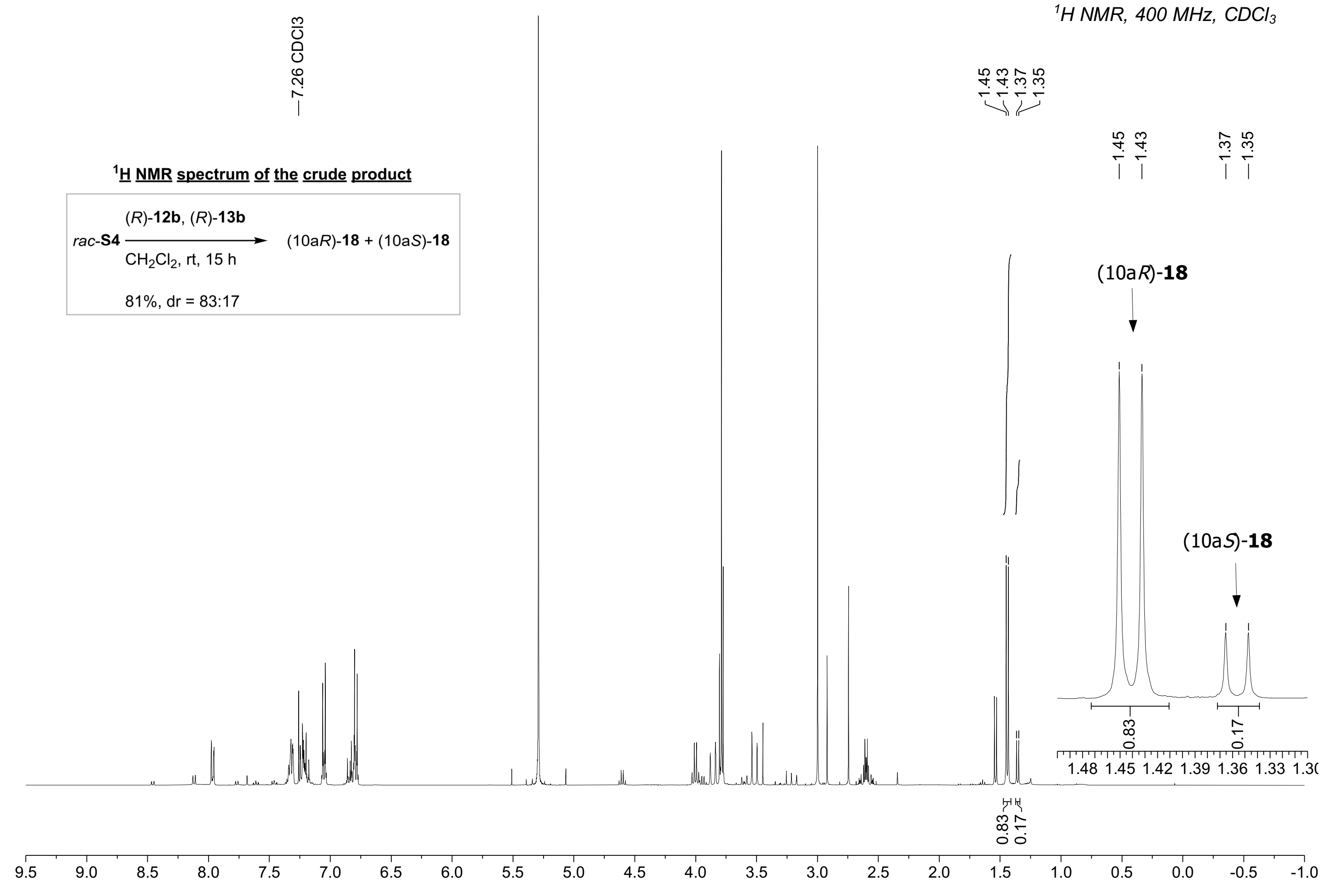

^1H NMR, 400 MHz, $CDCl_3$
Strand 2021

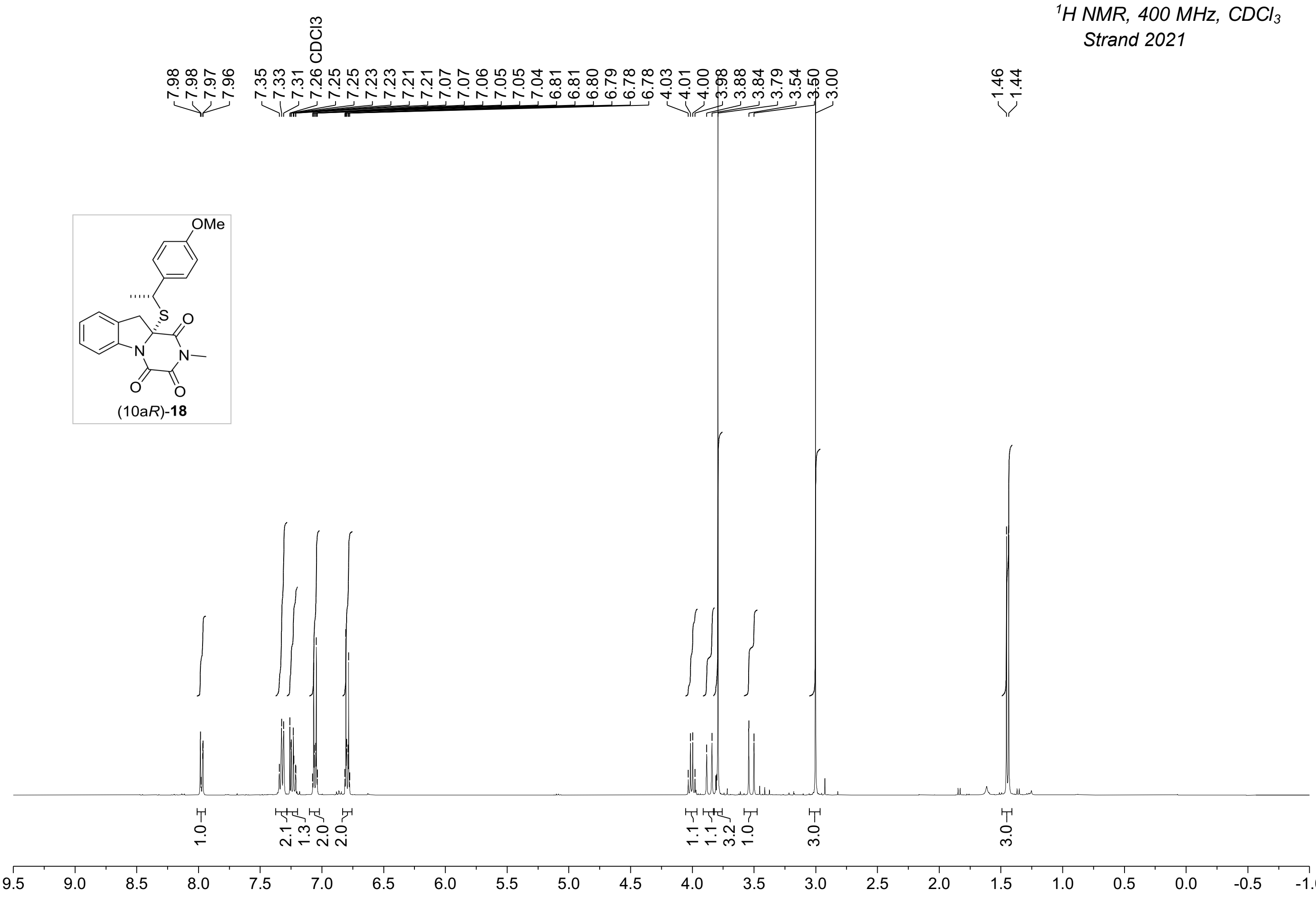

^{13}C NMR, 101 MHz, $CDCl_3$

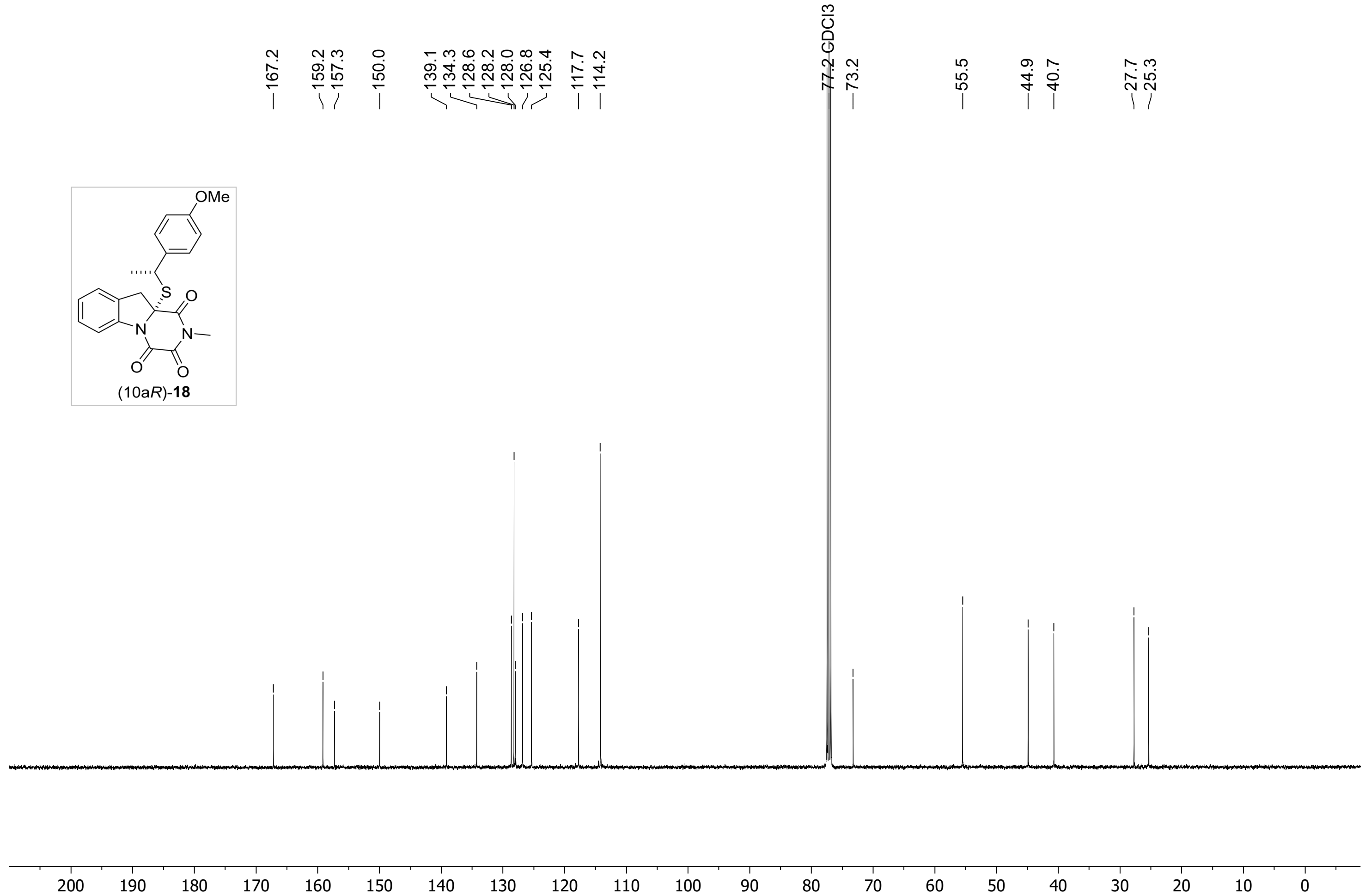

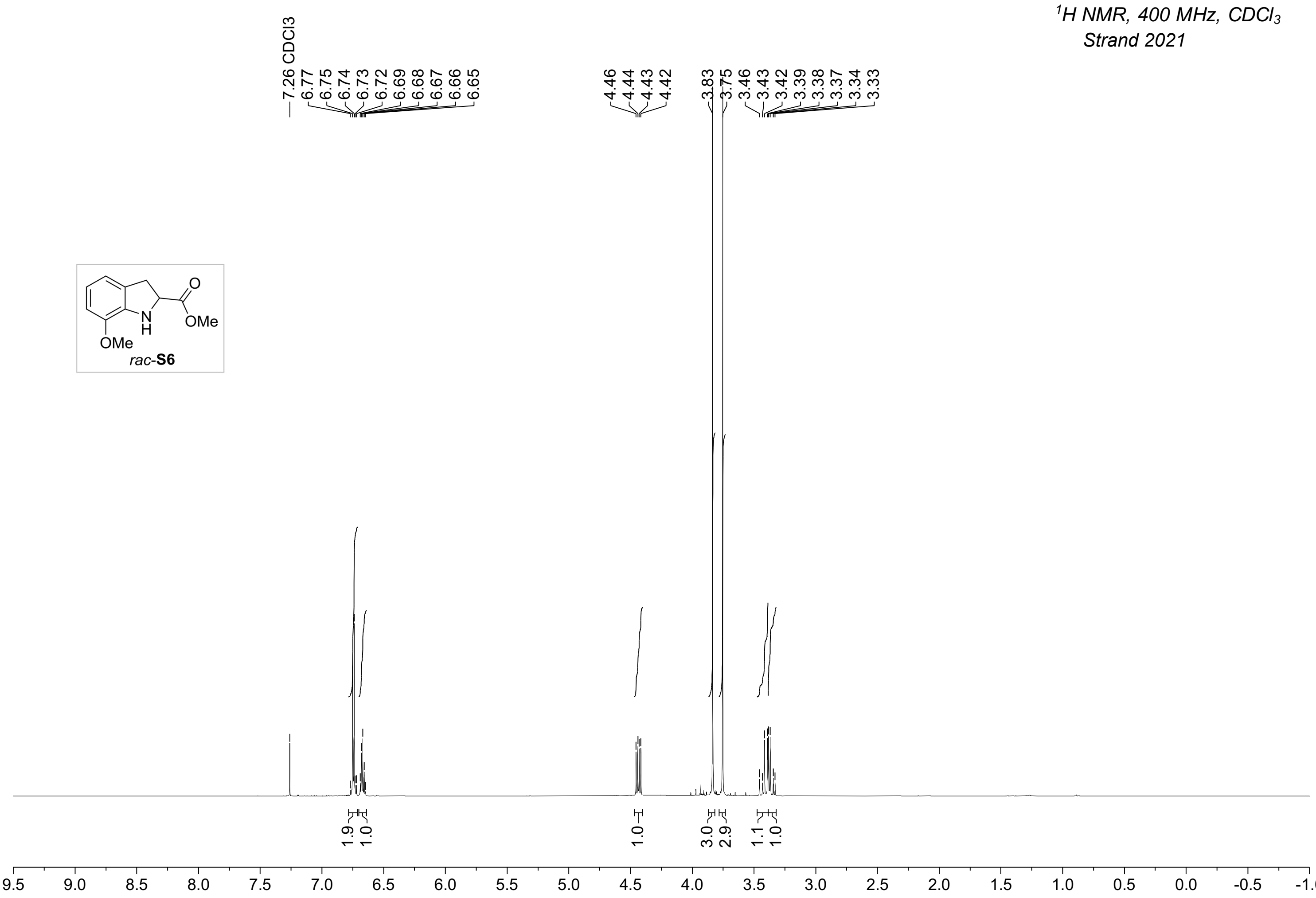

^{1}H NMR, 400 MHz, $CDCl_3$
Strand 2021
7.26 CDCl3
6.77
6.75
6.74
6.73
6.72
6.69
6.68
6.67
6.66
6.65
4.46
4.44
4.43
4.42
3.83
3.75
3.46
3.43
3.42
3.39
3.38
3.37
3.34
3.33
O
N
H
OMe
OMe
rac-S6
1.9
1.0
1.0
3.0
2.9
1.1
1.0
9.5 9.0 8.5 8.0 7.5 7.0 6.5 6.0 5.5 5.0 4.5 4.0 3.5 3.0 2.5 2.0 1.5 1.0 0.5 0.0 -0.5 -1.0

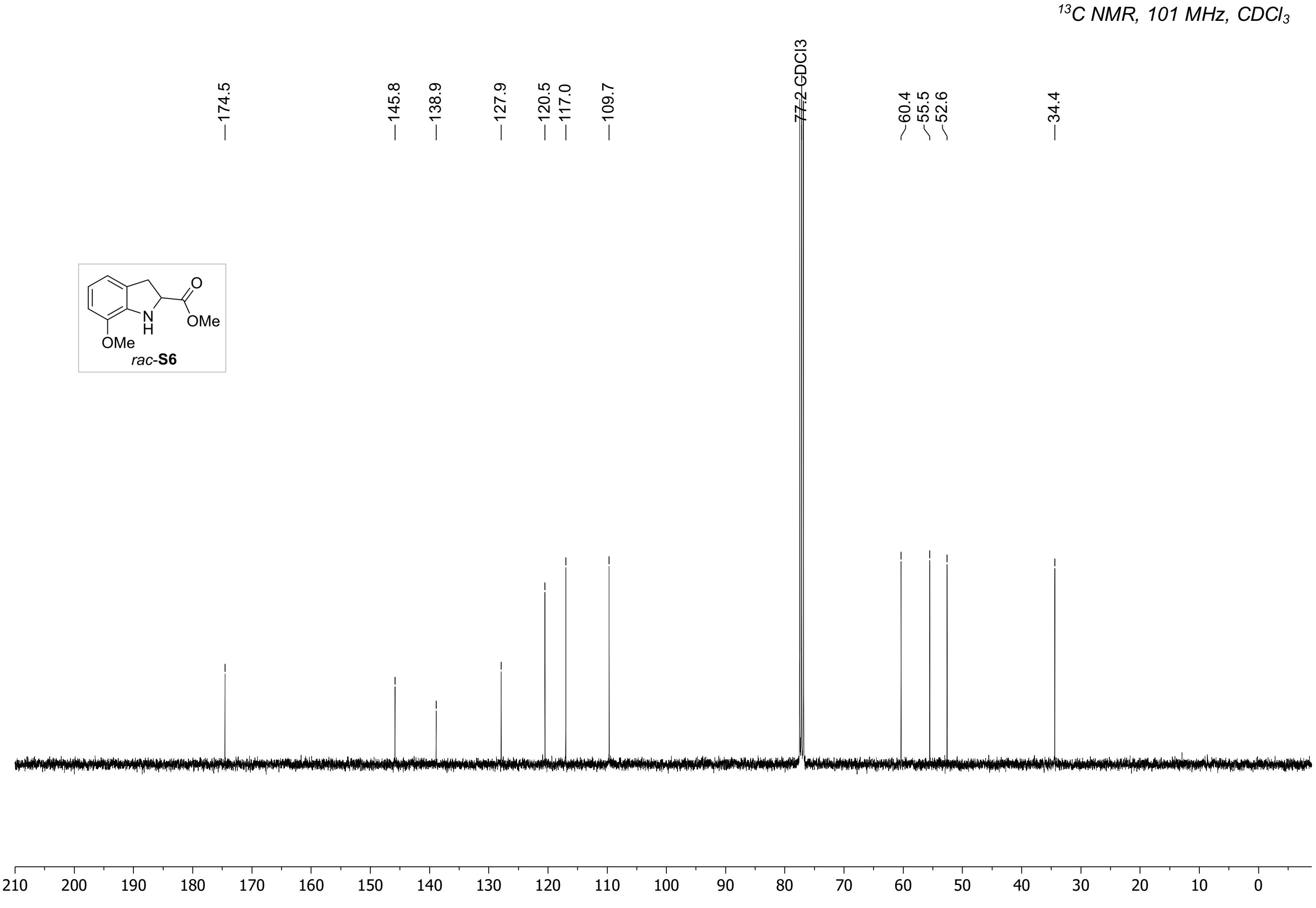

^{13}C NMR, 101 MHz, $CDCl_3$
174.5
145.8
138.9
127.9
120.5
117.0
109.7
77.2 CDCl3
60.4
55.5
52.6
34.4
O
N
H
OMe
OMe
rac-S6
210 200 190 180 170 160 150 140 130 120 110 100 90 80 70 60 50 40 30 20 10 0

^{1}H NMR, 400 MHz, $CDCl_3$
Strand 2021

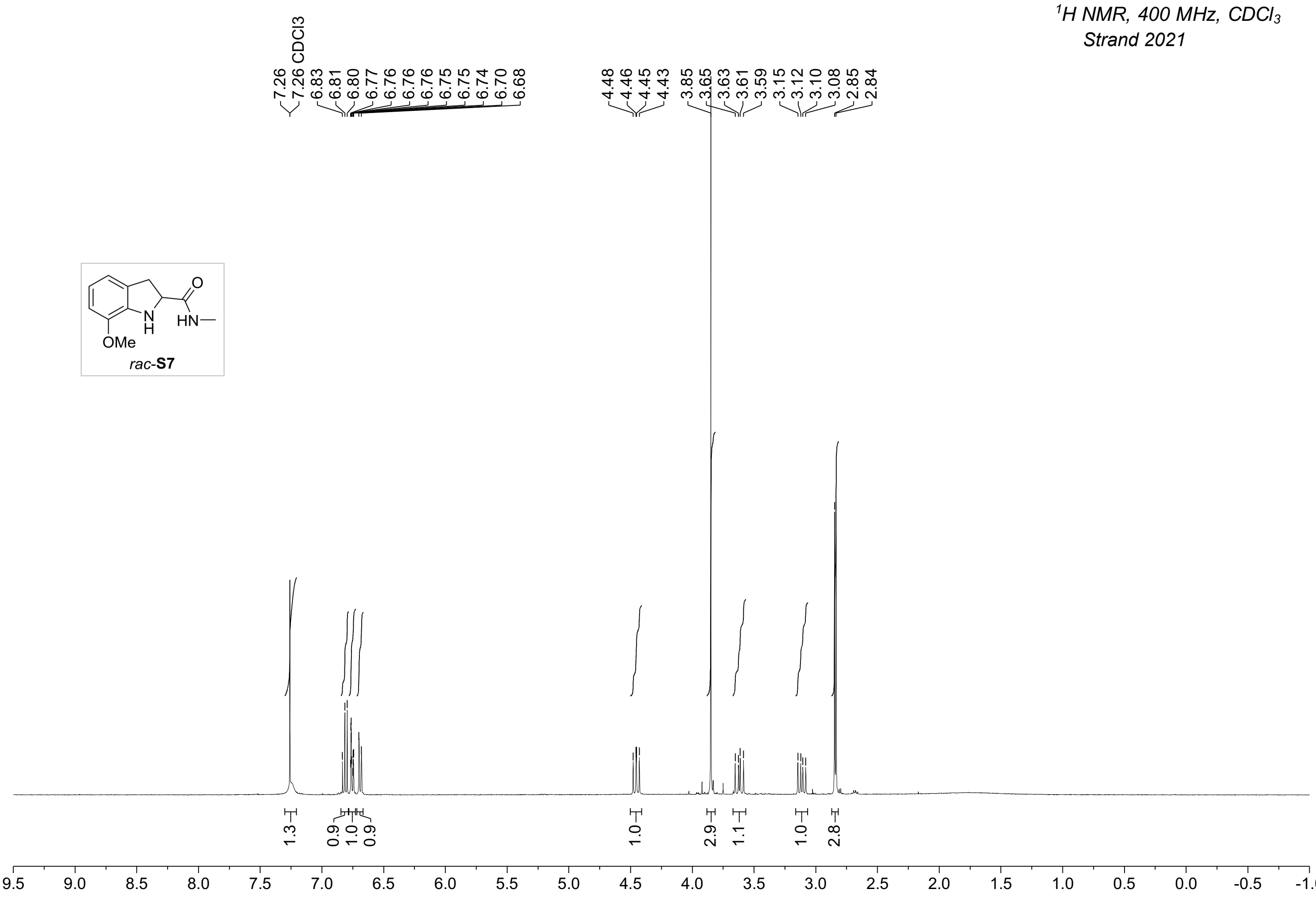

^{13}C NMR, 101 MHz, $CDCl_3$

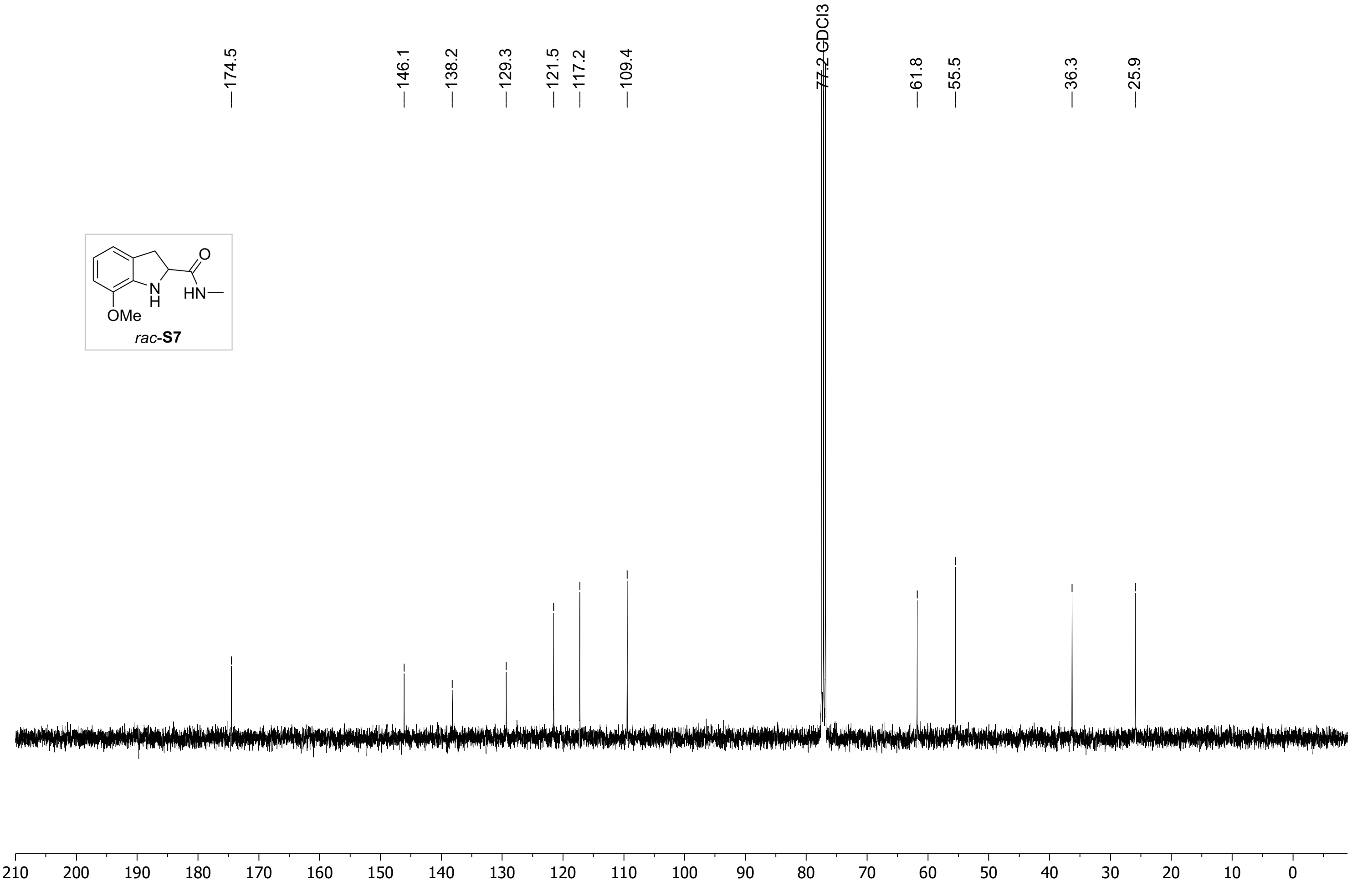

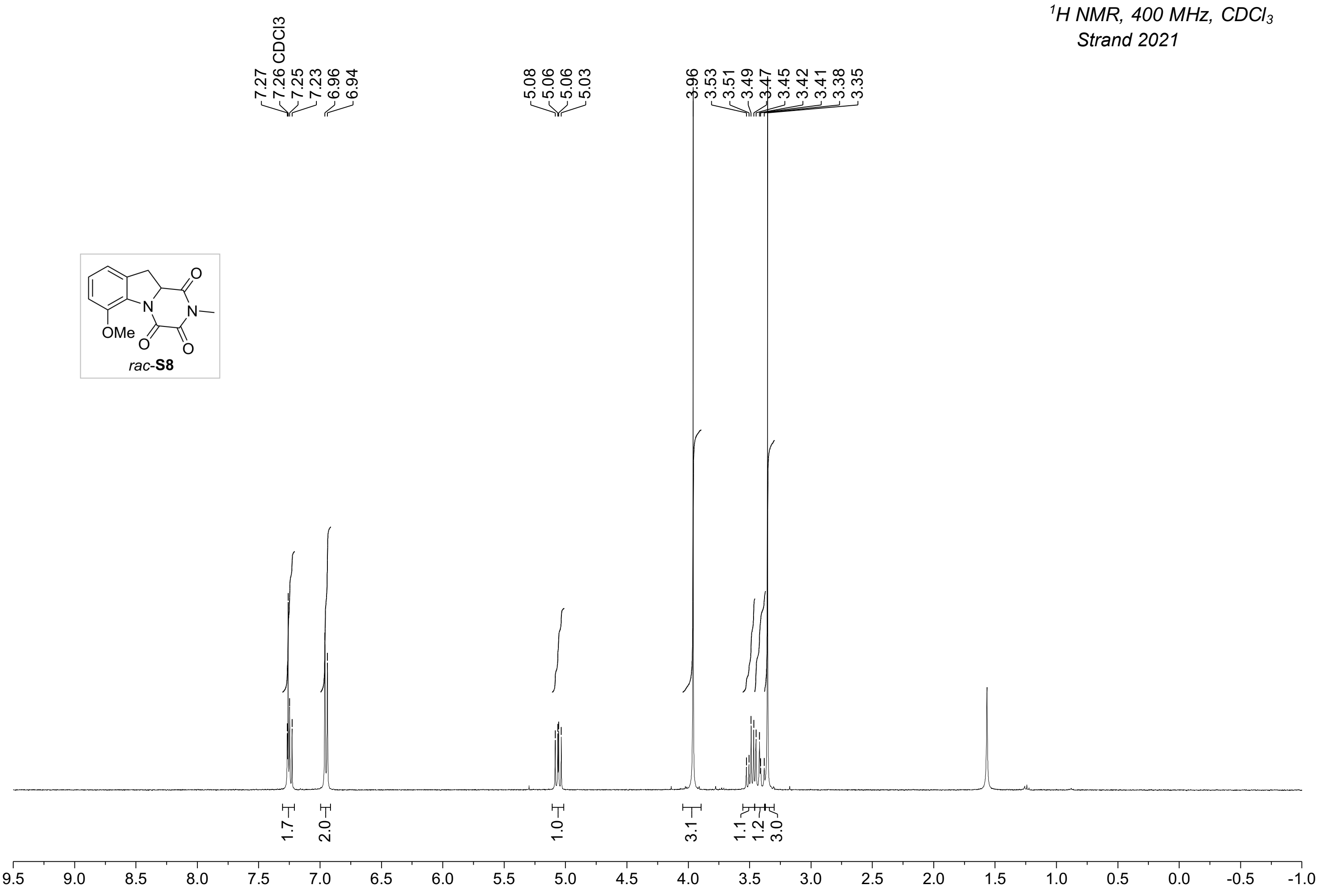
1H NMR, 400 MHz, CDCl3
Strand 2021
7.27
7.26 CDCl3
7.25
7.23
6.96
6.94
5.08
5.06
5.06
5.03
3.96
3.53
3.51
3.49
3.47
3.45
3.42
3.41
3.38
3.35
OMe
N
O
rac-S8
1.7
2.0
1.0
3.1
1.1
1.2
3.0
9.5 9.0 8.5 8.0 7.5 7.0 6.5 6.0 5.5 5.0 4.5 4.0 3.5 3.0 2.5 2.0 1.5 1.0 0.5 0.0 -0.5 -1.0

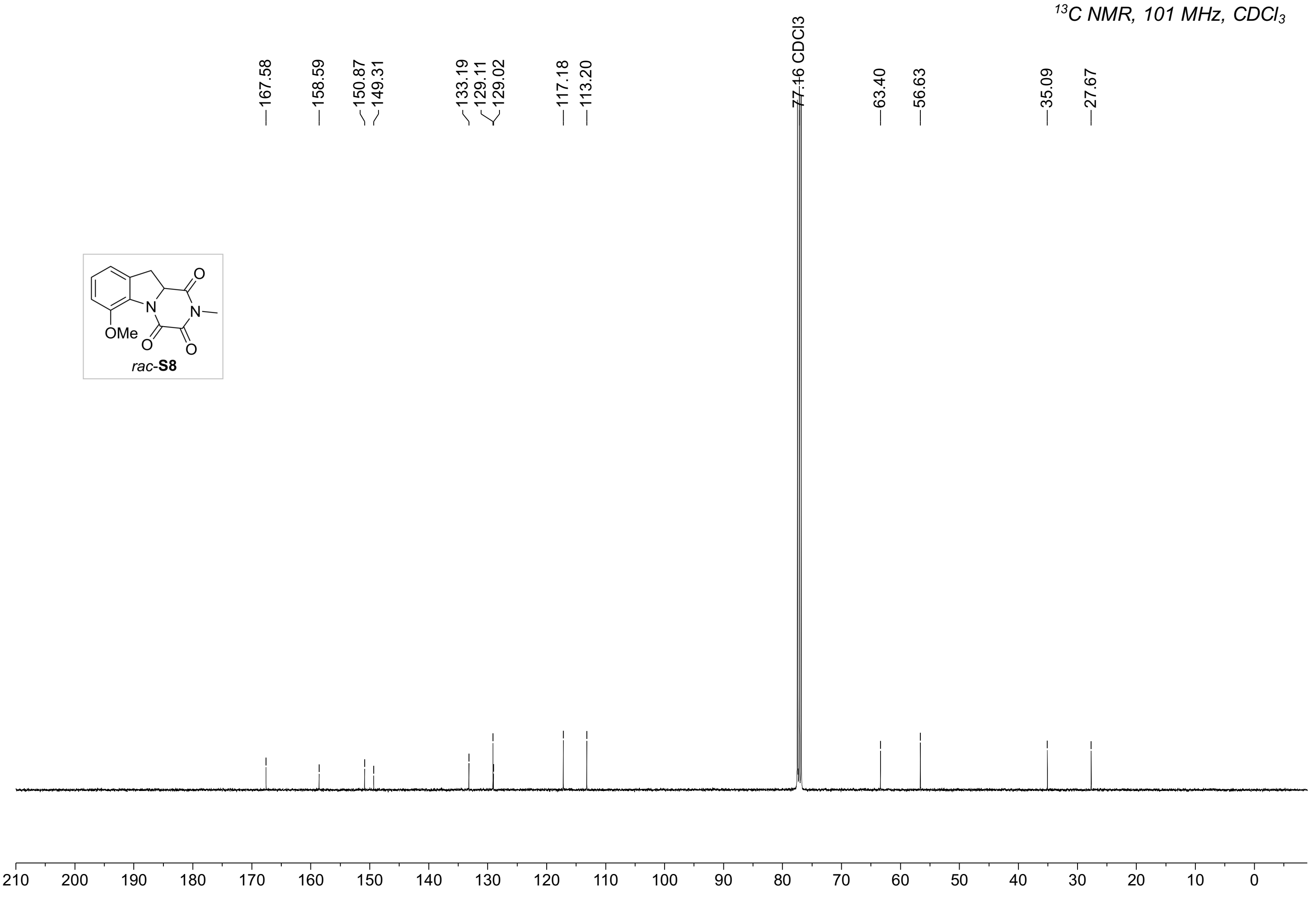
13C NMR, 101 MHz, CDCl3
167.58
158.59
150.87
149.31
133.19
129.11
129.02
117.18
113.20
77.16 CDCl3
63.40
56.63
35.09
27.67
OMe
N
O
rac-S8
210 200 190 180 170 160 150 140 130 120 110 100 90 80 70 60 50 40 30 20 10 0

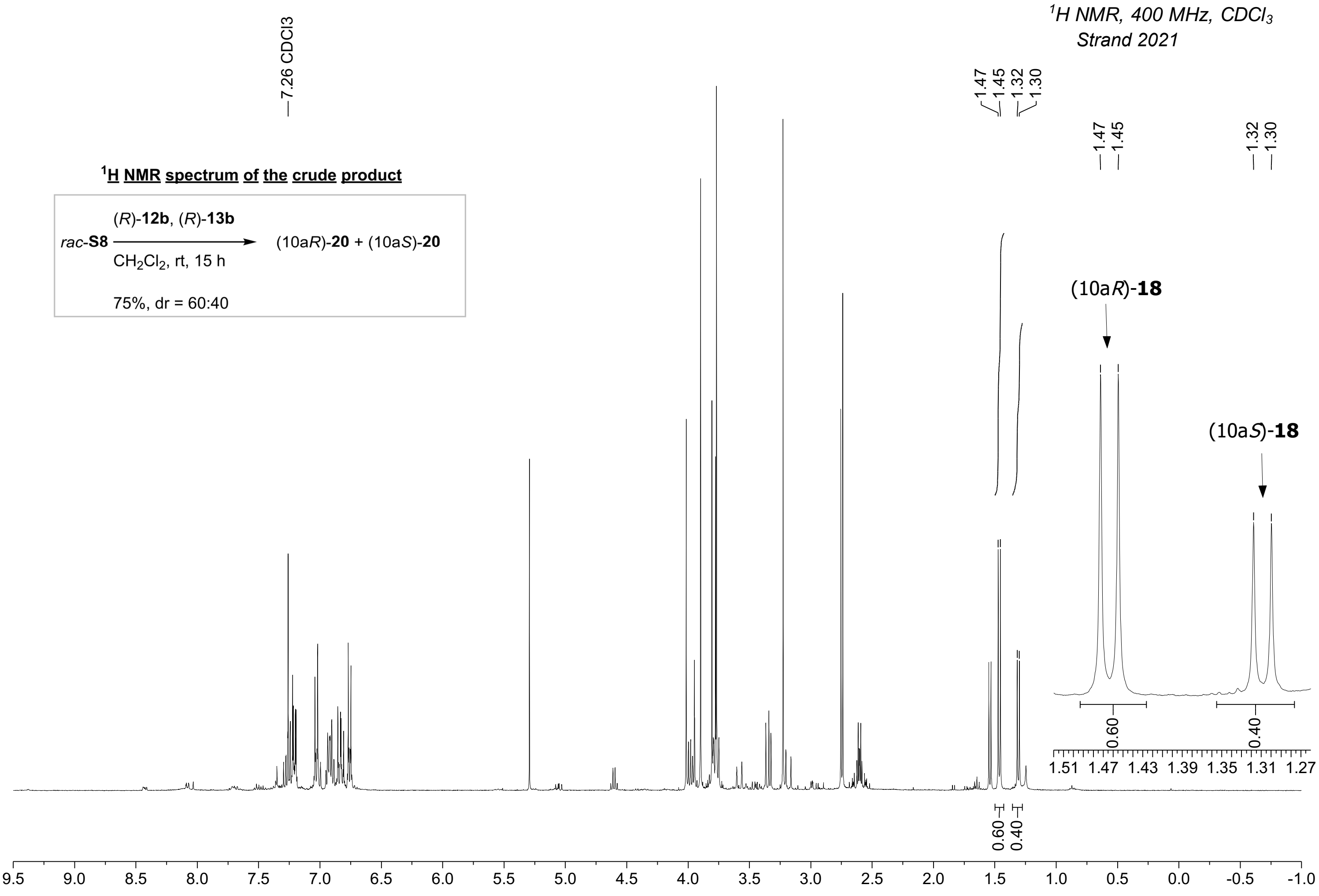
1H NMR, 400 MHz, CDCl3
Strand 2021
1H NMR spectrum of the crude product
(R)-12b, (R)-13b
rac-S8
CH2Cl2, rt, 15 h
(10aR)-20 + (10aS)-20
75%, dr = 60:40
7.26 CDCl3
1.47
1.45
1.32
1.30
(10aR)-18
(10aS)-18
0.60
0.40

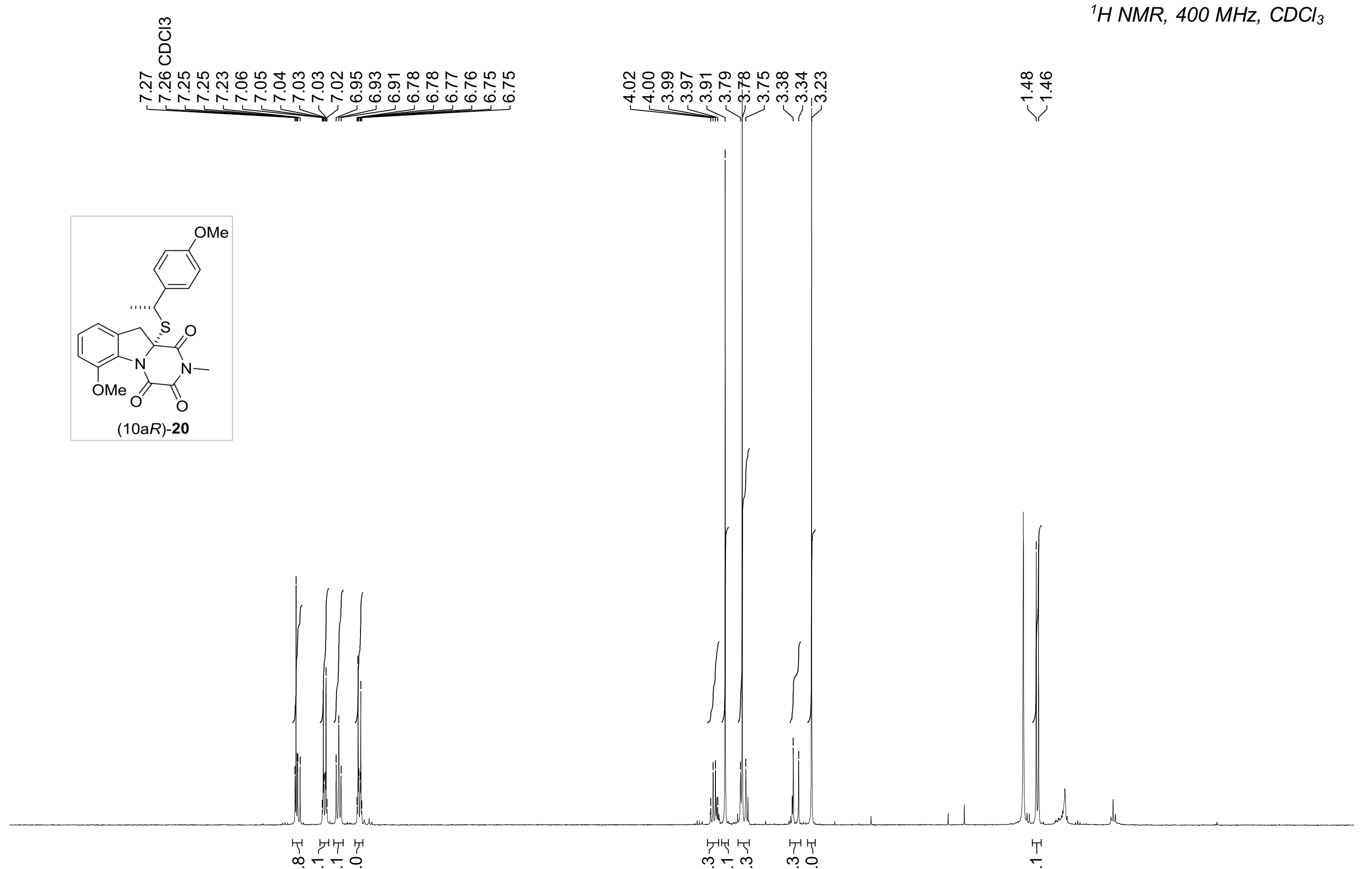
1H NMR, 400 MHz, CDCl3
7.26 CDCl3
OMe
S
N
O
OMe
(10aR)-20
1.8
2.1
2.1
2.0
1.3
3.1
4.3
1.3
3.0
3.1

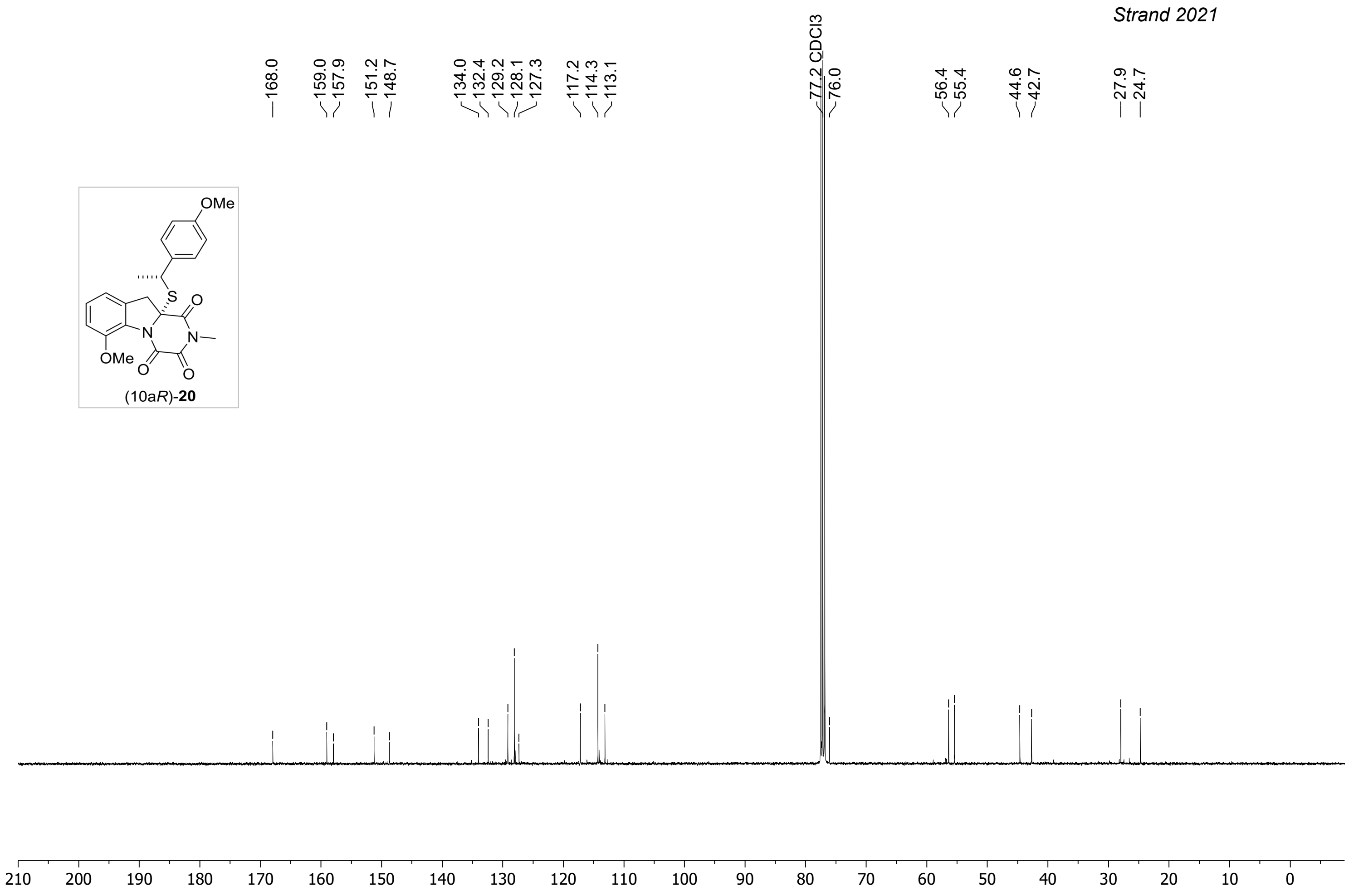

^{13}C NMR, 101 MHz, $CDCl_3$
Strand 2021
168.0
159.0
157.9
151.2
148.7
134.0
132.4
129.2
128.1
127.3
117.2
114.3
113.1
77.2 CDCl3
76.0
56.4
55.4
44.6
42.7
27.9
24.7
OMe
S
O
N
N
OMe
O
O
(10a*R*)-**20**
210 200 190 180 170 160 150 140 130 120 110 100 90 80 70 60 50 40 30 20 10 0

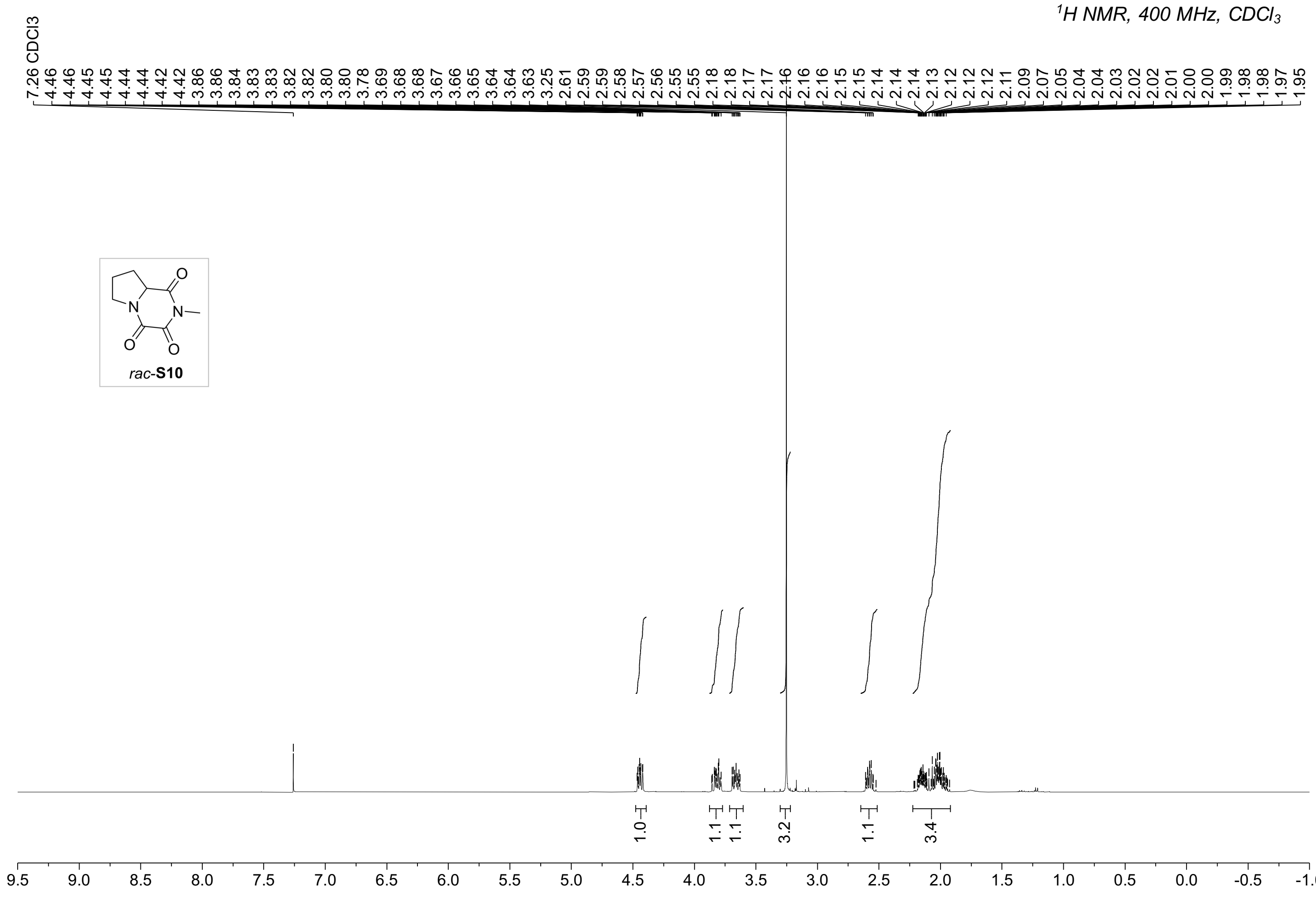

^{1}H NMR, 400 MHz, $CDCl_3$
7.26 CDCl3
4.46
4.46
4.45
4.45
4.44
4.44
4.42
4.42
3.86
3.86
3.84
3.83
3.83
3.82
3.82
3.80
3.80
3.78
3.69
3.68
3.68
3.67
3.66
3.65
3.64
3.64
3.63
3.25
2.61
2.60
2.59
2.59
2.58
2.57
2.56
2.55
2.55
2.18
2.18
2.17
2.17
2.16
2.16
2.16
2.15
2.15
2.14
2.14
2.14
2.13
2.13
2.12
2.12
2.12
2.11
2.09
2.07
2.05
2.04
2.04
2.03
2.02
2.02
2.01
2.00
2.00
1.99
1.98
1.98
1.97
1.95
O
N
N
O
O
rac-**S10**
1.0
1.1
1.1
3.2
1.1
3.4
9.5 9.0 8.5 8.0 7.5 7.0 6.5 6.0 5.5 5.0 4.5 4.0 3.5 3.0 2.5 2.0 1.5 1.0 0.5 0.0 -0.5 -1.0

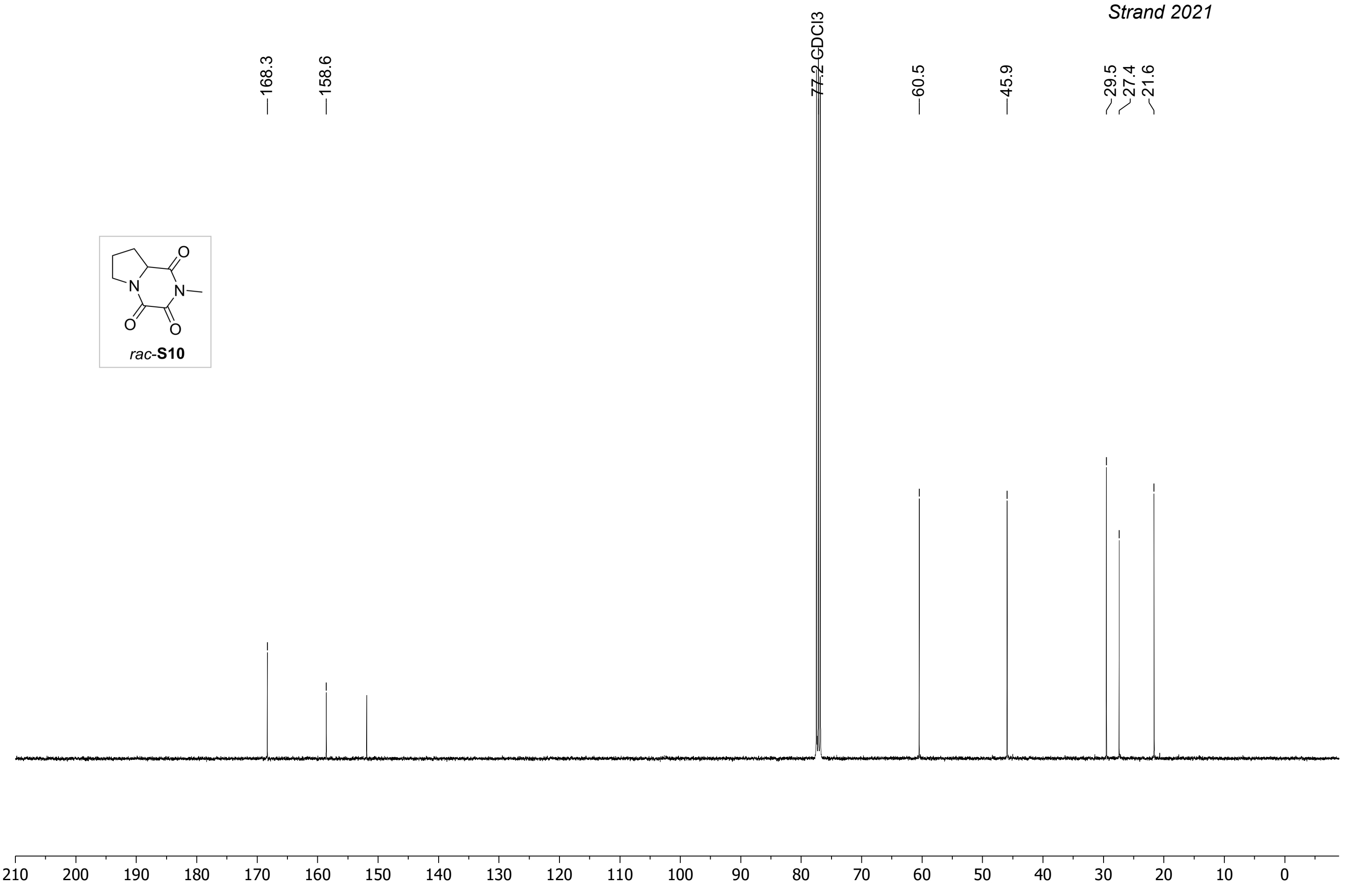

13C NMR, 101 MHz, CDCl3
Strand 2021
168.3
158.6
77.2 CDCl3
60.5
45.9
29.5
27.4
21.6
rac-S10
210 200 190 180 170 160 150 140 130 120 110 100 90 80 70 60 50 40 30 20 10 0

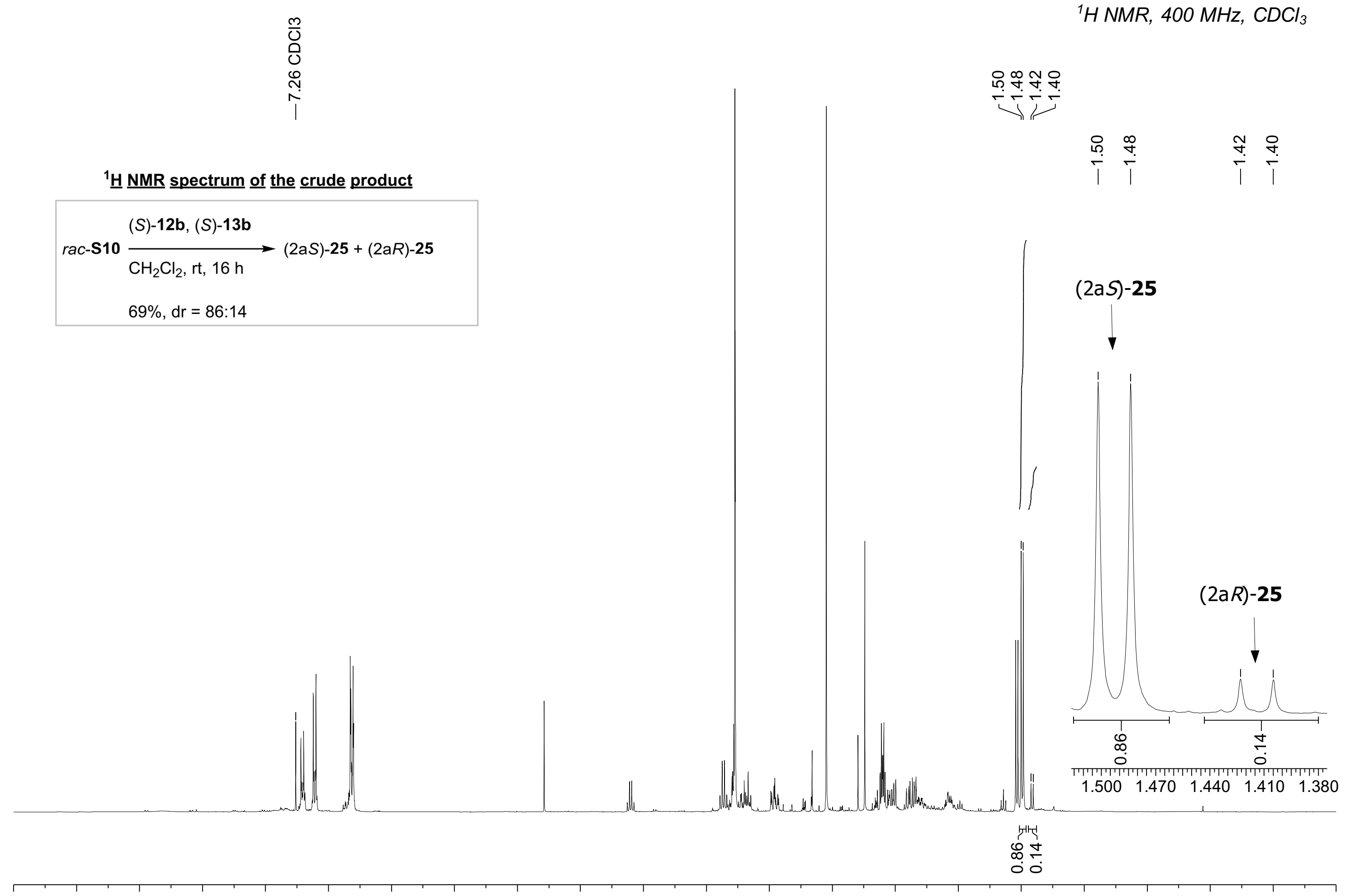

1H NMR, 400 MHz, CDCl3
7.26 CDCl3
1.50
1.48
1.42
1.40
1H NMR spectrum of the crude product
rac-S10
(S)-12b, (S)-13b
CH2Cl2, rt, 16 h
(2aS)-25 + (2aR)-25
69%, dr = 86:14
(2aS)-25
(2aR)-25
0.86
0.14
1.500 1.470 1.440 1.410 1.380
9.5 9.0 8.5 8.0 7.5 7.0 6.5 6.0 5.5 5.0 4.5 4.0 3.5 3.0 2.5 2.0 1.5 1.0 0.5 0.0 -0.5 -1.0

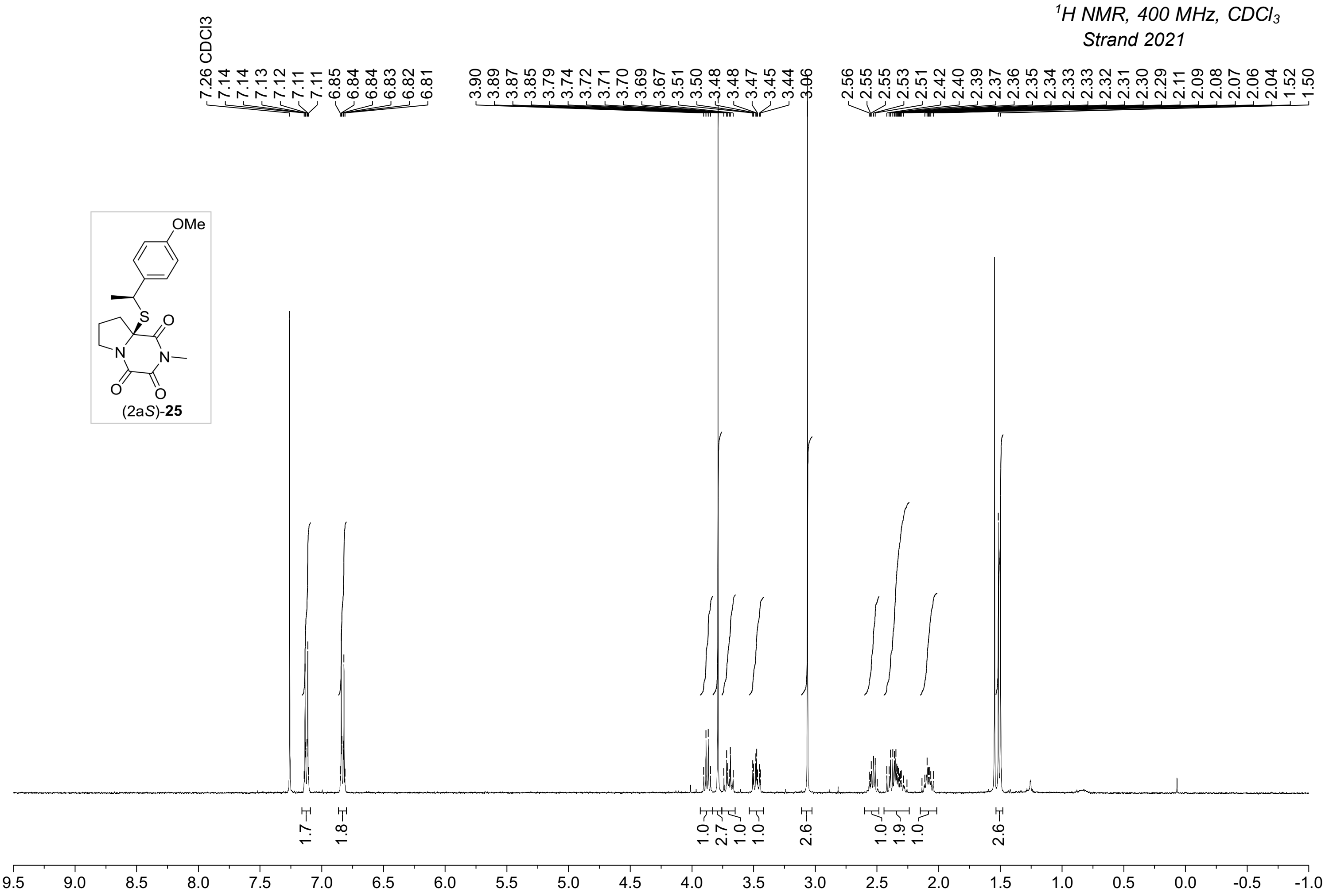
1H NMR, 400 MHz, CDCl3
Strand 2021
7.26 CDCl3
7.14 7.13 7.12 7.11 7.11 6.85 6.84 6.84 6.83 6.82 6.81
3.90 3.89 3.87 3.85 3.79 3.74 3.72 3.71 3.70 3.69 3.67 3.51 3.50 3.48 3.48 3.47 3.45 3.44 3.06
2.56 2.55 2.55 2.53 2.51 2.42 2.40 2.39 2.37 2.36 2.35 2.34 2.33 2.33 2.32 2.31 2.30 2.29 2.11 2.09 2.08 2.07 2.06 2.04 1.52 1.50
OMe
S
O
N
N
O
O
(2aS)-25
1.7
1.8
1.0
2.7
1.0
1.0
2.6
1.0
1.9
1.0
2.6
9.5 9.0 8.5 8.0 7.5 7.0 6.5 6.0 5.5 5.0 4.5 4.0 3.5 3.0 2.5 2.0 1.5 1.0 0.5 0.0 -0.5 -1.0

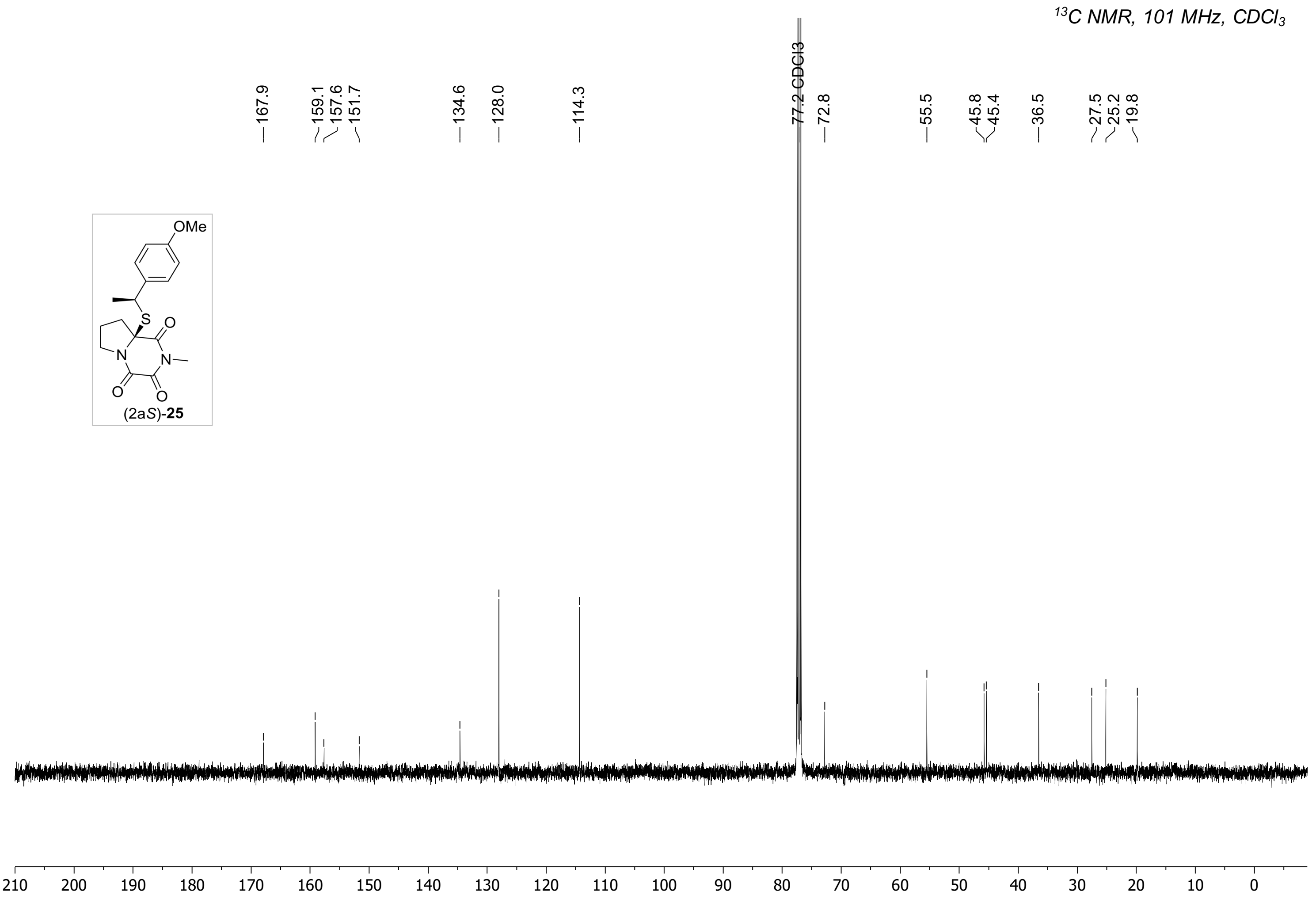
13C NMR, 101 MHz, CDCl3
167.9
159.1 157.6 151.7
134.6
128.0
114.3
77.2 CDCl3
72.8
55.5
45.8 45.4
36.5
27.5 25.2 19.8
OMe
S
O
N
N
O
O
(2aS)-25
210 200 190 180 170 160 150 140 130 120 110 100 90 80 70 60 50 40 30 20 10 0

^{1}H NMR, 400 MHz, $CDCl_3$
Strand 2021

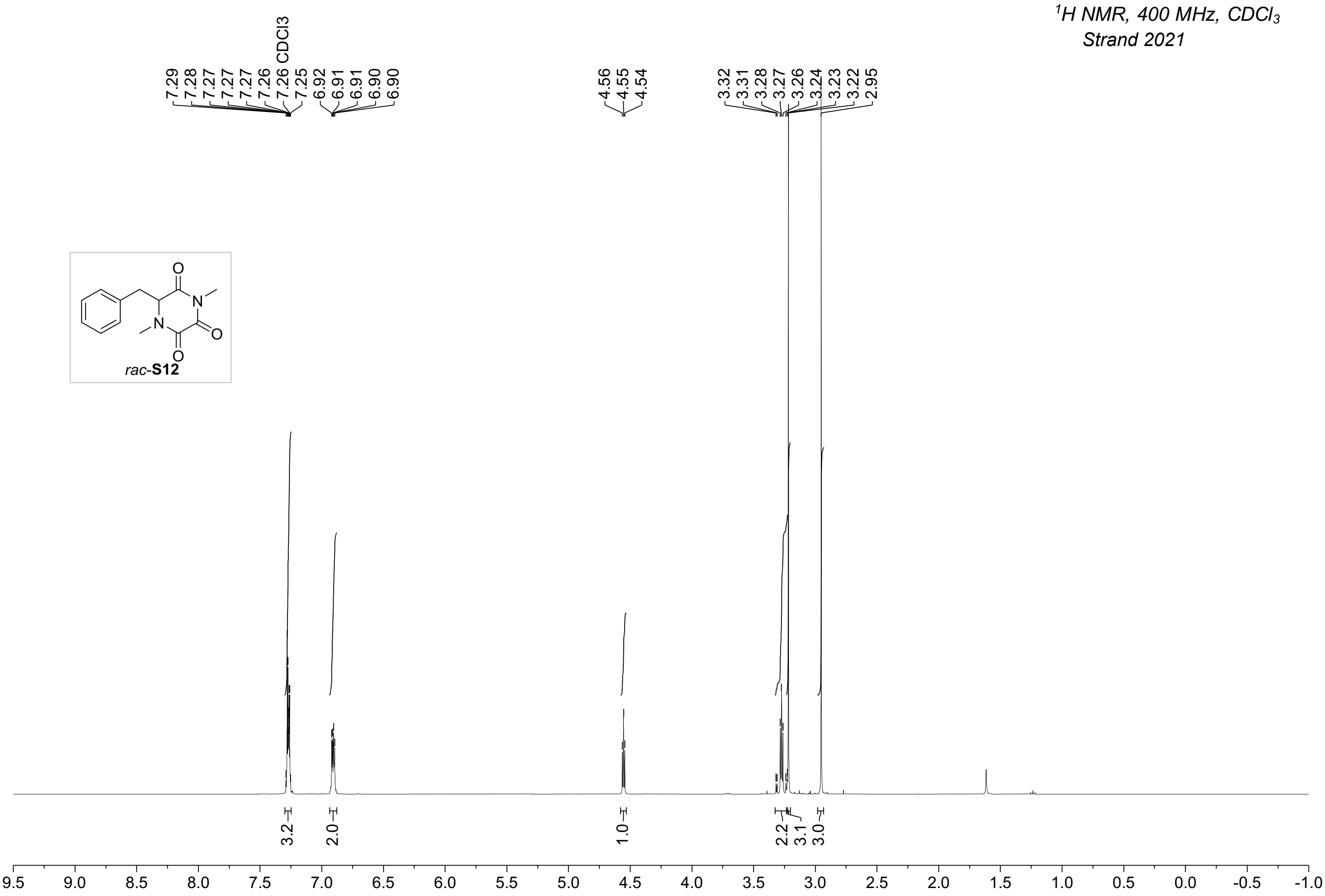

^{13}C NMR, 101 MHz, $CDCl_3$

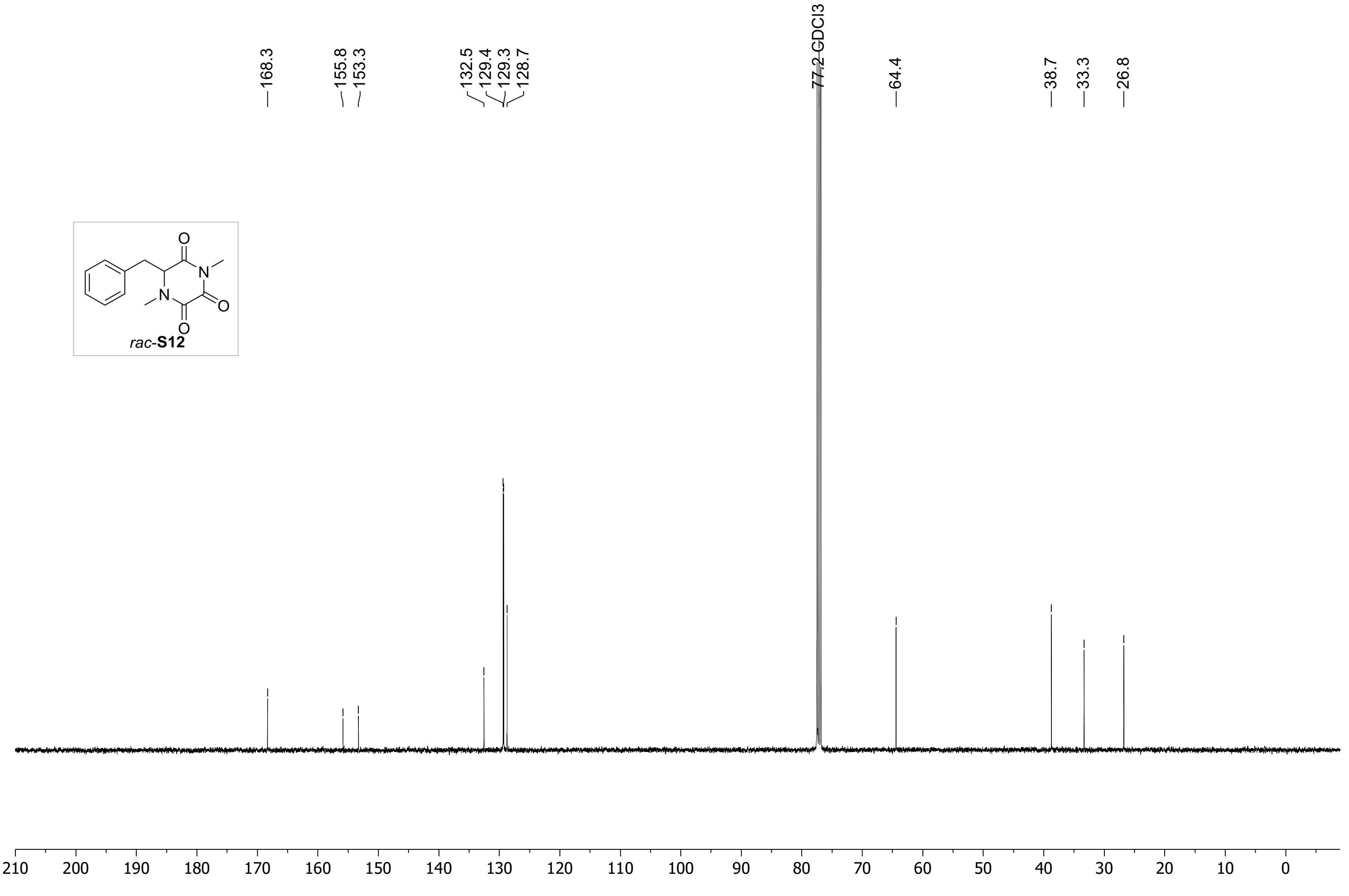

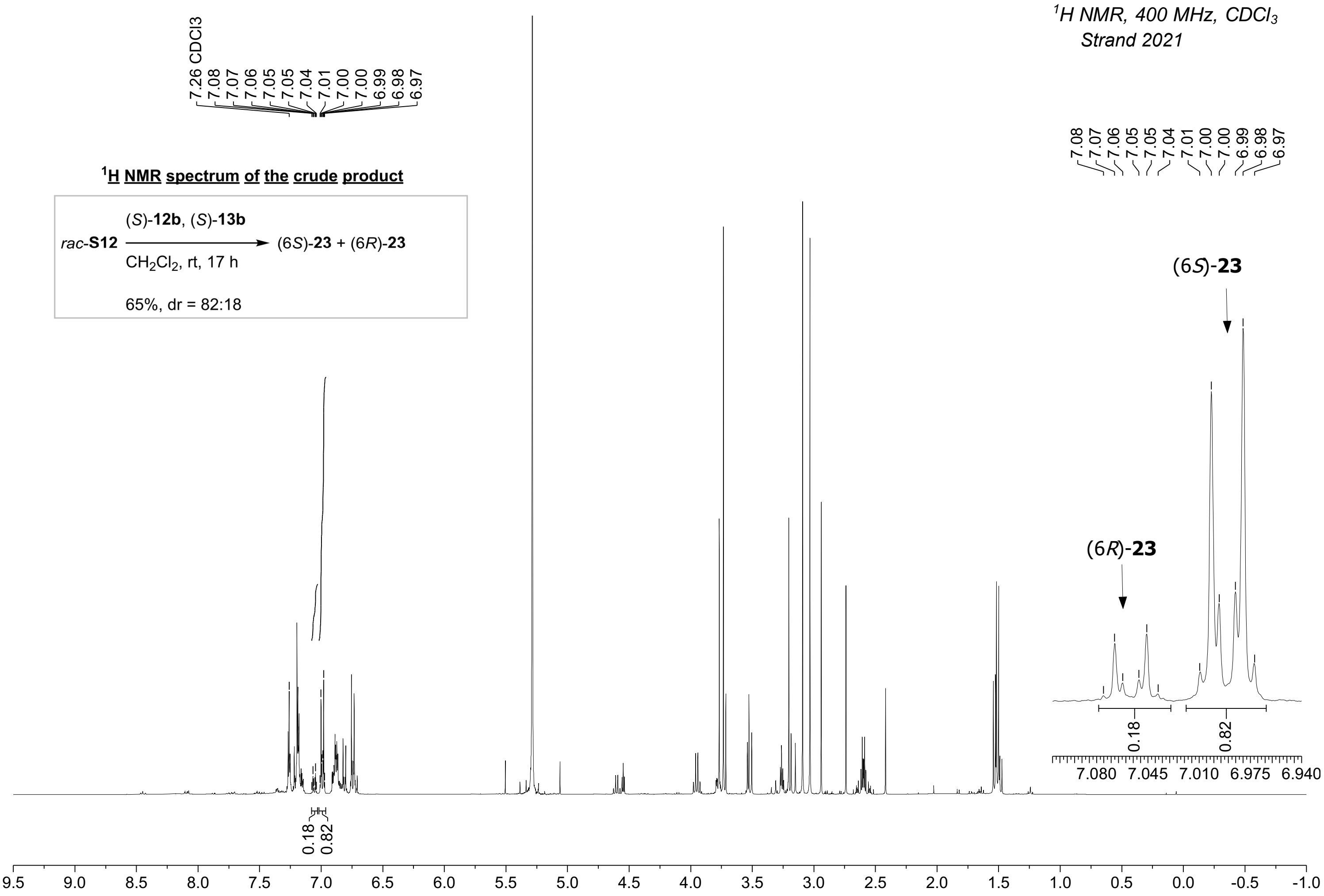
^{1}H NMR, 400 MHz, CDCl$_3$
Strand 2021
7.26 CDCl3
7.08 7.07 7.06 7.05 7.05 7.04 7.01 7.00 6.99 6.98 6.97
^{1}H NMR spectrum of the crude product
(S)-12b, (S)-13b
rac-S12 → (6S)-23 + (6R)-23
CH$_2$Cl$_2$, rt, 17 h
65%, dr = 82:18
(6S)-23
(6R)-23
0.18
0.82
7.080 7.045 7.010 6.975 6.940
9.5 9.0 8.5 8.0 7.5 7.0 6.5 6.0 5.5 5.0 4.5 4.0 3.5 3.0 2.5 2.0 1.5 1.0 0.5 0.0 -0.5 -1.0

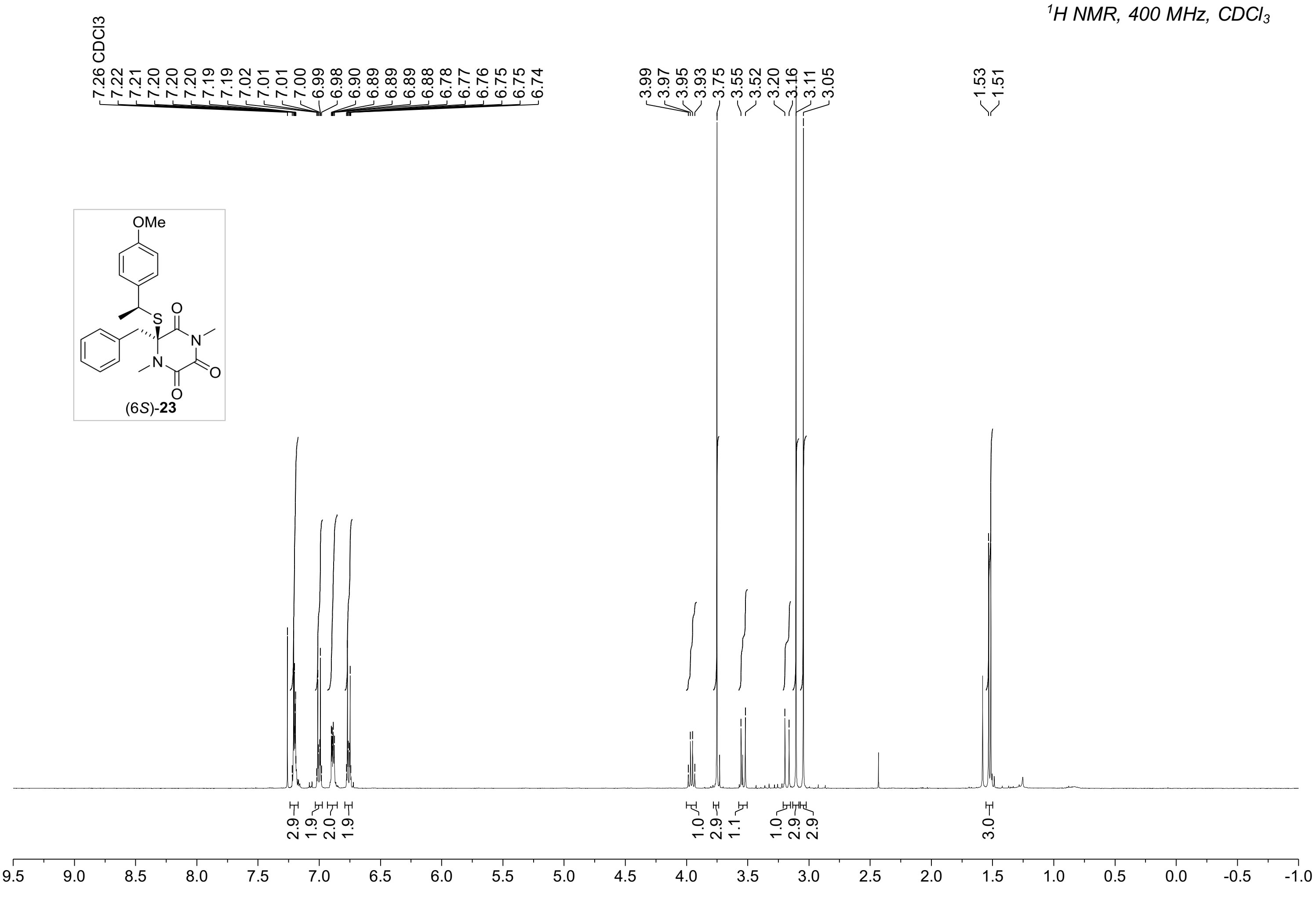
^{1}H NMR, 400 MHz, CDCl$_3$
7.26 CDCl3
7.22 7.21 7.20 7.20 7.20 7.19 7.19 7.02 7.01 7.00 6.99 6.98 6.90 6.89 6.89 6.88 6.78 6.77 6.76 6.75 6.74
3.99 3.97 3.95 3.93 3.75 3.55 3.52 3.20 3.16 3.11 3.05
1.53 1.51
OMe
S
O
N
O
(6S)-23
2.9 1.9 2.0 1.9
1.0 2.9 1.1 1.0 2.9 2.9
3.0
9.5 9.0 8.5 8.0 7.5 7.0 6.5 6.0 5.5 5.0 4.5 4.0 3.5 3.0 2.5 2.0 1.5 1.0 0.5 0.0 -0.5 -1.0

^{13}C NMR, 101 MHz, $CDCl_3$
Strand 2021

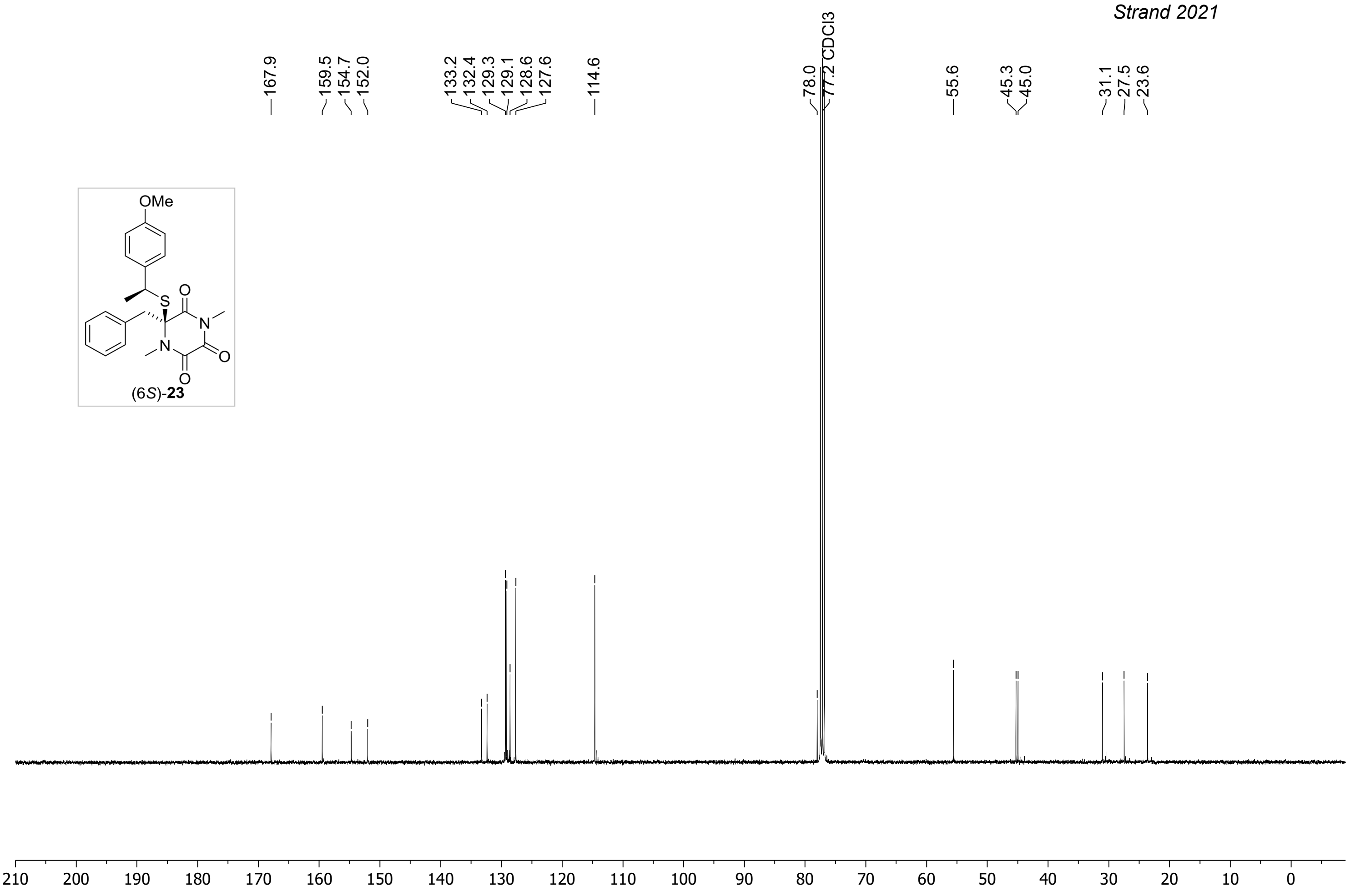

^{1}H NMR, 400 MHz, $CDCl_3$

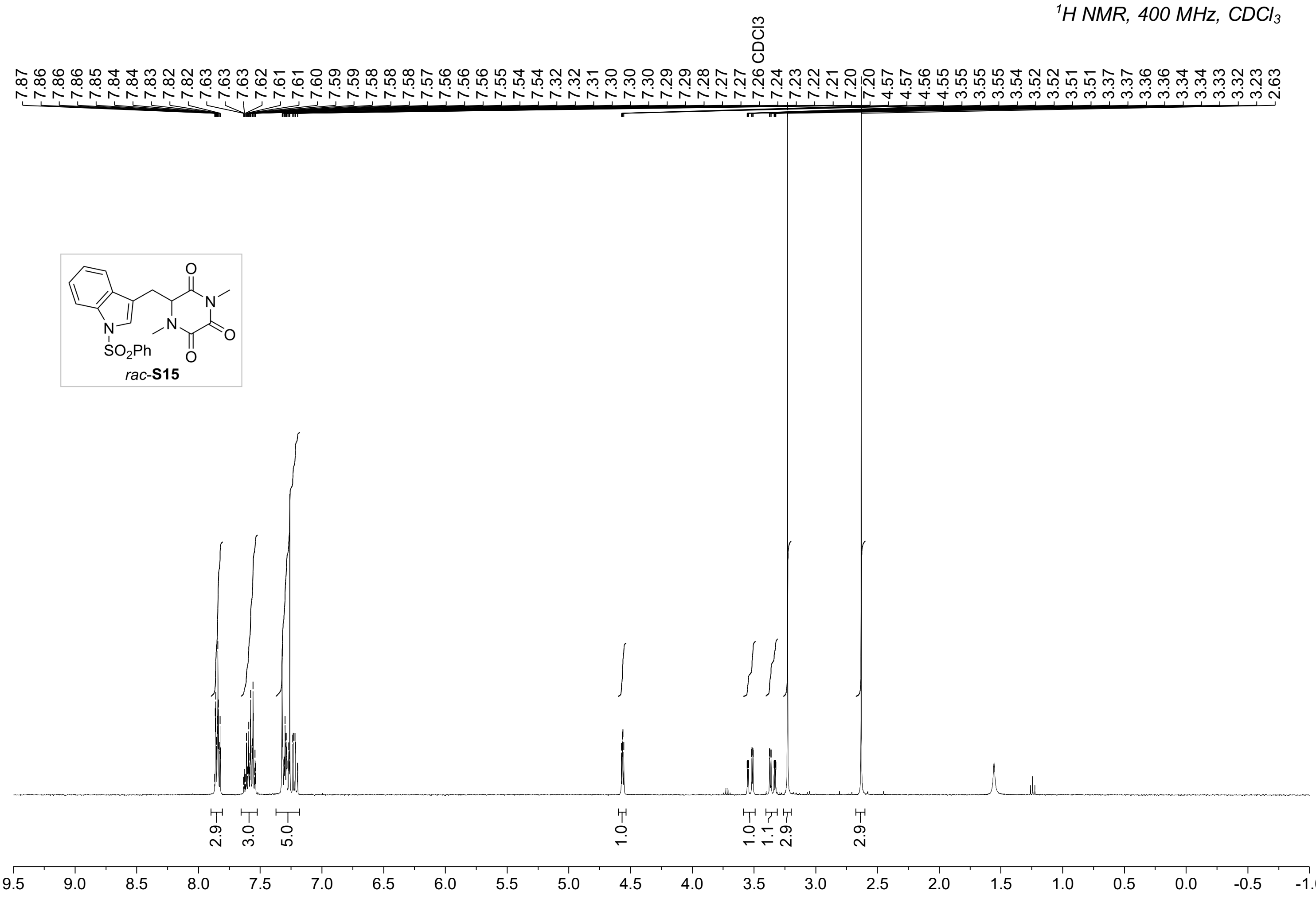

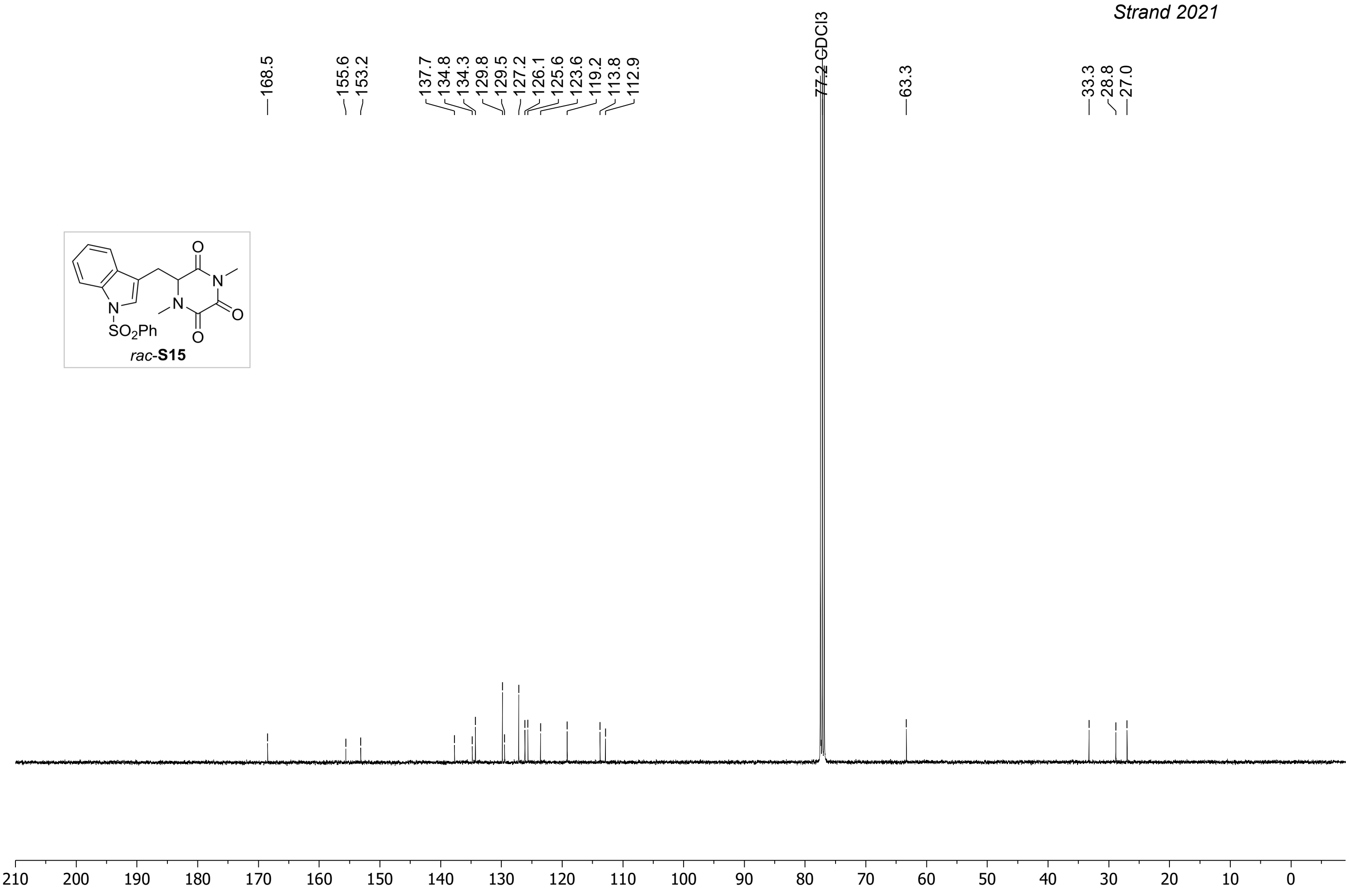
^{13}C NMR, 101 MHz, $CDCl_3$
Strand 2021
168.5
155.6
153.2
137.7
134.8
134.3
129.8
129.5
127.2
126.1
125.6
123.6
119.2
113.8
112.9
77.2 CDCl3
63.3
33.3
28.8
27.0
SO_2Ph
rac-**S15**

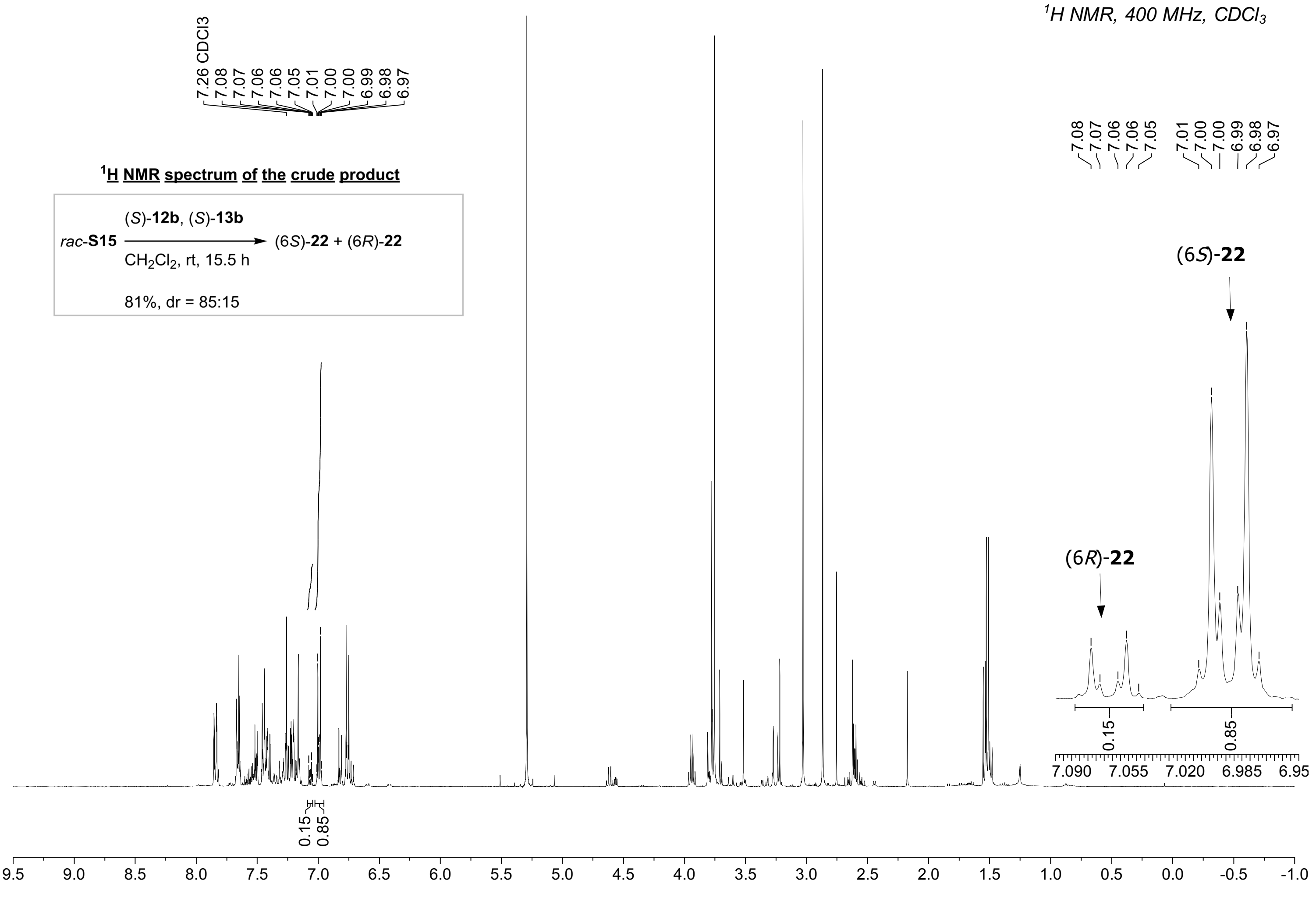
^{1}H NMR, 400 MHz, $CDCl_3$
7.26 CDCl3
7.08
7.07
7.06
7.06
7.05
7.01
7.00
7.00
6.99
6.98
6.97
^{1}H NMR spectrum of the crude product
rac-**S15**
(*S*)-**12b**, (*S*)-**13b**
CH_2Cl_2, rt, 15.5 h
(6*S*)-**22** + (6*R*)-**22**
81%, dr = 85:15
(6*S*)-**22**
(6*R*)-**22**
0.15
0.85

^{1}H NMR, 400 MHz, $CDCl_3$
Strand 2021

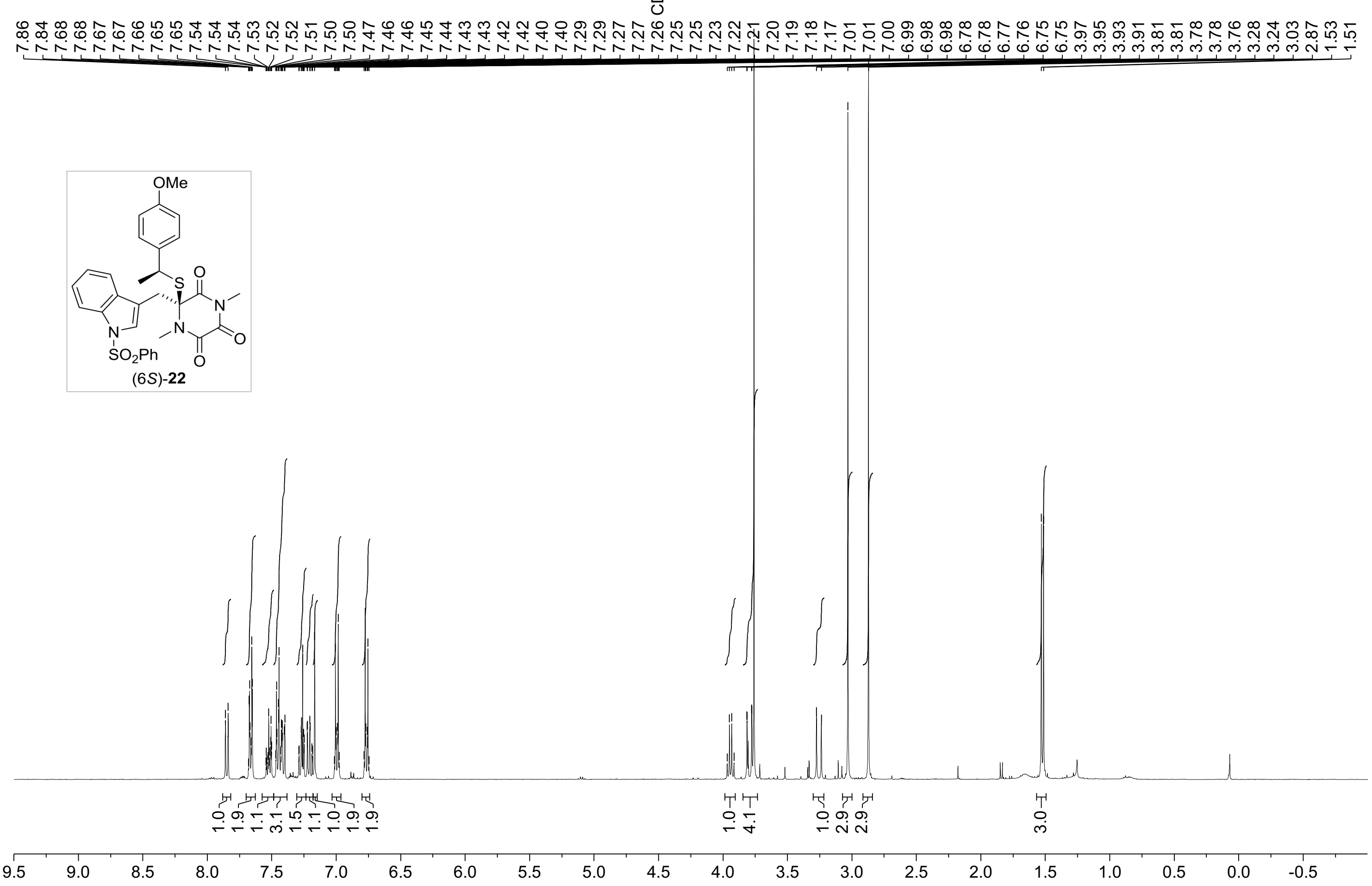

^{13}C NMR, 101 MHz, $CDCl_3$

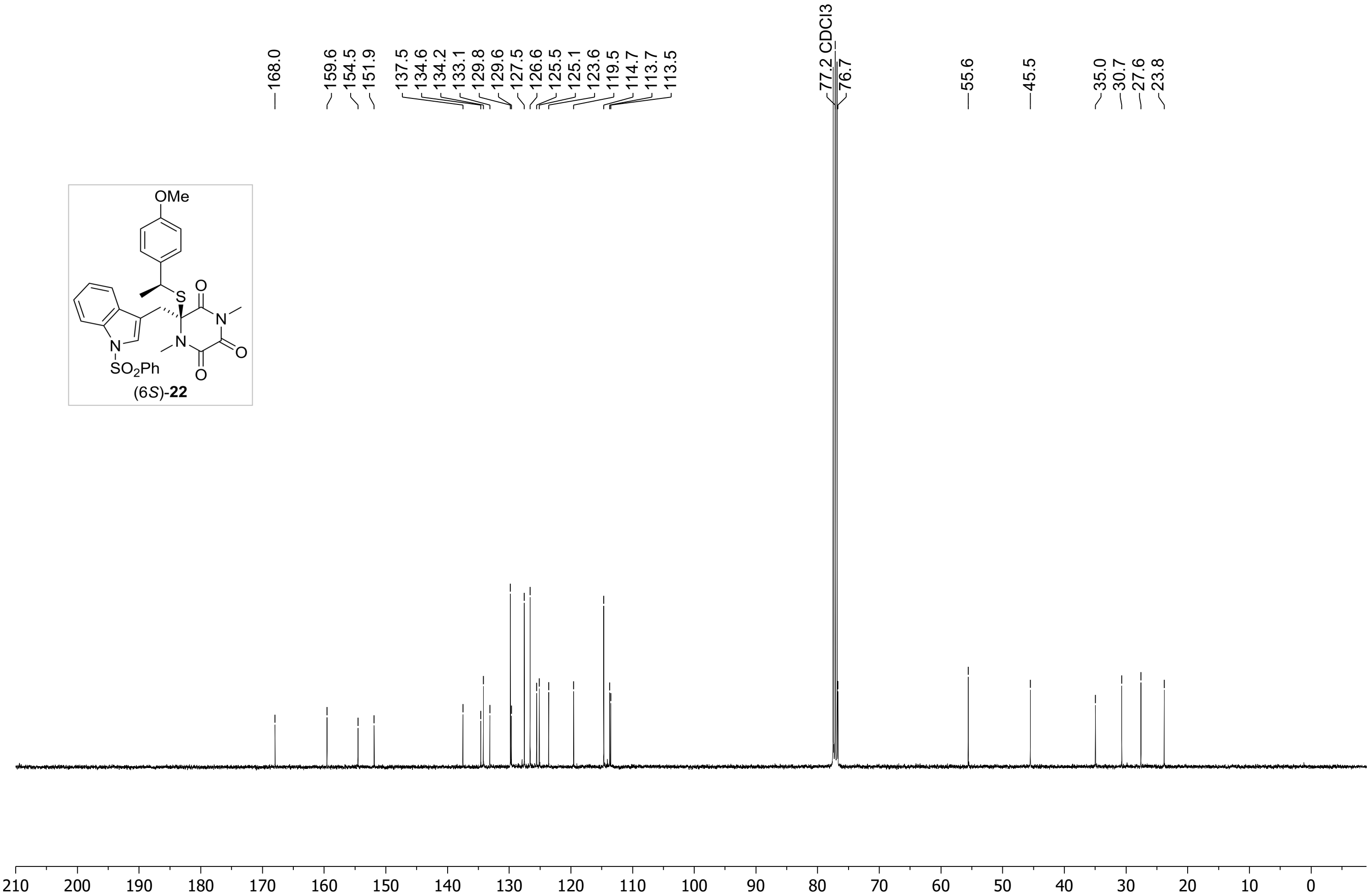

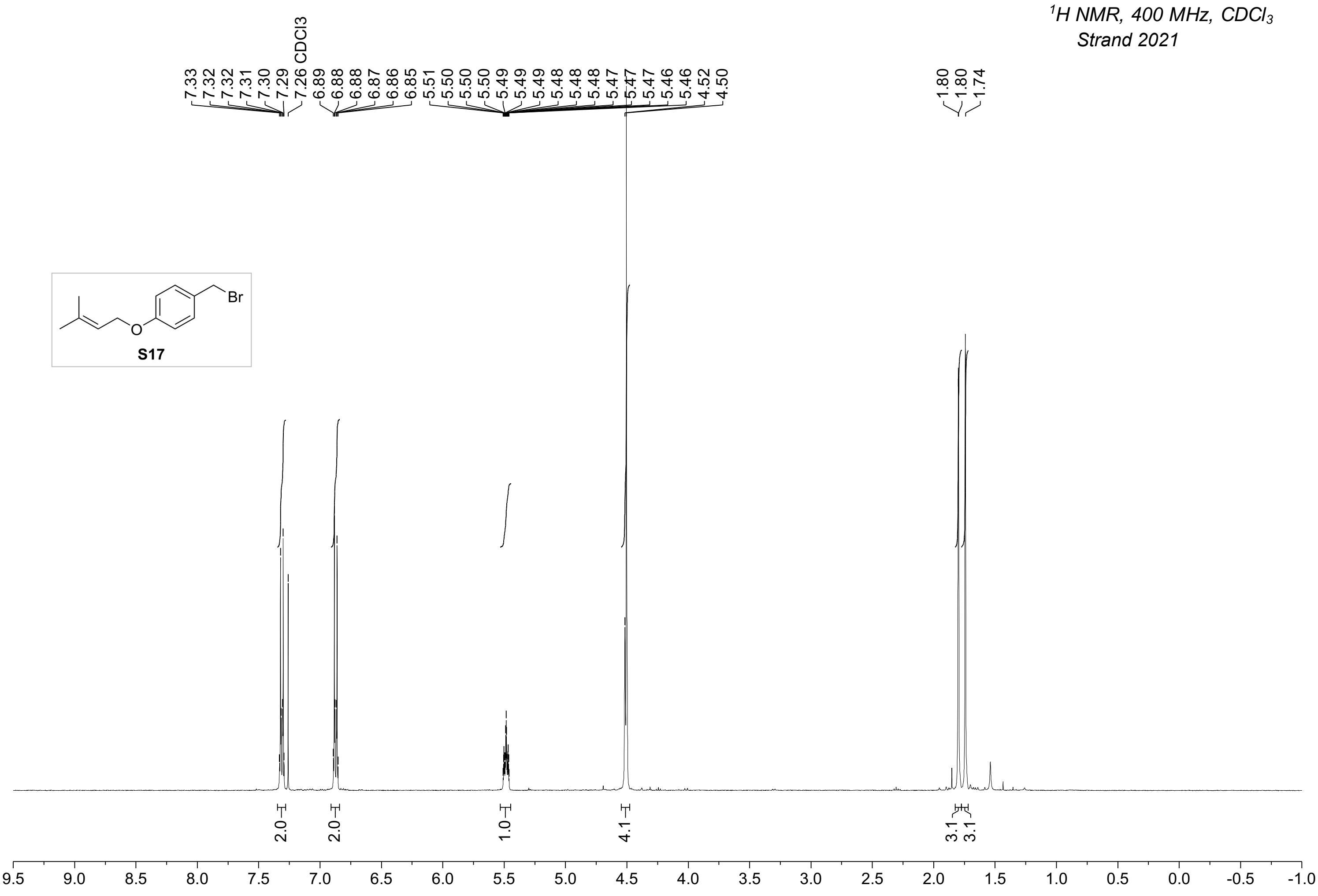
1H NMR, 400 MHz, CDCl3
Strand 2021
7.33 7.32 7.32 7.31 7.30 7.29 7.26 CDCl3 6.89 6.88 6.88 6.87 6.86 6.85 5.51 5.50 5.50 5.49 5.49 5.49 5.48 5.48 5.48 5.47 5.47 5.47 5.46 5.46 4.52 4.50
1.80 1.80 1.74
Br
O
S17
2.0 2.0 1.0 4.1 3.1 3.1
9.5 9.0 8.5 8.0 7.5 7.0 6.5 6.0 5.5 5.0 4.5 4.0 3.5 3.0 2.5 2.0 1.5 1.0 0.5 0.0 -0.5 -1.0

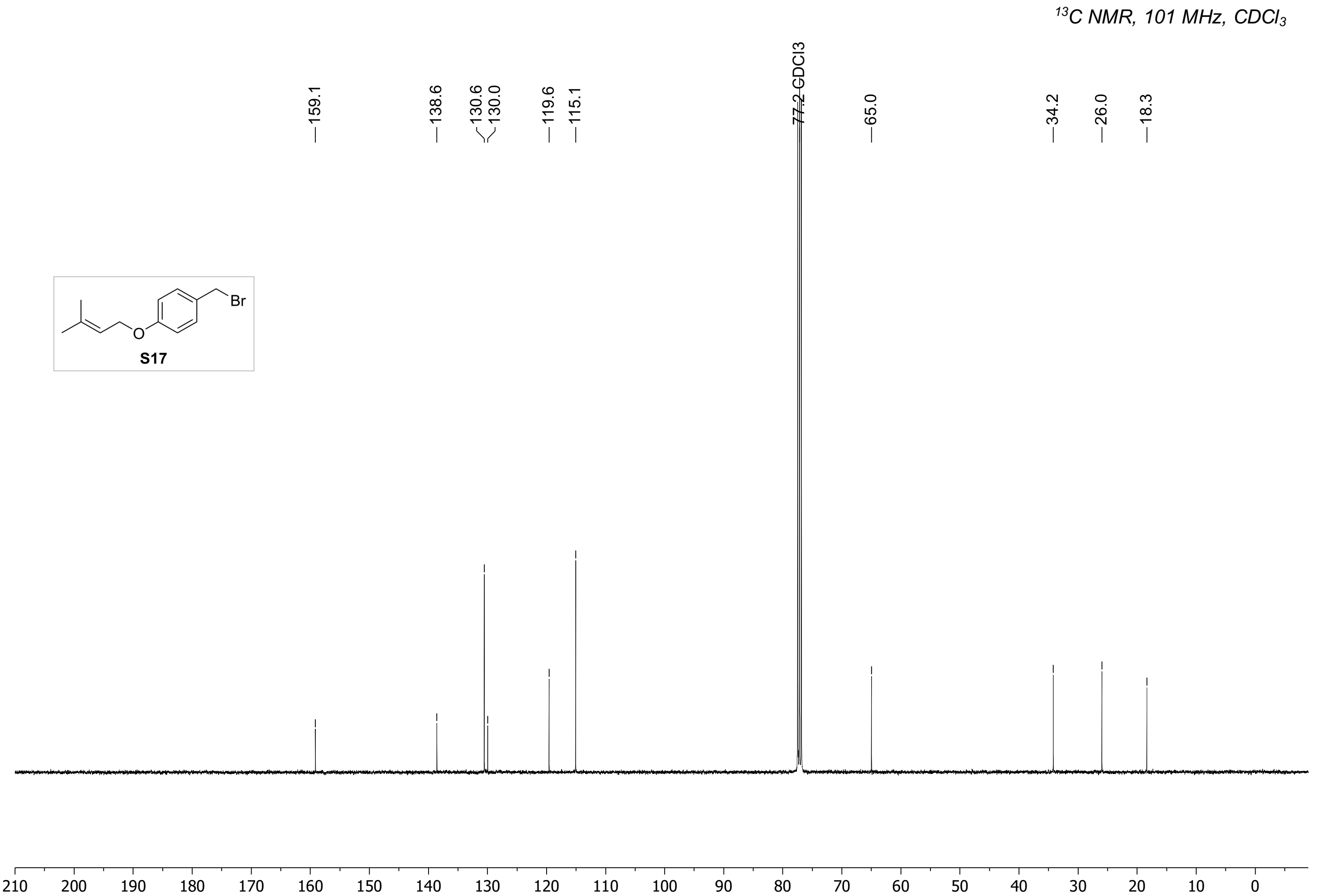
13C NMR, 101 MHz, CDCl3
159.1
138.6
130.6 130.0
119.6
115.1
77.2 CDCl3
65.0
34.2
26.0
18.3
Br
O
S17
210 200 190 180 170 160 150 140 130 120 110 100 90 80 70 60 50 40 30 20 10 0

^{1}H NMR, 400 MHz, $CDCl_3$
Strand 2021

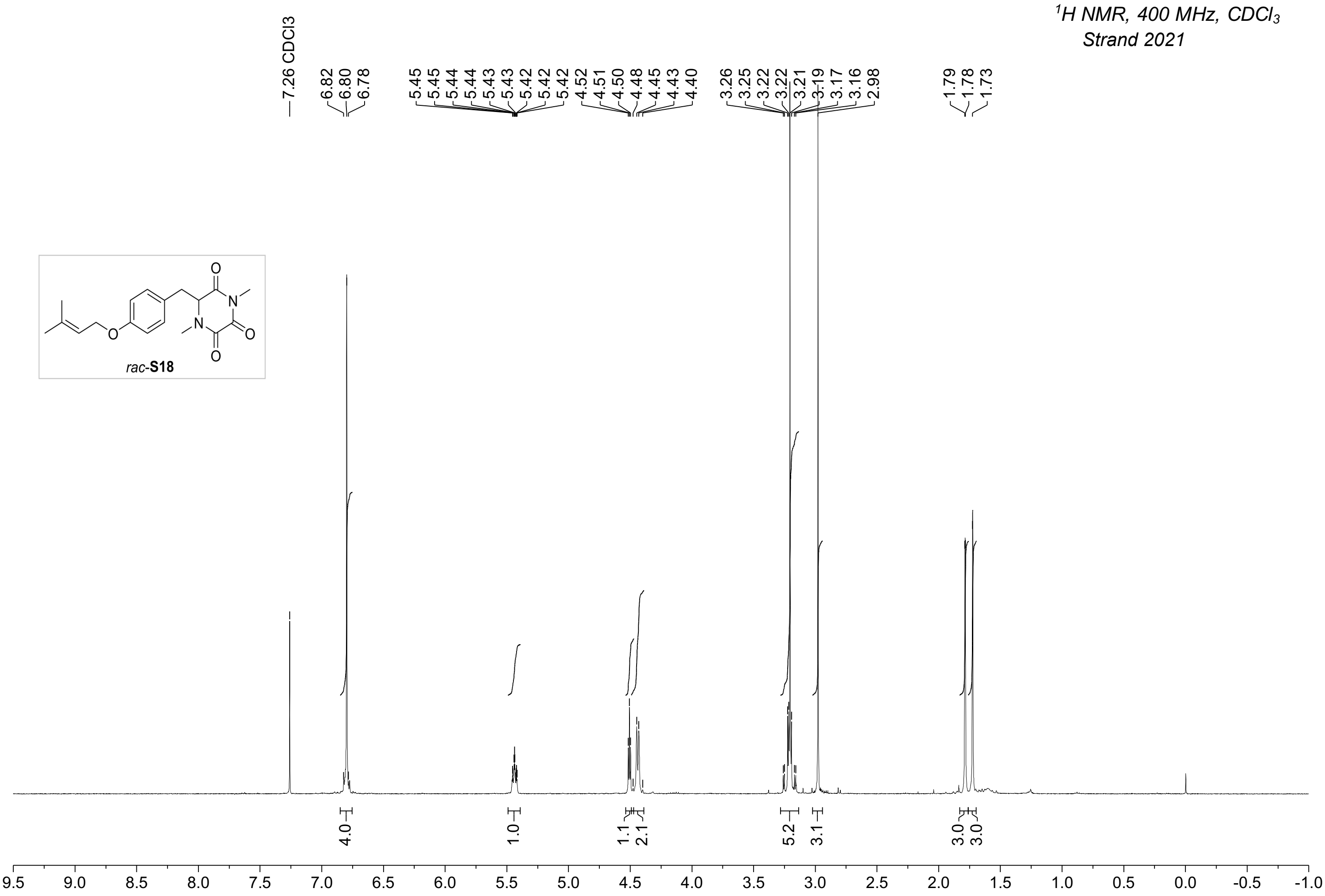

^{13}C NMR, 101 MHz, $CDCl_3$

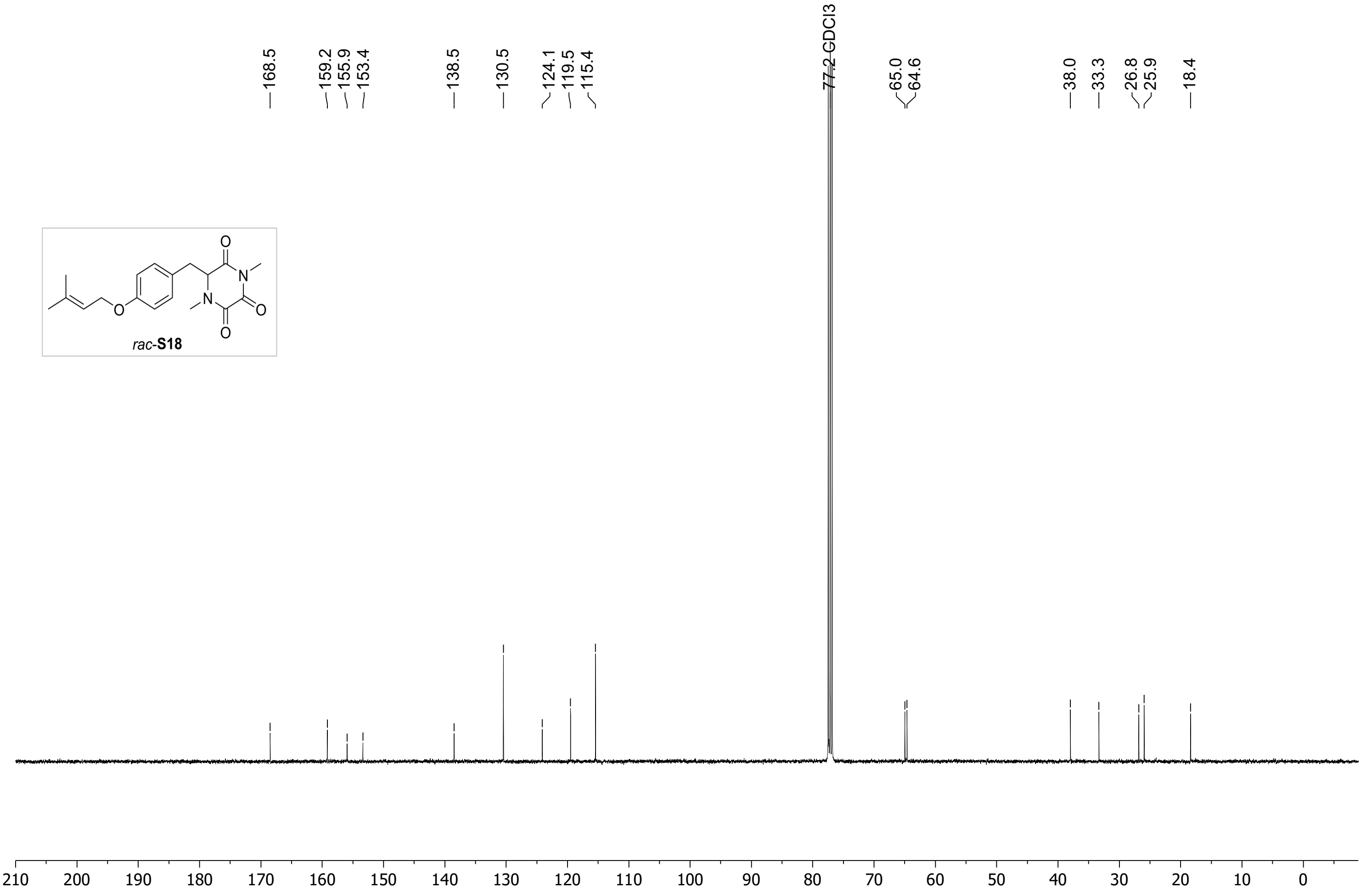

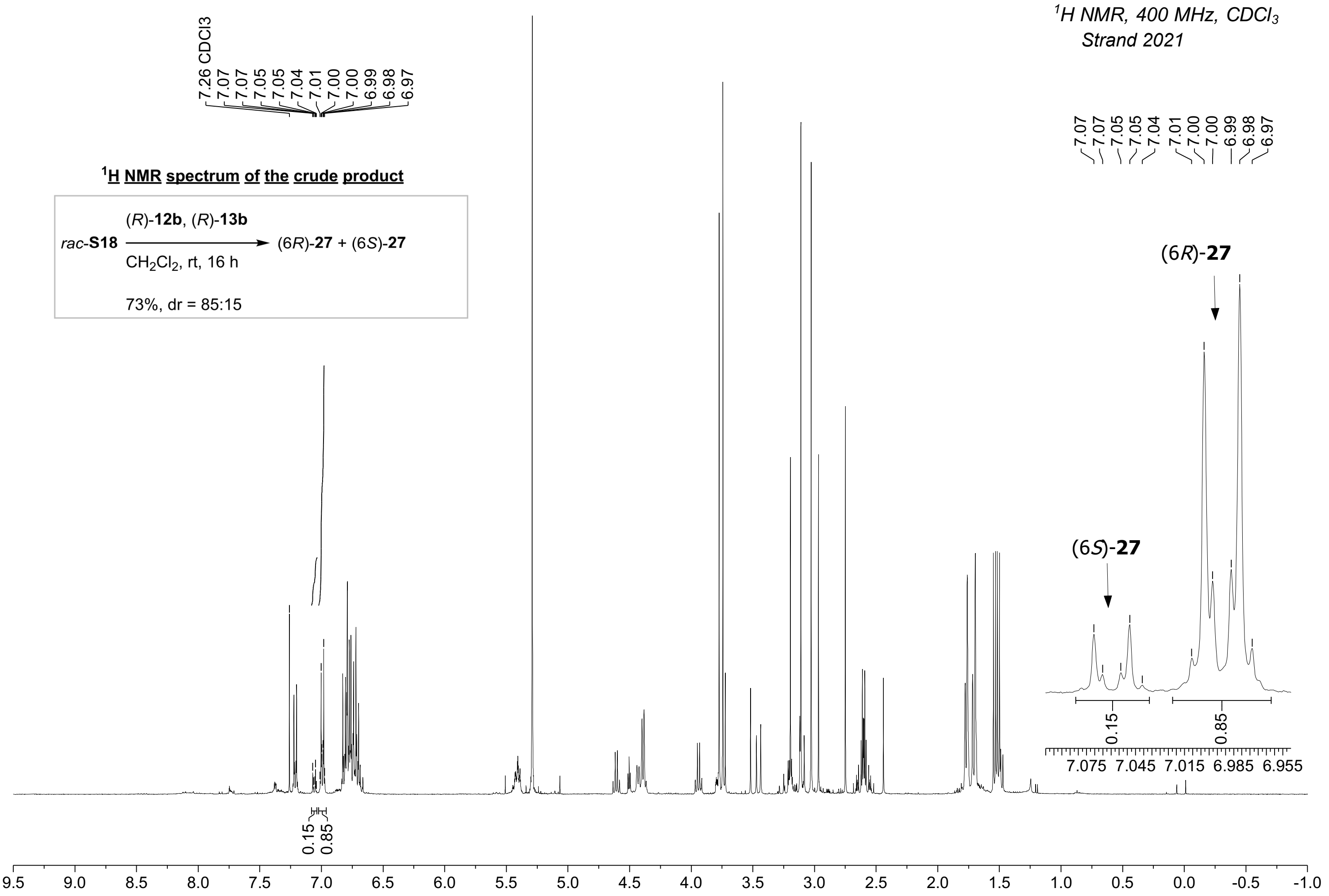

^{1}H NMR, 400 MHz, $CDCl_3$
Strand 2021
^{1}H NMR spectrum of the crude product
(R)-12b, (R)-13b
rac-S18 → (6R)-27 + (6S)-27
CH_2Cl_2, rt, 16 h
73%, dr = 85:15
7.26 CDCl3 7.07 7.07 7.05 7.05 7.04 7.01 7.00 7.00 6.99 6.98 6.97
(6R)-27
(6S)-27
0.15
0.85
7.075 7.045 7.015 6.985 6.955
9.5 9.0 8.5 8.0 7.5 7.0 6.5 6.0 5.5 5.0 4.5 4.0 3.5 3.0 2.5 2.0 1.5 1.0 0.5 0.0 -0.5 -1.0

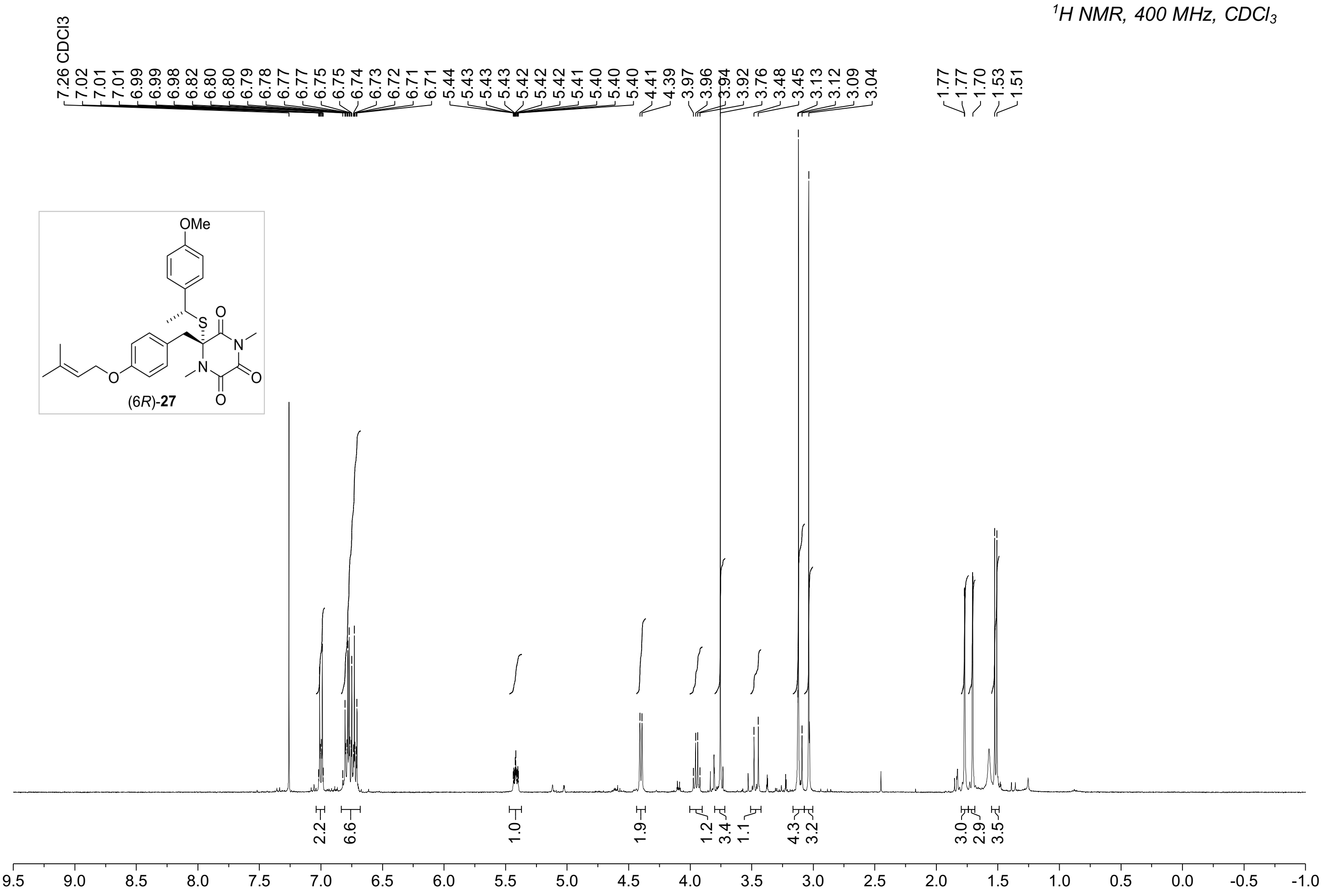

^{1}H NMR, 400 MHz, $CDCl_3$
7.26 CDCl3 7.02 7.01 7.01 6.99 6.99 6.98 6.82 6.80 6.80 6.79 6.78 6.77 6.77 6.75 6.75 6.74 6.73 6.72 6.71 5.44 5.43 5.43 5.42 5.42 5.42 5.41 5.40 5.40 5.40 4.41 4.39 3.97 3.96 3.94 3.92 3.76 3.48 3.45 3.13 3.12 3.09 3.04 1.77 1.77 1.70 1.53 1.51
OMe
S
O
N
O
(6R)-27
2.2 6.6 1.0 1.9 1.2 3.4 1.1 4.3 3.2 3.0 2.9 3.5
9.5 9.0 8.5 8.0 7.5 7.0 6.5 6.0 5.5 5.0 4.5 4.0 3.5 3.0 2.5 2.0 1.5 1.0 0.5 0.0 -0.5 -1.0

^{13}C NMR, 101 MHz, $CDCl_3$
Strand 2021

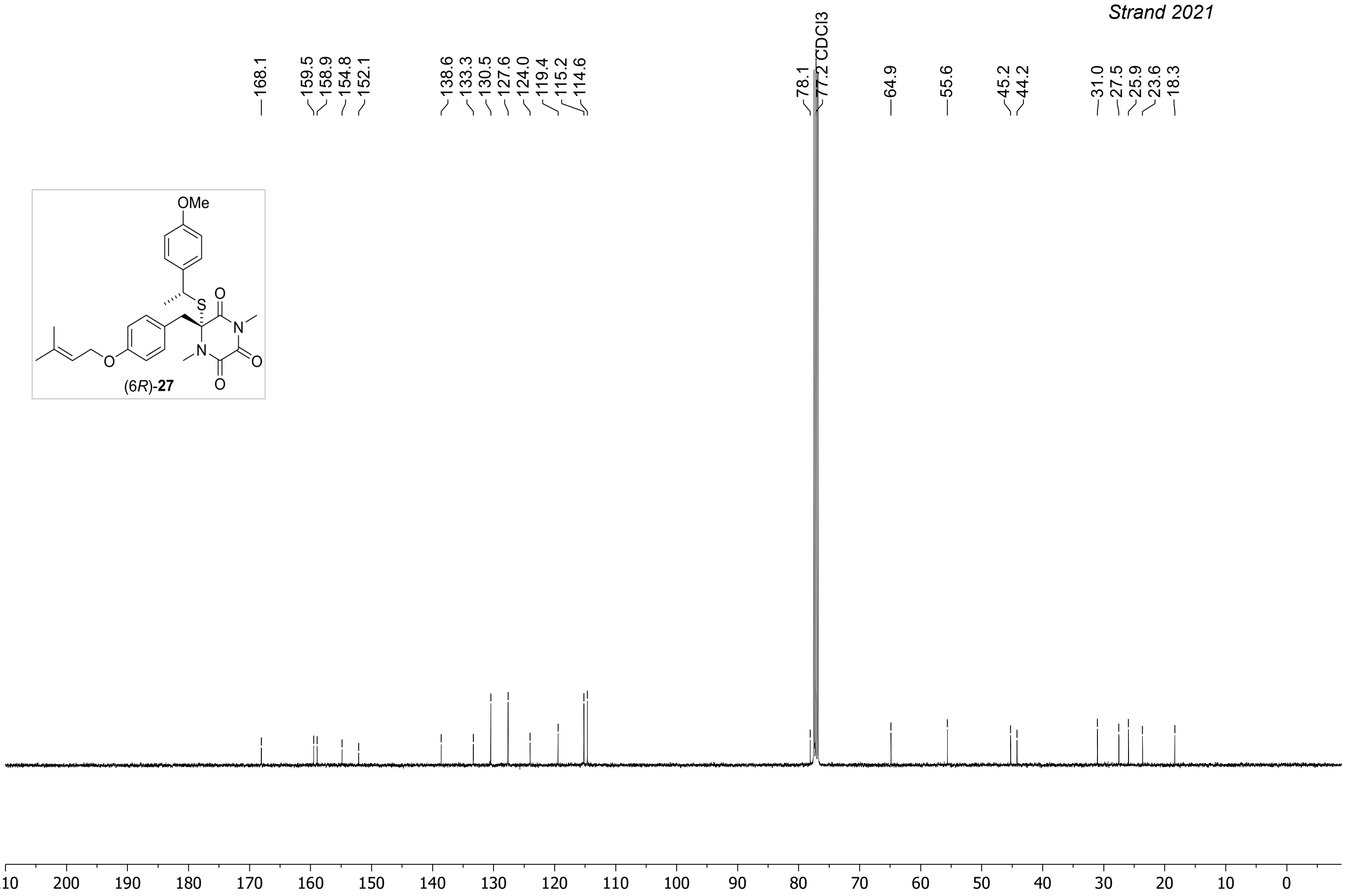

Supplement: Supplementary file 1 — ja1c10364_si_001.pdf [file ja1c10364_si_001.pdf]
