# Supplementary material for: Total Synthesis of (−)-Glionitrin A and B Enabled by an Asymmetric Oxidative Sulfenylation of Triketopiperazines
Source: J Am Chem Soc. 2021 Nov 22;143(50):21218–22. doi: 10.1021/jacs.1c10364 (PMC8704193; doi:10.1021/jacs.1c10364)
Supplement: Supplementary file 2 — ja1c10364_si_002.xyz [file ja1c10364_si_002.xyz]

77        TS-major 17C -1.118309 1.519937 0.273099C -0.848888 2.791214 -0.212633C -0.085478 3.048995 -1.346607C -0.587842 0.422219 -0.400847H -1.725553 1.409800 1.162026H 0.099491 4.054611 -1.697618H -0.778269 -0.583229 -0.040441C 0.422657 1.931270 -1.991209C 0.181862 0.627900 -1.533717C 0.838050 -0.373619 -2.451400H 0.090788 -0.970335 -2.994229N -1.377638 3.940303 0.530384O -0.929169 5.042721 0.261913O -2.224075 3.726460 1.377671H 1.466706 -1.086741 -1.905331C 1.620957 0.527126 -3.368706N 1.204983 1.874972 -3.153995N 2.477658 1.255119 -5.476751C 2.061908 0.163903 -4.657127C 1.741917 2.933880 -3.807221O 1.594619 4.113600 -3.481459C 2.538974 2.563585 -5.054456O 3.144820 3.439085 -5.656423C 3.164778 0.891991 -6.708253H 3.296780 1.793142 -7.303359H 2.569510 0.147265 -7.234041H 4.139874 0.449385 -6.474860O 2.200438 -0.992061 -5.073227C 6.202000 2.020984 -4.978877C 6.400666 2.644684 -6.191336C 6.164871 4.021606 -6.355315C 5.719297 4.741855 -5.278583H 6.370126 0.961289 -4.838807H 6.741767 2.054807 -7.035984H 6.303615 4.510652 -7.309910H 5.467044 5.794338 -5.312635N 5.552847 4.127164 -4.091031C 5.761412 2.797173 -3.896215N 5.450958 2.400504 -2.621728C 5.331393 3.681093 -1.846111C 4.887005 4.696993 -2.904801S 3.949301 1.301337 -2.681902H 6.364373 3.932614 -1.568687H 5.245396 5.707306 -2.705900H 3.807537 4.699979 -3.112943C 3.133867 3.564830 1.845694C 2.483178 3.944071 0.675015C 3.169077 3.960790 -0.536899C 4.523001 3.623112 -0.578093C 5.172079 3.255074 0.602551C 4.480244 3.211061 1.807925H 2.595093 3.554701 2.787682H 1.440826 4.246356 0.698305H 2.637006 4.236506 -1.444615H 6.229565 3.002723 0.574774H 4.995254 2.919451 2.717462C 4.760691 -0.230032 -2.059551H 3.904567 -0.916117 -2.044682C 5.274661 -0.028894 -0.641196H 5.738706 -0.948441 -0.271208H 4.456667 0.257187 0.023112H 6.018475 0.771901 -0.614441C 7.620842 -1.556993 -5.010486C 6.244114 -1.731443 -5.214073C 5.338916 -1.333535 -4.246051C 5.778775 -0.743001 -3.049565C 7.146160 -0.579554 -2.857813C 8.073279 -0.980251 -3.825744H 5.911729 -2.189040 -6.140015H 4.271650 -1.451612 -4.433123H 7.516604 -0.121928 -1.946380H 9.130724 -0.836571 -3.639914O 8.426839 -1.979908 -6.019977C 9.818993 -1.859832 -5.831604H 10.284838 -2.269140 -6.727291H 10.153955 -2.427647 -4.955537H 10.118810 -0.811264 -5.71337877        TS-minor 17C 3.126405 -0.509378 -7.782782C 3.189012 0.878469 -7.791254C 2.751389 1.684125 -6.744702C 2.617515 -1.148215 -6.652397H 3.481439 -1.062967 -8.642685H 2.810109 2.762281 -6.782418H 2.571075 -2.232189 -6.613727C 2.251286 1.012655 -5.639038C 2.188033 -0.387870 -5.575062C 1.632073 -0.812066 -4.235257H 0.621910 -1.236017 -4.338031N 3.784793 1.537631 -8.959909O 4.071292 2.722267 -8.859589O 3.977809 0.867045 -9.955571H 2.239906 -1.587878 -3.754288C 1.637893 0.494202 -3.486537N 1.770989 1.553289 -4.437339N 0.807923 2.124534 -1.950815C 0.946388 0.736454 -2.282867C 1.820539 2.865804 -4.093801O 2.203322 3.774778 -4.829092C 0.249031 2.393364 -0.632227H 0.132539 3.469725 -0.524640H -0.708016 1.879932 -0.540091H 0.920747 1.997836 0.134622O 0.536923 -0.112310 -1.493033C 5.942901 2.172734 -5.085123C 5.822980 2.823495 -6.295271C 5.309550 4.130622 -6.372666C 4.942498 4.763946 -5.213825H 6.301016 1.153712 -4.998801H 6.104035 2.315124 -7.211668H 5.166369 4.614770 -7.329397H 4.499821 5.751042 -5.171112N 5.107163 4.133290 -4.033481C 5.555501 2.854505 -3.926447N 5.480431 2.377338 -2.636172C 5.393748 3.628801 -1.814393C 4.644896 4.618858 -2.717374S 3.928927 1.290210 -2.673720H 6.433539 3.981564 -1.762998H 4.962211 5.651129 -2.563412H 3.549283 4.558728 -2.694307C 4.179512 3.082163 2.269673C 3.224639 3.422745 1.316995C 3.587019 3.605468 -0.015480C 4.918871 3.451947 -0.399382C 5.875599 3.129232 0.565923C 5.511682 2.936292 1.892634H 3.888940 2.941161 3.305748H 2.187831 3.554790 1.609631H 2.827093 3.883997 -0.739676H 6.916205 3.023307 0.266661H 6.265512 2.681505 2.630422C 4.741550 -0.243023 -2.056018H 3.893105 -0.938538 -2.082344C 5.226805 -0.091602 -0.622161H 5.665310 -1.031725 -0.273349H 4.397645 0.185797 0.032681H 5.982951 0.693095 -0.550341C 7.658466 -1.404192 -5.029016C 6.298081 -1.686262 -5.207385C 5.388627 -1.339447 -4.225952C 5.788818 -0.695717 -3.046837C 7.142772 -0.420293 -2.886166C 8.081010 -0.769884 -3.862049H 5.986172 -2.177221 -6.123274H 4.334391 -1.547503 -4.383596H 7.488311 0.089379 -1.992996H 9.127233 -0.539911 -3.699492O 8.475620 -1.784833 -6.044843C 9.854123 -1.519723 -5.907237H 10.328618 -1.897599 -6.812345H 10.274603 -2.033182 -5.034496H 10.047250 -0.443775 -5.817683C 1.291361 3.167178 -2.694221O 1.315521 4.327465 -2.29837718    rac-36   C -1.155404 -1.544150 -0.195042C -0.761455 -0.071165 -0.284956C 0.761455 0.071165 -0.284956C 1.155404 1.544150 -0.195042S -2.965554 -1.778657 -0.153785S 2.965554 1.778657 -0.153785O -1.269729 0.669118 0.794603O 1.269729 -0.669118 0.794603H -0.721708 -2.120871 -1.015209H -0.778624 -1.948422 0.746176H -1.125719 0.336942 -1.245509H 1.125719 -0.336942 -1.245509H 0.721708 2.120871 -1.015209H 0.778624 1.948422 0.746176H -3.205849 -1.334223 -1.398613H 3.205849 1.334223 -1.398613H -2.201579 0.429239 0.896118H 2.201579 -0.429239 0.89611834    (–)-glionitrin (1)   C -2.529395 -1.980090 -3.319355C -2.803575 -1.536377 -2.036817C -4.088318 -1.551995 -1.483826C -5.156756 -2.013698 -2.233994C -4.926295 -2.464579 -3.533899C -3.632155 -2.438376 -4.034218C -4.045400 -1.053045 -0.059341C -2.616738 -0.504681 0.071453N -1.908499 -1.014797 -1.086115C -0.564649 -0.830685 -1.176818C -0.019233 -0.085997 0.047053N -0.534553 -0.665493 1.262370C -1.888387 -0.898224 1.349494O 0.124219 -1.184621 -2.117804O -2.462442 -1.318845 2.330229C 0.232019 -0.611194 2.503859C 1.516968 -0.074874 -0.007107O 1.989135 0.623783 -1.117658S -2.641127 1.359824 0.101675S -0.584314 1.691825 -0.230567H -1.535054 -1.974239 -3.743907H -6.159381 -2.029082 -1.820593H -5.726603 -2.835308 -4.160931H -4.799352 -0.291303 0.152192H -4.176564 -1.860310 0.668981H -0.395997 -1.040668 3.281844H 1.151023 -1.193250 2.419543H 0.470498 0.426084 2.760899H 1.906171 0.430086 0.878824H 1.845445 -1.124553 0.008888H 1.707265 0.126696 -1.900195N -3.405924 -2.922950 -5.408281O -4.374404 -3.316858 -6.030593O -2.265704 -2.898165 -5.82961616   rac-S32    C 0.726091 0.244865 -1.107097C 1.541333 -0.267755 0.082637S 0.947915 0.418959 1.659863S -0.947915 -0.418959 1.659863C -1.541333 0.267755 0.082637C -0.726091 -0.244865 -1.107097O 1.361891 -0.097699 -2.320943O -1.361891 0.097699 -2.320943H 0.716961 1.344462 -1.059722H 1.526295 -1.361068 0.126547H 2.576872 0.063833 -0.032911H -2.576872 -0.063833 -0.032911H -1.526295 1.361068 0.126547H -0.716961 -1.344462 -1.059722H 0.965190 -0.910336 -2.658016H -0.965190 0.910336 -2.65801636   S33   C -2.578192 -1.761276 -3.391791C -2.795146 -1.485667 -2.052233C -3.926720 -1.932695 -1.363575C -4.897279 -2.666637 -2.024394C -4.722102 -2.956803 -3.377078C -3.576100 -2.504826 -4.015613C -3.819918 -1.536550 0.085412C -2.723999 -0.451261 0.053661N -1.980035 -0.746491 -1.163411C -0.644458 -0.537611 -1.309686C 0.064720 0.157388 -0.141138N -0.529121 -0.192795 1.134899C -1.855613 -0.464018 1.301157O -0.022918 -0.889206 -2.298092O -2.383371 -0.687415 2.375758C 0.297329 -0.077915 2.334618C 1.539489 -0.287424 -0.204693O 1.650591 -1.669487 -0.014976S -3.456892 1.245652 -0.103941S -0.014828 2.000929 -0.391903N -3.401811 -2.824571 -5.443963O -4.281294 -3.467375 -5.986812O -2.390327 -2.425841 -5.988626H -1.699305 -1.432567 -3.927014H -5.779852 -3.016841 -1.500184H -5.450699 -3.525540 -3.940065H -4.748517 -1.162772 0.515527H -3.481220 -2.380879 0.696788H -0.355324 -0.207170 3.194425H 1.065064 -0.854413 2.331271H 0.759376 0.912997 2.370190H 1.936437 0.026667 -1.174358H 2.104071 0.215623 0.581825H 1.414304 -2.086948 -0.854148H -4.061437 1.179304 1.094298H 1.035220 2.049408 -1.228526
